# Substituent effects on the stability of the four most stable tautomers of adenine and purine†

**DOI:** 10.1039/c9ra04615a

**Published:** 2019-10-02

**Authors:** Halina Szatylowicz, Anna Jezuita, Paulina H. Marek, Tadeusz M. Krygowski

**Affiliations:** Faculty of Chemistry, Warsaw University of Technology Noakowskiego 3 00-664 Warsaw Poland halina@ch.pw.edu.pl; Faculty of Chemistry, Opole University Oleska 48 45-052 Opole Poland; Faculty of Chemistry, University of Warsaw Pasteura 1 02-093 Warsaw Poland

## Abstract

Substituent effects at the C2-, C8- and N-positions of adenine and purine in their four the most stable tautomers are studied by means of B97D3/aug-cc-pvdz computation applying substituents of varying electronic properties: NO_2_, CN, CHO, Cl, F, H, Me, OMe, OH and NH_2_. The substituent effect is characterized by the substituent effect stabilization energy (SESE) and substituent Hammett constant *σ*. For adenine, SESE is obtained with purine as the reference system. Additionally, for both adenine and purine, SESE characteristics are estimated with benzene, imidazole and amino-pyrimidine as reference systems, when possible, taking into account substitution in topologically equivalent positions. The role of a C6–NH_2_ group in adenine in modifying the substitution effect is observed and discussed. Additionally, the proximity effect for some asymmetric substituents (*e.g.* CHO, OMe) is recognized and meticulously analyzed.

## Abstract 2

Substituent effects at the C2-, C8- and N-positions of adenine and purine in their four the most stable tautomers are examined using substituents of varying electronic properties: NO_2_, CN, CHO, Cl, F, H, Me, OMe, OH and NH_2_.

## Graphical abstract

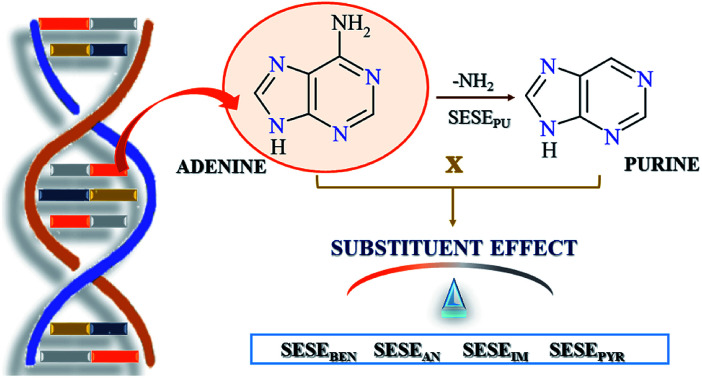

## Introduction

**Scheme 1:**
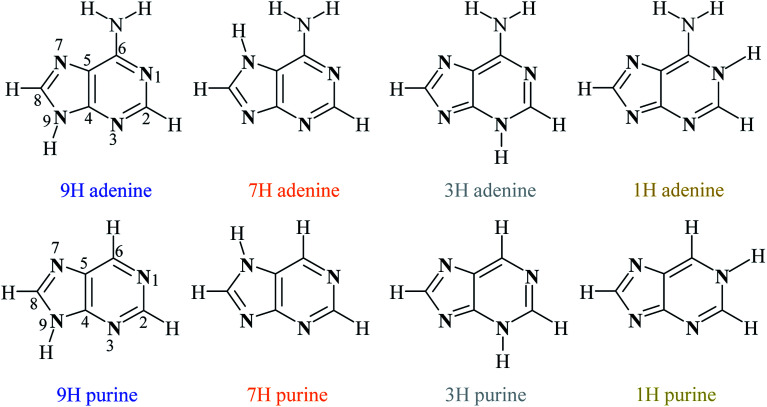
Structures and numbering scheme of four the most energetically favorable adenine and purine tautomers.

Adenine is one of five building blocks of DNA and RNA nucleic acids^1^ and is a simple amino derivative of purine. The low energetic prototropic abilities of adenine result from the presence of four endocyclic nitrogen atoms, one of which is bonded to a hydrogen atom. The proton may change its position, which leads to a formation of four low-energy tautomers. Among them, the 9H tautomer (Scheme 1) is the most stable, which has been confirmed both theoretically and experimentally; for details see Raczyńska *et al.*^2^ and references therein. In contrast, there are also possibilities of tautomeric forms in which the exocyclic amino-group or carbon atoms are involved, but these tautomeric rearrangements are highly energetically unfavorable.^3^ Proton transfers in tautomeric rearrangements are always accompanied by the migration of double bonds, that is, changes in π-electron structure.^4^ As a result, it can be expected that the different sensitivity of a given position to the substituent effect depends on the type of tautomer considered. This is the reason why the impact of the substituent effect on various properties of purine or adenine systems has been studied. More than half a century ago, the effect of substituent on the hydrogen bonding of adenine derivatives was studied using IR technique.^5^ This allowed to determine the association constants between variously substituted 1-cyclohexyluracil derivatives and 9-ethyladenine and to compare them with the corresponding data for thymine derivatives. Almost at the same time, substituent effects on the optical activity of some purine nucleosides were studied.^6^ The effect of exocyclic substituents of purine bases on the DNA curvature was also reported.^7,8^ The substituent effects at the major and minor grooves of the double helix on the hydration and stability of Z- *versus* B-DNA were also investigated.^9^ Subsequent studies concerned the influence of the substituent on process of pairing and polymerase recognition of simple unnatural base pairs.^10^

ADF computational package was used to study substituent effects on nanoswitches based on DNA base pairs^11^ and on the NMR shielding constants of complexes based on AT and AU base pairs.^12^ For AT and GC base pairs, systematic studies of the substituent effects on the individual hydrogen bond energies were carried out.^13^ Recently, the effect of the substitution of halogen atoms at the position 8 in adenine 9H, 7H, 3H and 1H tautomers on their relative stability, vibrational frequencies and Raman spectra, were studied by means of DFT at the B3LYP/6-311+G(d,p) level of theory.^14^ It was found that the stability of tautomers of substituted adenine molecules strongly depends on solvation. It was also found^15^ that, for 20 different substituents in the position 8 of adenine, electrostatic potentials at the bonding atoms of monomers correlate nicely with interaction energies of adenine–thymine pairs.

All of the above-mentioned papers present research results indicating that many important properties of nucleic acid bases significantly depend on the substituent attached to their molecules. In addition, it should be mentioned that in most cases observed changes of the studied systems were not related to the electron properties of substituents. It is well known that substituents can exert either electron withdrawing or electron donating properties. Thus, they may influence substituted systems in a different way and have a fundamental impact on the nature and consequences of the substituent effect.^16^

Classically, the quantitative characteristics of the substituent effect is described by the Hammett substituent constants *σ* (ref. 17 and 18) or their modifications.^19^ Positive values of *σ* denote that the substituent is electron-attractive, while negative ones stand for electron-donating properties. Substituent constants have great utility in the so-called Hammett equation, where physicochemical properties of the substituted systems *P*(X) are linearly related to the substituent constants *σ*(X), as shown in eqn (1)

1 *P*(X) = *ρσ*(X) + const

where *ρ* is termed reaction constant and characterizes sensitivity of the property *P*(X) on influence of the substituent X. For exhaustive reviews see ref. 16 and 20–23.

Dynamic development of applications of quantum chemistry in chemical research^24,25^ has resulted in the introduction of several models based on quantum chemistry describing substituents effects. One of them is the use of homodesmotic reactions^26–28^ which for disubstituted species is presented by eqn (2)

2 X–R–Y + R → R–X + R–Y

The difference between energy of products and substrates, named SESE (substituent effect stabilization energy),^29^ describes the substituent effect in energetic scale. A positive SESE value means that the intramolecular interactions between the substituents X and Y stabilize the X–R–Y system. This descriptor of the substituent effect takes into account all interactions in X–R–Y systems. Another quantum chemistry concept of the substituent effect is based on atomic charges. It is known that due to polarity of X–C bonds in substituted carbocyclic systems, atomic charges of substituents (in *e.g.* benzene derivatives) do not correlate with substituent constants.^30^ However, when instead of the charge of the X substituent, *q*(X), the sum of charges in the substituent X and the *ipso* carbon atom are taken into account, the correlations with the constant substituents are very good.^31,32^ This descriptor is named cSAR(X) (charge of the substituent active region) and independently of the atomic charge assessment method, it correlates well with substituent constants.^33^ Recently, it has been shown that in the case of series of disubstituted benzene, cyclohexadiene and bicyclooctane derivatives, both descriptors, SESE and cSAR(X), modeled theoretically correlate well with traditional substituent constants.^29,34–36^ It is important to note that cSAR(X) not only describes electron attracting/donating properties of the substituent, but also informs how these properties depend on its position in the substituted molecule. Another fruitful theoretical tool to study the substituent effect is the analysis of molecular electrostatic potential (MESP), which was recently published by Remya and Suresh^37^ and references therein.

Adenine and purine are π-electron systems. As mentioned above, four endocyclic nitrogen atoms with the possibility of prototropic rearrangements give four tautomers. There are several questions that we should ask here: (i) how substituent effect descriptors based on quantum chemistry work in such complex molecules and whether they differ from benzene systems; (ii) how the substituent effect works in four different tautomers; (iii) whether the substituent can change the stability of the tautomer; (iv) how far the amino group in adenine may differentiate the substituent effect comparing to purine.

## Methodology

The research objects were C2–X, C8–X and N–X substituted derivatives (where X = NO_2_, CN, CHO, Cl, F, H, Me, OMe, OH and NH_2_) of the four most stable adenine and purine tautomers (Scheme 1). In the case of asymmetrical substituents (CHO, OMe and OH), both rotamers were taken into account, the one with the lower energy was used for SESE calculations. For all studied systems, optimization without any symmetry constraints was performed using the Gaussian 09 package.^38^ According to the results of our previous research,^39^ the DFT-D methods were used, that is functionals B97D3 (ref. 40) and ωB97XD,^41^ both with Dunning's aug-cc-pvdz basis set.^42^ To confirm that calculated structures correspond to the minima on the potential energy surface the vibrational frequencies were calculated at the same level of theory. No imaginary frequencies were found for the obtained systems. Since the results of calculations obtained on both calculation levels are very similar, in the further part of the work the first ones (B97D3/aug-cc-pvdz) are presented and discussed.

## Results and discussion

Adenine and purine are closely related to each other structurally, differing only in the amino group in the position 6 of purine. Their four most stable tautomers are realized by migration of the proton between the nitrogen atoms in positions 9, 7, 3 and 1 and are named 9H, 7H, 3H and 1H, respectively (Scheme 1).

**Fig. 1:**
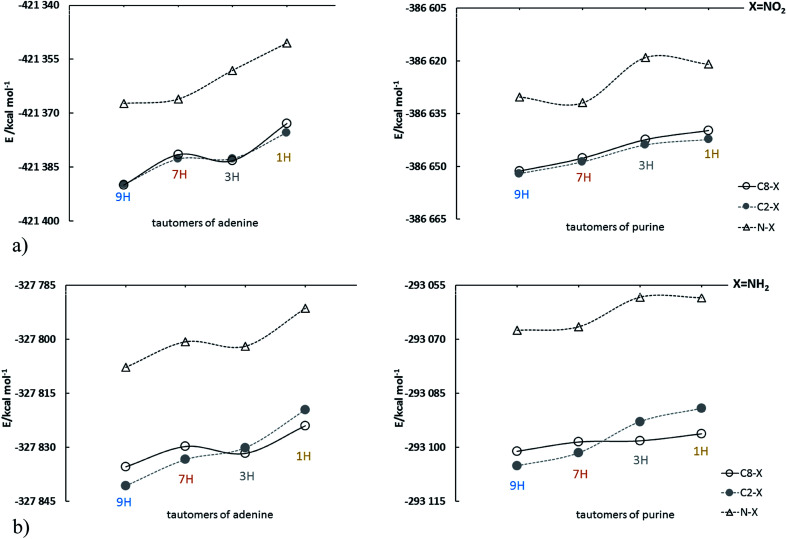
Energy changes of adenine and purine tautomers in sequence: 9H, 7H, 3H, 1H for (a) NO_2_ and (b) NH_2_ substituted derivatives; for clarity, the points are connected by a solid or dashed line.

The energy changes due to the presence of the most electron-accepting and electron-donating substituents (NO_2_ and NH_2_, respectively) in purine and adenine are presented in Fig. 1; the data are shown in the order: 9H, 7H, 3H and 1H.

For all presented derivatives, it is clearly visible that the substitution of the C–X type causes a decrease in the energy of the system as compared to the substitution at the nitrogen atom (N–X). Moreover, significant differences can be noticed when looking at the nitro and amino derivatives substituted at the C2 and C8 positions. In the case of the C2- and C8-nitro purine derivatives tautomer energies increase monotonically, while for adenine derivatives the lines are irregular and intersect between 7H and 3H tautomers. For amino derivatives of purine and adenine energy changes are similar. In both cases substitution at C2 leads to lower stability of 3H and 1H tautomers than in the case of C8 substitution. This means that the amino group affects energy of substituted tautomer derivatives strongly enough to modify the sequence of decreased stability. For the N–X systems shown in Fig. 1, the most significant changes in tautomer stability are observed for nitro-substituted purine derivatives, discussed below.

Relative energy values, *E*_rel_, (with respect to the 9H tautomer 9H), for studied C8–X, C2–X and N–X substituted derivatives of adenine and purine tautomers can be found in Tables S1–S3.† Firstly, obtained results show that in all cases, the 9H tautomer is the most stable for both adenine and purine derivatives. The only exception is 7-NO_2_-purine, more stable than 9-NO_2_-purine by *ca.* 1.7 kcal mol^−1^, and the reason is a formation of a weak C–H⋯O intramolecular hydrogen bond in the former. The mean values of average relative stability in substituted adenines is ∼10 kcal mol^−1^, whereas for purine series is only ∼7.3 kcal mol^−1^. Undoubtedly, this difference results from the presence of the NH_2_ group at the 6-position in adenine, which additionally leads to greater variability of relative energies in adenine series. Secondly, we noted that for both adenine and purine derivatives, in both C8 and C2 substituent positions, the *E*_rel_ values for tautomers 7H have the smallest range of variation (2.91 and 0.99 kcal mol^−1^ for adenine, and 1.15 and 0.59 kcal mol^−1^ for purine, respectively). The direct neighborhood of the C8 substituent in 9H and 7H tautomers is almost identical: one nitrogen atom with a lone pair in the molecular plane (2p_*xy*_) and one electron on the perpendicular 2p_*z*_ orbital and second nitrogen (in N–H group) with 2p_*z*_ electron pair. Thus, even if there are some proximity effects, they are similar, and the resulting *E*_rel_ differences are small. For C2–X substitution the ranges are the smallest, since there are no proximity effects that appear in the vicinity of C8. No such situations are found in other cases, with much larger *E*_rel_ ranges, generally between 8.07 kcal mol^−1^ (3H, C8–X substitution in adenine) and 13.21 kcal mol^−1^ (1H, C2–X substitution in adenine). In addition, it is worth noting that the substituents, except for the N–X cases with X = NO_2_, do not change the stability of purine tautomers (Tables S2 and S3†), which decreases in order of H9, H7, H3 and H1.

### Substituent effect (SE) descriptor based on relative stability of tautomer

The use of relative energy (*E*_rel_) differences with respect to the unsubstituted systems (X = H) allow for easier analysis of the SE. For 7H tautomers, this difference can be written as the energy of the homodesmotic reaction, named SESE_rel_:3
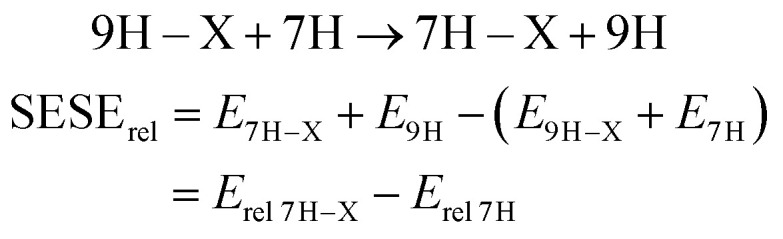


**Fig. 2:**
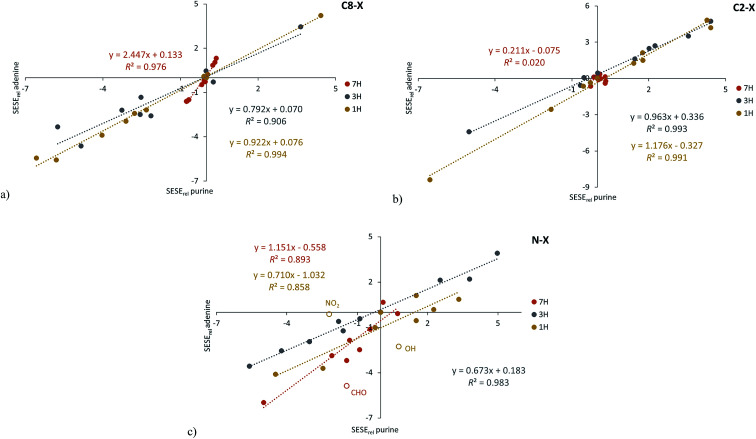
Correlations between SESE_rel_ values (in kcal mol^−1^) of adenine and purine tautomers for C8–X (a), C2–X (b) and N–X (c) positions of substituents; empty circles indicate substituents not included in the regression.

Analogous equations can be written for 3H and 1H tautomers. The obtained SESE_rel_ values for 7H, 3H and 1H substituted adenine and purine tautomers are gathered in Tables 1 and 2, while the comparison of the substituent effect observed for adenine and purine derivatives is shown in Fig. 2; in addition, the statistics of obtained linear regressions are collected in Table S4.†

**Table:** The obtained SESE_rel_ (in kcal mol^−1^) values for 7H, 3H and 1H adenine and purine tautomers substituted at the C8 and C2 positions (Scheme 1)

	Adenine						Purine					
	C8–X			C2–X			C8–X			C2–X		
	7H	3H	1H	7H	3H	1H	7H	3H	1H	7H	3H	1H
NO_2_	1.32	−0.28	0.22	−0.09	0.08	−2.55	0.38	0.27	0.05	0.04	−0.53	−1.81
CN	1.05	−2.58	−2.17	0.36	2.69	1.49	0.33	−2.14	−2.32	0.09	2.25	1.78
CHO	0.84	3.45	4.22	−0.21	−4.42	−8.39	0.25	3.65	4.43	0.32	−5.02	−6.54
Cl	−0.19	−2.5	−2.95	0.17	2.48	2.12	−0.09	−2.56	−3.11	−0.01	2.02	1.76
F	−0.47	−4.62	−5.57	0.12	4.72	4.82	−0.18	−4.84	−5.80	−0.14	4.43	4.28
**H**	**0.00**	**0.00**	**0.00**	**0.00**	**0.00**	**0.00**	**0.00**	**0.00**	**0.00**	**0.00**	**0.00**	**0.00**
Me	−0.26	0.45	0.10	−0.02	−0.57	−0.64	−0.04	−0.01	−0.09	0.12	−0.66	−0.55
OMe	−1.59	−1.33	−2.41	−0.63	0.46	−0.34	−0.77	−2.53	−2.78	−0.27	0.01	−0.27
OH	−1.58	−2.21	−3.90	−0.39	1.61	1.25	−0.74	−3.26	−4.03	0.30	1.46	1.42
NH_2_	−1.48	−3.32	−5.44	0.14	3.52	4.21	−0.67	−5.74	−6.56	0.30	3.55	4.42
Range	2.91	8.07	9.79	0.99	9.14	13.21	1.15	9.39	10.99	0.59	9.44	10.96
Average	−0.24	−1.29	−1.79	−0.05	1.06	0.20	−0.15	−1.72	−2.02	0.08	0.75	0.45
SD	1.08	2.29	3.00	0.29	2.57	3.75	0.44	2.76	3.24	0.19	2.66	3.17

**Table:** The obtained SESE_rel_ (in kcal mol^−1^) values for 7H, 3H, 1H adenine and purine tautomers substituted at the N–X positions

N–X	Adenine			Purine		
	7H	3H	1H	7H	3H	1H
NO_2_	−5.97	2.12	−0.13	−4.99	2.54	−2.20
CN	−3.20	3.90	0.87	−1.44	4.97	3.33
CHO	−4.87	2.20	0.18	−1.11	3.79	2.27
Cl	−1.84	−0.61	−1.02	−1.32	−1.79	−0.23
F	−2.88	−2.56	−4.10	−2.08	−4.22	−4.48
**H**	**0.00**	**0.00**	**0.00**	**0.00**	**0.00**	**0.00**
Me	0.67	−0.44	1.11	0.10	−0.89	1.51
OMe	−2.47	−1.95	−3.71	−0.89	−3.03	−2.46
OH	−1.12	−3.57	−2.28	−0.45	−5.59	0.77
NH_2_	−0.09	−1.23	−0.56	0.73	−1.60	1.52
Range	6.64	7.47	5.21	5.71	10.56	7.82
Average	−2.18	−0.21	−0.96	−1.15	−0.58	0.00
SD	2.04	2.34	1.73	1.59	3.45	2.41

To reveal the influence of the presence of the amino group on the substituent effect the dependencies of SESE_rel_(adenine) on SESE_rel_(purine) are analyzed (Fig. 2 and Table S4†). Three substitution positions are shown: C8–X, C2–X and N–X. For the C–X series, a slope greater than 1.00 has been found for derivatives of 7H C8–X and 1H C2–X, its values are 2.45 (±0.14) and 1.176 (±0.040), respectively. In both cases NH is located near the amino group of adenine and a substituted carbon atom. In other cases, the slopes are lower than 1.0. In all C–X cases *R*^2^ > 0.9, with the exception of 7H C2–X systems.

Relationships between SESE_rel_ values (adenine *vs.* purine) of N–X substituted tautomers do not lead to unequivocal conclusions. However, when some of the most deviating points are omitted (empty circles in Fig. 2c), the reasonable correlations can be noticed. The slope is the highest for 7H (1.15 ± 0.15, without X = CHO) then 0.71 (±0.12) for 1H (without X = NO_2_ and OH) and finally 0.673 (±0.031, *R*^2^ = 0.983) for 3H substituted tautomers.

Thus, the comparison of the substituent effect, based on SESE_rel_ model, in adenine with that present in purine, allows to conclude that more significant effect is observed in adenine when substituent is attached to the 5-membered ring.

Hammett *σ* constants are the most commonly used to describe the electron donating/accepting properties of the substituents. Their application to the data in Tables 1 and 2, this is SESE_rel_*vs. σ* regression (see Table S5†), leads to the observation that in the case of C8–X-substituted 7H tautomer, both adenine and purine, the determination coefficients are 0.878 and 0.810, respectively, with a large difference in the slope values: 2.206 (±0.291) and 0.855 (±0.147), respectively. This difference in the slopes can be related to a significant influence of the amino group in adenine on charge transfer from the substituent to the molecular moiety. It is important to note that in addition to these results, all other linear regressions with substituent constants always lead to *R*^2^ lower than 0.3 (for 3H C8–X purine series). These results provide important information that the model of interactions which describes well the substituent effect in benzene derivatives fails completely in some cases of adenine and purine derivatives. This problem will be discussed in more details later.

### Comparison of substituent effects from positions C8 and C2

Differences between the energies of pairs of the same molecule with different substitution positions allow to compare the substituent effect from these positions. For all adenine and purine tautomer series with substituents at the C8–X and C2–X positions

4 Δ*E*_8−2_ = *E*_C8–X_ − *E*_C2–X_

where *E*_C8–X_ and *E*_C2–X_ are the energies of the mono-substituted molecule at positions C8–X and C2–X, respectively.

It should be noticed that the close vicinity of C2 and C8 substitution positions in adenine and purine is topologically identical. The obtained Δ*E*_8−2_ values for all adenine and purine tautomers are given in Tables 3 and 4, respectively.

**Table:** Energy difference values, Δ*E*_8−2_ (eqn (4), in kcal mol^−1^), of the studied C8–X and C2–X substituted adenine (AD) tautomers

AD	Δ*E*_8−2_				Average	Range
	9H	7H	3H	1H		
NO_2_	−0.16	1.25	−0.52	2.61	0.79	3.12
CN	0.01	0.71	−5.26	−3.65	−2.05	5.96
CHO	−5.70	−4.64	2.17	6.92	−0.31	12.62
Cl	2.95	2.59	−2.02	−2.12	0.35	5.07
F	7.45	6.86	−1.89	−2.94	2.37	10.39
**H**	**0.00**	**0.00**	**0.00**	**0.00**	**0.00**	**0.00**
Me	−1.19	−1.43	−0.16	−0.45	−0.81	1.26
OMe	2.75	1.79	0.96	0.69	1.55	2.07
OH	4.45	3.26	0.82	−0.70	1.96	5.15
NH_2_	5.24	3.62	−1.60	−4.41	0.71	9.65
Range	13.15	11.51	7.43	11.33		
Average	1.58	1.40	−0.75	−0.41		
SD	3.77	3.11	2.08	3.33		

**Table:** Energy difference values, Δ*E*_8−2_ (eqn (4), in kcal mol^−1^), of the studied C8–X and C2–X substituted purine (PU) tautomers

PU	Δ*E*_8−2_				Average	Range
	9H	7H	3H	1H		
NO_2_	0.70	1.04	1.49	2.56	1.45	1.86
CN	0.69	0.93	−3.70	−3.41	−1.37	4.63
CHO	−4.36	−4.43	4.31	6.61	0.53	11.04
Cl	2.16	2.08	−2.42	−2.71	−0.22	4.87
F	6.19	6.15	−3.08	−3.89	1.34	10.08
**H**	**0.00**	**0.00**	**0.00**	**0.00**	**0.00**	**0.00**
Me	−1.62	−1.78	−0.96	−1.15	−1.38	0.81
OMe	1.22	0.72	−1.32	−1.29	−0.17	2.54
OH	3.16	2.13	−1.56	−2.29	0.36	5.45
NH_2_	4.01	3.03	−5.28	−6.97	−1.30	10.98
Range	10.55	10.58	9.59	13.58		
Average	1.21	0.99	−1.25	−1.25		
SD	2.95	2.82	2.73	3.73		

Some interesting points resulting from the comparison of the data presented in Tables 3 and 4 can be discussed. In both series (adenine and purine), for 7H and 9H tautomers the differences are positive (except for CHO and Me derivatives), and for 1H and 3H are mostly negative, what can be seen in the averaged data for all substituents. The mean values of Δ*E*_8−2_ averaged for all four tautomers of adenine and purine derivatives do not correlate with each other (*R*^2^ = 0.421), while the range values do very well (*R*^2^ = 0.954), as shown in Fig. S1.† This can be interpreted as follows: the nature of interactions caused by the substituent is different in purine and adenine, but the strength of the interactions is alike.

**Fig. 3:**
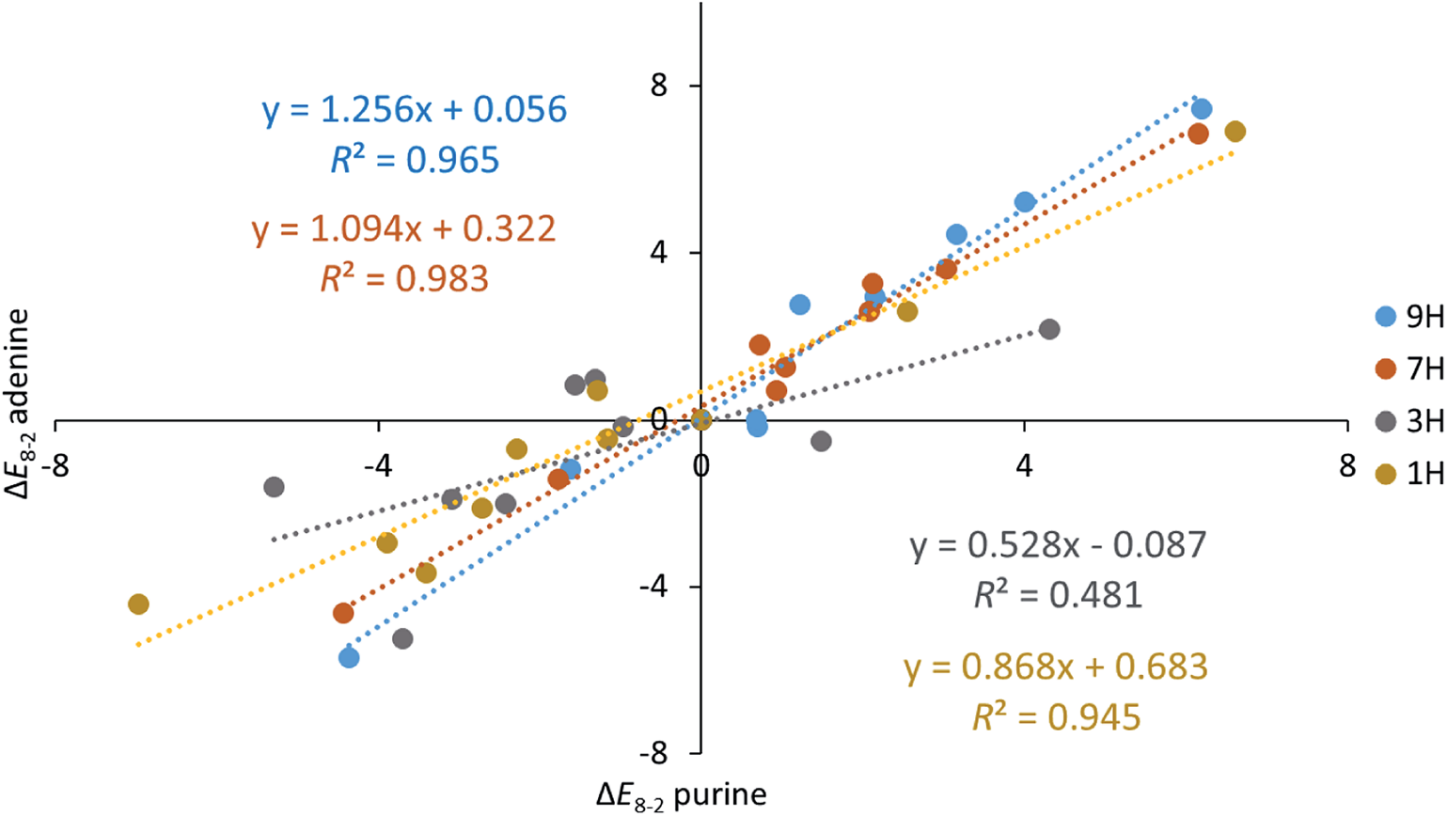
Relations between the Δ*E*_8−2_ values (in kcal mol^−1^) obtained for adenine and purine tautomers.

Fig. 3 presents a comparison of the obtained Δ*E*_8−2_ values for adenine and purine – in other words – shows how the amino group in adenine influences interactions in the molecules. As mentioned above, the closest vicinity of positions C2 and C8 (Scheme 1) is similar. Either there are two nitrogen atoms with one π-electron and a lone pair in the plane of the molecule, or there is one N-atom of this kind and NH group, with 2p_*z*_ electron pair. Obviously, in the first case, in N–C2–X–N fragment the nitrogen atoms can act as hydrogen bond acceptors, whereas in NH–C8–X–N they may play a role of a donor and an acceptor, respectively.

Despite the similarity of the closest vicinity for substituents in the C2–X and C8–X positions and the fact that their energy difference is considered, differences appear in Δ*E*_8−2_ for tautomers of adenine and purine. For 1H and 3H tautomers interactions are stronger in purine derivatives, and for 7H and 9H they are stronger in adenine series (Fig. 3). It is important to say that except for the 3H tautomer in all other cases the substituent effects are well linearly correlated with each other (*R*^2^ > 0.945) and slopes variety (between 0.868 and 1.256) could indicate dependence of the systems sensibility on the position of the substituent. Undoubtedly, the difference should be related to the presence of NH_2_ group in adenine.

### SESE model based on adenine and purine reactions

Energies of substituted purine (PU) and adenine (AD) can be applied for homodesmotic reactions:5
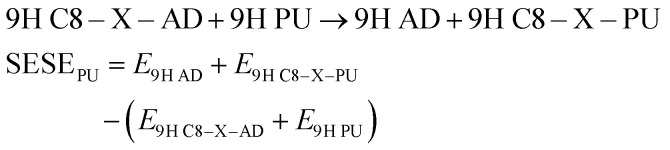


For C2–X substituted systems of the 9H tautomer we can write:

6 SESE_PU_ = *E*_9H AD_ + *E*_9H C2–X–PU_ − (*E*_9H C2–X–AD_ + *E*_9H PU_)

Analogous equations can be written for the remaining substituted tautomers. The results of the SESE_PU_ approach are gathered in Table 5. Positive SESE_PU_ values indicate greater stability of substrates than reaction products.

**Table:** Obtained SESE_PU_ values for C2 and C8 substituted adenine tautomers

SESE_PU_	9H		7H		3H		1H	
	C8–X	C2–X	C8–X	C2–X	C8–X	C2–X	C8–X	C2–X
NO_2_	2.51	1.65	1.58	1.79	3.05	1.05	2.34	2.39
CN	1.82	1.14	1.10	0.87	2.26	0.70	1.67	1.42
CHO	1.86	0.52	1.27	1.06	2.05	−0.09	2.06	2.36
Cl	−0.03	0.77	0.08	0.59	−0.09	0.30	−0.19	0.41
F	−0.45	0.81	−0.16	0.56	−0.68	0.51	−0.69	0.26
**H**	**0.00**	**0.00**	**0.00**	**0.00**	**0.00**	**0.00**	**0.00**	**0.00**
Me	−0.61	−0.19	−0.39	−0.04	−1.08	−0.28	−0.81	−0.10
OMe	−1.73	−0.19	−0.90	0.17	−2.93	−0.64	−2.09	−0.12
OH	−1.58	−0.29	−0.73	0.41	−2.63	−0.44	−1.71	−0.12
NH_2_	−1.79	−0.56	−0.98	−0.39	−4.21	−0.53	−2.91	−0.35
Range	4.30	2.22	2.56	2.18	7.26	1.69	5.26	2.74
Average	0.00	0.37	0.09	0.50	−0.43	0.06	−0.23	0.62
SD	1.57	0.72	0.93	0.63	2.40	0.56	1.79	1.05

Observed SESE_PU_ values are usually negative (*i.e.* destabilizing interactions) for electron-donating substituents and positive for the electron-attracting ones. In the case of strongly interacting substituents, for the C8–NH_2_ and C2–NH_2_ systems the average SESE_PU_ values (in kcal mol^−1^) are −2.47 and −0.46, whereas 2.37 and 1.72 for NO_2_ derivatives, respectively. These differences in the energy values of the substituent effects in adenine, in comparison with purine, can be connected to the presence of the amine group at the 6-position in adenine.

**Fig. 4:**
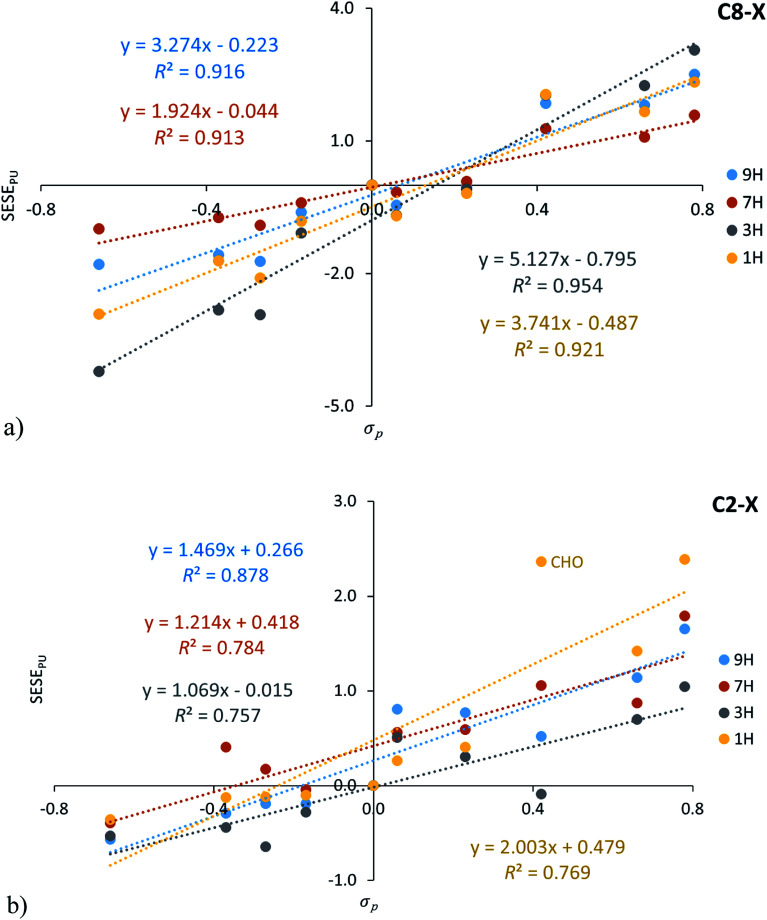
Dependence of SESE_PU_ (in kcal mol^−1^) on substituent constants *σ*_p_ for C8–X (a) and C2–X (b) positions of substituents in adenine systems.

It is interesting to compare the SESE_PU_ values with the traditional substituent effect descriptor, *i.e.* Hammett constants. Its mutual correlations for C8–X and C2–X adenine series are shown in Fig. 4, in addition, statistical details are presented in Table S6† (for both *σ*_p_ and *σ*_m_ relations).

A verification of the obtained interrelationships shows: (i) for C8–X systems SESE_PU_ correlates better with *σ*_p_ than with *σ*_m_, (ii) the same applies to C2–X series of 3H and 1H tautomers, when X = CHO systems are excluded, whereas (iii) for C2–X derivatives of 9H and 7H tautomers, a better correlation of SESE_PU_ with *σ*_m_ than with *σ*_p_ can be found.

Since SESE_PU_ values are based on differences in the energy values of the analogically substituted adenine and purine derivatives, obtained values inform about changes related to the amino group present in adenine tautomers. In the 7H tautomer the 2p_*z*_ electron pair residing at N7 atom hinders charge transfer between electron-attracting substituents at the C8 position and the amino group at C6 (Scheme 1). This can be confirmed by the smallest value of the slope for this series. For 9H tautomers, where there is no such problem with the charge transfer, the interaction is stronger, hence the slope is steeper. The situation is more complex for 3H and 1H tautomers and requires further study. However, it should be emphasized that for C8–X derivatives of 3H and 1H tautomers, the substituent effect is stronger than that observed for 9H and 7H, and the strongest occurring for the 3H C8–X adenine series (slope = 5.08 ± 0.40, Table S6†).

### The proximity effects

The proximity effect is associated with interactions of the functional group/substituent with the neighboring groups or even with the hydrogen atom, and in some cases with lone electron pairs. Due to this type of interaction, the electronic properties of the group/substituent can be significantly modified. Applications of the substituent effect descriptors estimated for *para*- and *meta*- substituted benzoic acids, such as the *σ* constants, to systems with proximity effects are usually not resulting with satisfactory correlations or even fail.^43–45^ Sayyed and Suresh^46^ showed that the use molecular electrostatic potential and a molecular fragment approach in conjunction with a rotation experiment on the COOH group of benzoic acid allowed quantifying the effects of proximity.

**Scheme 2:**
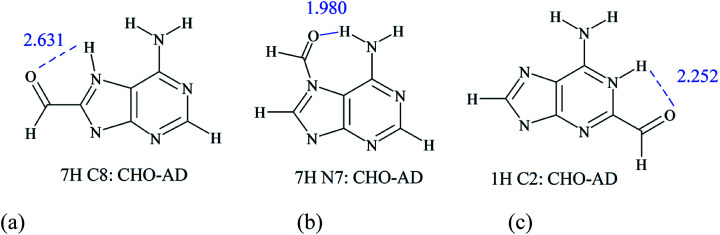
CO⋯HN interactions in selected formyl-adenine systems, with O⋯H distance marked in blue.

In the case of substituted adenine derivatives, the proximity effects appear frequently, and some examples are presented in detail below. As mentioned above, for C2- or C8- substituted adenines, depending on the type of tautomers, the substituent can be in direct neighborhood of the lone pair on the nitrogen atom in the plane of the molecule (or two such pairs) and the NH with the 2p_*z*_ electron lone pair. Thus, some tautomers of adenine with asymmetric substituents, for example C8–CHO in 7H and 9H and the C2–CHO in 1H and 3H systems, may exist in two conformations. One, with a low energy, when CH (from –CHO) interacts with a lone pair and the CO part with the NH, while in the second conformation, when CHO is rotated by 180°, strong repulsive interactions appear: CO is interacting with the lone pair and CH is interacting with NH. The observed difference in energy between these two rotamers is 6.59, 5.50, 9.53 and 5.92 kcal mol^−1^, respectively. Similar effects are observed for other nonsymmetric substituents, such as X = OH and OMe (Table S7†). These are examples of systems that can be obtained as energy molecule optimization results (local minima) and reveal possible interactions in the case of studied systems. Other substituents in the C2- and C8- position of adenine may have weaker proximity effects, but they still appear, and in many cases worse correlations with substituent constants or other substituent effect descriptors are observed. In this case, the proximity interactions CO⋯HN and CH⋯N (shown in Scheme 2) predominate the classical substituent effect based on resonance and inductive/field interactions. In other cases, this effect is not always significant, but it may contribute to lower values of determination coefficients, as shown in Table S6† and a few tables below.

Different interactions can be noticed in N-substituted adenine derivatives. Here, the proximity effect is mainly observed for N7- and N1- substituted species, where amino group in the 6-position is neighboring to substituents. Good examples of proximity effects are intramolecular hydrogen bonds presented in Scheme 2b.

### SESE model based on reactions with benzene or aniline as references

So far, substituted adenine and purine series, differing only in the NH_2_ group at C6, have been compared. One can ask how different are substituent effects observed in adenine and purine derivatives compared to those in benzene (BEN) and anilines (AN), which are considered classical objects in the SE research.

The corresponding equations are as follows:

7 SESE_BEN_ = *E*_9H AD_ + *E*_X–BEN_ − (*E*_9H C8–X–AD_ + *E*_BEN_)

8 SESE_AN_ = *E*_9H AD_ + *E*_4-X–AN_ − (*E*_9H C8–X–AD_ + *E*_AN_)

Analogous equations can be written for the remaining substituted tautomers of adenine and purines. The obtained SESE_BEN_ and SESE_AN_ values are presented in Tables 6 and S8,† respectively. It should be noted that the difference in values obtained for analogous derivatives of adenine and purines is SESE_PU_.

SESE_BEN_ (in kcal mol^−1^) estimated for adenine (AD) (a) and purine (PU) (b) with benzene as reference

**Table:** 

(a)	SESE_BEN_							
AD	9H		7H		3H		1H	
	C8–X	C2–X	C8–X	C2–X	C8–X	C2–X	C8–X	C2–X
NO_2_	−4.71	−4.87	−6.03	−4.78	−4.44	−4.95	−4.93	−2.33
CN	−4.07	−4.06	−5.12	−4.42	−1.49	−6.75	−1.90	−5.55
CHO	1.90	−3.80	1.06	−3.59	−1.56	0.61	−2.33	4.59
Cl	−2.82	0.13	−2.63	−0.04	−0.33	−2.35	0.13	−1.99
F	−1.85	5.60	−1.38	5.48	2.76	0.87	3.71	0.77
**H**	**0.00**	**0.00**	**0.00**	**0.00**	**0.00**	**0.00**	**0.00**	**0.00**
Me	3.24	2.05	3.50	2.08	2.79	2.62	3.14	2.70
OMe	4.41	7.17	6.01	7.80	5.74	6.71	6.82	7.51
OH	4.06	8.51	5.64	8.90	6.26	6.90	7.95	7.26
NH_2_	2.98	8.21	4.46	8.08	6.30	4.00	8.42	4.00
Range	9.13	13.38	12.03	13.68	10.73	13.65	13.35	13.06
Average	0.31	1.89	0.55	1.95	1.60	0.77	2.10	1.70
SD	3.47	5.22	4.34	5.34	3.74	4.55	4.64	4.29

**Table:** 

(b)	SESE_BEN_							
PU	9H		7H		3H		1H	
	C8–X	C2–X	C8–X	C2–X	C8–X	C2–X	C8–X	C2–X
NO_2_	−7.22	−6.53	−7.61	−6.57	−7.49	−6.00	−7.27	−4.72
CN	−5.89	−5.20	−6.22	−5.29	−3.75	−7.45	−3.57	−6.98
CHO	0.04	−4.32	−0.22	−4.64	−3.61	0.70	−4.39	2.22
Cl	−2.79	−0.64	−2.71	−0.63	−0.24	−2.65	0.32	−2.40
F	−1.40	4.79	−1.22	4.92	3.44	0.36	4.40	0.51
**H**	**0.00**	**0.00**	**0.00**	**0.00**	**0.00**	**0.00**	**0.00**	**0.00**
Me	3.86	2.24	3.90	2.12	3.87	2.90	3.95	2.80
OMe	6.14	7.36	6.91	7.62	8.67	7.35	8.92	7.63
OH	5.63	8.80	6.37	8.50	8.90	7.33	9.67	7.38
NH_2_	4.77	8.78	5.44	8.47	10.51	5.22	11.33	4.36
Range	13.36	15.32	14.51	15.06	18.00	14.80	18.60	14.60
Average	0.31	1.53	0.46	1.45	2.03	0.78	2.34	1.08
SD	4.75	5.79	5.12	5.80	6.09	5.13	6.38	4.83

In almost all cases the obtained values for both SESE_BEN_ and SESE_AN_ are negative for electron-attracting substituents and positive for electron-donating ones. In addition, their absolute values are higher for purine systems than for adenine ones. This can be depicted by the slope of a linear relation of the SESE descriptors (SESE_BEN_ and SESE_AN_) between the values for adenine and purine systems as shown in Table 7. Thus, the amino group of adenine reduces the influence of the substituent effect. Moreover, it can be noticed that this reduction is more visible for substitution at C8–X than at C2–X. In all cases the discussed regressions are characterized by very high determination coefficients (*R*^2^ > 0.95).

**Table:** Slope values, *a*, and determination coefficients, *R*^2^, of SESE_BEN_ and SESE_AN_ relations between values obtained for adenine (AD) and purine (PU) tautomers with substituents at positions C2–X and C8–X

	C8–X		C2–X	
	*a*	*R* ^2^	*a*	*R* ^2^
**SESE**_**BEN**_ **(AD) *vs.* SESE**_**BEN**_ **(PU)**				
9H	0.712 ± 0.058	0.949	0.899 ± 0.026	0.994
7H	0.842 ± 0.031	0.989	0.918 ± 0.025	0.994
3H	0.612 ± 0.022	0.990	0.885 ± 0.023	0.990
1H	0.724 ± 0.019	0.995	0.871 ± 0.062	0.995

**SESE**_**AN**_ **(AD) *vs.* SESE**_**AN**_ **(PU)**				
9H	0.774 ± 0.026	0.991	0.927 ± 0.019	0.997
7H	0.875 ± 0.015	0.998	0.938 ± 0.018	0.997
3H	0.708 ± 0.014	0.997	0.927 ± 0.012	0.998
1H	0.792 ± 0.011	0.998	0.881 ± 0.037	0.986

Both SESE_BEN_ and SESE_AN_ are based on homodesmotic reactions with benzene derivatives as references. Thus, these descriptors, by definition, include a comparison of the substituent effect in heterocyclic systems (adenine or purine) with that realized in traditional benzene series (monosubstituted benzene or aniline derivatives). In the case of *para*-substituted aniline derivatives, we can characterize the SE using their homodesmotic reaction with benzene, where energy, SESE_AN–BEN_, can be calculated as:

9 SESE_AN–BEN_ = *E*_X–BEN_ + *E*_AN_ − (*E*_4-X–AN_ + *E*_BEN_)

The obtained values are presented in Table S9.† In the same table, values from B3LYP/6-311++G(d,p) calculations from our previous paper,^34^ are also given. These values are congruous with the results calculated at B97D3/aug-cc-pvdz level of theory.

The use of the SESE concept, with appropriately selected reactions, allows the comparison of the influence of a transmission moiety on the substituent effect. Purine is a reference for SESE_PU_, whereas benzene for SESE_AN–BEN_. The obtained results of linear relation SESE_PU_(adenine) *vs.* SESE_AN–BEN_(aniline), shown in Table 8, reveal: (i) a comparable transmission of the substituent effect only for C8–X 3H adenine (slope = 1.062 ± 0.104); (ii) a weaker transmission of the substituent effect by the purine moiety than by benzene in all other cases; and (iii) the substituent effect acting from the adenine C8 position is stronger than from the C2 position.

**Table:** Slope values, *a*, and determination coefficients, *R*^2^, of relation SESE_PU_(AD) *vs.* SESE_AN–BEN_(AN) for C2–X and C8–X substituted adenine tautomers

	SESE_PU_(AD) *vs.* SESE_AN–BEN_(AN)			
	C8–X		C2–X	
	*a*	*R* ^2^	*a*	*R* ^2^
9H	0.710 ± 0.037	0.978	0.257 ± 0.073	0.609
7H	0.417 ± 0.024	0.975	0.236 ± 0.058	0.674
3H	1.062 ± 0.104	0.929	0.183 ± 0.064	0.502
1H	0.804 ± 0.054	0.966	0.455 ± 0.054	0.898

In conclusion, a subsequent study confirmed that the more significant effect is observed in adenine when the substituent is attached to the 5-membered ring (substitution at C8–X position).

### SESE model based on reactions with imidazole or pyrimidine as references

**Scheme 3:**
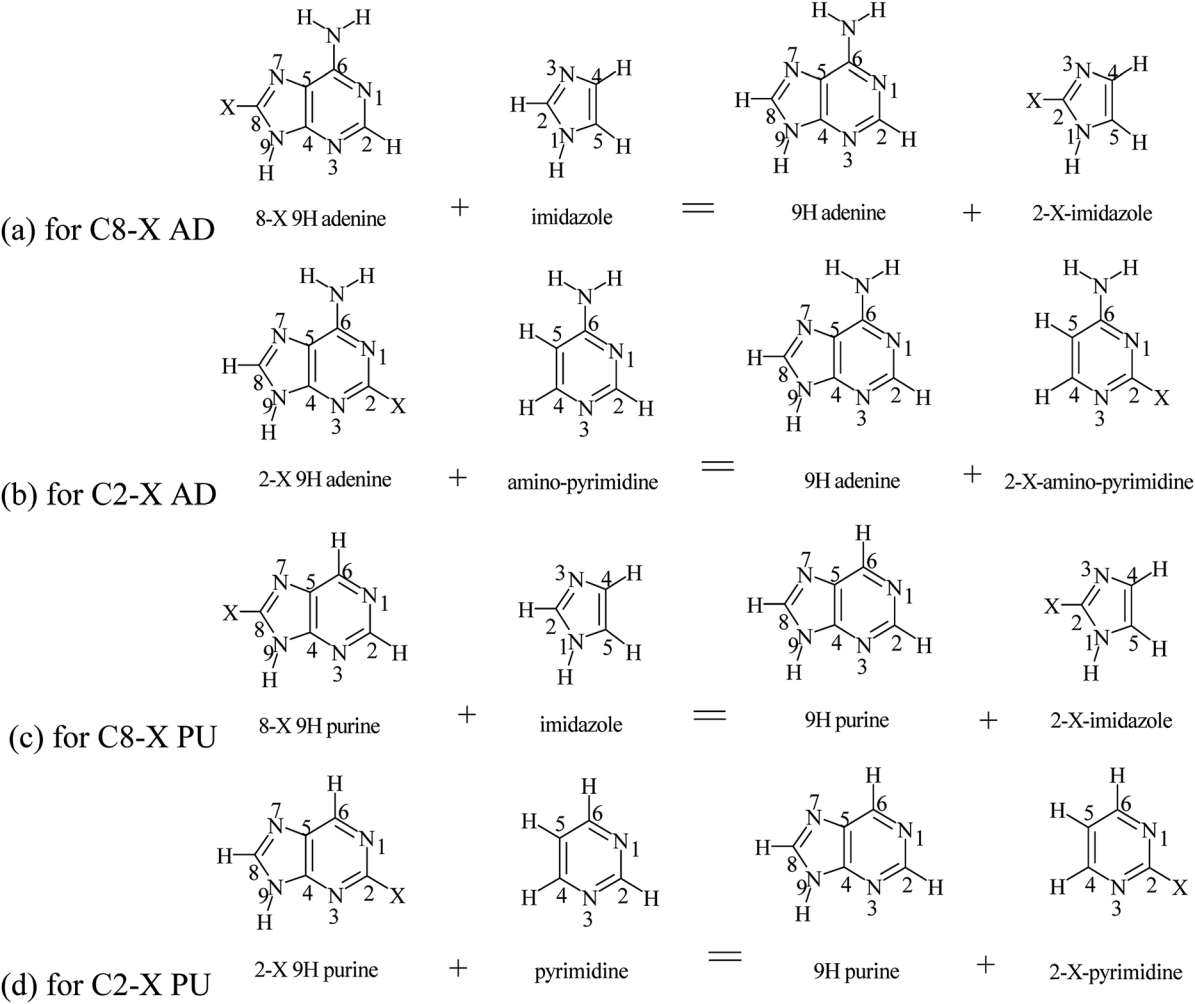
Homodesmotic reactions of adenine and purine with imidazole and aminopyrimidine (pyrimidine).

Purine (adenine) can be formed by the fusion of pyrimidine (NH_2_–pyrimidine) and imidazole rings. Therefore, it can be of interest how the substituent effects observed in adenine and purine derivatives differ from those in imidazole (IM) and amino-pyrimidine (PY), substituted in a similar topological environment as in the purine (adenine) molecules. For this purpose, appropriate homodesmotic reactions, for the 9H tautomer shown in Scheme 3 (analogous reactions can be written for 7H tautomer), are used. Their energy allow to calculate SESE_IM_ and SESE_PY_, both for adenine and purine C8–X and C2–X substituted series, respectively; for 9H adenine C8–X and C2–X derivatives, according to the following equations:

10 SESE_IM_ = *E*_9H AD_ + E_C2–X–IM_ − (*E*_9H C8–X–AD_ + *E*_IM_)

11 SESE_PY_ = *E*_9H AD_ + *E*_C2–X–PY_ − (*E*_9H C2–X–AD_ + *E*_PY_)

Analogous equations can be used for the remaining 9H and 7H tautomers of adenine and purine. The obtained values are presented in Table 9.

**Table:** SESE_IM_ and SESE_PY_ (in kcal mol^−1^) values for adenine and purine 9H and 7H tautomers with imidazole (IM) and aminopyrimidine (PY) as references

	Adenine				Purine			
	SESE_IM_		SESE_PY_		SESE_IM_		SESE_PY_	
	9H	7H	9H	7H	9H	7H	9H	7H
	C8–X	C8–X	C2–X	C2–X	C8–X	C8–X	C2–X	C2–X
NO_2_	−1.90	−3.21	0.54	0.63	−4.41	−4.79	0.71	0.67
CN	−1.67	−2.72	0.66	0.30	−3.49	−3.82	0.82	0.73
CHO	−1.77	−2.62	0.80	1.01	−3.63	−3.89	1.51	1.19
Cl	0.14	0.34	−0.73	−0.90	0.17	0.26	−0.72	−0.71
F	0.92	1.40	−1.16	−1.27	1.37	1.55	−1.18	−1.05
**H**	**0.00**	**0.00**	**0.00**	**0.00**	**0.00**	**0.00**	**0.00**	**0.00**
Me	1.13	1.39	−0.36	−0.33	1.74	1.78	−0.41	−0.53
OMe	2.44	4.03	−1.68	−1.05	4.17	4.93	−1.53	−1.26
OH	1.95	3.54	−1.88	−1.49	3.53	4.27	−1.20	−1.50
NH_2_	2.62	4.10	−1.57	−1.70	4.41	5.08	−1.82	−2.12
Range	4.52	7.32	2.68	2.72	8.82	9.87	3.34	3.31
Average	0.35	0.57	−0.49	−0.44	0.35	0.49	−0.35	−0.42
SD	1.64	2.66	0.98	0.90	3.12	3.52	1.06	1.04

For particular substituents, calculated SESE_PY_ and SESE_IM_ values have opposite signs. As in the case of SESE_PU_, SESE_PY_ values are negative (*i.e.* destabilizing interactions) for electron-donating substituents and usually positive for the electron-attracting ones. The opposite changes apply to SESE_IM_, that is for C8–X series both adenine and purine. Furthermore, the slopes of a linear relation of the SESE descriptors (SESE_IM_ and SESE_PY_) between the values for adenine and purine (both 9H and 7H) systems (Table 10) document that the amino group of adenine reduces the influence of the substituent effect. In addition, the substituent effect in the C8–X series is weaker than for the C2–X series and the discussed regressions are characterized by very high determination coefficients (*R*^2^ > 0.92).

**Table:** Slope values and determination coefficients of SESE_IM_ and SESE_PY_ relations between values obtained for 9H and 7H adenine (AD) and purine (PU) tautomers with substituents at positions C8–X and C2–X, respectively

	SESE_IM_(AD) *vs.* SESE_IM_(PU)		SESE_PY_(AD) *vs.* SESE_PY_(PU)	
	C8–X		C2–X	
	*a*	*R* ^2^	*a*	*R* ^2^
9H	0.523 ± 0.013	0.995	0.877 ± 0.087	0.925
7H	0.753 ± 0.012	0.998	0.851 ± 0.062	0.959

### Relations between SESE_BEN_, SESE_AN_, SESE_IM_ and SESE_PY_ descriptors and Hammett *σ*_p_ constants

Statistical details of the mutual dependences of SESE_BEN_, SESE_AN_, SESE_IM_ and SESE_PY_ on *σ*_p_ for C8–X and C2–X adenine and purine derivatives are presented in Table S10.†

It should be added that *σ*_p_ should be understood as the generally accepted characteristic of the substituent effect in cases where contributions from inductive/field and resonance effects are equal.^47–49^ The greater *R*^2^ of the regression SESE *vs. σ*_p_, the interactions between substituents in adenine/purine series are more similar to those in benzene. This could mean that in the studied systems the classical resonance and inductive/field effects are realized nearly alike. For almost all cases in the adenine series *R*^2^ values are worse than the ones in purines (Table S10†). This can be explained by the proximity effects of the NH_2_ group in adenine and thus more complex nature of interactions. Therefore, benzene (and pyrimidine) as a reference for the SE description in adenine systems is clearly worse than in purines. However, if aniline (*i.e.* 4-X–aniline) is a reference system, the determination coefficients improve, and SESE_AN_ becomes a good SE characteristic in both adenine and purine series. The same applies to SESE_IM_, but this descriptor can only be used for the C8–X series. In addition, better correlations of SESE_AN_ with *σ*_p_ for C8–X series than for C2–X indicate a greater role of the resonance effect in the first case.

Considering the slope absolute values and benzene (aniline) as a reference, for the purine derivatives the strength of the SE increases with the stability of tautomers for C2–X series, while opposite trends can be seen for C8–X systems. In the case of adenine derivatives, the picture is not so clear. In addition, again, lower absolute slope values in the second series may be related to the presence of the amino group in adenine.

### Classical substituent effect

**Fig. 5:**
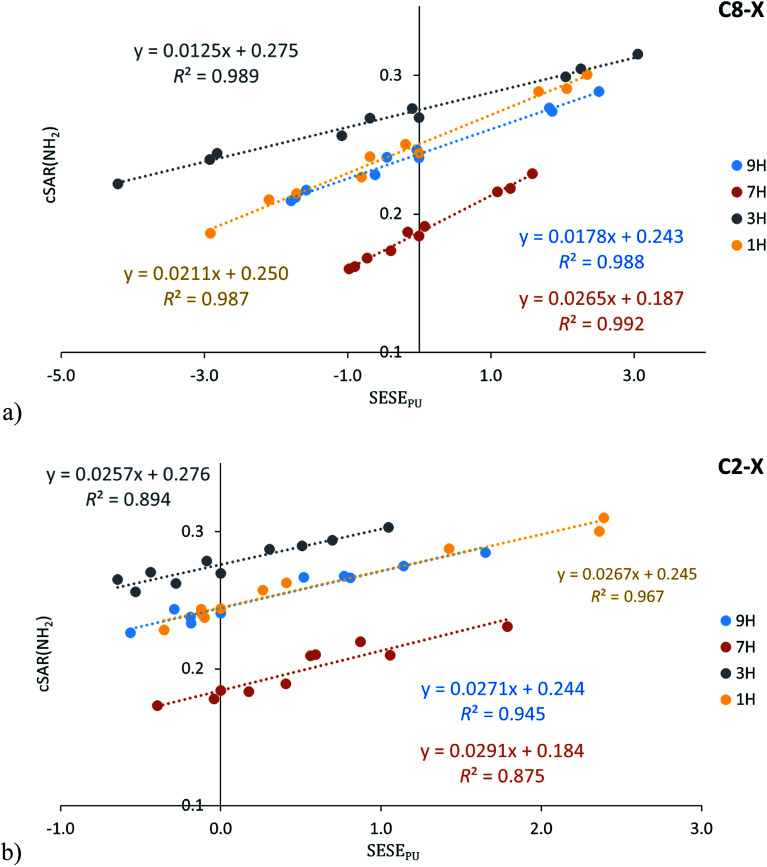
Dependence of cSAR(NH_2_) on SESE_PU_ (in kcal mol^−1^) for the C8–X (a) and C2–X (b) substituted adenine series.

The classical or traditional SE is the most common studied type of interaction – the properties of the “reaction site” Y (the fixed group in the series) are related to the properties of the X substituent in the disubstituted X–R–Y system. In the case of studied adenine derivatives, the C6-amino group is the fixed group (Y), purine moiety can be defined as R and substituents (X) can be introduced at the C2 or C8 positions (Scheme 1). The relationships between cSAR(NH_2_) and SESE_PUR_ (as the SE descriptor) for all studied adenine tautomers are shown in Fig. 5 (the obtained cSAR(NH_2_) values are gathered in Table S11†). In addition, statistics of these relations and others in which SESE_BEN_ and SESE_AN_ are used as the SE descriptor are presented in Table S12.†

**Scheme 4:**
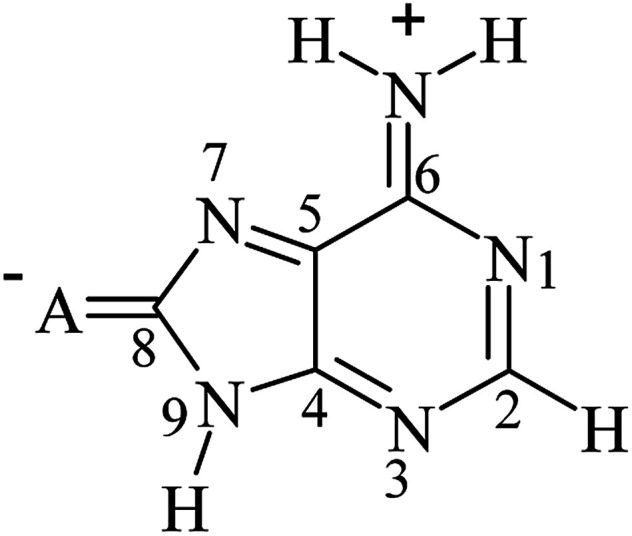
Intramolecular charge transfer from electron donating NH_2_ group to the electron attracting substituents.

Obtained dependences of cSAR(NH_2_) on SESE_PUR_ indicate an interesting difference between regressions for the C8–X and C2–X series. It is important to note the high values of the determination coefficient (for C8–X *R*^2^ ≥ 0.987, for C2–X *R*^2^ ≥ 0.875), which entitle us to a deeper analysis. The variability of cSAR(NH_2_) sensitivity to the SE in the C8–X series is significantly greater (slopes varies from 0.012 to 0.027) than in the C2–X series (the slope ranges from 0.025 to 0.029). This discrepancy can be easily explained by resonance structures for these two systems, which resemble the interactions with electron-attracting groups observed in the case of *para* and *meta* positions in the substituted aniline^34^ (or phenolate).^50^ Adenine C8–X derivatives resemble *para*-substitution in benzene, where the intramolecular charge transfer between the electron donating and electron accepting substituents can be described by canonical forms with charge separation, shown in Scheme 4. In the case of C2–X systems, this transfer requires the use of canonical forms with double charge separation (as for *meta*-nitrophenolate). In general, when the number of bonds between carbon atoms to which electron attracting and electron donating substituents are attached is odd, then a charge transfer occurs (as in *para* substituted benzene), if the number is even, then interactions are like those for *meta* substituted benzene.^51^

An another equivalent way of counting the bonds is the number of bonds between the electron-donating atom (group D, nitrogen this time) and the electron-accepting atom (group A, usually oxygen atom in the NO or NO_2_ groups or nitrogen atom in the CN group). In this case, if the number of all bonds between donating and accepting atoms in D and A groups, respectively, is even, then the charge transfer is privileged and well visible.^52^

The above observation is confirmed by determination coefficients of cSAR(NH_2_) relation to SESE_BEN_ and SESE_AN_ (obtained with benzene derivatives as a reference). In the case of the C8–X series, they are higher than 0.857 (excluding X = CHO, Table S12†), while in C2–X systems no correlation is observed, similarly as for substituted anilines in the *para* and *meta* positions, respectively.^34^

## Conclusions

Adenine, the amino derivative of purine, is a building block of DNA and RNA helices. Both, purine and adenine, can exist in various tautomeric forms. Therefore, impact of the substituent on their stability is a very important problem.

The strength of the substituent effect was analyzed depending both on the type of tautomers as well as on the substitution positions in the adenine and purine derivatives. The application of energetic descriptor, SESE, based on homodesmotic reactions with various reference systems, allowed to investigate different aspects of the substituent effect.

For all substituted adenine and purine series, the 9H tautomeric systems are the most stable (the only exception is the N–NO_2_ 9H purine), while the 1H tautomer systems are the least stable. In the case of 7H and 3H tautomers, the order of energy changes for substituted adenine derivatives differs from the sequences in purines, which can be explained by the presence (interaction) of the NH_2_ group. The amino group in adenine induces about 1.6 times greater energy difference between the most and least stable tautomers comparing to purine. In addition, substituents, except for the N–NO_2_ systems, do not change stability of purine tautomers, which decreases in the order 9H, 7H, 3H and 1H.

The application of different reference systems in homodesmotic reactions and Hammett substituent constants reveals various similarity aspects in interaction due to the substituent effect.

The results of SESE_rel_ (with respect to 9H tautomer) analysis indicate the stronger substituent effect in adenine than purine derivatives for substituents attached adjacent to the NH group of the ring. In addition, a more significant effect is observed in adenine systems when the substituent is attached to the 5-membered ring. This observation is also confirmed by the results of analysis of the differences between the energies of pairs of the same molecule with the substituent at the C8–X and C2–X positions, for all tautomers.

The SESE_PU_ (purine as reference) approach informs about changes in the substituent effect due to the presence of the amino group in adenine tautomers. Like in the substituted aniline derivatives, the obtained values show stabilizing and destabilizing interactions of the amino group with electron-attracting and electron-donating substituents, respectively. Moreover, these interactions are more pronounced in C8–X than in the C2–X series.

The description of the substituent effect in adenines and purines based on their similarity to monocyclic reference systems (benzene, aniline, imidazole or pyrimidine) allows to conclude that the amino group of adenine reduces the effect of substituent when compared to purine. This weakening is more pronounced for the C8–X substitution, independently of the tautomer. However, it should be emphasized that these SESE values, with the exception of SESE_PY_, suggest stabilizing interactions in the case of electron-donating substituents.

Dependences of SESE descriptors on the Hammett substituent constants allow to compare the similarity of the substituent effect in adenine (and purine) to that observed in benzene. The obtained relationships for SESE_PU_ reveal the similarity of the substituent effect in C8–X to the *para*, while in C2–X to the *meta* positions in benzene (documented by very good correlations with *σ*_p_ and *σ*_m_, respectively). As in the case of aniline derivatives,^34^ the sensitivity of the amino group to the substituent effect, along with much better correlations, exhibit C8–X systems when compared to C2–X ones. In addition, weaker transmission of the substituent effect by the purine moiety than by benzene is observed.

Furthermore, analysis of the classical substituent effect in adenine confirms the more pronounced substituent effect from the C8-position what can be explained by the stronger resonance effect in such arrangements.

Specific interactions between substituents and neighboring N or NH are the reason for deviations and lower similarity of the substituent effect when compared with systems without these kind of interactions. Particularly strong deviations are observed for CHO group.

## Conflicts of interest

The authors declare no competing financial interest.

## Supplementary Material

RA-009-C9RA04615A-s001
