# Supplementary material for: Substituent effects on the stability of the four most stable tautomers of adenine and purine
Source: RSC Adv. 2019 Oct 2;9(54):31343–56. doi: 10.1039/c9ra04615a (PMC9072591; doi:10.1039/c9ra04615a)
Supplement: RA-009-C9RA04615A-s001 [file RA-009-C9RA04615A-s001.pdf]

Electronic Supplementary Material (ESI) for RSC Advances.
This journal is © The Royal Society of Chemistry 2019

# Electronic Supplementary Information (ESI)

## Substituent effects on stability of the four most stable tautomers of adenine and purine

*Halina Szatylowicz,* [*a] *Anna Jezuita,*[b] *Paulina H. Marek,*[a,c] *and Tadeusz M. Krygowski*[c]

[a] Faculty of Chemistry, Warsaw University of Technology, Noakowskiego 3, 00-664 Warsaw (Poland) halina@ch.pw.edu.pl pmarek@ch.pw.edu.pl
[b] Faculty of Chemistry, Opole University, Oleska 48, 45-052 Opole (Poland) ajezuita@uni.opole.pl
[c] Faculty of Chemistry, University of Warsaw, Pasteura 1, 02-093 Warsaw (Poland) tmkryg@chem.uw.edu.pl

### Supplementary Information

**Figure S1** Dependences of (a) average $\Delta E_{8\text{-}2}$ values and (b) ranges of variability $\Delta E_{8\text{-}2}$ for adenine tautomers on that for purine tautomers SI 3

**Table S1** Relative energies ($E_{rel}$, relative to 9H tautomer) of the studied C8 and C2 substituted adenine derivatives for all four tautomers. SI 4

**Table S2** Relative energies ($E_{rel}$, relative to 9H tautomer) of the studied C8 and C2 substituted purine derivatives for all tautomers. SI 4

**Table S3** Relative energies ($E_{rel}$, relative to 9H tautomer) of the studied N-X substituted adenine and purine tautomer derivatives SI 5

**Table S4** The obtained slope, *a*, and determination coefficient, $R^2$, values of the linear regressions between $SESE_{rel}$ model for adenine and purine substituted tautomers, shown in Fig. 2. SI 5

**Table S5** The obtained slope, *a*, and determination coefficient, $R^2$, values of the linear regressions between $SESE_{rel}$ and σ for adenine and purine substituted tautomers. SI 6

**Table S6** Slope values, *a*, and determination coefficients, $R^2$, of relation $SESE_{PU}$ vs $\sigma_p$ and $\sigma_m$ for C2-X and C8-X substituted adenine tautomers. SI 6

**Table S7** Differences of energies between two conformations for X = CHO, OMe and OH substituted adenine derivatives. SI 6

**Table S8** $SESE_{AN}$ (in kcal/mol) estimated for adenine (AD) (a) and purine (PU) (b) with aniline as reference. SI 7

**Table S9** The $SESE_{AN-BEN}$ values obtained for *para* (4-X) and *meta* (3-X) substituted anilines. SI 8

**Table S10** Values of slopes, *a*, and determination coefficients, $R^2$, for studied dependences $SESE_{BEN}$, $SESE_{AN}$, $SESE_{IM}$ and $SESE_{PY}$ on $\sigma_p$ for adenine and purine derivatives for all tautomers. SI 9

**Table S11** The obtained cSAR($NH_2$) values for C2-X and C8-X substituted adenine tautomers. SI 10

**Table S12** Slope values, *a*, and determination coefficients, $R^2$, of relation cSAR($NH_2$) *vs* SESE for C2-X and C8-X substituted adenine tautomers. SI 10

**Table S13** Values of total energies and Gibbs free energies, cartesian coordinates of equilibrium geometries of C8-X and C2-X substituted adenine (AD) 9H, 7H, 3H and 1H tautomers. SI 11

**Table S14** Values of total energies and Gibbs free energies, cartesian coordinates of equilibrium geometries of NX substituted adenine (AD) 9H, 7H, 3H and 1H tautomers. SI 35

Table **S15** Values of total energies and Gibbs free energies, cartesian coordinates of equilibrium geometries of C8-X and C2-X substituted purine (PU) 9H, 7H, 3H and 1H tautomers. SI 45

Table **S16** Values of total energies and Gibbs free energies, cartesian coordinates of equilibrium geometries of NX substituted purine (PU) 9H, 7H, 3H and 1H tautomers. SI 63

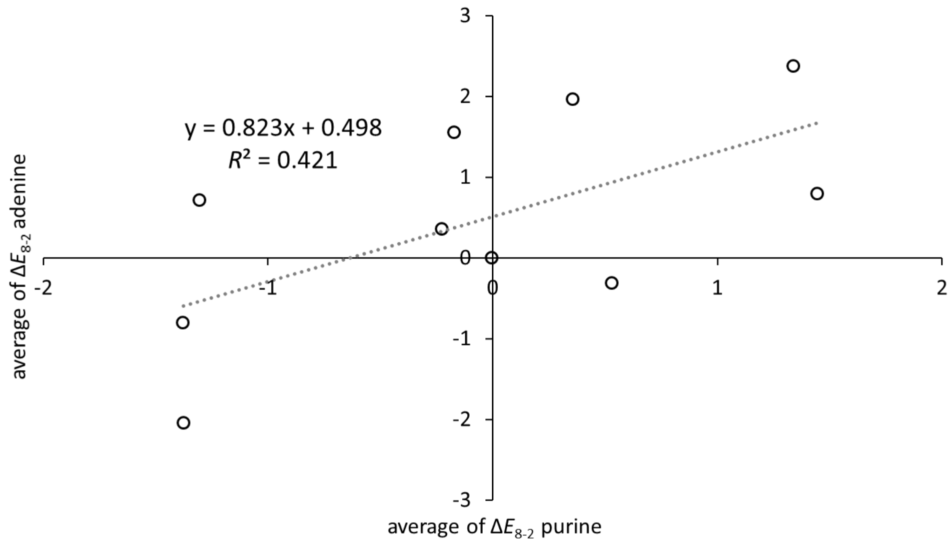

a)

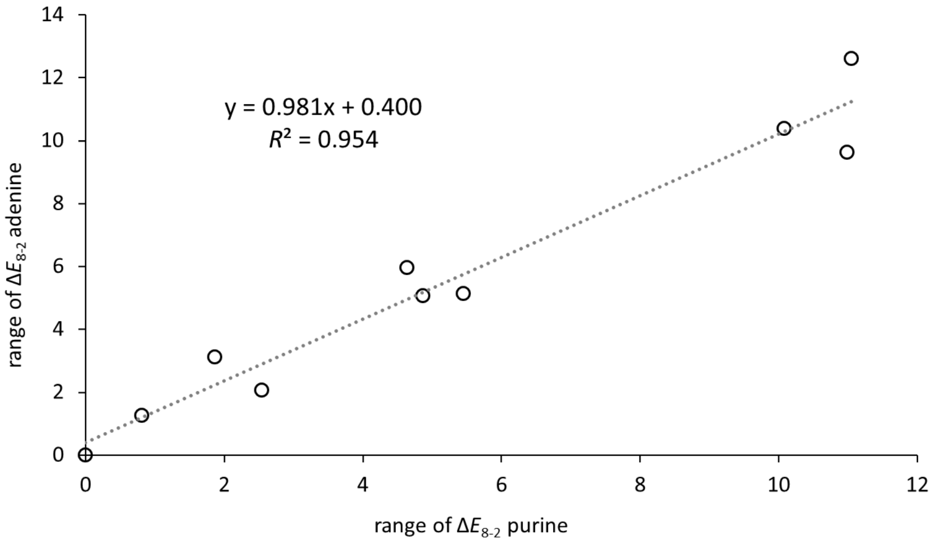

b)

**Figure S1**. Dependences of (a) average $\Delta E_{8\text{-}2}$ values and (b) ranges of variability $\Delta E_{8\text{-}2}$ for adenine tautomers on that for purine tautomers.

**Table S1**. Relative energies ($E_{rel}$, relative to 9H tautomer, in kcal mol$^{-1}$) of the studied C8 and C2 substituted adenine derivatives for all four tautomers.

| $E_{rel}$ kcal mol$^{-1}$ | C8-X | | | C2-X | | |
|---|---|---|---|---|---|---|
| | **7H** | **3H** | **1H** | **7H** | **3H** | **1H** |
| $NO_2$ | 8.50 | 6.81 | 17.16 | 7.09 | 7.17 | 14.39 |
| CN | 8.23 | 4.51 | 14.77 | 7.54 | 9.78 | 18.43 |
| CHO | 8.02 | 10.54 | 21.16 | 6.97 | 2.67 | 8.55 |
| Cl | 6.99 | 4.59 | 13.99 | 7.35 | 9.57 | 19.06 |
| F | 6.71 | 2.47 | 11.37 | 7.30 | 11.81 | 21.76 |
| **H** | **7.18** | **7.09** | **16.94** | **7.18** | **7.09** | **16.94** |
| Me | 6.92 | 7.54 | 17.04 | 7.16 | 6.52 | 16.30 |
| OMe | 5.59 | 5.76 | 14.53 | 6.55 | 7.55 | 16.60 |
| OH | 5.60 | 4.88 | 13.04 | 6.79 | 8.70 | 18.19 |
| $NH_2$ | 5.70 | 3.77 | 11.50 | 7.32 | 10.61 | 21.15 |
| *range* | 2.91 | 8.07 | 9.79 | 0.99 | 9.14 | 13.21 |
| *average* | 6.95 | 5.80 | 15.15 | 7.12 | 8.14 | 17.14 |
| *SD* | 1.08 | 2.30 | 3.00 | 0.29 | 2.57 | 3.75 |

**Table S2**. Relative energies ($E_{rel}$, relative to 9H tautomer, in kcal mol$^{-1}$) of the studied C8 and C2 substituted purine derivatives for all tautomers.

| $E_{rel}$ kcal mol$^{-1}$ | C8-X | | | C2-X | | |
|---|---|---|---|---|---|---|
| | **7H** | **3H** | **1H** | **7H** | **3H** | **1H** |
| $NO_2$ | 3.67 | 8.96 | 11.55 | 3.33 | 8.17 | 9.69 |
| CN | 3.62 | 6.56 | 9.18 | 3.38 | 10.94 | 13.28 |
| CHO | 3.54 | 12.34 | 15.93 | 3.62 | 3.67 | 4.95 |
| Cl | 3.20 | 6.14 | 8.39 | 3.28 | 10.71 | 13.26 |
| F | 3.11 | 3.85 | 5.70 | 3.15 | 13.12 | 15.77 |
| **H** | **3.29** | **8.69** | **11.50** | **3.29** | **8.69** | **11.50** |
| Me | 3.25 | 8.68 | 11.41 | 3.41 | 8.03 | 10.94 |
| OMe | 2.52 | 6.16 | 8.72 | 3.02 | 8.70 | 11.23 |
| OH | 2.55 | 5.43 | 7.47 | 3.59 | 10.16 | 12.92 |
| $NH_2$ | 2.62 | 2.95 | 4.93 | 3.59 | 12.25 | 15.92 |
| *range* | 1.15 | 9.39 | 10.99 | 0.59 | 9.44 | 10.96 |
| *average* | 3.14 | 6.98 | 9.48 | 3.37 | 9.44 | 11.95 |
| *SD* | 0.44 | 2.76 | 3.24 | 0.19 | 2.66 | 3.17 |

**Table S3**. Relative energies ($E_{rel}$, relative to 9H tautomer, in kcal mol$^{-1}$) of the studied N-X substituted adenine and purine tautomer derivatives

| $E_{rel}$ kcal mol$^{-1}$ | N-X adenine | | | N-X purine | | |
|---|---|---|---|---|---|---|
| | **7H** | **3H** | **1H** | **7H** | **3H** | **1H** |
| $NO_2$ | 1.21 | 9.21 | 16.81 | -1.70 | 11.23 | 9.30 |
| CN | 3.98 | 10.99 | 17.81 | 1.85 | 13.66 | 14.83 |
| CHO | 2.31 | 9.29 | 17.12 | 2.18 | 12.48 | 13.77 |
| Cl | 5.34 | 6.48 | 15.92 | 1.97 | 6.90 | 11.27 |
| F | 4.30 | 4.53 | 12.84 | 1.21 | 4.47 | 7.02 |
| **H** | **7.18** | **7.09** | **16.94** | **3.29** | **8.69** | **11.50** |
| Me | 7.85 | 6.65 | 18.05 | 3.39 | 7.80 | 13.01 |
| OMe | 4.71 | 5.14 | 13.23 | 2.40 | 5.66 | 9.04 |
| OH | 6.06 | 3.52 | 14.66 | 2.84 | 3.10 | 12.27 |
| $NH_2$ | 7.09 | 5.85 | 16.38 | 4.02 | 7.10 | 13.02 |
| *range* | 6.64 | 7.47 | 5.21 | 5.71 | 10.56 | 7.82 |
| *average* | 5.00 | 6.88 | 15.98 | 2.14 | 8.11 | 11.50 |
| *SD* | 2.15 | 2.34 | 1.82 | 1.59 | 3.45 | 2.41 |

**Table S4**. The obtained slope, *a*, and determination coefficient, $R^2$, values of the linear regressions between $SESE_{rel}$ model for adenine and purine substituted tautomers, shown in Fig. 2.

| | *a* | $R^2$ |
|---|---|---|
| C8-X | | |
| 7H | 2.447 ± 0.136 | 0.976 |
| 3H | 0.792 ± 0.090 | 0.906 |
| 1H | 0.922 ± 0.003 | 0.994 |
| C2-X | | |
| 7H | 0.211 ± 0.523 | 0.020 |
| 3H | 0.963 ± 0.029 | 0.993 |
| 1H | 1.176 ± 0.040 | 0.991 |
| N-X | | |
| 7H | 1.146 ± 0.256 | 0.715 |
| 7H without CHO | 1.151 ± 0.150 | 0.893 |
| 3H | 0.673 ± 0.031 | 0.983 |
| 1H | 0.580 ± 0.171 | 0.591 |
| 1H without $NO_2$, OH | 0.710 ± 0.118 | 0.858 |

**Table S5**. The obtained slope, *a*, and determination coefficient, $R^2$, values of the linear regressions between $SESE_{rel}$ and σ for adenine and purine substituted tautomers.

| | adenine | | purine | |
|---|---|---|---|---|
| | *a* | $R^2$ | *a* | $R^2$ |
| $SESE_{rel}$ vs $\sigma_p$ | C8-X | | | |
| 7H | **2.206** ± 0.291 | **0.878** | **0.855** ± 0.147 | **0.810** |
| 3H | 1.497 ± 1.682 | 0.090 | 3.306 ± 1.768 | 0.304 |
| 1H | 3.348 ± 1.976 | 0.264 | 3.815 ± 2.093 | 0.294 |
| | C2-X | | | |
| 7H | 0.205 ± 0.212 | 0.105 | -0.050 ± 0.149 | 0.014 |
| 3H | -1.484 ± 1.906 | 0.070 | -1.884 ± 1.934 | 0.106 |
| 1H | -3.633 ± 2.576 | 0.199 | -3.099 ± 2.176 | 0.202 |
| $SESE_{rel}$ vs $\sigma_m$ | C2-X | | | |
| 7H | 0.217 ± 0.360 | 0.044 | -0.128 ± 0.242 | 0.034 |
| 3H | -0.294 ± 3.250 | 0.001 | -1.024 ± 3.343 | 0.012 |
| 1H | -3.501 ± 4.569 | 0.068 | -2.913 ± 3.874 | 0.066 |

**Table S6**. Slope values, *a*, and determination coefficients, $R^2$, of relation $SESE_{PU}$ *vs* $\sigma_p$ and $\sigma_m$ for C2-X and C8-X substituted adenine tautomers.

| | **C8-X** | | **C2-X** | |
|---|---|---|---|---|
| $SESE_{PU}$ *vs* $\sigma_p$ | *a* | $R^2$ | *a* | $R^2$ |
| 9H | 3.27 ± 0.35 | 0.916 | 1.47 ± 0.19 / 1.55 ± 0.18 * | 0.878 / 0.912* |
| 7H | 1.92 ± 0.21 | 0.913 | 1.21 ± 0.23 | 0.784 |
| 3H | 5.08 ± 0.40 | 0.954 | 1.07 ± 0.21 / 1.18 ± 0.17 * | 0.757 / 0.869* |
| 1H | 3.74 ± 0.39 | 0.921 | 2.00 ± 0.39 / 1.77 ± 0.28 * | 0.769 / 0.848* |
| $SESE_{PU}$ *vs* $\sigma_m$ | *a* | $R^2$ | *a* | $R^2$ |
| 9H | 4.57 ± 1.16 | 0.659 | 2.47 ± 0.26 | 0.921 |
| 7H | 2.76 ± 0.65 | 0.693 | 2.13 ± 0.26 | 0.892 |
| 3H | 7.08 ± 1.75 | 0.672 | 1.74 ± 0.36 | 0.746 |
| 1H | 5.15 ± 1.35 | 0.645 | 3.06 ± 0.77 / 2.80 ± 0.49 * | 0.665 / 0.827* |

* without X = CHO

**Table S7.** Differences of energies (in kcal/mol) between two conformations for X = CHO, OMe and OH substituted adenine derivatives.

(a)

| AD | **9H** | | **7H** | | **3H** | | **1H** | |
|---|---|---|---|---|---|---|---|---|
| X | C8-X | C2-X | C8-X | C2-X | C8-X | C2-X | C8-X | C2-X |
| CHO | 5.5 | 0.2 | 6.6 | 1.4 | 0.4 | 5.9 | 0.5 | 9.8 |
| OMe | 5.1 | 0.2 | 6.0 | 1.6 | 0.7 | 5.5 | 0.2 | 8.5 |
| OH | 5.7 | 0.1 | 6.8 | 1.1 | 0.2 | 6.8 | 0.7 | 9.1 |

(b)

| AD | N9-X | N7-X | N3-X | N1-X |
|---|---|---|---|---|
| | **9H** | **7H** | **3H** | **1H** |
| CHO | 4.8 | 5.3 | 5.5 | 5.2 |
| OMe | - | - | - | - |
| OH | - | - | - | - |

**Table S8**. $SESE_{AN}$ (in kcal/mol) estimated for adenine (AD) (a) and purine (PU) (b) with aniline as reference.

(a)

| AD | $SESE_{AN}$ | | | | | | | |
|---|---|---|---|---|---|---|---|---|
| | 9H | | 7H | | 3H | | 1H | |
| | C8-X | C2-X | C8-X | C2-X | C8-X | C2-X | C8-X | C2-X |
| $NO_2$ | -8.00 | -8.16 | -9.31 | -8.06 | -7.72 | -8.24 | -8.22 | -5.61 |
| CN | -6.34 | -6.32 | -7.39 | -6.68 | -3.76 | -9.02 | -4.16 | -7.82 |
| CHO | -1.15 | -6.85 | -1.99 | -6.63 | -4.61 | -2.44 | -5.38 | 1.54 |
| Cl | -2.48 | 0.48 | -2.28 | 0.31 | 0.02 | -2.00 | 0.48 | -1.64 |
| F | -0.57 | 6.88 | -0.10 | 6.77 | 4.04 | 2.15 | 4.99 | 2.06 |
| **H** | **0.00** | **0.00** | **0.00** | **0.00** | **0.00** | **0.00** | **0.00** | **0.00** |
| Me | 3.90 | 2.71 | 4.16 | 2.73 | 3.44 | 3.28 | 3.80 | 3.35 |
| OMe | 6.51 | 9.27 | 8.11 | 9.90 | 7.84 | 8.81 | 8.92 | 9.61 |
| OH | 6.24 | 10.69 | 7.83 | 11.09 | 8.26 | 9.08 | 10.14 | 9.44 |
| $NH_2$ | 5.55 | 10.79 | 7.04 | 10.65 | 8.87 | 7.27 | 10.99 | 6.58 |
| *range* | 14.51 | 18.95 | 17.42 | 19.16 | 16.60 | 18.10 | 19.21 | 17.43 |
| *average* | 0.37 | 1.95 | 0.61 | 2.01 | 1.64 | 0.89 | 2.16 | 1.75 |
| *SD* | 5.14 | 7.36 | 6.15 | 7.46 | 5.82 | 6.49 | 6.75 | 5.84 |

(b)

| PU | $SESE_{AN}$ | | | | | | | |
|---|---|---|---|---|---|---|---|---|
| | 9H | | 7H | | 3H | | 1H | |
| | C8-X | C2-X | C8-X | C2-X | C8-X | C2-X | C8-X | C2-X |
| $NO_2$ | -10.51 | -9.81 | -10.89 | -9.85 | -10.78 | -9.29 | -10.56 | -8.00 |
| CN | -8.15 | -7.46 | -8.48 | -7.55 | -6.02 | -9.71 | -5.83 | -9.24 |
| CHO | -3.01 | -7.37 | -3.26 | -7.69 | -6.66 | -2.35 | -7.44 | -0.82 |
| Cl | -2.45 | -0.29 | -2.36 | -0.28 | 0.11 | -2.31 | 0.66 | -2.05 |
| F | -0.12 | 6.07 | 0.06 | 6.21 | 4.72 | 1.64 | 5.68 | 1.79 |
| **H** | **0.00** | **0.00** | **0.00** | **0.00** | **0.00** | **0.00** | **0.00** | **0.00** |
| Me | 4.51 | 2.90 | 4.55 | 2.77 | 4.52 | 3.56 | 4.60 | 3.45 |
| OMe | 8.24 | 9.46 | 9.01 | 9.72 | 10.77 | 9.45 | 11.02 | 9.73 |
| OH | 7.82 | 10.98 | 8.56 | 10.69 | 11.08 | 9.52 | 11.85 | 9.57 |
| $NH_2$ | 7.34 | 11.35 | 8.02 | 11.05 | 13.08 | 7.80 | 13.90 | 6.93 |
| *range* | 18.75 | 21.16 | 19.90 | 20.90 | 23.86 | 19.24 | 24.46 | 18.97 |
| *average* | 0.37 | 1.58 | 0.52 | 1.51 | 2.08 | 0.83 | 2.39 | 1.14 |
| *SD* | 6.62 | 7.93 | 7.02 | 7.94 | 8.20 | 7.00 | 8.52 | 6.59 |

**Table S9**. The $SESE_{AN\text{-}BEN}$ values (in kcal/mol) obtained for *para* (4-X) and *meta* (3-X) substituted anilines.

| method | B97D3 | | B3LYP | |
|---|---|---|---|---|
| bases | aug-cc-pvdz | | 6-311++Gdp | |
| | C4-X | C3-X | C4-X* | C3-X* |
| $NO_2$ | 3.29 | 0.79 | 3.17 | 0.75 |
| CN | 2.27 | 0.68 | 2.25 | 0.62 |
| CHO | 3.05 | 0.78 | 2.80 | 0.69 |
| Cl | -0.35 | 0.31 | -0.27 | 0.34 |
| F | -1.28 | 0.27 | -1.19 | 0.46 |
| H | 0.00 | 0.00 | 0.00 | 0.00 |
| Me | -0.65 | -0.05 | -0.65 | -0.03 |
| OMe | -2.10 | 0.04 | -2.05 | 0.24 |
| OH | -2.19 | 0.24 | -2.14 | 0.47 |
| $NH_2$ | -2.57 | -0.15 | -2.57 | 0.14 |
| range | 5.86 | 0.94 | 5.74 | 0.78 |
| average | -0.05 | 0.29 | -0.07 | 0.37 |
| SD | 2.19 | 0.35 | 2.11 | 0.28 |

* data taken from [H. Szatylowicz, T. Siodla, O. A. Stasyuk and T. M. Krygowski, *Phys. Chem. Chem. Phys.,* 2016, **18**, 11711- 11721]

**Table S10**. Values of slopes, *a*, and determination coefficients, $R^2$, for studied dependences $SESE_{BEN}$, $SESE_{AN}$, $SESE_{IM}$ and $SESE_{PY}$ on $\sigma_p$ for adenine and purine derivatives for all tautomers.

| | adenine | | purine | |
|---|---|---|---|---|
| | $SESE_{BEN}$ *vs* $\sigma_p$ | | | |
| | **C8-X** | | | |
| | *a* | $R^2$ | *a* | $R^2$ |
| 9H | -6.17 ± 1.53 | 0.670 | -9.44 ± 1.47 | 0.838 |
| 7H | -8.37 ± 1.54 | 0.788 | -10.30 ± 1.50 | 0.885 |
| 3H | -7.66 ± 0.96 | 0.888 | -12.75 ± 1.25 | 0.928 |
| 1H | -9.52 ± 1.17 | 0.892 | -13.26 ± 1.45 | 0.913 |
| | **C2-X** | | | |
| 9H | -10.42 ± 1.59 | 0.842 | -11.89 ± 1.46 | 0.892 |
| 7H | -10.63 ± 1.66 | 0.837 | -11.84 ± 1.53 | 0.882 |
| 3H | -8.67 ± 1.68 | 0.769 | -10.01 ± 1.74 | 0.806 |
| 1H | -6.79 ± 2.26 | 0.531 | -8.79 ± 2.02 | 0.702 |
| | $SESE_{AN}$ *vs* $\sigma_p$ | | | |
| | **C8-X** | | | |
| | *a* | $R^2$ | *a* | $R^2$ |
| 9H | -10.58 ± 1.28 | 0.896 | -13.85 ± 1.36 | 0.928 |
| 7H | -12.79 ± 1.39 | 0.914 | -14.71 ± 1.43 | 0.929 |
| 3H | -12.03 ± 1.38 | 0.905 | -17.16 ±1.72 | 0.926 |
| 1H | -13.93 ± 1.63 | 0.901 | -17.67 ± 1.95 | 0.911 |
| | **C2-X** | | | |
| 9H | -14.83 ± 2.11 | 0.861 | -16.30 ± 1.97 | 0.895 |
| 7H | -15.04 ± 2.15 | 0.859 | -16.25 ± 2.06 | 0.886 |
| 3H | -13.35 ± 1.62 | 0.895 | -14.42 ± 1.71 | 0.899 |
| 1H | -11.20 ± 2.12 | 0.778 | -13.20 ± 1.96 | 0.850 |
| | $SESE_{IM}$ *vs* $\sigma_p$ | | | |
| | **C8-X** | | | |
| | *a* | $R^2$ | *a* | $R^2$ |
| 9H | -3.57 ± 0.41 | 0.906 | -6.85 ± 0.73 | 0.916 |
| 7H | -5.78 ± 0.66 | 0.905 | -7.70 ± 0.84 | 0.913 |
| | $SESE_{PY}$ *vs* $\sigma_p$ | | | |
| | **C2-X** | | | |
| | *a* | $R^2$ | *a* | $R^2$ |
| 9H | 1.87 ± 0.42 | 0.717 | 2.04 ± 0.46 | 0.708 |
| 7H | 1.67 ± 0.42 | 0.664 | 2.09 ± 0.38 | 0.789 |

**Table S11**. The obtained cSAR($NH_2$) values for C2-X and C8-X substituted adenine tautomers.

| AD-X | cSAR($NH_2$) | | | | | | | |
|---|---|---|---|---|---|---|---|---|
| | **9H** | | **7H** | | **3H** | | **1H** | |
| | C8-X | C2-X | C8-X | C2-X | C8-X | C2-X | C8-X | C2-X |
| $NO_2$ | 0.2885 | 0.2847 | 0.2291 | 0.2305 | 0.3156 | 0.3033 | 0.3009 | 0.3103 |
| CN | 0.2767 | 0.2752 | 0.2159 | 0.2196 | 0.3048 | 0.2937 | 0.2887 | 0.2877 |
| CHO | 0.2741 | 0.2666 | 0.2185 | 0.2098 | 0.2989 | 0.2785 | 0.2906 | 0.3002 |
| Cl | 0.2464 | 0.2676 | 0.1909 | 0.2103 | 0.2763 | 0.2873 | 0.2505 | 0.2626 |
| F | 0.2411 | 0.2665 | 0.1869 | 0.2095 | 0.2691 | 0.2898 | 0.2415 | 0.2575 |
| **H** | 0.2405 | 0.2405 | 0.1841 | 0.1841 | 0.2696 | 0.2696 | 0.2439 | 0.2439 |
| Me | 0.2283 | 0.2333 | 0.1734 | 0.1780 | 0.2567 | 0.2622 | 0.2268 | 0.2373 |
| OMe | 0.2120 | 0.2379 | 0.1620 | 0.1834 | 0.2393 | 0.2653 | 0.2101 | 0.2401 |
| OH | 0.2171 | 0.2435 | 0.1679 | 0.1888 | 0.2438 | 0.2706 | 0.2149 | 0.2436 |
| $NH_2$ | 0.2095 | 0.2263 | 0.1602 | 0.1730 | 0.2219 | 0.2595 | 0.1862 | 0.2284 |
| *range* | 0.0791 | 0.0584 | 0.0689 | 0.0575 | 0.0937 | 0.0438 | 0.1147 | 0.0819 |
| *average* | 0.2434 | 0.2542 | 0.1889 | 0.1987 | 0.2696 | 0.2780 | 0.2454 | 0.2612 |
| *SD* | 0.0282 | 0.0201 | 0.0247 | 0.0196 | 0.0303 | 0.0149 | 0.0381 | 0.0286 |

**Table S12**. Slope values, *a*, and determination coefficients, $R^2$, of relation cSAR($NH_2$) *vs* SESE for C2-X and C8-X substituted adenine tautomers.

| | C8-X | | C2-X | |
|---|---|---|---|---|
| | *a* | $R^2$ | *a* | $R^2$ |
| | cSAR($NH_2$) *vs* $SESE_{PU}$ | | | |
| 9H | 0.0178 ± 0.0007 | 0.988 | 0.0271 ± 0.0023 | 0.945 |
| 7H | 0.0265 ± 0.0008 | 0.992 | 0.0291 ± 0.0039 | 0.875 |
| 3H | 0.0125 ± 0.0005 | 0.989 | 0.0250 ± 0.0029 | 0.904 |
| 1H | 0.0211 ± 0.0009 | 0.987 | 0.0267 ± 0.0018 | 0.968 |
| | cSAR($NH_2$) *vs* $SESE_{BEN}$ | | | |
| 9H | -0.0064 ± 0.0018 | 0.613 | -0.0028 ± 0.0009 | 0.534 |
| | -0.0071 ± 0.0011 | 0.857* | | |
| 7H | -0.0048 ± 0.0011 | 0.713 | -0.0027 ± 0.0009 | 0.525 |
| | -0.0049 ± 0.0006 | 0.905* | | |
| 3H | -0.0078 ± 0.0008 | 0.916 | -0.0027 ± 0.0007 | 0.643 |
| 1H | -0.0078 ± 0.0009 | 0.908 | -0.0034 ± 0.0020 | 0.267 |
| | cSAR($NH_2$) *vs* $SESE_{AN}$ | | | |
| 9H | -0.0052 ± 0.0007 | 0.886 | -0.0021 ± 0.0006 | 0.564 |
| | -0.0050 ± 0.0004 | 0.962* | | |
| 7H | -0.0038 ± 0.0005 | 0.886 | -0.0020 ± 0.0006 | 0.559 |
| | -0.0036 ± 0.0003 | 0.960* | | |
| 3H | -0.0051 ± 0.0004 | 0.943 | -0.0019 ± 0.0005 | 0.663 |
| 1H | -0.0055 ± 0.0005 | 0.938 | -0.0036 ± 0.0012 | 0.533 |

* without X = CHO

**Table S13.** Values of total energies and Gibbs free energies, cartesian coordinates of equilibrium geometries of C8-X and C2-X substituted adenine (AD) 9H, 7H, 3H and 1H tautomers.

C8-$NO_2$-AD_9H $E_{tot}$ = -671.528174 a.u.
Gibbs free energy $E_G$= -671.453526 a.u.

| | | | | |
|---|---|---|---|---|
| 7 | N | 3.051385 | -0.01772 | 0.000257 |
| 6 | C | 2.746118 | -1.33337 | 0.00002 |
| 7 | N | 1.53543 | -1.92744 | -0.00014 |
| 6 | C | 0.550393 | -1.01753 | -0.00024 |
| 6 | C | 0.701554 | 0.39419 | -0.0001 |
| 6 | C | 2.039737 | 0.87859 | 0.000164 |
| 7 | N | -0.52075 | 1.035663 | -0.00042 |
| 6 | C | -1.38885 | 0.039864 | -8.4E-05 |
| 7 | N | -0.81619 | -1.21783 | -2E-06 |
| 7 | N | 2.329792 | 2.200113 | -0.00034 |
| 1 | H | 3.297922 | 2.492331 | 0.00136 |
| 1 | H | 1.588182 | 2.886888 | 0.001235 |
| 1 | H | 3.606125 | -2.01057 | 0.000368 |
| 1 | H | -1.32741 | -2.09385 | 0.000222 |
| 7 | N | -2.83393 | 0.172764 | 0.000083 |
| 8 | O | -3.46926 | -0.89808 | 0.000245 |
| 8 | O | -3.31558 | 1.302564 | 0.000032 |

C2-$NO_2$-AD_9H $E_{tot}$ = -671.527920 a.u.
Gibbs free energy $E_G$= -671.452683 a.u.

| | | | | |
|---|---|---|---|---|
| 7 | N | 3.046942 | -0.01773 | 0.000271 |
| 6 | C | 2.721601 | -1.30837 | 0.000153 |
| 7 | N | 1.536128 | -1.91409 | -5.5E-06 |
| 6 | C | 0.550419 | -1.00068 | -8.5E-05 |
| 6 | C | 0.699478 | 0.403469 | -2.7E-05 |
| 6 | C | 2.036062 | 0.883051 | 0.000136 |
| 7 | N | -0.53464 | 1.038806 | -0.00028 |
| 6 | C | -1.41325 | 0.046862 | 6.23E-05 |
| 7 | N | -0.8178 | -1.20576 | -7.5E-05 |
| 7 | N | 2.341987 | 2.202056 | -0.00032 |
| 1 | H | 3.312858 | 2.484092 | 0.001186 |
| 1 | H | 1.606485 | 2.894618 | 0.001048 |
| 1 | H | -1.27995 | -2.10767 | -6.1E-05 |
| 7 | N | 3.919078 | -2.26255 | 0.000517 |
| 8 | O | 3.670941 | -3.46573 | -3.5E-05 |
| 8 | O | 5.037433 | -1.75457 | 0.001265 |
| 1 | H | -2.49484 | 0.164399 | 0.000112 |

C8-CN-AD_9H $E_{tot}$ = -559.285658 a.u.
Gibbs free energy $E_G$= -559.213562 a.u.

| | | | | |
|---|---|---|---|---|
| 7 | N | -2.69641 | -0.02423 | -0.00023 |
| 6 | C | -2.38169 | -1.33751 | -1.6E-05 |
| 7 | N | -1.16594 | -1.91953 | 0.000096 |
| 6 | C | -0.18879 | -0.99872 | 0.000181 |
| 6 | C | -0.34955 | 0.406884 | 0.00007 |
| 6 | C | -1.69086 | 0.879203 | -0.00015 |
| 7 | N | 0.868412 | 1.05841 | 0.000246 |
| 6 | C | 1.766702 | 0.072743 | 0.000038 |
| 7 | N | 1.178073 | -1.19282 | -6.1E-05 |
| 7 | N | -1.99264 | 2.199695 | 0.000651 |
| 1 | H | -2.96295 | 2.483723 | -0.00187 |
| 1 | H | -1.2568 | 2.892178 | -0.00188 |
| 1 | H | -3.23594 | -2.022 | -0.00031 |
| 1 | H | 1.659664 | -2.08493 | -0.00016 |
| 6 | C | 3.179585 | 0.236019 | -7.3E-05 |
| 7 | N | 4.349025 | 0.332665 | -0.00015 |

C2-CN-AD_9H $E_{tot}$ = -559.285679 a.u.
Gibbs free energy $E_G$= -559.214063 a.u.

| | | | | |
|---|---|---|---|---|
| 7 | N | 2.707322 | -0.0276 | 0.000394 |
| 6 | C | 2.387419 | -1.34143 | 0.000631 |
| 7 | N | 1.169701 | -1.92426 | 0.0003 |
| 6 | C | 0.197908 | -0.99881 | 0.000106 |
| 6 | C | 0.360527 | 0.403264 | 1.79E-05 |
| 6 | C | 1.699396 | 0.873706 | 5.63E-05 |
| 7 | N | -0.86708 | 1.052321 | 5.02E-05 |
| 6 | C | -1.75591 | 0.069837 | -0.00012 |
| 7 | N | -1.17327 | -1.18903 | -6.1E-05 |
| 7 | N | 2.007235 | 2.193509 | -0.00063 |
| 1 | H | 2.977914 | 2.475431 | 0.001759 |
| 1 | H | 1.272208 | 2.886522 | 0.001618 |
| 1 | H | -1.64609 | -2.08525 | -0.00011 |
| 1 | H | -2.83649 | 0.198267 | -0.00021 |
| 6 | C | 3.518081 | -2.25069 | 0.000342 |
| 7 | N | 4.431238 | -2.98403 | 0.000146 |

C8-CHO-AD_9H $E_{tot}$ = -580.377851 a.u.
Gibbs free energy $E_G$= -580.295182 a.u.

| | | | | |
|---|---|---|---|---|
| 7 | N | -2.75516 | -0.21694 | -0.00021 |
| 6 | C | -2.31075 | -1.49311 | -2.5E-05 |
| 7 | N | -1.0445 | -1.954 | 0.000129 |
| 6 | C | -0.15797 | -0.94504 | 0.000199 |
| 6 | C | -0.45971 | 0.443685 | 0.000056 |
| 6 | C | -1.84254 | 0.779489 | -0.00015 |
| 7 | N | 0.686237 | 1.206 | 0.000255 |
| 6 | C | 1.681948 | 0.31035 | 0.000055 |
| 7 | N | 1.216827 | -0.99926 | -5.2E-05 |
| 7 | N | -2.27373 | 2.063953 | 0.000543 |
| 1 | H | -3.26751 | 2.250206 | -0.00175 |
| 1 | H | -1.6099 | 2.825688 | -0.00176 |
| 1 | H | -3.09419 | -2.25783 | -0.00019 |
| 1 | H | 1.805223 | -1.82567 | -0.00019 |
| 6 | C | 3.108805 | 0.633609 | 0.000004 |
| 8 | O | 3.988866 | -0.22394 | -0.00021 |
| 1 | H | 3.329063 | 1.726862 | 0.000115 |

C2-CHO-AD_9H $E_{tot}$ = -580.368390 a.u.
Gibbs free energy $E_G$= -580.286234 a.u.

| | | | | |
|---|---|---|---|---|
| 7 | N | 2.74373 | -0.24696 | -7.1E-06 |
| 6 | C | 2.293642 | -1.52667 | -0.00019 |
| 7 | N | 1.017433 | -1.96458 | -0.00016 |
| 6 | C | 0.154087 | -0.94277 | -0.00026 |
| 6 | C | 0.463469 | 0.436726 | -0.00022 |
| 6 | C | 1.844206 | 0.760338 | -7.1E-05 |
| 7 | N | -0.68766 | 1.212792 | -0.00072 |
| 6 | C | -1.67625 | 0.329145 | 0.000106 |
| 7 | N | -1.23217 | -0.98339 | 0.000247 |
| 7 | N | 2.290952 | 2.04245 | -0.00097 |
| 1 | H | 3.285719 | 2.219932 | 0.001386 |
| 1 | H | 1.633981 | 2.809665 | 0.001312 |
| 1 | H | -1.79747 | -1.8243 | 0.000641 |
| 6 | C | 3.396173 | -2.55271 | 0.000419 |
| 8 | O | 3.228385 | -3.75862 | 0.000795 |
| 1 | H | 4.41503 | -2.08959 | 0.001152 |
| 1 | H | -2.73684 | 0.573254 | 0.000341 |

C8-CHO-AD_9H $E_{tot}$ = -580.369005 a.u.
Gibbs free energy $E_G$= -580.286774 a.u.

| | | | | |
|---|---|---|---|---|
| 7 | N | 2.735495 | -0.13251 | 0.000183 |
| 6 | C | 2.275491 | -1.40377 | 0.000328 |
| 7 | N | 1.004037 | -1.84911 | 8.79E-05 |
| 6 | C | 0.131231 | -0.82676 | 8.1E-05 |
| 6 | C | 0.447833 | 0.556022 | 8.32E-05 |
| 6 | C | 1.835909 | 0.875336 | 2.46E-05 |
| 7 | N | -0.68368 | 1.335977 | 0.000383 |
| 6 | C | -1.69652 | 0.46487 | 4.76E-05 |
| 7 | N | -1.24359 | -0.86179 | -9.9E-05 |
| 7 | N | 2.277168 | 2.155233 | -0.00032 |
| 1 | H | 3.272029 | 2.335197 | 0.001323 |
| 1 | H | 1.617037 | 2.920739 | 0.001356 |
| 1 | H | 3.04972 | -2.17784 | -9.9E-06 |
| 1 | H | -1.81356 | -1.70036 | -0.00027 |
| 6 | C | -3.13722 | 0.762826 | -1.6E-05 |
| 8 | O | -3.62355 | 1.882712 | 0.000109 |
| 1 | H | -3.7857 | -0.15537 | -0.00036 |

C2-CHO-AD_9H $E_{tot}$ = --580.368769 a.u.
Gibbs free energy $E_G$= -580.287453 a.u.

| | | | | |
|---|---|---|---|---|
| 7 | N | 0.980462 | 1.447537 | 0.000038 |
| 6 | C | 1.522341 | 0.211110 | 0.000264 |
| 7 | N | 0.886732 | -0.985811 | 0.000332 |
| 6 | C | -0.444536 | -0.834088 | 0.000223 |
| 6 | C | -1.155632 | 0.385198 | -0.000023 |
| 6 | C | -0.362706 | 1.564859 | -0.000204 |
| 7 | N | -2.526697 | 0.169261 | 0.000375 |
| 6 | C | -2.650039 | -1.151032 | -0.000294 |
| 7 | N | -1.430739 | -1.809300 | 0.000387 |
| 7 | N | -0.911612 | 2.805253 | -0.000896 |
| 1 | H | -0.302224 | 3.611883 | 0.001312 |
| 1 | H | -1.915229 | 2.919038 | 0.001486 |
| 1 | H | -1.273384 | -2.810126 | 0.000608 |
| 6 | C | 3.022830 | 0.093499 | 0.000435 |
| 1 | H | -3.592911 | -1.694519 | -0.000423 |
| 1 | H | 3.366962 | -0.971638 | 0.000463 |
| 8 | O | 3.800990 | 1.029952 | 0.000561 |

C8-Cl-AD_9H $E_{tot}$ = -926.728065 a.u.
Gibbs free energy $E_G$= -926.663 a.u.

| | | | | |
|---|---|---|---|---|
| 7 | N | -2.80562 | -0.03014 | 0.006289 |
| 6 | C | -2.49557 | -1.34259 | 0.004669 |
| 7 | N | -1.2781 | -1.92359 | -0.00085 |
| 6 | C | -0.30372 | -1.00156 | -0.00329 |
| 6 | C | -0.46172 | 0.399317 | -0.00323 |
| 6 | C | -1.79585 | 0.872003 | -0.00096 |
| 7 | N | 0.773768 | 1.052237 | -0.00043 |
| 6 | C | 1.643569 | 0.067604 | -0.0005 |
| 7 | N | 1.072919 | -1.19592 | -0.00264 |
| 7 | N | -2.1027 | 2.197444 | -0.03228 |
| 1 | H | -3.06853 | 2.469556 | 0.093513 |
| 1 | H | -1.37022 | 2.882132 | 0.095229 |
| 1 | H | -3.34983 | -2.02713 | 0.009894 |
| 1 | H | 1.563556 | -2.08186 | -0.00282 |
| 17 | Cl | 3.357786 | 0.240379 | 0.002737 |

C8-F-AD_9H $E_{tot}$ = -566.298514 a.u.
Gibbs free energy $E_G$= -566.230975 a.u.

| | | | | |
|---|---|---|---|---|
| 7 | N | -2.40905 | -0.1512 | 0.005658 |
| 6 | C | -2.01939 | -1.44093 | 0.004872 |
| 7 | N | -0.76661 | -1.9437 | -0.00078 |
| 6 | C | 0.14607 | -0.96298 | -0.00303 |
| 6 | C | -0.09785 | 0.425072 | -0.00321 |
| 6 | C | -1.45683 | 0.812719 | -0.00153 |
| 7 | N | 1.099946 | 1.156507 | -9.5E-05 |
| 6 | C | 2.009867 | 0.222565 | 0.00071 |
| 7 | N | 1.537605 | -1.07353 | -0.0018 |
| 7 | N | -1.8484 | 2.116848 | -0.03467 |
| 1 | H | -2.82904 | 2.324738 | 0.098849 |
| 1 | H | -1.1623 | 2.846449 | 0.101304 |
| 1 | H | -2.82869 | -2.17797 | 0.008451 |
| 1 | H | 2.084715 | -1.92593 | -0.00173 |
| 9 | F | 3.327782 | 0.42881 | 0.003966 |

C8-H-AD_9H $E_{tot}$ = -467.096786 a.u.
Gibbs free energy $E_G$= -467.019892 a.u.

| | | | | |
|---|---|---|---|---|
| 7 | N | 1.962029 | 0.527275 | 0.004592 |
| 6 | C | 1.304445 | 1.706449 | 0.002924 |
| 7 | N | -0.02351 | 1.934087 | -0.00124 |
| 6 | C | -0.71338 | 0.780032 | -0.00262 |
| 6 | C | -0.17923 | -0.52523 | -0.00271 |
| 6 | C | 1.234633 | -0.6137 | -0.00179 |
| 7 | N | -1.18892 | -1.48459 | 0.002006 |
| 6 | C | -2.30842 | -0.7791 | 0.002786 |
| 7 | N | -2.08635 | 0.591594 | -0.00028 |
| 7 | N | 1.889474 | -1.80705 | -0.03371 |
| 1 | H | 2.892382 | -1.80781 | 0.096014 |
| 1 | H | 1.367686 | -2.66248 | 0.09916 |
| 1 | H | 1.940976 | 2.597346 | 0.008033 |
| 1 | H | -3.31484 | -1.19342 | 0.006012 |
| 1 | H | -2.78351 | 1.326384 | -0.00041 |

C2-Cl-AD_9H $E_{tot}$ = -926.732773 a.u.
Gibbs free energy $E_G$= -926.667577 a.u.

| | | | | |
|---|---|---|---|---|
| 7 | N | -2.80734 | -0.0337 | 0.019792 |
| 6 | C | -2.47941 | -1.33143 | 0.005714 |
| 7 | N | -1.27922 | -1.91941 | -0.00546 |
| 6 | C | -0.3015 | -0.99388 | -0.00096 |
| 6 | C | -0.46067 | 0.406281 | 0.013339 |
| 6 | C | -1.79933 | 0.871222 | 0.023965 |
| 7 | N | 0.771099 | 1.053895 | 0.013972 |
| 6 | C | 1.656351 | 0.071058 | 0.000401 |
| 7 | N | 1.067699 | -1.1887 | -0.0091 |
| 7 | N | -2.11524 | 2.189889 | 0.038241 |
| 1 | H | -3.08736 | 2.466006 | 0.045147 |
| 1 | H | -1.38355 | 2.886223 | 0.041454 |
| 1 | H | 1.537289 | -2.08622 | -0.01997 |
| 17 | Cl | -3.85189 | -2.43694 | 0.001266 |
| 1 | H | 2.737532 | 0.194593 | -0.00335 |

C2-F-AD_9H $E_{tot}$ = -566.310389 a.u.
Gibbs free energy $E_G$= -566.242457 a.u.

| | | | | |
|---|---|---|---|---|
| 7 | N | -2.41619 | -0.15724 | 0.017325 |
| 6 | C | -1.99565 | -1.42294 | 0.004033 |
| 7 | N | -0.77128 | -1.94381 | -0.00625 |
| 6 | C | 0.148608 | -0.95875 | -0.00144 |
| 6 | C | -0.10093 | 0.428 | 0.011992 |
| 6 | C | -1.4664 | 0.808703 | 0.021178 |
| 7 | N | 1.087373 | 1.153675 | 0.014247 |
| 6 | C | 2.033778 | 0.230743 | 0.002212 |
| 7 | N | 1.526292 | -1.06537 | -0.00773 |
| 7 | N | -1.8615 | 2.105746 | 0.029822 |
| 1 | H | -2.84784 | 2.32412 | 0.054519 |
| 1 | H | -1.17204 | 2.843565 | 0.05246 |
| 1 | H | 2.052527 | -1.93085 | -0.01763 |
| 1 | H | 3.104888 | 0.422643 | -4.1E-05 |
| 9 | F | -2.99448 | -2.33469 | 0.000832 |

C2-H-AD_9H $E_{tot}$ = -467.096786 a.u.
Gibbs free energy $E_G$=-467.019892 a.u.

| | | | | |
|---|---|---|---|---|
| 7 | N | 1.962029 | 0.527275 | 0.004592 |
| 6 | C | 1.304445 | 1.706449 | 0.002924 |
| 7 | N | -0.02351 | 1.934087 | -0.00124 |
| 6 | C | -0.71338 | 0.780032 | -0.00262 |
| 6 | C | -0.17923 | -0.52523 | -0.00271 |
| 6 | C | 1.234633 | -0.6137 | -0.00179 |
| 7 | N | -1.18892 | -1.48459 | 0.002006 |
| 6 | C | -2.30842 | -0.7791 | 0.002786 |
| 7 | N | -2.08635 | 0.591594 | -0.00028 |
| 7 | N | 1.889474 | -1.80705 | -0.03371 |
| 1 | H | 2.892382 | -1.80781 | 0.096014 |
| 1 | H | 1.367686 | -2.66248 | 0.09916 |
| 1 | H | 1.940976 | 2.597346 | 0.008033 |
| 1 | H | -3.31484 | -1.19342 | 0.006012 |
| 1 | H | -2.78351 | 1.326384 | -0.00041 |

C8-Me-AD_9H $E_{tot}$ = -506.398603 a.u.
Gibbs free energy $E_G$=-506.29734 a.u.

| | | | | |
|---|---|---|---|---|
| 7 | N | -2.45329 | -0.10302 | 0.006983 |
| 6 | C | -2.09174 | -1.40278 | 0.005275 |
| 7 | N | -0.8526 | -1.9345 | -0.00118 |
| 6 | C | 0.087738 | -0.97488 | -0.00427 |
| 6 | C | -0.12524 | 0.41862 | -0.00477 |
| 6 | C | -1.47692 | 0.835079 | -0.00242 |
| 7 | N | 1.083376 | 1.111687 | -0.0004 |
| 6 | C | 2.017527 | 0.169197 | 0.000985 |
| 7 | N | 1.466576 | -1.11222 | -0.00276 |
| 7 | N | -1.83636 | 2.151097 | -0.04285 |
| 1 | H | -2.8072 | 2.379831 | 0.126621 |
| 1 | H | -1.12948 | 2.854179 | 0.126144 |
| 1 | H | -2.9187 | -2.12039 | 0.012297 |
| 1 | H | 1.970078 | -1.991 | -0.00227 |
| 6 | C | 3.491565 | 0.401851 | 0.004501 |
| 1 | H | 3.966429 | -0.04837 | 0.893153 |
| 1 | H | 3.968099 | -0.03556 | -0.88967 |
| 1 | H | 3.677777 | 1.483323 | 0.012035 |

C2-Me-AD_9H $E_{tot}$ = -506.396710 a.u.
Gibbs free energy $E_G$=-506.29638 a.u.

| | | | | |
|---|---|---|---|---|
| 7 | N | -2.44549 | -0.10853 | 0.013165 |
| 6 | C | -2.09655 | -1.4204 | 0.007372 |
| 7 | N | -0.84502 | -1.93126 | -0.00276 |
| 6 | C | 0.091644 | -0.96828 | -0.0053 |
| 6 | C | -0.12178 | 0.423799 | -0.00246 |
| 6 | C | -1.4782 | 0.831503 | 0.003786 |
| 7 | N | 1.083738 | 1.122091 | 0.000967 |
| 6 | C | 2.00809 | 0.175341 | -0.002 |
| 7 | N | 1.470793 | -1.10612 | -0.00647 |
| 7 | N | -1.84127 | 2.145418 | -0.03002 |
| 1 | H | -2.81398 | 2.373773 | 0.12772 |
| 1 | H | -1.13562 | 2.852904 | 0.124273 |
| 1 | H | 1.976208 | -1.98378 | -0.00885 |
| 1 | H | 3.083417 | 0.343029 | -0.00082 |
| 6 | C | -3.23822 | -2.40422 | 0.014047 |
| 1 | H | -3.8731 | -2.24183 | 0.900442 |
| 1 | H | -3.87628 | -2.24936 | -0.87152 |
| 1 | H | -2.85449 | -3.43236 | 0.017231 |

C8-OMe-AD_9H $E_{tot}$ = -581.577754 a.u.
Gibbs free energy $E_G$= -581.472704 a.u.

| | | | | |
|---|---|---|---|---|
| 7 | N | -2.78495 | 0.270621 | 0.00619 |
| 6 | C | -2.68422 | -1.07221 | 0.005121 |
| 7 | N | -1.56777 | -1.83232 | -0.0004 |
| 6 | C | -0.46345 | -1.07378 | -0.00334 |
| 6 | C | -0.40224 | 0.334835 | -0.00473 |
| 6 | C | -1.64433 | 1.002837 | -0.00334 |
| 7 | N | 0.924131 | 0.787615 | -0.00019 |
| 6 | C | 1.632019 | -0.32703 | 0.001051 |
| 7 | N | 0.867814 | -1.4836 | -0.00093 |
| 7 | N | -1.74707 | 2.368079 | -0.05368 |
| 1 | H | -2.65402 | 2.765224 | 0.158397 |
| 1 | H | -0.92554 | 2.914755 | 0.17007 |
| 1 | H | -3.63313 | -1.61807 | 0.012029 |
| 1 | H | 1.212162 | -2.43554 | -0.00062 |
| 8 | O | 2.965689 | -0.4561 | 0.003888 |
| 6 | C | 3.694486 | 0.798326 | 0.000613 |
| 1 | H | 4.752494 | 0.510708 | -0.00232 |
| 1 | H | 3.438958 | 1.379873 | -0.89786 |
| 1 | H | 3.444885 | 1.381156 | 0.900001 |

C2-OMe-AD_9H $E_{tot}$ = -581.582143 a.u.
Gibbs free energy $E_G$= -581.477071 a.u.

| | | | | |
|---|---|---|---|---|
| 7 | N | 1.172431 | 0.743748 | -0.01034 |
| 6 | C | 1.271205 | -0.60296 | -0.00689 |
| 7 | N | 0.290019 | -1.52498 | -0.0008 |
| 6 | C | -0.92248 | -0.94173 | 0.000879 |
| 6 | C | -1.19661 | 0.438243 | 0.000564 |
| 6 | C | -0.05916 | 1.287303 | -0.00255 |
| 7 | N | -2.56853 | 0.681959 | -0.00362 |
| 6 | C | -3.11518 | -0.52146 | -0.00403 |
| 7 | N | -2.16853 | -1.54371 | -0.00078 |
| 7 | N | -0.17266 | 2.644035 | 0.029323 |
| 1 | H | 0.661257 | 3.199244 | -0.10836 |
| 1 | H | -1.08139 | 3.064806 | -0.10774 |
| 1 | H | -2.34393 | -2.54101 | -0.00067 |
| 8 | O | 2.557264 | -1.0306 | -0.01291 |
| 1 | H | -4.18246 | -0.73333 | -0.00679 |
| 6 | C | 2.766683 | -2.4562 | -0.01125 |
| 1 | H | 3.85732 | -2.57989 | -0.01602 |
| 1 | H | 2.326762 | -2.91418 | 0.888457 |
| 1 | H | 2.318457 | -2.9176 | -0.90512 |

C8-OMe-AD_9H $E_{tot}$ = -581.569543 a.u.
Gibbs free energy $E_G$= -581.464982 a.u.

| | | | | |
|---|---|---|---|---|
| 7 | N | -2.78414 | 0.28138 | 0.008074 |
| 6 | C | -2.70655 | -1.06384 | 0.004425 |
| 7 | N | -1.60305 | -1.84225 | -0.00209 |
| 6 | C | -0.48762 | -1.09937 | -0.00241 |
| 6 | C | -0.39955 | 0.305334 | -0.00058 |
| 6 | C | -1.63256 | 0.995011 | 0.000801 |
| 7 | N | 0.929066 | 0.736475 | 0.007161 |
| 6 | C | 1.625919 | -0.38243 | 0.007135 |
| 7 | N | 0.837944 | -1.53332 | 0.000768 |
| 7 | N | -1.7078 | 2.359449 | -0.04299 |
| 1 | H | -2.60714 | 2.781977 | 0.148896 |
| 1 | H | -0.8711 | 2.893517 | 0.152357 |
| 1 | H | -3.66475 | -1.59303 | 0.009449 |
| 1 | H | 1.134391 | -2.50031 | 0.00049 |
| 8 | O | 2.97111 | -0.39746 | 0.013974 |
| 6 | C | 3.61758 | -1.68257 | 0.007826 |
| 1 | H | 3.35351 | -2.26114 | 0.910553 |
| 1 | H | 3.359054 | -2.24942 | -0.90384 |
| 1 | H | 4.692797 | -1.46844 | 0.012585 |

C2-OMe-AD_9H $E_{tot}$ = -581.581759 a.u.
Gibbs free energy $E_G$= -581.476584 a.u.

| | | | | |
|---|---|---|---|---|
| 7 | N | 1.174032 | 0.793835 | -0.00302 |
| 6 | C | 1.298732 | -0.55015 | 0.000068 |
| 7 | N | 0.333552 | -1.48937 | 0.004074 |
| 6 | C | -0.88268 | -0.92642 | 0.004 |
| 6 | C | -1.18606 | 0.452017 | 0.004112 |
| 6 | C | -0.07064 | 1.320238 | 0.003429 |
| 7 | N | -2.56347 | 0.668177 | -0.00364 |
| 6 | C | -3.08393 | -0.54622 | -0.00531 |
| 7 | N | -2.11859 | -1.5506 | -0.00039 |
| 7 | N | -0.20248 | 2.67699 | 0.041224 |
| 1 | H | 0.619943 | 3.2412 | -0.12656 |
| 1 | H | -1.11619 | 3.079552 | -0.11916 |
| 1 | H | -2.27349 | -2.55137 | -0.00152 |
| 8 | O | 2.550524 | -1.07086 | -0.0036 |
| 6 | C | 3.650528 | -0.13995 | -0.0022 |
| 1 | H | 4.549462 | -0.77014 | 0.0003 |
| 1 | H | 3.625102 | 0.496978 | -0.90012 |
| 1 | H | 3.620931 | 0.499075 | 0.894014 |
| 1 | H | -4.14704 | -0.77885 | -0.01055 |

C8-OH-AD_9H $E_{tot}$ = -542.301221 a.u.
Gibbs free energy $E_G$= -542.220982 a.u.

| | | | | |
|---|---|---|---|---|
| 7 | N | -2.41898 | -0.12014 | 0.006536 |
| 6 | C | -2.05175 | -1.41558 | 0.005832 |
| 7 | N | -0.80551 | -1.93629 | -9.1E-05 |
| 6 | C | 0.123263 | -0.97187 | -0.0032 |
| 6 | C | -0.09789 | 0.420565 | -0.0049 |
| 6 | C | -1.4492 | 0.826579 | -0.00324 |
| 7 | N | 1.11389 | 1.125945 | 7.95E-05 |
| 6 | C | 2.024621 | 0.173914 | 0.001081 |
| 7 | N | 1.511861 | -1.10831 | -0.00152 |
| 7 | N | -1.82405 | 2.142048 | -0.05131 |
| 1 | H | -2.79394 | 2.35059 | 0.150426 |
| 1 | H | -1.12976 | 2.846718 | 0.160293 |
| 1 | H | -2.87192 | -2.1405 | 0.01172 |
| 1 | H | 2.039039 | -1.97271 | -0.00136 |
| 8 | O | 3.362373 | 0.337007 | 0.004601 |
| 1 | H | 3.521142 | 1.296595 | 0.001374 |

C2-OH-AD_9H $E_{tot}$ = -542.308123 a.u.
Gibbs free energy $E_G$= -542.228153 a.u.

| | | | | |
|---|---|---|---|---|
| 7 | N | -1.59565 | 0.732259 | 0.009828 |
| 6 | C | -1.66958 | -0.60926 | 0.003569 |
| 7 | N | -0.67781 | -1.52279 | -0.00307 |
| 6 | C | 0.527595 | -0.92613 | -0.00176 |
| 6 | C | 0.779441 | 0.459732 | 0.002971 |
| 6 | C | -0.36741 | 1.293121 | 0.006729 |
| 7 | N | 2.147765 | 0.724222 | 0.00758 |
| 6 | C | 2.712818 | -0.46985 | 0.004374 |
| 7 | N | 1.782255 | -1.50776 | -0.002 |
| 7 | N | -0.27569 | 2.648957 | -0.01265 |
| 1 | H | -1.1199 | 3.195832 | 0.088026 |
| 1 | H | 0.628313 | 3.088882 | 0.088176 |
| 1 | H | 1.973953 | -2.50205 | -0.00533 |
| 1 | H | 3.783234 | -0.66528 | 0.006286 |
| 8 | O | -2.93783 | -1.09806 | 0.006018 |
| 1 | H | -2.84534 | -2.06625 | 0.002192 |

C8-OH-AD_9H $E_{tot}$ = -542.292184 a.u.
Gibbs free energy $E_G$=-542.213349 a.u.

| | | | | |
|---|---|---|---|---|
| 7 | N | -2.42323 | -0.11787 | 0.010082 |
| 6 | C | -2.06755 | -1.41671 | 0.005142 |
| 7 | N | -0.82579 | -1.94883 | -0.00384 |
| 6 | C | 0.109685 | -0.99026 | -0.00603 |
| 6 | C | -0.09648 | 0.402078 | -0.0035 |
| 6 | C | -1.44533 | 0.820569 | 0.00122 |
| 7 | N | 1.115572 | 1.100766 | 0.001627 |
| 6 | C | 2.025218 | 0.152989 | -0.00011 |
| 7 | N | 1.498089 | -1.13662 | -0.00596 |
| 7 | N | -1.80416 | 2.138255 | -0.03962 |
| 1 | H | -2.77254 | 2.364357 | 0.14763 |
| 1 | H | -1.09749 | 2.837809 | 0.145993 |
| 1 | H | -2.89424 | -2.13417 | 0.012357 |
| 1 | H | 1.992386 | -2.02091 | -0.0042 |
| 8 | O | 3.353674 | 0.406679 | 0.005044 |
| 1 | H | 3.85417 | -0.4228 | -0.0066 |

C2-OH-AD_9H $E_{tot}$ = -542.308312 a.u.
Gibbs free energy $E_G$= -542.227964 a.u.

| | | | | |
|---|---|---|---|---|
| 7 | N | -2.43487 | -0.14946 | 0.014406 |
| 6 | C | -2.04568 | -1.44547 | 0.004444 |
| 7 | N | -0.80976 | -1.96237 | -0.00556 |
| 6 | C | 0.113393 | -0.98543 | -0.00426 |
| 6 | C | -0.11698 | 0.405257 | 0.002709 |
| 6 | C | -1.47624 | 0.802052 | 0.009853 |
| 7 | N | 1.080742 | 1.117392 | 0.008368 |
| 6 | C | 2.0156 | 0.183208 | 0.002891 |
| 7 | N | 1.491193 | -1.10712 | -0.00516 |
| 7 | N | -1.85105 | 2.110552 | -0.01643 |
| 1 | H | -2.82473 | 2.338512 | 0.132714 |
| 1 | H | -1.14752 | 2.822712 | 0.125014 |
| 1 | H | 2.006242 | -1.97917 | -0.00954 |
| 1 | H | 3.089026 | 0.361871 | 0.004779 |
| 8 | O | -3.05186 | -2.35894 | 0.007065 |
| 1 | H | -3.87436 | -1.83998 | 0.011617 |

C8-$NH_2$-AD_9H $E_{tot}$ = -522.439419 a.u
Gibbs free energy $E_G$= -522.347455 a.u

| | | | | |
|---|---|---|---|---|
| 7 | N | -2.43342 | -0.11678 | 0.005091 |
| 6 | C | -2.06818 | -1.41293 | 0.005296 |
| 7 | N | -0.82218 | -1.93493 | -0.00203 |
| 6 | C | 0.108298 | -0.9712 | -0.00281 |
| 6 | C | -0.10979 | 0.420932 | -0.00311 |
| 6 | C | -1.46118 | 0.827325 | -0.00474 |
| 7 | N | 1.098245 | 1.124344 | -0.00715 |
| 6 | C | 2.025272 | 0.177659 | 0.000592 |
| 7 | N | 1.494623 | -1.1088 | -0.00459 |
| 7 | N | -1.83257 | 2.145592 | -0.05872 |
| 1 | H | -2.79883 | 2.354933 | 0.160255 |
| 1 | H | -1.13448 | 2.842291 | 0.168448 |
| 1 | H | -2.8893 | -2.13683 | 0.015086 |
| 1 | H | 2.001183 | -1.98127 | 0.080494 |
| 7 | N | 3.391618 | 0.392401 | 0.0896 |
| 1 | H | 3.619174 | 1.372494 | -0.05702 |
| 1 | H | 3.961502 | -0.21507 | -0.49406 |

C2-$NH_2$-AD_9H $E_{tot}$ = -522.447764 a.u.
Gibbs free energy $E_G$= -522.355774 a.u

| | | | | |
|---|---|---|---|---|
| 7 | N | 2.454677 | -0.12805 | -0.04902 |
| 6 | C | 2.08476 | -1.43623 | -0.06598 |
| 7 | N | 0.833293 | -1.95446 | -0.04613 |
| 6 | C | -0.09416 | -0.98555 | -0.02743 |
| 6 | C | 0.128795 | 0.406344 | -0.01512 |
| 6 | C | 1.487364 | 0.807918 | -0.01969 |
| 7 | N | -1.07291 | 1.112904 | 0.000541 |
| 6 | C | -2.00412 | 0.175804 | 5.99E-05 |
| 7 | N | -1.4728 | -1.11331 | -0.01461 |
| 7 | N | 1.850985 | 2.122375 | 0.043743 |
| 1 | H | 2.817561 | 2.354386 | -0.14422 |
| 1 | H | 1.140814 | 2.824884 | -0.11486 |
| 1 | H | -1.98457 | -1.98688 | -0.02382 |
| 1 | H | 3.121273 | -2.34304 | -0.15612 |
| 7 | N | -3.07829 | 0.34895 | 0.009487 |
| 1 | H | 4.034146 | -1.9982 | 0.112351 |
| 1 | H | 2.897234 | -3.29659 | 0.099345 |

C8-$NO_2$-AD_7H  $E_{tot}$ = -671.514633 a.u.
Gibbs free energy  $E_G$= -671.440228 a.u.

| | | | | |
|---|---|---|---|---|
| 7 | N | -1.9551 | -0.49365 | 0.001328 |
| 6 | C | -1.32718 | -1.69881 | -0.00813 |
| 7 | N | -0.01691 | -1.9628 | 0.000457 |
| 6 | C | 0.743247 | -0.8473 | 0.003918 |
| 6 | C | 0.1989 | 0.469894 | 0.000115 |
| 6 | C | -1.20809 | 0.621382 | 0.009085 |
| 7 | N | 1.291625 | 1.318933 | -0.00749 |
| 6 | C | 2.393614 | 0.495358 | -0.00813 |
| 7 | N | 2.130259 | -0.79467 | 0.002423 |
| 7 | N | -1.84683 | 1.838198 | -0.0314 |
| 1 | H | -2.84661 | 1.801365 | 0.139275 |
| 1 | H | -1.37044 | 2.655681 | 0.328739 |
| 1 | H | -1.99665 | -2.56469 | -0.0176 |
| 1 | H | 1.347315 | 2.33058 | -0.04855 |
| 7 | N | 3.722177 | 1.103841 | -0.02084 |
| 8 | O | 3.73699 | 2.348694 | -0.03759 |
| 8 | O | 4.698603 | 0.365949 | -0.01452 |

C2-$NO_2$-AD_7H  $E_{tot}$ = -671.516624 a.u.
Gibbs free energy  $E_G$= -671.4435 a.u.

| | | | | |
|---|---|---|---|---|
| 7 | N | -1.96367 | -0.50606 | 0.037673 |
| 6 | C | -1.30252 | -1.66757 | -0.01593 |
| 7 | N | -0.01037 | -1.94734 | -0.04173 |
| 6 | C | 0.742849 | -0.82309 | -0.04754 |
| 6 | C | 0.181461 | 0.481418 | 0.004456 |
| 6 | C | -1.22084 | 0.618285 | 0.06843 |
| 7 | N | 1.270973 | 1.340571 | -0.02854 |
| 6 | C | 2.398151 | 0.539999 | -0.1021 |
| 7 | N | 2.125827 | -0.75373 | -0.11019 |
| 7 | N | -1.88304 | 1.824198 | 0.099021 |
| 1 | H | -2.87722 | 1.754646 | 0.292676 |
| 1 | H | -1.41755 | 2.618709 | 0.52037 |
| 1 | H | 1.259939 | 2.352361 | -0.05921 |
| 1 | H | 3.396301 | 0.972684 | -0.14591 |
| 7 | N | -2.21211 | -2.87548 | -0.04973 |
| 8 | O | -2.03281 | -3.72798 | 0.815309 |
| 8 | O | -3.05577 | -2.90124 | -0.94361 |

C8-CN-AD_7H  $E_{tot}$ = -559.272535 a.u.
Gibbs free energy  $E_G$= -559.200373 a.u.

| | | | | |
|---|---|---|---|---|
| 7 | N | -1.94893 | -0.49362 | -0.00432 |
| 6 | C | -1.31743 | -1.69605 | -0.00963 |
| 7 | N | -0.00562 | -1.95327 | 0.006606 |
| 6 | C | 0.750955 | -0.8347 | 0.009112 |
| 6 | C | 0.202522 | 0.47697 | -0.00122 |
| 6 | C | -1.20334 | 0.62262 | 0.004976 |
| 7 | N | 1.290004 | 1.332338 | -0.00979 |
| 6 | C | 2.419842 | 0.520159 | -0.007 |
| 7 | N | 2.136124 | -0.77856 | 0.010038 |
| 7 | N | -1.84727 | 1.840887 | -0.04088 |
| 1 | H | -2.8465 | 1.789276 | 0.132767 |
| 1 | H | -1.38499 | 2.644211 | 0.368294 |
| 1 | H | -1.98278 | -2.56512 | -0.02029 |
| 1 | H | 1.297828 | 2.343152 | -0.07565 |
| 6 | C | 3.724996 | 1.092976 | -0.01994 |
| 7 | N | 4.775038 | 1.614725 | -0.03159 |

C2-CN-AD_7H  $E_{tot}$ = -559.273660 a.u.
Gibbs free energy  $E_G$= -559.201542 a.u.

| | | | | |
|---|---|---|---|---|
| 7 | N | -1.97013 | -0.50756 | -0.00283 |
| 6 | C | -1.32569 | -1.70242 | -0.00818 |
| 7 | N | -0.00852 | -1.95518 | 0.006965 |
| 6 | C | 0.736926 | -0.83186 | 0.010033 |
| 6 | C | 0.177302 | 0.474123 | 0.000512 |
| 6 | C | -1.2263 | 0.611577 | 0.00518 |
| 7 | N | 1.266289 | 1.332777 | -0.00865 |
| 6 | C | 2.396773 | 0.530303 | -0.00808 |
| 7 | N | 2.124799 | -0.76182 | 0.00883 |
| 7 | N | -1.88386 | 1.822536 | -0.04365 |
| 1 | H | -2.88254 | 1.760483 | 0.130196 |
| 1 | H | -1.43025 | 2.628715 | 0.36928 |
| 1 | H | 1.256791 | 2.343251 | -0.06992 |
| 6 | C | -2.19917 | -2.86195 | -0.02234 |
| 7 | N | -2.91088 | -3.79175 | -0.03367 |
| 1 | H | 3.39563 | 0.96358 | -0.0194 |

C8-CHO-AD_7H $E_{tot}$ = -580.365063 a.u.
Gibbs free energy $E_G$= -580.282361 a.u.

| | | | | |
|---|---|---|---|---|
| 7 | N | -1.95033 | -0.49711 | -0.00057 |
| 6 | C | -1.31556 | -1.69992 | -0.00777 |
| 7 | N | -0.00501 | -1.95661 | 0.003081 |
| 6 | C | 0.753473 | -0.8365 | 0.006139 |
| 6 | C | 0.201704 | 0.478428 | -0.00025 |
| 6 | C | -1.20654 | 0.619514 | 0.006309 |
| 7 | N | 1.284544 | 1.332253 | -0.00827 |
| 6 | C | 2.415237 | 0.533076 | -0.00744 |
| 7 | N | 2.134453 | -0.77159 | 0.004994 |
| 7 | N | -1.85043 | 1.836299 | -0.03904 |
| 1 | H | -2.849 | 1.792514 | 0.138247 |
| 1 | H | -1.37862 | 2.648827 | 0.338813 |
| 1 | H | -1.98145 | -2.56885 | -0.01665 |
| 1 | H | 1.316818 | 2.345019 | -0.0502 |
| 6 | C | 3.76111 | 1.123106 | -0.02222 |
| 8 | O | 3.947976 | 2.336188 | -0.03675 |
| 1 | H | 4.591993 | 0.381285 | -0.01995 |

C2-CHO-AD_7H $E_{tot}$ = -580.357663 a.u.
Gibbs free energy $E_G$= -580.275525 a.u.

| | | | | |
|---|---|---|---|---|
| 7 | N | -2.09234 | -0.44428 | -0.00546 |
| 6 | C | -1.47836 | -1.65277 | -0.01005 |
| 7 | N | -0.15878 | -1.91802 | 0.006378 |
| 6 | C | 0.608989 | -0.81194 | 0.009404 |
| 6 | C | 0.069998 | 0.502307 | -0.00108 |
| 6 | C | -1.33354 | 0.659957 | 0.003275 |
| 7 | N | 1.171178 | 1.343949 | -0.01192 |
| 6 | C | 2.291027 | 0.525116 | -0.01047 |
| 7 | N | 1.999933 | -0.76231 | 0.008012 |
| 7 | N | -1.9683 | 1.885566 | -0.04714 |
| 1 | H | -2.9681 | 1.8331 | 0.126406 |
| 1 | H | -1.50671 | 2.676572 | 0.386845 |
| 1 | H | 1.175551 | 2.354382 | -0.07424 |
| 6 | C | -2.349 | -2.88156 | -0.02395 |
| 8 | O | -3.56669 | -2.87693 | -0.0386 |
| 1 | H | -1.75079 | -3.82662 | -0.0195 |
| 1 | H | 3.295874 | 0.944426 | -0.02241 |

C8-CHO-AD_7H $E_{tot}$ = -580.354559 a.u.
Gibbs free energy $E_G$= -580.272533 a.u.

| | | | | |
|---|---|---|---|---|
| 7 | N | -1.94976 | -0.47843 | -0.00692 |
| 6 | C | -1.33544 | -1.69234 | -0.00912 |
| 7 | N | -0.02981 | -1.97146 | 0.01027 |
| 6 | C | 0.749427 | -0.86579 | 0.013243 |
| 6 | C | 0.21869 | 0.457503 | 0.000448 |
| 6 | C | -1.18619 | 0.623955 | 0.002457 |
| 7 | N | 1.316995 | 1.289869 | -0.00934 |
| 6 | C | 2.443904 | 0.46405 | -0.00373 |
| 7 | N | 2.129363 | -0.82898 | 0.015307 |
| 7 | N | -1.81001 | 1.853474 | -0.04775 |
| 1 | H | -2.81047 | 1.817852 | 0.123673 |
| 1 | H | -1.33638 | 2.647913 | 0.365878 |
| 1 | H | -2.01606 | -2.54969 | -0.02048 |
| 1 | H | 1.330035 | 2.301435 | -0.07051 |
| 6 | C | 3.787685 | 1.077306 | -0.01834 |
| 1 | H | 3.763855 | 2.202349 | -0.03936 |
| 8 | O | 4.840326 | 0.465549 | -0.00951 |

C2-CHO-AD_7H $E_{tot}$ = -580.355458 a.u.
Gibbs free energy $E_G$= -580.273565 a.u.

| | | | | |
|---|---|---|---|---|
| 7 | N | -2.04106 | -0.51287 | -0.01246 |
| 6 | C | -1.39158 | -1.70959 | -0.01466 |
| 7 | N | -0.06985 | -1.93659 | 0.005748 |
| 6 | C | 0.661116 | -0.80854 | 0.009067 |
| 6 | C | 0.088 | 0.494212 | -0.00371 |
| 6 | C | -1.31566 | 0.616104 | -0.00136 |
| 7 | N | 1.166346 | 1.364926 | -0.01208 |
| 6 | C | 2.30721 | 0.574273 | -0.00753 |
| 7 | N | 2.051107 | -0.71963 | 0.011181 |
| 7 | N | -1.98518 | 1.825501 | -0.05281 |
| 1 | H | -2.98168 | 1.746424 | 0.128563 |
| 1 | H | -1.54523 | 2.623345 | 0.391781 |
| 1 | H | 1.144893 | 2.3748 | -0.07907 |
| 1 | H | 3.300738 | 1.020144 | -0.01778 |
| 6 | C | -2.32768 | -2.89202 | -0.03374 |
| 8 | O | -1.98191 | -4.05803 | -0.02232 |
| 1 | H | -3.40499 | -2.58736 | -0.06031 |

C8-Cl-AD_7H $E_{tot}$ = -926.716931 a.u.
Gibbs free energy $E_G$= -926.651791 a.u.

| | | | | |
|---|---|---|---|---|
| 7 | N | -1.95875 | -0.50126 | -0.00946 |
| 6 | C | -1.31891 | -1.695 | -0.00885 |
| 7 | N | -0.00139 | -1.9409 | 0.011872 |
| 6 | C | 0.742744 | -0.81749 | 0.01264 |
| 6 | C | 0.182794 | 0.486047 | -0.00368 |
| 6 | C | -1.2169 | 0.622053 | -0.00155 |
| 7 | N | 1.276274 | 1.352138 | -0.01102 |
| 6 | C | 2.39393 | 0.539297 | -0.00469 |
| 7 | N | 2.136715 | -0.74853 | 0.015895 |
| 7 | N | -1.87474 | 1.839266 | -0.05544 |
| 1 | H | -2.87089 | 1.764327 | 0.130999 |
| 1 | H | -1.42946 | 2.627346 | 0.401958 |
| 1 | H | -1.97441 | -2.57178 | -0.0196 |
| 1 | H | 1.288121 | 2.360907 | -0.09081 |
| 17 | Cl | 3.963231 | 1.248462 | -0.02311 |

C2-Cl-AD_7H $E_{tot}$ = -926.721057 a.u.
Gibbs free energy $E_G$= -926.656036 a.u.

| | | | | |
|---|---|---|---|---|
| 7 | N | -1.96296 | -0.50317 | -0.00367 |
| 6 | C | -1.3148 | -1.68161 | -0.00791 |
| 7 | N | -0.01408 | -1.94355 | 0.007715 |
| 6 | C | 0.738561 | -0.82027 | 0.010126 |
| 6 | C | 0.183463 | 0.485478 | -0.0012 |
| 6 | C | -1.21813 | 0.61867 | 0.002448 |
| 7 | N | 1.27774 | 1.342771 | -0.00903 |
| 6 | C | 2.402496 | 0.5372 | -0.00683 |
| 7 | N | 2.125372 | -0.75604 | 0.010651 |
| 7 | N | -1.88273 | 1.827707 | -0.04844 |
| 1 | H | -2.87933 | 1.756635 | 0.13462 |
| 1 | H | -1.43303 | 2.630535 | 0.375864 |
| 1 | H | 1.271914 | 2.352642 | -0.07563 |
| 17 | Cl | -2.37746 | -3.08891 | -0.02569 |
| 1 | H | 3.40383 | 0.964717 | -0.01749 |

C8-F-AD_7H $E_{tot}$ = -566.287822 a.u.
Gibbs free energy $E_G$= -566.220271 a.u.

| | | | | |
|---|---|---|---|---|
| 7 | N | -1.95888 | -0.50066 | -0.01127 |
| 6 | C | -1.32231 | -1.69395 | -0.01056 |
| 7 | N | -0.00371 | -1.94212 | 0.011935 |
| 6 | C | 0.741412 | -0.82175 | 0.012946 |
| 6 | C | 0.183237 | 0.483026 | -0.00353 |
| 6 | C | -1.21361 | 0.622331 | -0.00131 |
| 7 | N | 1.283625 | 1.350856 | -0.00497 |
| 6 | C | 2.382742 | 0.523988 | -0.00227 |
| 7 | N | 2.13917 | -0.75681 | 0.019163 |
| 7 | N | -1.87169 | 1.840279 | -0.05439 |
| 1 | H | -2.86781 | 1.762665 | 0.131443 |
| 1 | H | -1.42918 | 2.625479 | 0.41074 |
| 1 | H | -1.97824 | -2.57042 | -0.02191 |
| 1 | H | 1.301231 | 2.357335 | -0.11065 |
| 9 | F | 3.594846 | 1.076551 | -0.01678 |

C2-F-AD_7H $E_{tot}$ = -566.298761 a.u.
Gibbs free energy $E_G$= -566.230971 a.u.

| | | | | |
|---|---|---|---|---|
| 7 | N | -1.96518 | -0.50559 | -0.00485 |
| 6 | C | -1.3079 | -1.67291 | -0.0087 |
| 7 | N | -0.01513 | -1.94667 | 0.006793 |
| 6 | C | 0.738782 | -0.82282 | 0.009409 |
| 6 | C | 0.18172 | 0.482473 | -0.00121 |
| 6 | C | -1.21984 | 0.616461 | 0.002052 |
| 7 | N | 1.276353 | 1.34131 | -0.00757 |
| 6 | C | 2.400667 | 0.537202 | -0.00628 |
| 7 | N | 2.124269 | -0.75699 | 0.010387 |
| 7 | N | -1.88343 | 1.825793 | -0.04797 |
| 1 | H | -2.8795 | 1.758043 | 0.138332 |
| 1 | H | -1.43036 | 2.629197 | 0.371339 |
| 1 | H | 1.270184 | 2.350992 | -0.0753 |
| 9 | F | -2.12548 | -2.75082 | -0.02055 |
| 1 | H | 3.401919 | 0.964965 | -0.0166 |

C8-H-AD_7H $E_{tot}$ = -467.085338 a.u.
Gibbs free energy $E_G$= -467.00833 a.u.

| | | | | |
|---|---|---|---|---|
| 7 | N | -1.96277 | -0.50017 | -0.01 |
| 6 | C | -1.32248 | -1.69578 | -0.00871 |
| 7 | N | -0.0069 | -1.94233 | 0.012901 |
| 6 | C | 0.742057 | -0.8186 | 0.01347 |
| 6 | C | 0.182197 | 0.48572 | -0.00383 |
| 6 | C | -1.2189 | 0.620205 | -0.00265 |
| 7 | N | 1.272567 | 1.348798 | -0.01317 |
| 6 | C | 2.402066 | 0.548237 | -0.00608 |
| 7 | N | 2.131882 | -0.74505 | 0.016318 |
| 7 | N | -1.87607 | 1.839342 | -0.05822 |
| 1 | H | -2.87193 | 1.76392 | 0.130168 |
| 1 | H | -1.4308 | 2.624304 | 0.404726 |
| 1 | H | -1.98003 | -2.57131 | -0.01873 |
| 1 | H | 3.400837 | 0.982174 | -0.01724 |
| 1 | H | 1.261259 | 2.358063 | -0.08695 |

C2-H-AD_7H $E_{tot}$ = -467.085338 a.u.
Gibbs free energy $E_G$= -467.00833 a.u.

| | | | | |
|---|---|---|---|---|
| 7 | N | -1.96277 | -0.50017 | -0.01 |
| 6 | C | -1.32248 | -1.69578 | -0.00871 |
| 7 | N | -0.0069 | -1.94233 | 0.012901 |
| 6 | C | 0.742057 | -0.8186 | 0.01347 |
| 6 | C | 0.182197 | 0.48572 | -0.00383 |
| 6 | C | -1.2189 | 0.620205 | -0.00265 |
| 7 | N | 1.272567 | 1.348798 | -0.01317 |
| 6 | C | 2.402066 | 0.548237 | -0.00608 |
| 7 | N | 2.131882 | -0.74505 | 0.016318 |
| 7 | N | -1.87607 | 1.839342 | -0.05822 |
| 1 | H | -2.87193 | 1.76392 | 0.130168 |
| 1 | H | -1.4308 | 2.624304 | 0.404726 |
| 1 | H | -1.98003 | -2.57131 | -0.01873 |
| 1 | H | 3.400837 | 0.982174 | -0.01724 |
| 1 | H | 1.261259 | 2.358063 | -0.08695 |

C8-Me-AD_7H $E_{tot}$ = -506.387571 a.u.
Gibbs free energy $E_G$= -506.288694 a.u.

| | | | | |
|---|---|---|---|---|
| 7 | N | -1.96162 | -0.49656 | -0.01316 |
| 6 | C | -1.32795 | -1.6943 | -0.00969 |
| 7 | N | -0.01226 | -1.94648 | 0.014821 |
| 6 | C | 0.741832 | -0.82699 | 0.01535 |
| 6 | C | 0.188129 | 0.479216 | -0.0047 |
| 6 | C | -1.20994 | 0.620215 | -0.00531 |
| 7 | N | 1.284606 | 1.335209 | -0.01193 |
| 6 | C | 2.421692 | 0.535447 | -0.00343 |
| 7 | N | 2.130636 | -0.75835 | 0.021007 |
| 7 | N | -1.86205 | 1.84491 | -0.06291 |
| 1 | H | -2.85782 | 1.768747 | 0.127214 |
| 1 | H | -1.41791 | 2.619042 | 0.419812 |
| 1 | H | -1.98864 | -2.56749 | -0.02022 |
| 1 | H | 1.275957 | 2.343873 | -0.09482 |
| 6 | C | 3.797599 | 1.115011 | -0.02216 |
| 1 | H | 3.967803 | 1.707928 | -0.9371 |
| 1 | H | 3.964895 | 1.775544 | 0.845443 |
| 1 | H | 4.525217 | 0.294768 | 0.008704 |

C2-Me-AD_7H $E_{tot}$ = -506.385299 a.u.
Gibbs free energy $E_G$= -506.284691 a.u.

| | | | | |
|---|---|---|---|---|
| 7 | N | -1.95518 | -0.50342 | -0.00291 |
| 6 | C | -1.32927 | -1.7151 | -0.00614 |
| 7 | N | -0.00544 | -1.94077 | 0.012202 |
| 6 | C | 0.742092 | -0.81759 | 0.011282 |
| 6 | C | 0.186536 | 0.486219 | -0.00452 |
| 6 | C | -1.21481 | 0.614578 | 0.00157 |
| 7 | N | 1.277982 | 1.348659 | -0.0165 |
| 6 | C | 2.405867 | 0.546626 | -0.01271 |
| 7 | N | 2.132852 | -0.7467 | 0.010964 |
| 7 | N | -1.87449 | 1.834449 | -0.05197 |
| 1 | H | -2.86875 | 1.754174 | 0.143853 |
| 1 | H | -1.42992 | 2.614782 | 0.419939 |
| 1 | H | 1.267168 | 2.357594 | -0.09408 |
| 6 | C | -2.23899 | -2.91685 | -0.02423 |
| 1 | H | -2.92176 | -2.89108 | 0.840793 |
| 1 | H | -2.86613 | -2.90347 | -0.93105 |
| 1 | H | -1.64461 | -3.83877 | -0.00051 |
| 1 | H | 3.40551 | 0.978495 | -0.0266 |

C8-OMe-AD_7H $E_{tot}$ = -581.568845 a.u.
Gibbs free energy $E_G$= -581.464301 a.u.

| | | | | |
|---|---|---|---|---|
| 7 | N | 2.784998 | 0.21089 | 0.018535 |
| 6 | C | 2.655038 | -1.13404 | 0.017309 |
| 7 | N | 1.531583 | -1.86992 | -0.00846 |
| 6 | C | 0.410971 | -1.12346 | -0.01234 |
| 6 | C | 0.427089 | 0.29607 | 0.00486 |
| 6 | C | 1.661005 | 0.958412 | 0.005268 |
| 7 | N | -0.9195 | 0.677888 | -0.00099 |
| 6 | C | -1.64098 | -0.50156 | -0.00205 |
| 7 | N | -0.90075 | -1.59584 | -0.0223 |
| 7 | N | 1.798939 | 2.340628 | 0.058981 |
| 1 | H | 2.750201 | 2.643459 | -0.13364 |
| 1 | H | 1.099481 | 2.882541 | -0.43833 |
| 1 | H | 3.596911 | -1.69242 | 0.031403 |
| 1 | H | -1.31476 | 1.601192 | 0.12064 |
| 8 | O | -2.97522 | -0.41802 | 0.013844 |
| 6 | C | -3.66417 | -1.69904 | 0.016914 |
| 1 | H | -3.4062 | -2.26493 | -0.89005 |
| 1 | H | -3.37522 | -2.27751 | 0.906485 |
| 1 | H | -4.73029 | -1.44398 | 0.036902 |

C2-OMe-AD_7H $E_{tot}$ = -581.571698 a.u.
Gibbs free energy $E_G$= -581.466513 a.u.

| | | | | |
|---|---|---|---|---|
| 7 | N | -1.17118 | 0.746123 | 0.008924 |
| 6 | C | -1.28018 | -0.60615 | 0.01049 |
| 7 | N | -0.31051 | -1.52667 | 0.026179 |
| 6 | C | 0.92903 | -0.99006 | 0.016863 |
| 6 | C | 1.168908 | 0.405401 | -0.00477 |
| 6 | C | 0.059205 | 1.274876 | 0.003082 |
| 7 | N | 2.555867 | 0.535878 | -0.02212 |
| 6 | C | 3.065965 | -0.74763 | -0.01648 |
| 7 | N | 2.131834 | -1.68629 | 0.013889 |
| 7 | N | 0.168505 | 2.655337 | -0.05679 |
| 1 | H | -0.70628 | 3.133586 | 0.140391 |
| 1 | H | 0.974237 | 3.071066 | 0.397485 |
| 1 | H | 3.094685 | 1.387976 | -0.10804 |
| 8 | O | -2.57193 | -1.01611 | 0.005856 |
| 1 | H | 4.139282 | -0.93016 | -0.03404 |
| 6 | C | -2.79089 | -2.44255 | 0.016496 |
| 1 | H | -3.88242 | -2.55775 | 0.010374 |
| 1 | H | -2.34162 | -2.91142 | -0.87241 |
| 1 | H | -2.35319 | -2.89636 | 0.918924 |

C8-OMe-AD_7H $E_{tot}$ = -581.559228 a.u.
Gibbs free energy $E_G$= -581.454953 a.u.

| | | | | |
|---|---|---|---|---|
| 7 | N | -1.93794 | -0.48467 | -0.01185 |
| 6 | C | -1.29947 | -1.67702 | -0.00485 |
| 7 | N | 0.01921 | -1.92543 | 0.022058 |
| 6 | C | 0.769375 | -0.80549 | 0.015892 |
| 6 | C | 0.206014 | 0.496536 | -0.00858 |
| 6 | C | -1.18677 | 0.636002 | -0.00448 |
| 7 | N | 1.300563 | 1.371007 | -0.00618 |
| 6 | C | 2.42739 | 0.552541 | -0.0137 |
| 7 | N | 2.157923 | -0.73765 | 0.021206 |
| 7 | N | -1.84316 | 1.862138 | -0.06034 |
| 1 | H | -2.83905 | 1.771906 | 0.125725 |
| 1 | H | -1.41358 | 2.620301 | 0.461049 |
| 1 | H | -1.95506 | -2.55417 | -0.01378 |
| 1 | H | 1.275874 | 2.363215 | -0.20017 |
| 8 | O | 3.684105 | 1.023484 | -0.03642 |
| 6 | C | 3.857207 | 2.451355 | -0.05209 |
| 1 | H | 4.94098 | 2.61459 | -0.03788 |
| 1 | H | 3.433773 | 2.88946 | -0.97357 |
| 1 | H | 3.401189 | 2.912071 | 0.841445 |

C2-OMe-AD_7H $E_{tot}$ = -581.569196 a.u.
Gibbs free energy $E_G$= -581.464275 a.u.

| | | | | |
|---|---|---|---|---|
| 7 | N | -1.98637 | -0.49806 | 0.004537 |
| 6 | C | -1.35407 | -1.69608 | 0.005084 |
| 7 | N | -0.04018 | -1.95178 | 0.023541 |
| 6 | C | 0.709678 | -0.83524 | 0.016674 |
| 6 | C | 0.163653 | 0.476894 | -0.00448 |
| 6 | C | -1.23284 | 0.616023 | 0.002763 |
| 7 | N | 1.265014 | 1.33076 | -0.01772 |
| 6 | C | 2.383036 | 0.518224 | -0.0111 |
| 7 | N | 2.100555 | -0.77367 | 0.017439 |
| 7 | N | -1.88963 | 1.836329 | -0.05408 |
| 1 | H | -2.88156 | 1.767046 | 0.154995 |
| 1 | H | -1.43267 | 2.621635 | 0.396622 |
| 1 | H | 1.265111 | 2.33864 | -0.10591 |
| 8 | O | -2.12578 | -2.81204 | -0.00355 |
| 1 | H | 3.386853 | 0.940452 | -0.02652 |
| 6 | C | -3.5533 | -2.62875 | -0.02749 |
| 1 | H | -3.86325 | -2.07299 | -0.92652 |
| 1 | H | -3.96612 | -3.64588 | -0.04129 |
| 1 | H | -3.89463 | -2.08511 | 0.867737 |

C8-OH-AD_7H $E_{tot}$ = -542.292302 a.u.
Gibbs free energy $E_G$= -542.212344 a.u.

| | | | | |
|---|---|---|---|---|
| 7 | N | -2.40804 | -0.16261 | 4.04E-05 |
| 6 | C | -2.01331 | -1.45475 | 0.001022 |
| 7 | N | -0.76579 | -1.95258 | 0.008835 |
| 6 | C | 0.183065 | -0.99803 | -0.00719 |
| 6 | C | -0.11523 | 0.390201 | -0.02653 |
| 6 | C | -1.45608 | 0.794447 | -0.007 |
| 7 | N | 1.130381 | 1.032503 | -0.0462 |
| 6 | C | 2.065343 | 0.021617 | -0.05242 |
| 7 | N | 1.563621 | -1.19714 | -0.0184 |
| 7 | N | -1.86834 | 2.119641 | -0.06012 |
| 1 | H | -2.85777 | 2.229344 | 0.14586 |
| 1 | H | -1.28164 | 2.796904 | 0.416168 |
| 1 | H | -2.8253 | -2.18937 | 0.003725 |
| 1 | H | 1.332716 | 2.016766 | -0.1644 |
| 8 | O | 3.369488 | 0.347685 | -0.08872 |
| 1 | H | 3.860133 | -0.49273 | -0.09302 |

C2-OH-AD_7H $E_{tot}$ = -542.297497 a.u.
Gibbs free energy $E_G$= -542.217166 a.u.

| | | | | |
|---|---|---|---|---|
| 7 | N | -1.59514 | 0.74378 | -0.00116 |
| 6 | C | -1.6849 | -0.60279 | -0.00055 |
| 7 | N | -0.70887 | -1.51907 | 0.017972 |
| 6 | C | 0.525843 | -0.97335 | 0.014695 |
| 6 | C | 0.749811 | 0.426869 | -0.00312 |
| 6 | C | -0.36625 | 1.28527 | 0.000267 |
| 7 | N | 2.135743 | 0.57147 | -0.01488 |
| 6 | C | 2.658961 | -0.70631 | -0.00933 |
| 7 | N | 1.735582 | -1.65567 | 0.015305 |
| 7 | N | -0.2745 | 2.665074 | -0.05651 |
| 1 | H | -1.15443 | 3.135653 | 0.135026 |
| 1 | H | 0.529681 | 3.094245 | 0.387199 |
| 1 | H | 2.666582 | 1.429099 | -0.09512 |
| 1 | H | 3.73425 | -0.87731 | -0.02282 |
| 8 | O | -2.96218 | -1.06862 | -0.01121 |
| 1 | H | -2.88152 | -2.03862 | -0.00453 |

C8-OH-AD_7H  $E_{tot}$ = -542.281660 a.u.
Gibbs free energy  $E_G$= -542.20272 a.u.

| | | | | |
|---|---|---|---|---|
| 7 | N | -1.94581 | -0.49057 | -0.01117 |
| 6 | C | -1.30825 | -1.68234 | -0.00939 |
| 7 | N | 0.011649 | -1.92983 | 0.015022 |
| 6 | C | 0.759117 | -0.81042 | 0.007808 |
| 6 | C | 0.197895 | 0.49112 | -0.01318 |
| 6 | C | -1.19491 | 0.63087 | 0.000163 |
| 7 | N | 1.29475 | 1.368609 | 0.005172 |
| 6 | C | 2.417796 | 0.542178 | -0.01405 |
| 7 | N | 2.152096 | -0.74295 | 0.020448 |
| 7 | N | -1.8504 | 1.856215 | -0.04464 |
| 1 | H | -2.84551 | 1.768767 | 0.145979 |
| 1 | H | -1.41366 | 2.613977 | 0.47124 |
| 1 | H | -1.96294 | -2.55999 | -0.01943 |
| 1 | H | 1.276488 | 2.332269 | -0.3096 |
| 8 | O | 3.674739 | 1.03107 | -0.0553 |
| 1 | H | 3.66824 | 1.98664 | 0.109126 |

C2-OH-AD_7H  $E_{tot}$ = -542.295711 a.u.
Gibbs free energy  $E_G$= -542.215488 a.u.

| | | | | |
|---|---|---|---|---|
| 7 | N | -1.96797 | -0.53171 | -0.00775 |
| 6 | C | -1.31543 | -1.72309 | -0.00974 |
| 7 | N | -0.00646 | -1.96778 | 0.01064 |
| 6 | C | 0.732121 | -0.83892 | 0.011604 |
| 6 | C | 0.17049 | 0.464971 | -0.00426 |
| 6 | C | -1.22969 | 0.591476 | -0.00246 |
| 7 | N | 1.26098 | 1.331752 | -0.01108 |
| 6 | C | 2.388748 | 0.533511 | -0.0051 |
| 7 | N | 2.119973 | -0.76218 | 0.01605 |
| 7 | N | -1.89603 | 1.804501 | -0.05769 |
| 1 | H | -2.88941 | 1.73074 | 0.141838 |
| 1 | H | -1.44656 | 2.59537 | 0.390209 |
| 1 | H | 1.248972 | 2.340446 | -0.08936 |
| 1 | H | 3.387584 | 0.967234 | -0.01548 |
| 8 | O | -2.11714 | -2.82151 | -0.02445 |
| 1 | H | -3.0287 | -2.48391 | -0.03804 |

C8-NH2-AD_7H  $E_{tot}$ = -522.430336 a.u.
Gibbs free energy  $E_G$= -522.338657 a.u.

| | | | | |
|---|---|---|---|---|
| 7 | N | -1.93757 | -0.49698 | 0.006836 |
| 6 | C | -1.29406 | -1.68474 | 0.014114 |
| 7 | N | 0.027849 | -1.92563 | 0.025122 |
| 6 | C | 0.770638 | -0.80228 | -0.00306 |
| 6 | C | 0.202257 | 0.49697 | -0.03242 |
| 6 | C | -1.18999 | 0.628127 | -0.00222 |
| 7 | N | 1.29198 | 1.380881 | -0.0314 |
| 6 | C | 2.425938 | 0.573025 | -0.04957 |
| 7 | N | 2.160672 | -0.72372 | 0.001381 |
| 7 | N | -1.85323 | 1.850544 | -0.05147 |
| 1 | H | -2.84386 | 1.75945 | 0.159506 |
| 1 | H | -1.41094 | 2.615041 | 0.449673 |
| 1 | H | -1.94411 | -2.56606 | 0.021235 |
| 1 | H | 1.273429 | 2.338698 | -0.36111 |
| 7 | N | 3.687295 | 1.119287 | -0.17515 |
| 1 | H | 4.415744 | 0.4264 | -0.02716 |
| 1 | H | 3.851412 | 1.974813 | 0.347514 |

C2-NH2-AD_7H  $E_{tot}$ = -522.436103 a.u.
Gibbs free energy  $E_G$= -522.344372 a.u.

| | | | | |
|---|---|---|---|---|
| 7 | N | -1.98356 | -0.51386 | 0.031323 |
| 6 | C | -1.34715 | -1.72119 | 0.05001 |
| 7 | N | -0.02282 | -1.96304 | 0.055132 |
| 6 | C | 0.721525 | -0.84163 | 0.035878 |
| 6 | C | 0.167422 | 0.464482 | 0.010102 |
| 6 | C | -1.23312 | 0.596212 | 0.013945 |
| 7 | N | 1.263371 | 1.326265 | -0.02274 |
| 6 | C | 2.386506 | 0.523074 | -0.02124 |
| 7 | N | 2.111852 | -0.77159 | 0.019953 |
| 7 | N | -1.88801 | 1.818212 | -0.06577 |
| 1 | H | -2.87967 | 1.750105 | 0.146327 |
| 1 | H | -1.43266 | 2.604602 | 0.385151 |
| 1 | H | 1.254956 | 2.333535 | -0.11648 |
| 1 | H | 3.387307 | 0.951485 | -0.04935 |
| 7 | N | -2.17951 | -2.82119 | 0.122191 |
| 1 | H | -1.75211 | -3.70611 | -0.12208 |
| 1 | H | -3.13507 | -2.67619 | -0.17829 |

C8-$NO_2$-AD_3H $E_{tot}$ = -671.517325 a.u.
Gibbs free energy $E_G$= -671.444252 a.u.

| | | | | |
|---|---|---|---|---|
| 7 | N | 1.942399 | 0.350453 | 0.000333 |
| 6 | C | 1.357998 | 1.532717 | 9E-05 |
| 7 | N | 0.01124 | 1.7428 | -0.00024 |
| 6 | C | -0.82023 | 0.644681 | -0.00019 |
| 6 | C | -0.24232 | -0.65908 | 8.61E-05 |
| 6 | C | 1.164623 | -0.78092 | 0.000252 |
| 7 | N | -1.2569 | -1.57917 | 0.000241 |
| 6 | C | -2.33617 | -0.77557 | 0.000129 |
| 7 | N | -2.15781 | 0.585122 | -0.00031 |
| 7 | N | 1.794987 | -1.97211 | -0.00012 |
| 1 | H | 2.805669 | -2.00638 | 0.00221 |
| 1 | H | 1.251093 | -2.82546 | 0.00182 |
| 1 | H | 1.981206 | 2.429908 | 0.00013 |
| 1 | H | -0.37253 | 2.684577 | -0.00045 |
| 7 | N | -3.6986 | -1.34202 | 0.000294 |
| 8 | O | -4.6382 | -0.54297 | -2.6E-05 |
| 8 | O | -3.79635 | -2.57132 | 0.000745 |

C2-$NO_2$-AD_3H $E_{tot}$ = -671.516501 a.u.
Gibbs free energy $E_G$= -671.442059 a.u.

| | | | | |
|---|---|---|---|---|
| 7 | N | 1.962134 | 0.430105 | 0.000244 |
| 6 | C | 1.376937 | 1.59742 | 2.7E-05 |
| 7 | N | 0.043212 | 1.84366 | -0.00027 |
| 6 | C | -0.79382 | 0.75882 | -0.00022 |
| 6 | C | -0.22538 | -0.55981 | 1.01E-05 |
| 6 | C | 1.179861 | -0.69486 | 0.00014 |
| 7 | N | -1.25009 | -1.46273 | 0.000112 |
| 6 | C | -2.34516 | -0.65573 | 0.000265 |
| 7 | N | -2.1341 | 0.705543 | -0.00028 |
| 7 | N | 1.806112 | -1.89151 | -0.00046 |
| 1 | H | 2.816201 | -1.93253 | 0.002552 |
| 1 | H | 1.256123 | -2.74012 | 0.002085 |
| 1 | H | -3.35672 | -1.06315 | 0.000266 |
| 1 | H | -0.27689 | 2.812888 | -0.00044 |
| 7 | N | 2.219556 | 2.831299 | 0.000155 |
| 8 | O | 1.60186 | 3.909268 | -0.00013 |
| 8 | O | 3.434895 | 2.694674 | 0.000616 |

C8-CN-AD_3H $E_{tot}$ = -559.278473 a.u.
Gibbs free energy $E_G$= -559.205701 a.u.

| | | | | |
|---|---|---|---|---|
| 7 | N | 1.937225 | 0.348865 | 0.000303 |
| 6 | C | 1.353518 | 1.53086 | 6.01E-05 |
| 7 | N | 0.005653 | 1.738676 | -0.00027 |
| 6 | C | -0.82641 | 0.641601 | -0.00019 |
| 6 | C | -0.24875 | -0.6616 | 8.33E-05 |
| 6 | C | 1.157485 | -0.78161 | 0.000214 |
| 7 | N | -1.2614 | -1.58384 | 0.000153 |
| 6 | C | -2.36401 | -0.78813 | 0.000429 |
| 7 | N | -2.16356 | 0.585096 | -0.00032 |
| 7 | N | 1.789495 | -1.97373 | -0.00031 |
| 1 | H | 2.800018 | -2.00691 | 0.002384 |
| 1 | H | 1.24644 | -2.82724 | 0.001993 |
| 1 | H | 1.975327 | 2.428951 | 8.99E-05 |
| 1 | H | -0.37833 | 2.680183 | -0.00051 |
| 6 | C | -3.6864 | -1.33644 | 0.00035 |
| 7 | N | -4.76917 | -1.78657 | 0.000273 |

C2-CN-AD_3H $E_{tot}$ = -559.270093 a.u.
Gibbs free energy $E_G$= -559.198058 a.u.

| | | | | |
|---|---|---|---|---|
| 7 | N | 1.969919 | 0.441826 | 9.36E-05 |
| 6 | C | 1.395761 | 1.635248 | -7.4E-05 |
| 7 | N | 0.039807 | 1.852506 | -0.00015 |
| 6 | C | -0.79039 | 0.76321 | -6.6E-05 |
| 6 | C | -0.22026 | -0.55013 | 9.46E-05 |
| 6 | C | 1.18343 | -0.67957 | 0.000106 |
| 7 | N | -1.24488 | -1.45769 | 0.000654 |
| 6 | C | -2.33981 | -0.65502 | -0.00056 |
| 7 | N | -2.13095 | 0.708371 | 0.000241 |
| 7 | N | 1.810643 | -1.87713 | -0.00036 |
| 1 | H | 2.820565 | -1.91771 | 0.002493 |
| 1 | H | 1.260862 | -2.72582 | 0.002204 |
| 1 | H | -3.35103 | -1.06325 | -0.00013 |
| 1 | H | -0.33489 | 2.799067 | -0.00017 |
| 6 | C | 2.221994 | 2.811869 | 3.37E-05 |
| 7 | N | 2.864326 | 3.792561 | 0.00012 |

C8-CHO-AD_3H $E_{tot}$ = -580.361048 a.u.
Gibbs free energy $E_G$= -580.278319 a.u.

| | | | | |
|---|---|---|---|---|
| 7 | N | 1.946593 | 0.439622 | 0.000281 |
| 6 | C | 1.376372 | 1.62904 | 2.83E-05 |
| 7 | N | 0.032432 | 1.854398 | -0.00025 |
| 6 | C | -0.81755 | 0.768605 | -0.00016 |
| 6 | C | -0.25331 | -0.5452 | 0.000136 |
| 6 | C | 1.151785 | -0.6804 | 0.000281 |
| 7 | N | -1.27955 | -1.44656 | 0.00039 |
| 6 | C | -2.38045 | -0.63826 | 0.0002 |
| 7 | N | -2.15065 | 0.730415 | -0.00035 |
| 7 | N | 1.771135 | -1.87998 | 0.000139 |
| 1 | H | 2.781187 | -1.9244 | 0.002086 |
| 1 | H | 1.218196 | -2.72693 | 0.001823 |
| 1 | H | 2.0105 | 2.518611 | -2.9E-05 |
| 1 | H | -0.33959 | 2.800852 | -0.00049 |
| 6 | C | -3.72635 | -1.2488 | 0.00021 |
| 8 | O | -4.78329 | -0.63389 | 0.000619 |
| 1 | H | -3.69048 | -2.36785 | -0.00024 |

C2-CHO-AD_3H $E_{tot}$ = -580.364512 a.u.
Gibbs free energy $E_G$= -580.281952 a.u.

| | | | | |
|---|---|---|---|---|
| 7 | N | 1.967249 | 0.444632 | 0.000163 |
| 6 | C | 1.385433 | 1.639967 | -9.6E-06 |
| 7 | N | 0.036415 | 1.848571 | -0.00022 |
| 6 | C | -0.79491 | 0.767183 | -0.00014 |
| 6 | C | -0.21934 | -0.54858 | 2.03E-05 |
| 6 | C | 1.186738 | -0.67672 | 8.34E-05 |
| 7 | N | -1.237 | -1.45941 | 7.85E-05 |
| 6 | C | -2.33833 | -0.6589 | 0.000505 |
| 7 | N | -2.13752 | 0.702327 | -0.00012 |
| 7 | N | 1.807966 | -1.87954 | -0.00057 |
| 1 | H | 2.817389 | -1.9258 | 0.002422 |
| 1 | H | 1.253825 | -2.72519 | 0.001997 |
| 1 | H | -3.34678 | -1.07442 | 0.000512 |
| 1 | H | -0.2988 | 2.813487 | -0.00032 |
| 6 | C | 2.232923 | 2.858215 | 4.28E-05 |
| 8 | O | 1.762611 | 3.989998 | -0.00017 |
| 1 | H | 3.327529 | 2.652435 | 0.00045 |

C8-CHO-AD_3H $E_{tot}$ = -580.360469 a.u.
Gibbs free energy $E_G$= -580.277681 a.u.

| | | | | |
|---|---|---|---|---|
| 7 | N | 1.939583 | 0.360708 | 0.000362 |
| 6 | C | 1.340558 | 1.53633 | 0.000263 |
| 7 | N | -0.00876 | 1.728975 | -2.9E-05 |
| 6 | C | -0.83155 | 0.622528 | -0.0001 |
| 6 | C | -0.23697 | -0.67657 | 6.63E-06 |
| 6 | C | 1.172934 | -0.77768 | 0.00014 |
| 7 | N | -1.23144 | -1.61016 | 6.01E-05 |
| 6 | C | -2.35327 | -0.83954 | 0.000144 |
| 7 | N | -2.1642 | 0.541471 | -0.00019 |
| 7 | N | 1.814384 | -1.96414 | -0.00044 |
| 1 | H | 2.825047 | -1.99108 | 0.002133 |
| 1 | H | 1.274528 | -2.82018 | 0.001521 |
| 1 | H | 1.952489 | 2.44128 | 0.00041 |
| 1 | H | -0.40114 | 2.667024 | -0.00013 |
| 6 | C | -3.73452 | -1.36951 | 0.000204 |
| 8 | O | -4.03594 | -2.55454 | 0.000184 |
| 1 | H | -4.51003 | -0.56183 | 0.000219 |

C2-CHO-AD_3H $E_{tot}$ = -580.355066 a.u.
Gibbs free energy $E_G$= -580.272806 a.u.

| | | | | |
|---|---|---|---|---|
| 7 | N | 2.082648 | 0.391685 | 0.000418 |
| 6 | C | 1.530983 | 1.595821 | 0.000176 |
| 7 | N | 0.173406 | 1.816548 | -0.00021 |
| 6 | C | -0.67187 | 0.745214 | -0.00017 |
| 6 | C | -0.11845 | -0.57566 | 7.99E-05 |
| 6 | C | 1.288217 | -0.71711 | 0.000283 |
| 7 | N | -1.15031 | -1.46995 | -0.00012 |
| 6 | C | -2.23988 | -0.65238 | 0.000675 |
| 7 | N | -2.01689 | 0.704355 | -0.00035 |
| 7 | N | 1.896871 | -1.92435 | -0.00027 |
| 1 | H | 2.90654 | -1.97574 | 0.002199 |
| 1 | H | 1.336622 | -2.76601 | 0.001782 |
| 1 | H | -3.25454 | -1.05222 | 0.000468 |
| 1 | H | -0.19423 | 2.767138 | -0.00041 |
| 6 | C | 2.365103 | 2.831912 | 0.000283 |
| 8 | O | 3.580983 | 2.853228 | 0.000506 |
| 1 | H | 1.761939 | 3.781601 | -0.00011 |

C8-Cl-AD_3H $E_{tot}$ = -926.720746 a.u.
Gibbs free energy $E_G$= -926.655651 a.u.

| | | | | |
|---|---|---|---|---|
| 7 | N | 1.944357 | 0.352402 | 0.000109 |
| 6 | C | 1.364181 | 1.534826 | -2.3E-05 |
| 7 | N | 0.013865 | 1.740684 | -0.0002 |
| 6 | C | -0.81581 | 0.644167 | -0.00016 |
| 6 | C | -0.2391 | -0.65832 | -1.6E-05 |
| 6 | C | 1.162275 | -0.77821 | 9.3E-06 |
| 7 | N | -1.26291 | -1.58123 | 0.000195 |
| 6 | C | -2.34267 | -0.77843 | -7.2E-05 |
| 7 | N | -2.15773 | 0.586168 | -4E-05 |
| 7 | N | 1.798506 | -1.97313 | -0.0008 |
| 1 | H | 2.808502 | -2.00421 | 0.002816 |
| 1 | H | 1.256972 | -2.82682 | 0.002393 |
| 1 | H | 1.984988 | 2.4335 | 8.62E-05 |
| 1 | H | -0.37152 | 2.681324 | -0.00029 |
| 17 | Cl | -3.94641 | -1.42896 | 0.00033 |

C2-Cl-AD_3H $E_{tot}$ = -926.717529 a.u.
Gibbs free energy $E_G$= -926.65264 a.u.

| | | | | |
|---|---|---|---|---|
| 7 | N | 1.950454 | 0.455204 | 1.45E-05 |
| 6 | C | 1.370993 | 1.623858 | -0.00016 |
| 7 | N | 0.02822 | 1.849829 | -0.00023 |
| 6 | C | -0.80512 | 0.748054 | -0.00013 |
| 6 | C | -0.23166 | -0.55758 | 1.54E-05 |
| 6 | C | 1.168044 | -0.67787 | 1.39E-05 |
| 7 | N | -1.25976 | -1.4732 | 0.000397 |
| 6 | C | -2.35044 | -0.67645 | -0.00026 |
| 7 | N | -2.14226 | 0.693789 | 0.000212 |
| 7 | N | 1.811083 | -1.86767 | -0.00073 |
| 1 | H | 2.821059 | -1.89723 | 0.002857 |
| 1 | H | 1.269642 | -2.72169 | 0.00231 |
| 1 | H | -3.36249 | -1.08289 | 3.69E-05 |
| 1 | H | -0.34245 | 2.796156 | -0.00027 |
| 17 | Cl | 2.354684 | 3.068155 | 6.73E-05 |

C8-F-AD_3H $E_{tot}$ = -566.294574 a.u.
Gibbs free energy $E_G$= -566.227142 a.u.

| | | | | |
|---|---|---|---|---|
| 7 | N | 1.942541 | 0.352287 | 7.07E-05 |
| 6 | C | 1.363983 | 1.534793 | -2.7E-05 |
| 7 | N | 0.012879 | 1.739995 | -0.00018 |
| 6 | C | -0.81558 | 0.644249 | -0.00016 |
| 6 | C | -0.23934 | -0.65966 | -5E-05 |
| 6 | C | 1.160367 | -0.77864 | -4.3E-05 |
| 7 | N | -1.26643 | -1.58279 | 0.000145 |
| 6 | C | -2.33029 | -0.77191 | -4.8E-05 |
| 7 | N | -2.15928 | 0.585836 | -3.7E-05 |
| 7 | N | 1.798454 | -1.97405 | -0.0009 |
| 1 | H | 2.808333 | -2.00391 | 0.00292 |
| 1 | H | 1.258406 | -2.82847 | 0.002473 |
| 1 | H | 1.984578 | 2.433568 | 8.89E-05 |
| 1 | H | -0.37291 | 2.680417 | -0.00026 |
| 9 | F | -3.57022 | -1.26913 | 0.000298 |

C2-F-AD_3H $E_{tot}$ = -566.291563 a.u.
Gibbs free energy $E_G$= -566.224013 a.u.

| | | | | |
|---|---|---|---|---|
| 7 | N | 1.957967 | 0.453321 | 6.76E-05 |
| 6 | C | 1.366241 | 1.607954 | -6.8E-05 |
| 7 | N | 0.033841 | 1.853099 | -0.00018 |
| 6 | C | -0.80511 | 0.749031 | -9.1E-05 |
| 6 | C | -0.22844 | -0.55425 | 4.79E-05 |
| 6 | C | 1.170204 | -0.67906 | 5.39E-05 |
| 7 | N | -1.2581 | -1.47224 | 0.000544 |
| 6 | C | -2.34801 | -0.67897 | -0.0003 |
| 7 | N | -2.13973 | 0.694319 | 0.000104 |
| 7 | N | 1.809395 | -1.87011 | -0.00046 |
| 1 | H | 2.819202 | -1.9043 | 0.002665 |
| 1 | H | 1.26418 | -2.7218 | 0.002154 |
| 1 | H | -3.36023 | -1.08497 | -4.6E-05 |
| 1 | H | -0.32441 | 2.804612 | -0.00022 |
| 9 | F | 2.119618 | 2.725312 | 1.69E-05 |

C8-H-AD_3H $E_{tot}$ = -467.085495 a.u.
Gibbs free energy $E_G$= -467.008726 a.u.

| | | | | |
|---|---|---|---|---|
| 7 | N | 1.956751 | 0.45435 | 0.000391 |
| 6 | C | 1.376085 | 1.637636 | 0.000118 |
| 7 | N | 0.027617 | 1.844188 | -0.00022 |
| 6 | C | -0.80757 | 0.749745 | 0.000092 |
| 6 | C | -0.23136 | -0.55468 | 0.000106 |
| 6 | C | 1.170301 | -0.6737 | -6.9E-05 |
| 7 | N | -1.256 | -1.47343 | 0.000079 |
| 6 | C | -2.3504 | -0.67884 | 0.000098 |
| 7 | N | -2.14758 | 0.689367 | -5.4E-05 |
| 7 | N | 1.805039 | -1.86988 | -0.00118 |
| 1 | H | 2.814875 | -1.90314 | 0.00295 |
| 1 | H | 1.259925 | -2.72134 | 0.002325 |
| 1 | H | 1.998167 | 2.535542 | 0.000062 |
| 1 | H | -3.3607 | -1.09023 | 0.000181 |
| 1 | H | -0.35538 | 2.786045 | -0.00071 |

C2-H-AD_3H $E_{tot}$ = -467.085495 a.u.
Gibbs free energy $E_G$= -467.008726 a.u.

| | | | | |
|---|---|---|---|---|
| 7 | N | 1.956751 | 0.45435 | 0.000391 |
| 6 | C | 1.376085 | 1.637636 | 0.000118 |
| 7 | N | 0.027617 | 1.844188 | -0.00022 |
| 6 | C | -0.80757 | 0.749745 | 0.000092 |
| 6 | C | -0.23136 | -0.55468 | 0.000106 |
| 6 | C | 1.170301 | -0.6737 | -6.9E-05 |
| 7 | N | -1.256 | -1.47343 | 0.000079 |
| 6 | C | -2.3504 | -0.67884 | 0.000098 |
| 7 | N | -2.14758 | 0.689367 | -5.4E-05 |
| 7 | N | 1.805039 | -1.86988 | -0.00118 |
| 1 | H | 2.814875 | -1.90314 | 0.00295 |
| 1 | H | 1.259925 | -2.72134 | 0.002325 |
| 1 | H | 1.998167 | 2.535542 | 0.000062 |
| 1 | H | -3.3607 | -1.09023 | 0.000181 |
| 1 | H | -0.35538 | 2.786045 | -0.00071 |

C8-Me-AD_3H $E_{tot}$ = -506.386585 a.u.
Gibbs free energy $E_G$= -506.287823 a.u.

| | | | | |
|---|---|---|---|---|
| 7 | N | 1.954797 | 0.453989 | 0.008523 |
| 6 | C | 1.375169 | 1.634386 | -0.06502 |
| 7 | N | 0.024862 | 1.838206 | -0.1008 |
| 6 | C | -0.80932 | 0.744414 | -0.05774 |
| 6 | C | -0.23152 | -0.55755 | 0.022239 |
| 6 | C | 1.167758 | -0.6739 | 0.054013 |
| 7 | N | -1.25768 | -1.47401 | 0.052735 |
| 6 | C | -2.36309 | -0.69055 | -0.00902 |
| 7 | N | -2.14606 | 0.682377 | -0.07864 |
| 7 | N | 1.805493 | -1.86874 | 0.127953 |
| 1 | H | 2.814837 | -1.89899 | 0.15448 |
| 1 | H | 1.261789 | -2.71973 | 0.168963 |
| 1 | H | 1.995881 | 2.532558 | -0.10139 |
| 1 | H | -0.35857 | 2.777819 | -0.15788 |
| 6 | C | -3.74914 | -1.24938 | -0.00394 |
| 1 | H | -4.29001 | -0.94927 | -0.91729 |
| 1 | H | -3.71157 | -2.34536 | 0.054097 |
| 1 | H | -4.3209 | -0.8564 | 0.853658 |

C2-Me-AD_3H $E_{tot}$ = -506.386326 a.u.
Gibbs free energy $E_G$= -506.284818 a.u.

| | | | | |
|---|---|---|---|---|
| 7 | N | 1.95918 | 0.443145 | 8.43E-05 |
| 6 | C | 1.399987 | 1.643224 | -2.9E-05 |
| 7 | N | 0.044053 | 1.835623 | -0.00016 |
| 6 | C | -0.79668 | 0.744786 | -0.00013 |
| 6 | C | -0.2308 | -0.56118 | -3.2E-05 |
| 6 | C | 1.168214 | -0.68058 | -3E-05 |
| 7 | N | -1.2631 | -1.47542 | 0.00029 |
| 6 | C | -2.3506 | -0.67425 | -0.0002 |
| 7 | N | -2.13681 | 0.694627 | 0.000121 |
| 7 | N | 1.802556 | -1.87821 | -0.00089 |
| 1 | H | 2.812195 | -1.91235 | 0.003031 |
| 1 | H | 1.256466 | -2.7289 | 0.002404 |
| 1 | H | -3.36432 | -1.07729 | 3.44E-05 |
| 1 | H | -0.34402 | 2.775182 | -0.0002 |
| 6 | C | 2.26599 | 2.872665 | 0.000124 |
| 1 | H | 2.07211 | 3.4929 | -0.89176 |
| 1 | H | 2.071441 | 3.493193 | 0.89166 |
| 1 | H | 3.318827 | 2.567453 | 0.000549 |

C8-OMe-AD_3H $E_{tot}$ = -581.568580 a.u.
Gibbs free energy $E_G$= -581.463461 a.u.

| | | | | |
|---|---|---|---|---|
| 7 | N | 1.963561 | 0.389726 | -0.00019 |
| 6 | C | 1.35879 | 1.557638 | -0.00017 |
| 7 | N | 0.000397 | 1.728438 | -0.00018 |
| 6 | C | -0.80373 | 0.614591 | -0.0001 |
| 6 | C | -0.19406 | -0.67632 | -0.00013 |
| 6 | C | 1.205376 | -0.7593 | -0.00027 |
| 7 | N | -1.19917 | -1.62098 | 0.000143 |
| 6 | C | -2.30466 | -0.84079 | 0.000325 |
| 7 | N | -2.14046 | 0.531152 | 0.000152 |
| 7 | N | 1.877612 | -1.94142 | -0.00137 |
| 1 | H | 2.887446 | -1.94351 | 0.003466 |
| 1 | H | 1.36116 | -2.80944 | 0.003 |
| 1 | H | 1.956032 | 2.472018 | -9.8E-05 |
| 1 | H | -0.40913 | 2.658534 | -0.00015 |
| 8 | O | -3.55835 | -1.32005 | 0.000564 |
| 6 | C | -3.67081 | -2.75833 | 0.000611 |
| 1 | H | -4.74906 | -2.96214 | 0.000787 |
| 1 | H | -3.19248 | -3.1814 | -0.89676 |
| 1 | H | -3.1922 | -3.18137 | 0.897846 |

C2-OMe-AD_3H $E_{tot}$ = -581.570115 a.u.
Gibbs free energy $E_G$= -581.466612 a.u.

| | | | | |
|---|---|---|---|---|
| 7 | N | 1.972319 | 0.350517 | 9.59E-05 |
| 6 | C | 1.430021 | 1.549325 | 1.33E-05 |
| 7 | N | 0.090666 | 1.809088 | -0.0002 |
| 6 | C | -0.77874 | 0.733367 | -0.00018 |
| 6 | C | -0.24618 | -0.58595 | -0.00011 |
| 6 | C | 1.147034 | -0.74958 | -8.7E-05 |
| 7 | N | -1.30516 | -1.47431 | 4.05E-05 |
| 6 | C | -2.36813 | -0.6465 | 0.000204 |
| 7 | N | -2.11641 | 0.719831 | -0.00011 |
| 7 | N | 1.743392 | -1.96645 | -0.001 |
| 1 | H | 2.750674 | -2.03878 | 0.003198 |
| 1 | H | 1.1662 | -2.79647 | 0.002294 |
| 1 | H | -3.39292 | -1.02014 | 0.000323 |
| 1 | H | -0.24321 | 2.768701 | -0.0003 |
| 8 | O | 2.17722 | 2.672715 | 0.000137 |
| 6 | C | 3.612384 | 2.486442 | 0.000139 |
| 1 | H | 4.023796 | 3.502865 | 9.38E-05 |
| 1 | H | 3.925099 | 1.934533 | 0.89874 |
| 1 | H | 3.92509 | 1.934444 | -0.89841 |

C8-OMe-AD_3H  $E_{tot}$ = -581.567454 a.u.
Gibbs free energy  $E_G$= -581.462549 a.u.

| | | | | |
|---|---|---|---|---|
| 7 | N | 1.911482 | 0.354717 | 4.32E-06 |
| 6 | C | 1.331842 | 1.536397 | -3.9E-05 |
| 7 | N | -0.02353 | 1.733605 | -0.0002 |
| 6 | C | -0.84858 | 0.636522 | -0.0002 |
| 6 | C | -0.26936 | -0.66658 | -0.00016 |
| 6 | C | 1.131282 | -0.777 | -0.00017 |
| 7 | N | -1.28568 | -1.5933 | 2.28E-07 |
| 6 | C | -2.37806 | -0.7983 | 3.93E-05 |
| 7 | N | -2.19006 | 0.573896 | -8.9E-05 |
| 7 | N | 1.772294 | -1.97474 | -0.0012 |
| 1 | H | 2.781738 | -2.00424 | 0.003298 |
| 1 | H | 1.231138 | -2.82816 | 0.002701 |
| 1 | H | 1.947266 | 2.438427 | 0.000106 |
| 1 | H | -0.41277 | 2.672344 | -0.00022 |
| 8 | O | -3.60085 | -1.35377 | 0.000148 |
| 6 | C | -4.71392 | -0.4369 | 4.29E-05 |
| 1 | H | -5.60828 | -1.07304 | 9.92E-05 |
| 1 | H | -4.69055 | 0.200911 | 0.897632 |
| 1 | H | -4.69053 | 0.200726 | -0.89768 |

C2-OMe-AD_3H  $E_{tot}$ = -581.561330 a.u.
Gibbs free energy  $E_G$= -581.456966 a.u.

| | | | | |
|---|---|---|---|---|
| 7 | N | 1.9477 | 0.481835 | 0.000191 |
| 6 | C | 1.366897 | 1.662344 | 7.29E-05 |
| 7 | N | 0.014045 | 1.864121 | -0.00019 |
| 6 | C | -0.81 | 0.748831 | -0.00018 |
| 6 | C | -0.23038 | -0.54511 | -7.5E-05 |
| 6 | C | 1.172361 | -0.64724 | -1.5E-05 |
| 7 | N | -1.25511 | -1.47156 | 1.79E-05 |
| 6 | C | -2.34964 | -0.68392 | 0.000209 |
| 7 | N | -2.14749 | 0.689696 | -0.00017 |
| 7 | N | 1.819045 | -1.83699 | -0.00086 |
| 1 | H | 2.828967 | -1.8623 | 0.002943 |
| 1 | H | 1.279739 | -2.692 | 0.002245 |
| 1 | H | -3.35979 | -1.09528 | 0.000293 |
| 1 | H | -0.4007 | 2.789942 | -0.00037 |
| 8 | O | 2.201475 | 2.728761 | 0.000215 |
| 6 | C | 1.638062 | 4.050814 | 0.000236 |
| 1 | H | 1.034304 | 4.225987 | 0.908501 |
| 1 | H | 2.49843 | 4.730291 | 0.000473 |
| 1 | H | 1.034679 | 4.226169 | -0.90824 |

C8-OH-AD_3H  $E_{tot}$ = -542.293445 a.u.
Gibbs free energy  $E_G$= -542.213071 a.u.

| | | | | |
|---|---|---|---|---|
| 7 | N | 1.934924 | 0.348774 | -8.3E-05 |
| 6 | C | 1.355988 | 1.53011 | -7.8E-05 |
| 7 | N | 0.001826 | 1.729642 | -0.00014 |
| 6 | C | -0.82508 | 0.633955 | -0.0001 |
| 6 | C | -0.24513 | -0.67025 | -0.00012 |
| 6 | C | 1.15324 | -0.78329 | -0.00021 |
| 7 | N | -1.27473 | -1.58889 | 6.76E-05 |
| 6 | C | -2.35902 | -0.78148 | 0.000226 |
| 7 | N | -2.16657 | 0.580325 | 5.82E-05 |
| 7 | N | 1.798972 | -1.97935 | -0.0013 |
| 1 | H | 2.808652 | -2.00346 | 0.003208 |
| 1 | H | 1.264406 | -2.83639 | 0.00286 |
| 1 | H | 1.972889 | 2.431247 | 2.09E-05 |
| 1 | H | -0.38786 | 2.668285 | -0.00014 |
| 8 | O | -3.61798 | -1.26582 | 0.000321 |
| 1 | H | -3.53189 | -2.23418 | 0.000267 |

C2-OH-AD_3H  $E_{tot}$ = -542.294453 a.u.
Gibbs free energy  $E_G$= -542.214882 a.u.

| | | | | |
|---|---|---|---|---|
| 7 | N | 1.968854 | 0.453897 | 7.3E-05 |
| 6 | C | 1.385327 | 1.633357 | -1.7E-05 |
| 7 | N | 0.045903 | 1.857692 | -0.00017 |
| 6 | C | -0.7914 | 0.753656 | -0.00014 |
| 6 | C | -0.21904 | -0.54857 | -6E-05 |
| 6 | C | 1.17952 | -0.67293 | -7.1E-05 |
| 7 | N | -1.24993 | -1.46756 | 0.000195 |
| 6 | C | -2.33808 | -0.67222 | -1.9E-06 |
| 7 | N | -2.12743 | 0.700435 | 1.67E-06 |
| 7 | N | 1.810886 | -1.87047 | -0.00092 |
| 1 | H | 2.819879 | -1.91484 | 0.003085 |
| 1 | H | 1.257632 | -2.71676 | 0.002307 |
| 1 | H | -3.35077 | -1.0771 | 0.000163 |
| 1 | H | -0.31788 | 2.806515 | -0.00026 |
| 8 | O | 2.131843 | 2.763371 | 0.000128 |
| 1 | H | 3.059969 | 2.470606 | 0.000138 |

C8-OH-AD_3H  $E_{tot}$ = -542.293128 a.u.
Gibbs free energy  $E_G$= -542.212789 a.u.

| | | | | |
|---|---|---|---|---|
| 7 | N | 1.922788 | 0.354763 | 4.54E-06 |
| 6 | C | 1.340122 | 1.534107 | -2.4E-05 |
| 7 | N | -0.01548 | 1.729401 | -0.00017 |
| 6 | C | -0.83797 | 0.630419 | -0.00017 |
| 6 | C | -0.25466 | -0.67159 | -0.00014 |
| 6 | C | 1.144958 | -0.77974 | -0.00017 |
| 7 | N | -1.27096 | -1.60349 | 3.63E-05 |
| 6 | C | -2.35872 | -0.81255 | 0.000115 |
| 7 | N | -2.17942 | 0.55955 | -6E-05 |
| 7 | N | 1.789539 | -1.97505 | -0.00119 |
| 1 | H | 2.799118 | -2.00139 | 0.003203 |
| 1 | H | 1.251452 | -2.83045 | 0.00263 |
| 1 | H | 1.953747 | 2.437498 | 0.000113 |
| 1 | H | -0.40569 | 2.66766 | -0.0002 |
| 8 | O | -3.60183 | -1.33767 | 0.000226 |
| 1 | H | -4.21458 | -0.58336 | 0.000178 |

C2-OH-AD_3H  $E_{tot}$ = -542.283675 a.u.
Gibbs free energy  $E_G$= -542.204802 a.u.

| | | | | |
|---|---|---|---|---|
| 7 | N | 1.964607 | 0.463798 | 0.00027 |
| 6 | C | 1.39449 | 1.644117 | 0.000111 |
| 7 | N | 0.04325 | 1.86271 | -0.00025 |
| 6 | C | -0.79402 | 0.756556 | -0.00023 |
| 6 | C | -0.22381 | -0.54337 | -5E-05 |
| 6 | C | 1.176196 | -0.66004 | 4.97E-05 |
| 7 | N | -1.25686 | -1.46086 | -5.6E-05 |
| 6 | C | -2.34413 | -0.66477 | 0.000391 |
| 7 | N | -2.13049 | 0.708216 | -0.00035 |
| 7 | N | 1.813559 | -1.85371 | -0.00075 |
| 1 | H | 2.823354 | -1.88665 | 0.002853 |
| 1 | H | 1.267863 | -2.70481 | 0.002127 |
| 1 | H | -3.35773 | -1.0674 | 0.000406 |
| 1 | H | -0.36148 | 2.795377 | -0.00056 |
| 8 | O | 2.22568 | 2.719664 | 0.000211 |
| 1 | H | 1.719766 | 3.546861 | 0.000327 |

C8-$NH_2$-AD_3H  $E_{tot}$ = -522.433418 a.u.
Gibbs free energy  $E_G$= -522.342048 a.u.

| | | | | |
|---|---|---|---|---|
| 7 | N | 1.93107 | 0.352474 | -0.00023 |
| 6 | C | 1.353137 | 1.533539 | -0.00011 |
| 7 | N | -0.00483 | 1.729538 | -0.00011 |
| 6 | C | -0.83102 | 0.633961 | -8.8E-05 |
| 6 | C | -0.24918 | -0.67086 | -0.00021 |
| 6 | C | 1.149167 | -0.77941 | -0.0004 |
| 7 | N | -1.26969 | -1.59557 | 2.96E-06 |
| 6 | C | -2.37073 | -0.79957 | 0.000326 |
| 7 | N | -2.17035 | 0.5758 | 0.000117 |
| 7 | N | 1.794946 | -1.97929 | -0.00172 |
| 1 | H | 2.804145 | -2.00587 | 0.003595 |
| 1 | H | 1.256935 | -2.83394 | 0.003155 |
| 1 | H | 1.96726 | 2.436377 | 1.59E-05 |
| 1 | H | -0.39467 | 2.667754 | -4E-05 |
| 7 | N | -3.636 | -1.30589 | 0.000474 |
| 1 | H | -3.77374 | -2.30456 | 7.16E-05 |
| 1 | H | -4.42638 | -0.68041 | 0.000128 |

C2-$NH_2$-AD_3H  $E_{tot}$ = -522.430863 a.u.
Gibbs free energy  $E_G$= -522.340542 a.u.

| | | | | |
|---|---|---|---|---|
| 7 | N | 1.966316 | 0.465645 | 0.000243 |
| 6 | C | 1.390457 | 1.661822 | 0.000166 |
| 7 | N | 0.033981 | 1.862307 | -0.00028 |
| 6 | C | -0.79571 | 0.75248 | -0.00029 |
| 6 | C | -0.22137 | -0.54341 | -0.00019 |
| 6 | C | 1.179641 | -0.65437 | -0.0001 |
| 7 | N | -1.25092 | -1.468 | -0.0003 |
| 6 | C | -2.34115 | -0.67718 | 0.000657 |
| 7 | N | -2.13319 | 0.697315 | -0.0005 |
| 7 | N | 1.812928 | -1.85314 | -0.00106 |
| 1 | H | 2.822017 | -1.89293 | 0.003175 |
| 1 | H | 1.261686 | -2.70044 | 0.002213 |
| 1 | H | -3.35319 | -1.08401 | 0.000661 |
| 1 | H | -0.37087 | 2.793687 | -0.00058 |
| 7 | N | 2.196452 | 2.769199 | 0.000185 |
| 1 | H | 3.194702 | 2.622362 | 0.000326 |
| 1 | H | 1.833103 | 3.709533 | 0.000344 |

C8-$NO_2$-AD_1H  $E_{tot}$ = -671.500835 a.u.
Gibbs free energy  $E_G$= -671.428109 a.u.

| | | | | |
|---|---|---|---|---|
| 7 | N | 1.834676 | -0.48512 | 0.014291 |
| 6 | C | 1.226675 | -1.74285 | 0.03459 |
| 7 | N | -0.06008 | -1.94874 | 0.036463 |
| 6 | C | -0.82134 | -0.80815 | 0.014241 |
| 6 | C | -0.2636 | 0.524884 | -0.00218 |
| 6 | C | 1.121792 | 0.69155 | -0.00311 |
| 7 | N | -1.26678 | 1.45127 | 0.005142 |
| 6 | C | -2.34183 | 0.637259 | 0.014035 |
| 7 | N | -2.16766 | -0.71415 | 0.019853 |
| 7 | N | 1.760681 | 1.890938 | -0.06918 |
| 1 | H | 2.716643 | 1.987454 | 0.250711 |
| 1 | H | 1.162226 | 2.703177 | 0.043519 |
| 1 | H | 1.928838 | -2.57969 | 0.050451 |
| 1 | H | 2.849173 | -0.4517 | -0.01114 |
| 7 | N | -3.70469 | 1.215275 | 0.014327 |
| 8 | O | -4.6306 | 0.466033 | -0.29823 |
| 8 | O | -3.80801 | 2.404486 | 0.325092 |

C2-$NO_2$-AD_1H  $E_{tot}$ = -671.504987 a.u.
Gibbs free energy  $E_G$= -671.430871 a.u.

| | | | | |
|---|---|---|---|---|
| 7 | N | 1.821619 | -0.51578 | 0.020951 |
| 6 | C | 1.181953 | -1.74067 | 0.006213 |
| 7 | N | -0.10129 | -1.9492 | -0.00893 |
| 6 | C | -0.84489 | -0.80942 | -0.01002 |
| 6 | C | -0.26955 | 0.522251 | 0.00551 |
| 6 | C | 1.123324 | 0.664806 | 0.021758 |
| 7 | N | -1.26139 | 1.450247 | 0.012589 |
| 6 | C | -2.37767 | 0.656213 | -0.00137 |
| 7 | N | -2.19569 | -0.69466 | -0.01409 |
| 7 | N | 1.779484 | 1.851849 | -0.00352 |
| 1 | H | 2.75937 | 1.920913 | 0.238398 |
| 1 | H | 1.205111 | 2.67876 | 0.114596 |
| 1 | H | -3.37713 | 1.093473 | -0.00084 |
| 1 | H | 2.841691 | -0.57048 | 0.020559 |
| 7 | N | 2.133114 | -2.87444 | 0.011338 |
| 8 | O | 3.345284 | -2.56463 | 0.026709 |
| 8 | O | 1.686322 | -4.01182 | -2.7E-05 |

C8-CN-AD_1H  $E_{tot}$ = -559.262127 a.u.
Gibbs free energy  $E_G$= -559.1899 a.u.

| | | | | |
|---|---|---|---|---|
| 7 | N | 1.829419 | -0.48385 | 0.011031 |
| 6 | C | 1.22123 | -1.74045 | 0.003563 |
| 7 | N | -0.06624 | -1.94398 | -0.00705 |
| 6 | C | -0.82771 | -0.80274 | -0.01096 |
| 6 | C | -0.26925 | 0.530265 | -0.00238 |
| 6 | C | 1.11567 | 0.692885 | 0.01007 |
| 7 | N | -1.26982 | 1.458168 | 0.008929 |
| 6 | C | -2.37015 | 0.652475 | 0.004175 |
| 7 | N | -2.17242 | -0.71135 | -0.00558 |
| 7 | N | 1.760668 | 1.893482 | -0.03735 |
| 1 | H | 2.701108 | 1.981222 | 0.329511 |
| 1 | H | 1.158086 | 2.7005 | 0.092031 |
| 1 | H | 1.921902 | -2.57869 | 0.008805 |
| 1 | H | 2.843818 | -0.44913 | -0.01383 |
| 6 | C | -3.69164 | 1.208938 | 0.012448 |
| 7 | N | -4.76872 | 1.671109 | 0.020038 |

C2-CN-AD_1H  $E_{tot}$ = -559.256303 a.u.
Gibbs free energy  $E_G$= -559.184415 a.u.

| | | | | |
|---|---|---|---|---|
| 7 | N | 1.849765 | -0.49622 | 0.011055 |
| 6 | C | 1.235683 | -1.75808 | 0.005503 |
| 7 | N | -0.06336 | -1.95463 | -0.00473 |
| 6 | C | -0.81373 | -0.81841 | -0.0104 |
| 6 | C | -0.25205 | 0.517852 | -0.00153 |
| 6 | C | 1.133536 | 0.6764 | 0.010278 |
| 7 | N | -1.25526 | 1.439388 | 0.008855 |
| 6 | C | -2.35904 | 0.636588 | 0.002411 |
| 7 | N | -2.16343 | -0.7166 | -0.00702 |
| 7 | N | 1.781229 | 1.875701 | -0.037 |
| 1 | H | 2.724546 | 1.960257 | 0.323168 |
| 1 | H | 1.179766 | 2.682331 | 0.098183 |
| 1 | H | -3.36388 | 1.061586 | 0.006653 |
| 1 | H | 2.865026 | -0.46861 | -0.01392 |
| 6 | C | 2.154187 | -2.85981 | 0.015832 |
| 7 | N | 2.956517 | -3.71584 | 0.024327 |

C8-CHO-AD_1H $E_{tot}$ = -580.344124 a.u.
Gibbs free energy $E_G$=-580.262066 a.u.

| | | | | |
|---|---|---|---|---|
| 7 | N | 1.824815 | -0.39242 | 0.012742 |
| 6 | C | 1.198066 | -1.64202 | 0.005143 |
| 7 | N | -0.09178 | -1.828 | -0.00606 |
| 6 | C | -0.83975 | -0.67609 | -0.01016 |
| 6 | C | -0.26146 | 0.650834 | -0.00121 |
| 6 | C | 1.127695 | 0.793115 | 0.011211 |
| 7 | N | -1.2425 | 1.593035 | 0.010535 |
| 6 | C | -2.36301 | 0.815045 | 0.00478 |
| 7 | N | -2.17964 | -0.55633 | -0.00601 |
| 7 | N | 1.783482 | 1.986351 | -0.03396 |
| 1 | H | 2.729685 | 2.06718 | 0.318577 |
| 1 | H | 1.186897 | 2.798929 | 0.090445 |
| 1 | H | 1.888241 | -2.48906 | 0.01087 |
| 1 | H | 2.839621 | -0.37267 | -0.01034 |
| 6 | C | -3.74391 | 1.356184 | 0.013206 |
| 8 | O | -4.03306 | 2.543727 | 0.028817 |
| 1 | H | -4.52521 | 0.555447 | 0.004283 |

C2-CHO-AD_1H $E_{tot}$ = -580.355145 a.u.
Gibbs free energy $E_G$= -580.272473 a.u.

| | | | | |
|---|---|---|---|---|
| 7 | N | 1.846864 | -0.48795 | 0.020428 |
| 6 | C | 1.246716 | -1.74433 | 0.007174 |
| 7 | N | -0.05792 | -1.95168 | -0.00805 |
| 6 | C | -0.81064 | -0.82289 | -0.00948 |
| 6 | C | -0.25383 | 0.515971 | 0.00455 |
| 6 | C | 1.138827 | 0.679091 | 0.019773 |
| 7 | N | -1.25539 | 1.432922 | 0.0118 |
| 6 | C | -2.3637 | 0.625782 | -0.00021 |
| 7 | N | -2.16668 | -0.72087 | -0.01218 |
| 7 | N | 1.770431 | 1.88125 | -0.00925 |
| 1 | H | 2.745377 | 1.968311 | 0.246787 |
| 1 | H | 1.178082 | 2.694477 | 0.115466 |
| 1 | H | -3.36767 | 1.05299 | 0.000999 |
| 1 | H | 2.868505 | -0.51373 | 0.01939 |
| 6 | C | 2.196654 | -2.86874 | 0.012616 |
| 8 | O | 3.416484 | -2.69823 | 0.028107 |
| 1 | H | 1.721036 | -3.87412 | 0.001342 |

C8-CHO-AD_1H $E_{tot}$ = -580.343273 a.u.
Gibbs free energy $E_G$= -580.261129 a.u.

| | | | | |
|---|---|---|---|---|
| 7 | N | 1.835315 | -0.47635 | 0.009655 |
| 6 | C | 1.240486 | -1.74087 | 0.003623 |
| 7 | N | -0.04334 | -1.96048 | -0.00553 |
| 6 | C | -0.82271 | -0.82845 | -0.00965 |
| 6 | C | -0.27592 | 0.513957 | -0.00282 |
| 6 | C | 1.106961 | 0.691347 | 0.008456 |
| 7 | N | -1.28957 | 1.4235 | 0.009079 |
| 6 | C | -2.38884 | 0.607748 | 0.005607 |
| 7 | N | -2.16397 | -0.7525 | -0.00364 |
| 7 | N | 1.741318 | 1.89888 | -0.04027 |
| 1 | H | 2.678072 | 1.995117 | 0.334269 |
| 1 | H | 1.129326 | 2.69823 | 0.092136 |
| 1 | H | 1.952152 | -2.57013 | 0.008581 |
| 1 | H | 2.849093 | -0.42994 | -0.01768 |
| 6 | C | -3.73298 | 1.233221 | 0.013937 |
| 8 | O | -4.79521 | 0.630856 | 0.014141 |
| 1 | H | -3.68517 | 2.352495 | 0.020193 |

C2-CHO-AD_1H $E_{tot}$ = -580.339473 a.u.
Gibbs free energy $E_G$= -580.257639 a.u.

| | | | | |
|---|---|---|---|---|
| 7 | N | 1.786291 | -0.60532 | 0.009309 |
| 6 | C | 1.173971 | -1.86923 | 0.00744 |
| 7 | N | -0.13168 | -2.04462 | -0.00064 |
| 6 | C | -0.869 | -0.90781 | -0.0081 |
| 6 | C | -0.29868 | 0.428191 | -0.00336 |
| 6 | C | 1.090425 | 0.572917 | 0.007434 |
| 7 | N | -1.2907 | 1.35622 | 0.006638 |
| 6 | C | -2.40527 | 0.559427 | 0.003585 |
| 7 | N | -2.22238 | -0.79066 | -0.00291 |
| 7 | N | 1.74711 | 1.769864 | -0.04379 |
| 1 | H | 2.683871 | 1.847603 | 0.33529 |
| 1 | H | 1.148865 | 2.578428 | 0.095434 |
| 1 | H | -3.40546 | 0.99566 | 0.008416 |
| 1 | H | 2.803263 | -0.58343 | -0.01487 |
| 6 | C | 2.135773 | -3.00104 | 0.017516 |
| 8 | O | 1.838329 | -4.1804 | 0.014989 |
| 1 | H | 3.216316 | -2.67472 | 0.02734 |

C8-Cl-AD_1H $E_{tot}$ = -926.705777 a.u.
Gibbs free energy $E_G$= -926.640506 a.u.

| | | | | |
|---|---|---|---|---|
| 7 | N | 1.837789 | -0.4892 | 0.000646 |
| 6 | C | 1.232278 | -1.74099 | 0.00534 |
| 7 | N | -0.05883 | -1.9433 | 0.001961 |
| 6 | C | -0.81674 | -0.80341 | -0.00794 |
| 6 | C | -0.25652 | 0.531318 | -0.00855 |
| 6 | C | 1.122496 | 0.690442 | -0.00155 |
| 7 | N | -1.26753 | 1.459108 | 0.003135 |
| 6 | C | -2.3463 | 0.647837 | 0.006855 |
| 7 | N | -2.16447 | -0.70921 | 0.0036 |
| 7 | N | 1.787438 | 1.891284 | -0.06952 |
| 1 | H | 2.67337 | 1.975032 | 0.41947 |
| 1 | H | 1.171782 | 2.690103 | 0.058749 |
| 1 | H | 1.929619 | -2.58196 | 0.013618 |
| 1 | H | 2.851252 | -0.4507 | -0.0433 |
| 17 | Cl | -3.94584 | 1.308399 | 0.019091 |

C2-Cl-AD_1H $E_{tot}$ = -926.702403 a.u.
Gibbs free energy $E_G$= -926.637384 a.u.

| | | | | |
|---|---|---|---|---|
| 7 | N | 1.839498 | -0.50067 | 0.003836 |
| 6 | C | 1.214501 | -1.73975 | 0.00555 |
| 7 | N | -0.06 | -1.9451 | 0.001651 |
| 6 | C | -0.8258 | -0.80317 | -0.00911 |
| 6 | C | -0.26069 | 0.529465 | -0.00784 |
| 6 | C | 1.117217 | 0.680296 | 0.000973 |
| 7 | N | -1.2735 | 1.45576 | 0.004548 |
| 6 | C | -2.36612 | 0.656237 | 0.006024 |
| 7 | N | -2.16716 | -0.70805 | 0.001465 |
| 7 | N | 1.790564 | 1.872721 | -0.06103 |
| 1 | H | 2.693949 | 1.945781 | 0.395416 |
| 1 | H | 1.185714 | 2.678605 | 0.071615 |
| 1 | H | -3.37473 | 1.072794 | 0.013251 |
| 1 | H | 2.852985 | -0.47948 | -0.04213 |
| 17 | Cl | 2.34794 | -3.07591 | 0.019723 |

C8-F-AD_1H $E_{tot}$ = -566.280394 a.u.
Gibbs free energy $E_G$= -566.212364 a.u

| | | | | |
|---|---|---|---|---|
| 7 | N | 1.836326 | -0.48973 | -0.002 |
| 6 | C | 1.232704 | -1.73978 | 0.005166 |
| 7 | N | -0.05975 | -1.94192 | 0.004103 |
| 6 | C | -0.81691 | -0.80403 | -0.00625 |
| 6 | C | -0.25667 | 0.533276 | -0.0084 |
| 6 | C | 1.120624 | 0.690954 | -0.00312 |
| 7 | N | -1.27105 | 1.460369 | 0.003607 |
| 6 | C | -2.33347 | 0.640819 | 0.008733 |
| 7 | N | -2.16573 | -0.70979 | 0.00666 |
| 7 | N | 1.791527 | 1.891161 | -0.07474 |
| 1 | H | 2.664229 | 1.974009 | 0.438479 |
| 1 | H | 1.174778 | 2.689995 | 0.049438 |
| 1 | H | 1.928802 | -2.58173 | 0.013133 |
| 1 | H | 2.849503 | -0.44964 | -0.05048 |
| 9 | F | -3.56853 | 1.148494 | 0.018907 |

C2-F-AD_1H $E_{tot}$ = -566.275710 a.u.
Gibbs free energy $E_G$= -566.208069 a.u

| | | | | |
|---|---|---|---|---|
| 7 | N | 1.843815 | -0.50091 | 0.004092 |
| 6 | C | 1.207702 | -1.72522 | 0.005575 |
| 7 | N | -0.05601 | -1.9514 | 0.002792 |
| 6 | C | -0.82559 | -0.80647 | -0.00858 |
| 6 | C | -0.25775 | 0.52692 | -0.00788 |
| 6 | C | 1.117268 | 0.682431 | 0.000439 |
| 7 | N | -1.27365 | 1.45435 | 0.004599 |
| 6 | C | -2.36311 | 0.65644 | 0.007624 |
| 7 | N | -2.16333 | -0.71148 | 0.003321 |
| 7 | N | 1.793902 | 1.874269 | -0.06641 |
| 1 | H | 2.681683 | 1.951429 | 0.420228 |
| 1 | H | 1.184008 | 2.678536 | 0.055461 |
| 1 | H | -3.37261 | 1.070858 | 0.015243 |
| 1 | H | 2.857219 | -0.48724 | -0.05111 |
| 9 | F | 2.09651 | -2.74372 | 0.015823 |

C8-H-AD_1H $E_{tot}$ = -467.069792 a.u.
Gibbs free energy $E_G$= -466.99272 a.u.

| | | | | |
|---|---|---|---|---|
| 7 | N | 1.843838 | -0.48974 | -0.00037 |
| 6 | C | 1.238324 | -1.74401 | 0.005322 |
| 7 | N | -0.0518 | -1.94645 | 0.002588 |
| 6 | C | -0.8139 | -0.80699 | -0.00787 |
| 6 | C | -0.25316 | 0.529442 | -0.00976 |
| 6 | C | 1.1257 | 0.687191 | -0.00332 |
| 7 | N | -1.26468 | 1.454384 | 0.003038 |
| 6 | C | -2.35845 | 0.651982 | 0.008009 |
| 7 | N | -2.15946 | -0.7083 | 0.0051 |
| 7 | N | 1.789146 | 1.88985 | -0.07085 |
| 1 | H | 2.673125 | 1.975185 | 0.421389 |
| 1 | H | 1.168191 | 2.684323 | 0.060485 |
| 1 | H | 1.937806 | -2.58348 | 0.014141 |
| 1 | H | -3.36668 | 1.070055 | 0.017315 |
| 1 | H | 2.857129 | -0.45 | -0.04418 |

C2-H-AD_1H $E_{tot}$ = -467.069792 a.u.
Gibbs free energy $E_G$= -466.99272 a.u.

| | | | | |
|---|---|---|---|---|
| 7 | N | 1.843838 | -0.48974 | -0.00037 |
| 6 | C | 1.238324 | -1.74401 | 0.005322 |
| 7 | N | -0.0518 | -1.94645 | 0.002588 |
| 6 | C | -0.8139 | -0.80699 | -0.00787 |
| 6 | C | -0.25316 | 0.529442 | -0.00976 |
| 6 | C | 1.1257 | 0.687191 | -0.00332 |
| 7 | N | -1.26468 | 1.454384 | 0.003038 |
| 6 | C | -2.35845 | 0.651982 | 0.008009 |
| 7 | N | -2.15946 | -0.7083 | 0.0051 |
| 7 | N | 1.789146 | 1.88985 | -0.07085 |
| 1 | H | 2.673125 | 1.975185 | 0.421389 |
| 1 | H | 1.168191 | 2.684323 | 0.060485 |
| 1 | H | 1.937806 | -2.58348 | 0.014141 |
| 1 | H | -3.36668 | 1.070055 | 0.017315 |
| 1 | H | 2.857129 | -0.45 | -0.04418 |

C8-Me-AD_1H    $E_{tot}$ = -506.371454 a.u.
Gibbs free energy    $E_G$=-506.270763 a.u.

| | | | | |
|---|---|---|---|---|
| 7 | N | 1.843535 | -0.48706 | -0.01734 |
| 6 | C | 1.237493 | -1.73316 | -0.13095 |
| 7 | N | -0.05357 | -1.93059 | -0.18135 |
| 6 | C | -0.81431 | -0.79191 | -0.11493 |
| 6 | C | -0.25087 | 0.539179 | 0.009985 |
| 6 | C | 1.125494 | 0.689609 | 0.062473 |
| 7 | N | -1.26358 | 1.459842 | 0.075298 |
| 6 | C | -2.36879 | 0.671633 | -0.01406 |
| 7 | N | -2.15562 | -0.69071 | -0.12643 |
| 7 | N | 1.801366 | 1.891636 | 0.108384 |
| 1 | H | 2.644818 | 1.92835 | 0.674259 |
| 1 | H | 1.173207 | 2.672783 | 0.282048 |
| 1 | H | 1.934043 | -2.57391 | -0.1763 |
| 1 | H | 2.857259 | -0.44432 | -0.04181 |
| 6 | C | -3.7523 | 1.235966 | 0.00874 |
| 1 | H | -3.71507 | 2.328406 | 0.114507 |
| 1 | H | -4.2866 | 0.973632 | -0.91974 |
| 1 | H | -4.32803 | 0.802781 | 0.843526 |

C2-Me-AD_1H    $E_{tot}$ = -506.370739 a.u.
Gibbs free energy    $E_G$=-506.269624 a.u.

| | | | | |
|---|---|---|---|---|
| 7 | N | 1.824737 | -0.51139 | -0.00187 |
| 6 | C | 1.223773 | -1.77437 | 0.005661 |
| 7 | N | -0.07544 | -1.94579 | 0.004994 |
| 6 | C | -0.82817 | -0.80073 | -0.00605 |
| 6 | C | -0.26165 | 0.530891 | -0.01079 |
| 6 | C | 1.116069 | 0.67202 | -0.0061 |
| 7 | N | -1.26968 | 1.463531 | 0.002705 |
| 6 | C | -2.36589 | 0.667497 | 0.010598 |
| 7 | N | -2.17315 | -0.69559 | 0.009362 |
| 7 | N | 1.796838 | 1.866368 | -0.07663 |
| 1 | H | 2.675315 | 1.940506 | 0.427642 |
| 1 | H | 1.18493 | 2.668176 | 0.05293 |
| 1 | H | -3.3725 | 1.089643 | 0.0204 |
| 1 | H | 2.837556 | -0.47029 | -0.05306 |
| 6 | C | 2.167402 | -2.94443 | 0.017053 |
| 1 | H | 2.823631 | -2.94107 | -0.87095 |
| 1 | H | 2.810303 | -2.9338 | 0.914613 |
| 1 | H | 1.574576 | -3.86642 | 0.015819 |

C8-OMe-AD_1H    $E_{tot}$ = -581.554600 a.u.
Gibbs free energy    $E_G$= -581.44955 a.u

| | | | | |
|---|---|---|---|---|
| 7 | N | 1.805936 | -0.49501 | -0.00723 |
| 6 | C | 1.200013 | -1.7403 | 0.00435 |
| 7 | N | -0.09658 | -1.93558 | 0.004287 |
| 6 | C | -0.8491 | -0.79565 | -0.01086 |
| 6 | C | -0.28477 | 0.542683 | -0.01496 |
| 6 | C | 1.093523 | 0.688977 | -0.00816 |
| 7 | N | -1.28876 | 1.472047 | -0.00634 |
| 6 | C | -2.3805 | 0.66851 | -0.00033 |
| 7 | N | -2.19397 | -0.69607 | 0.00185 |
| 7 | N | 1.780262 | 1.888841 | -0.08445 |
| 1 | H | 2.600206 | 1.977537 | 0.511351 |
| 1 | H | 1.14969 | 2.681686 | 0.015694 |
| 1 | H | 1.890976 | -2.58629 | 0.015144 |
| 1 | H | 2.818569 | -0.45538 | -0.06411 |
| 8 | O | -3.59844 | 1.227965 | 0.00746 |
| 6 | C | -4.71593 | 0.310136 | 0.00971 |
| 1 | H | -5.60779 | 0.949931 | 0.017016 |
| 1 | H | -4.69561 | -0.32326 | -0.89025 |
| 1 | H | -4.68581 | -0.3309 | 0.903948 |

C2-OMe-AD_1H    $E_{tot}$ = -581.555696 a.u.
Gibbs free energy    $E_G$= -581.450664 a.u

| | | | | |
|---|---|---|---|---|
| 7 | N | 1.827005 | -0.54669 | 0.006125 |
| 6 | C | 1.194045 | -1.78279 | 0.014064 |
| 7 | N | -0.0935 | -1.97076 | 0.011439 |
| 6 | C | -0.84244 | -0.81663 | -0.00369 |
| 6 | C | -0.26029 | 0.508381 | -0.01021 |
| 6 | C | 1.115771 | 0.640904 | -0.00377 |
| 7 | N | -1.26624 | 1.45175 | 0.000163 |
| 6 | C | -2.36402 | 0.667733 | 0.007319 |
| 7 | N | -2.18145 | -0.7034 | 0.008653 |
| 7 | N | 1.809574 | 1.826449 | -0.07662 |
| 1 | H | 2.688583 | 1.8912 | 0.428086 |
| 1 | H | 1.206457 | 2.635328 | 0.049037 |
| 1 | H | -3.36865 | 1.094528 | 0.014367 |
| 1 | H | 2.84021 | -0.5412 | -0.05129 |
| 8 | O | 2.099784 | -2.78712 | 0.025872 |
| 6 | C | 1.535234 | -4.12448 | 0.023657 |
| 1 | H | 0.923589 | -4.27431 | -0.87761 |
| 1 | H | 2.404964 | -4.792 | 0.030459 |
| 1 | H | 0.911183 | -4.272 | 0.916699 |

C8-OMe-AD_1H    $E_{tot}$ = -581.554279 a.u.

Gibbs free energy    $E_G$= -581.449095 a.u

| | | | | |
|---|---|---|---|---|
| 7 | N | 1.856561 | -0.52292 | -0.013 |
| 6 | C | 1.224597 | -1.75528 | 0.000212 |
| 7 | N | -0.07334 | -1.92728 | 0.008899 |
| 6 | C | -0.80785 | -0.77195 | 0.000444 |
| 6 | C | -0.2127 | 0.554824 | -0.00486 |
| 6 | C | 1.164726 | 0.67564 | -0.00758 |
| 7 | N | -1.20621 | 1.501581 | 0.010464 |
| 6 | C | -2.31043 | 0.713514 | 0.020164 |
| 7 | N | -2.14689 | -0.65241 | 0.019746 |
| 7 | N | 1.881458 | 1.860193 | -0.08775 |
| 1 | H | 2.697438 | 1.928038 | 0.516883 |
| 1 | H | 1.269641 | 2.666714 | 0.016954 |
| 1 | H | 1.899327 | -2.61486 | 0.004259 |
| 1 | H | 2.868715 | -0.50243 | -0.08403 |
| 8 | O | -3.56009 | 1.196844 | 0.032855 |
| 6 | C | -3.67351 | 2.635815 | 0.032144 |
| 1 | H | -4.75207 | 2.837895 | 0.039169 |
| 1 | H | -3.19019 | 3.059828 | 0.926354 |
| 1 | H | -3.20184 | 3.057961 | -0.86902 |

C2-OMe-AD_1H    $E_{tot}$ = -581.542138 a.u.

Gibbs free energy    $E_G$= -581.437865 a.u

| | | | | |
|---|---|---|---|---|
| 7 | N | 1.848803 | -0.41708 | -0.00839 |
| 6 | C | 1.266695 | -1.68131 | -0.00435 |
| 7 | N | -0.01535 | -1.90644 | 0.000716 |
| 6 | C | -0.80504 | -0.78786 | -0.00762 |
| 6 | C | -0.27749 | 0.564845 | -0.00651 |
| 6 | C | 1.08821 | 0.745701 | -0.00598 |
| 7 | N | -1.31978 | 1.465913 | 0.013787 |
| 6 | C | -2.38437 | 0.635366 | 0.018763 |
| 7 | N | -2.14851 | -0.72594 | 0.010994 |
| 7 | N | 1.753768 | 1.954318 | -0.08345 |
| 1 | H | 2.575874 | 2.059894 | 0.506518 |
| 1 | H | 1.109904 | 2.737114 | 0.009669 |
| 1 | H | -3.40536 | 1.02172 | 0.031537 |
| 8 | H | 2.852576 | -0.32365 | -0.1112 |
| 6 | O | 2.089585 | -2.76382 | 0.000253 |
| 6 | C | 3.510152 | -2.57507 | 0.017092 |
| 1 | H | 3.859701 | -2.05711 | -0.89569 |
| 1 | H | 3.833337 | -2.02977 | 0.922623 |
| 1 | H | 3.936343 | -3.58511 | 0.035804 |

C8-OH-AD_1H    $E_{tot}$ = -542.280433 a.u.

Gibbs free energy    $E_G$= -542.199927 a.u.

| | | | | |
|---|---|---|---|---|
| 7 | N | 1.816125 | -0.49437 | -0.00733 |
| 6 | C | 1.205483 | -1.73724 | 0.005059 |
| 7 | N | -0.09085 | -1.92938 | 0.007204 |
| 6 | C | -0.84026 | -0.78664 | -0.006 |
| 6 | C | -0.27037 | 0.549593 | -0.01115 |
| 6 | C | 1.106954 | 0.692628 | -0.00782 |
| 7 | N | -1.27387 | 1.485218 | -0.00044 |
| 6 | C | -2.36075 | 0.687344 | 0.007085 |
| 7 | N | -2.18427 | -0.67845 | 0.008173 |
| 7 | N | 1.79695 | 1.889311 | -0.08721 |
| 1 | H | 2.623856 | 1.974622 | 0.499115 |
| 1 | H | 1.171787 | 2.685993 | 0.014996 |
| 1 | H | 1.893698 | -2.58564 | 0.014322 |
| 1 | H | 2.82875 | -0.45839 | -0.06641 |
| 8 | O | -3.59948 | 1.215709 | 0.016929 |
| 1 | H | -4.2109 | 0.459445 | 0.020811 |

C2-OH-AD_1H    $E_{tot}$ = -542.279325 a.u.

Gibbs free energy    $E_G$= -542.199126 a.u.

| | | | | |
|---|---|---|---|---|
| 7 | N | 1.852626 | -0.516 | 0.004206 |
| 6 | C | 1.234392 | -1.75244 | 0.009176 |
| 7 | N | -0.04849 | -1.96464 | 0.006771 |
| 6 | C | -0.81667 | -0.82282 | -0.00635 |
| 6 | C | -0.24992 | 0.510874 | -0.0092 |
| 6 | C | 1.123218 | 0.663892 | -0.00231 |
| 7 | N | -1.26818 | 1.440162 | 0.002996 |
| 6 | C | -2.35567 | 0.642239 | 0.00777 |
| 7 | N | -2.15581 | -0.72682 | 0.006375 |
| 7 | N | 1.802484 | 1.857459 | -0.07343 |
| 1 | H | 2.677461 | 1.934771 | 0.436679 |
| 1 | H | 1.188289 | 2.658763 | 0.047988 |
| 1 | H | -3.36572 | 1.055864 | 0.015606 |
| 1 | H | 2.865393 | -0.4947 | -0.05611 |
| 8 | O | 2.13575 | -2.76893 | 0.018711 |
| 1 | H | 1.607303 | -3.58637 | 0.015014 |

C8-OH-AD_1H $E_{tot}$ = -542.279278 a.u.
Gibbs free energy $E_G$= -542.198816 a.u.

| | | | | |
|---|---|---|---|---|
| 7 | N | 1.829347 | -0.4886 | -0.01015 |
| 6 | C | 1.223827 | -1.73397 | 0.004035 |
| 7 | N | -0.071 | -1.9315 | 0.009978 |
| 6 | C | -0.82725 | -0.79302 | -0.00182 |
| 6 | C | -0.26123 | 0.54651 | -0.00896 |
| 6 | C | 1.114659 | 0.695331 | -0.00852 |
| 7 | N | -1.27751 | 1.468365 | 0.003324 |
| 6 | C | -2.3616 | 0.653896 | 0.013518 |
| 7 | N | -2.17225 | -0.70186 | 0.015884 |
| 7 | N | 1.805345 | 1.893637 | -0.08878 |
| 1 | H | 2.629087 | 1.977325 | 0.502687 |
| 1 | H | 1.181121 | 2.690102 | 0.017306 |
| 1 | H | 1.915824 | -2.57942 | 0.011197 |
| 1 | H | 2.841529 | -0.44787 | -0.07351 |
| 8 | O | -3.61487 | 1.146275 | 0.02405 |
| 1 | H | -3.52419 | 2.114505 | 0.019462 |

C2-OH-AD_1H $E_{tot}$ = -542.264826 a.u.
Gibbs free energy $E_G$= -542.186423 a.u.

| | | | | |
|---|---|---|---|---|
| 7 | N | 1.860778 | -0.48852 | -0.04538 |
| 6 | C | 1.248802 | -1.74352 | -0.05383 |
| 7 | N | -0.03095 | -1.94791 | -0.0424 |
| 6 | C | -0.8003 | -0.80913 | -0.03557 |
| 6 | C | -0.24588 | 0.530132 | 0.007002 |
| 6 | C | 1.124393 | 0.689312 | -0.0067 |
| 7 | N | -1.26999 | 1.450289 | 0.056571 |
| 6 | C | -2.3513 | 0.642924 | 0.034208 |
| 7 | N | -2.14062 | -0.72276 | -0.01502 |
| 7 | N | 1.804215 | 1.886081 | -0.06911 |
| 1 | H | 2.661728 | 1.961674 | 0.471265 |
| 1 | H | 1.183499 | 2.682383 | 0.055877 |
| 1 | H | -3.36449 | 1.048281 | 0.057408 |
| 1 | H | 2.847942 | -0.42781 | -0.27423 |
| 8 | O | 2.098016 | -2.81278 | -0.12277 |
| 1 | H | 2.899823 | -2.64419 | 0.396937 |

C8-$NH_2$-AD_1H $E_{tot}$ = -522.421093 a.u.
Gibbs free energy $E_G$= -522.329075 a.u.

| | | | | |
|---|---|---|---|---|
| 7 | N | 1.821857 | -0.49234 | -0.02741 |
| 6 | C | 1.21548 | -1.73462 | -0.00935 |
| 7 | N | -0.08137 | -1.9297 | 0.017763 |
| 6 | C | -0.83563 | -0.78924 | 0.024631 |
| 6 | C | -0.26752 | 0.551266 | 0.014924 |
| 6 | C | 1.10838 | 0.694076 | -0.00595 |
| 7 | N | -1.2755 | 1.478128 | 0.042265 |
| 6 | C | -2.37496 | 0.67575 | 0.068735 |
| 7 | N | -2.17676 | -0.69266 | 0.062461 |
| 7 | N | 1.807606 | 1.892835 | -0.09238 |
| 1 | H | 2.598649 | 1.982696 | 0.543099 |
| 1 | H | 1.173336 | 2.683854 | 0.002073 |
| 1 | H | 1.90458 | -2.58243 | -0.01845 |
| 1 | H | 2.83225 | -0.45043 | -0.11261 |
| 7 | N | -3.64262 | 1.187497 | 0.150532 |
| 1 | H | -3.75299 | 2.157641 | -0.11676 |
| 1 | H | -4.39191 | 0.556552 | -0.10547 |

C2-$NH_2$-AD_1H $E_{tot}$ = -522.414063 a.u.
Gibbs free energy $E_G$= -522.322177 a.u.

| | | | | |
|---|---|---|---|---|
| 7 | N | 1.867562 | -0.50066 | -0.05628 |
| 6 | C | 1.268699 | -1.7602 | -0.05508 |
| 7 | N | -0.0244 | -1.96193 | -0.03304 |
| 6 | C | -0.79338 | -0.82558 | -0.02672 |
| 6 | C | -0.23962 | 0.513722 | 0.007689 |
| 6 | C | 1.130796 | 0.672629 | -0.01391 |
| 7 | N | -1.26452 | 1.434731 | 0.062252 |
| 6 | C | -2.34543 | 0.627138 | 0.050375 |
| 7 | N | -2.1353 | -0.73858 | 0.003588 |
| 7 | N | 1.807358 | 1.872476 | -0.07883 |
| 1 | H | 2.66835 | 1.946645 | 0.456202 |
| 1 | H | 1.185269 | 2.665748 | 0.057663 |
| 1 | H | -3.35857 | 1.032572 | 0.080142 |
| 1 | H | 2.857444 | -0.44475 | -0.2724 |
| 7 | N | 2.164839 | -2.82777 | -0.15604 |
| 1 | H | 2.936185 | -2.80332 | 0.508435 |
| 1 | H | 1.660408 | -3.70925 | -0.09495 |

**Table S14.** Values of total energies and Gibbs free energies, cartesian coordinates of equilibrium geometries of NX substituted adenine (AD) 9H, 7H, 3H and 1H tautomers.

$N9-NO_2-AD\_9H$ $E_{tot}$ = -671.492041 a.u.
Gibbs free energy $E_G$= -671.419679 a.u.

| | | | | |
|---|---|---|---|---|
| 7 | N | 3.075438 | -0.009967 | 0.000172 |
| 6 | C | 2.785791 | -1.324234 | 0.000101 |
| 7 | N | 1.576966 | -1.929022 | 0.000073 |
| 6 | C | 0.587013 | -1.031651 | 0.000010 |
| 6 | C | 0.720821 | 0.369295 | -0.000006 |
| 6 | C | 2.045854 | 0.870583 | 0.000057 |
| 7 | N | -0.516285 | 1.020554 | -0.000059 |
| 6 | C | -1.411119 | 0.061619 | 0.000068 |
| 7 | N | -0.814654 | -1.212695 | 0.000005 |
| 7 | N | 2.320976 | 2.198131 | -0.000386 |
| 1 | H | 3.285411 | 2.500587 | 0.001177 |
| 1 | H | 1.571673 | 2.875653 | 0.001082 |
| 1 | H | 3.648551 | -1.997558 | 0.000365 |
| 1 | H | -2.491476 | 0.165958 | 0.000098 |
| 7 | N | -1.562733 | -2.447789 | 0.000013 |
| 8 | O | -2.779166 | -2.307408 | 0.000331 |
| 8 | O | -0.910202 | -3.470692 | 0.000254 |

$N7-NO_2-AD\_7H$ $E_{tot}$ = -671.4901144 a.u.
Gibbs free energy $E_G$= -671.41608 a.u.

| | | | | |
|---|---|---|---|---|
| 7 | N | -3.06426 | -0.0483 | 0.048716 |
| 6 | C | -2.74386 | -1.35259 | 0.087473 |
| 7 | N | -1.52304 | -1.91601 | 0.051302 |
| 6 | C | -0.53534 | -1.01093 | 0.008296 |
| 6 | C | -0.73839 | 0.392584 | 0.013428 |
| 6 | C | -2.07208 | 0.873166 | -0.02083 |
| 7 | N | 0.569442 | 0.91627 | -0.09651 |
| 6 | C | 1.455828 | -0.18047 | -0.1253 |
| 7 | N | 0.833249 | -1.32502 | -0.07293 |
| 7 | N | -2.44421 | 2.17163 | -0.17177 |
| 1 | H | -3.43052 | 2.366275 | -0.05002 |
| 1 | H | -1.76584 | 2.900924 | 0.009197 |
| 1 | H | -3.592 | -2.0425 | 0.146075 |
| 1 | H | 2.528461 | -0.01473 | -0.17337 |
| 7 | N | 1.031958 | 2.246792 | 0.109752 |
| 8 | O | 0.193977 | 3.075621 | 0.457497 |
| 8 | O | 2.22501 | 2.432731 | -0.07979 |

N9-CN-AD_9H $E_{tot}$ = -559.263292 a.u.
Gibbs free energy $E_G$= -559.192009 a.u.

| | | | | |
|---|---|---|---|---|
| 7 | N | 2.701585 | -0.026905 | 0.000157 |
| 6 | C | 2.400440 | -1.339361 | 0.000688 |
| 7 | N | 1.182962 | -1.926024 | 0.000356 |
| 6 | C | 0.212950 | -1.010358 | 0.000267 |
| 6 | C | 0.352256 | 0.386750 | 0.000077 |
| 6 | C | 1.685776 | 0.869443 | -0.000176 |
| 7 | N | -0.886302 | 1.031726 | 0.000551 |
| 6 | C | -1.776775 | 0.071917 | 0.000063 |
| 7 | N | -1.185026 | -1.218516 | 0.000036 |
| 7 | N | 1.982513 | 2.192212 | -0.000805 |
| 1 | H | 2.951901 | 2.478624 | 0.001488 |
| 1 | H | 1.245428 | 2.882884 | 0.001609 |
| 1 | H | 3.256400 | -2.020992 | -0.000068 |
| 1 | H | -2.859268 | 0.176949 | -0.000017 |
| 6 | C | -1.838178 | -2.395507 | -0.000152 |
| 7 | N | -2.440106 | -3.401936 | -0.000275 |

N7-CN-AD_7H $E_{tot}$ = -559.256945 a.u.
Gibbs free energy $E_G$= -559.186408 a.u.

| | | | | |
|---|---|---|---|---|
| 7 | N | -2.69055 | -0.03091 | 0.035411 |
| 6 | C | -2.37146 | -1.34331 | 0.023268 |
| 7 | N | -1.15698 | -1.91725 | -0.00054 |
| 6 | C | -0.15779 | -1.01634 | -0.01179 |
| 6 | C | -0.37287 | 0.376347 | -0.001 |
| 6 | C | -1.69058 | 0.877746 | 0.021718 |
| 7 | N | 0.917929 | 0.950648 | -0.01759 |
| 6 | C | 1.821281 | -0.14327 | -0.03742 |
| 7 | N | 1.216158 | -1.29945 | -0.035 |
| 7 | N | -2.01811 | 2.199233 | 0.017204 |
| 1 | H | -2.99787 | 2.441892 | 0.080521 |
| 1 | H | -1.32089 | 2.928828 | 0.067306 |
| 1 | H | -3.22616 | -2.02723 | 0.034941 |
| 1 | H | 2.893943 | 0.039487 | -0.05223 |
| 6 | C | 1.219311 | 2.257667 | -0.0069 |
| 7 | N | 1.451731 | 3.409719 | 0.004696 |

N9-CHO-AD_9H $E_{tot}$ = -580.374927 a.u.
Gibbs free energy $E_G$= -580.293037 a.u.

| | | | | |
|---|---|---|---|---|
| 7 | N | 2.744507 | -0.235900 | 0.000188 |
| 6 | C | 2.301101 | -1.506963 | -0.000104 |
| 7 | N | 1.026912 | -1.957136 | -0.000144 |
| 6 | C | 0.156390 | -0.939526 | -0.000190 |
| 6 | C | 0.455421 | 0.431478 | -0.000065 |
| 6 | C | 1.831625 | 0.766475 | 0.000142 |
| 7 | N | -0.710496 | 1.206354 | -0.000355 |
| 6 | C | -1.696693 | 0.337886 | 0.000088 |
| 7 | N | -1.250211 | -0.998650 | 0.000031 |
| 7 | N | 2.271668 | 2.049652 | -0.000506 |
| 1 | H | 3.266142 | 2.229982 | 0.001736 |
| 1 | H | 1.612548 | 2.815148 | 0.001639 |
| 1 | H | 3.078234 | -2.277574 | 0.000388 |
| 1 | H | -2.759786 | 0.563381 | 0.000238 |
| 6 | C | -2.040600 | -2.165596 | 0.000212 |
| 8 | O | -3.255316 | -2.143632 | 0.000225 |
| 1 | H | -1.414883 | -3.081083 | 0.000244 |

N7-CHO-AD_7H $E_{tot}$ = -580.371250 a.u.
Gibbs free energy $E_G$= -580.287887 a.u.

| | | | | |
|---|---|---|---|---|
| 7 | N | -2.82581 | 0.188357 | 0.030636 |
| 6 | C | -2.60311 | -1.13892 | 0.012089 |
| 7 | N | -1.43124 | -1.79929 | -0.01165 |
| 6 | C | -0.37481 | -0.96968 | -0.01645 |
| 6 | C | -0.47225 | 0.439213 | 0.001274 |
| 6 | C | -1.76072 | 1.028716 | 0.025865 |
| 7 | N | 0.863568 | 0.902896 | -0.01164 |
| 6 | C | 1.662068 | -0.26481 | -0.03616 |
| 7 | N | 0.974726 | -1.37286 | -0.03962 |
| 7 | N | -1.99969 | 2.360637 | 0.04468 |
| 1 | H | -2.96243 | 2.668359 | 0.061535 |
| 1 | H | -1.2224 | 3.015703 | 0.041572 |
| 1 | H | -3.50418 | -1.76146 | 0.017204 |
| 1 | H | 2.748618 | -0.18635 | -0.05013 |
| 6 | C | 1.394297 | 2.188458 | -0.00404 |
| 8 | O | 0.74628 | 3.224504 | 0.016704 |
| 1 | H | 2.504262 | 2.170943 | -0.01925 |

N9-CHO-AD_9H $E_{tot}$ = -580.367327 a.u.
Gibbs free energy $E_G$= -580.285083 a.u.

| | | | | |
|---|---|---|---|---|
| 7 | N | 2.829687 | -0.290032 | 0.000066 |
| 6 | C | 2.414950 | -1.572474 | 0.000336 |
| 7 | N | 1.155530 | -2.057890 | 0.000194 |
| 6 | C | 0.252752 | -1.069693 | 0.000140 |
| 6 | C | 0.523524 | 0.311043 | -0.000008 |
| 6 | C | 1.890042 | 0.684220 | -0.000166 |
| 7 | N | -0.650901 | 1.071436 | 0.000364 |
| 6 | C | -1.617521 | 0.185400 | 0.000039 |
| 7 | N | -1.158114 | -1.148565 | 0.000076 |
| 7 | N | 2.291016 | 1.981124 | -0.000799 |
| 1 | H | 3.279680 | 2.190620 | 0.001609 |
| 1 | H | 1.609197 | 2.726222 | 0.001652 |
| 1 | H | 3.211422 | -2.323298 | 0.000032 |
| 1 | H | -2.683730 | 0.405767 | 0.000020 |
| 6 | C | -2.014575 | -2.271879 | 0.000005 |
| 8 | O | -1.654413 | -3.425705 | -0.000028 |
| 1 | H | -3.082074 | -1.949620 | 0.000080 |

N7-CHO-AD_7H $E_{tot}$ = -580.362767 a.u.
Gibbs free energy $E_G$= -580.27991 a.u.

| | | | | |
|---|---|---|---|---|
| 7 | N | -2.72643 | 0.156198 | -0.03273 |
| 6 | C | -2.53719 | -1.1793 | -0.04222 |
| 7 | N | -1.37889 | -1.85769 | 0.002623 |
| 6 | C | -0.30371 | -1.05362 | 0.022024 |
| 6 | C | -0.37285 | 0.360776 | -0.00431 |
| 6 | C | -1.64565 | 0.964271 | 0.000403 |
| 7 | N | 0.969323 | 0.795639 | 0.001528 |
| 6 | C | 1.745875 | -0.38133 | 0.043379 |
| 7 | N | 1.033892 | -1.47855 | 0.057239 |
| 7 | N | -1.88156 | 2.326517 | -0.02012 |
| 1 | H | -2.85771 | 2.557447 | 0.143859 |
| 1 | H | -1.23031 | 2.923931 | 0.476716 |
| 1 | H | -3.45082 | -1.78117 | -0.07764 |
| 1 | H | 2.83096 | -0.31192 | 0.049127 |
| 6 | C | 1.495604 | 2.092409 | -0.11808 |
| 8 | O | 2.686155 | 2.336609 | -0.10238 |
| 1 | H | 0.709247 | 2.86498 | -0.24622 |

N9-Cl-AD_9H $E_{tot}$ = -926.679501 a.u.
Gibbs free energy $E_G$= -926.61612 a.u.

| | | | | |
|---|---|---|---|---|
| 7 | N | -2.813263 | -0.031582 | 0.010803 |
| 6 | C | -2.501077 | -1.344464 | 0.004543 |
| 7 | N | -1.283828 | -1.923309 | -0.00297 |
| 6 | C | -0.312389 | -1.001406 | -0.00256 |
| 6 | C | -0.465135 | 0.399257 | 0.002792 |
| 6 | C | -1.803147 | 0.868714 | 0.007798 |
| 7 | N | 0.765089 | 1.055549 | 0.005446 |
| 6 | C | 1.664284 | 0.088161 | 0.000623 |
| 7 | N | 1.068107 | -1.170600 | -0.00488 |
| 7 | N | -2.106724 | 2.192875 | -0.00685 |
| 1 | H | -3.074814 | 2.471794 | 0.075864 |
| 1 | H | -1.371716 | 2.881319 | 0.073814 |
| 1 | H | -3.354046 | -2.030472 | 0.006672 |
| 1 | H | 2.745850 | 0.197955 | 0.000728 |
| 17 | Cl | 1.894575 | -2.681357 | -0.01067 |

N9-F-AD_9H $E_{tot}$ = -566.212576 a.u.
Gibbs free energy $E_G$= -566.149434 a.u

| | | | | |
|---|---|---|---|---|
| 7 | N | -2.420863 | -0.153021 | 0.018612 |
| 6 | C | -2.029488 | -1.444873 | 0.003636 |
| 7 | N | -0.781008 | -1.952904 | -0.006947 |
| 6 | C | 0.130221 | -0.969674 | -0.000767 |
| 6 | C | -0.103011 | 0.422643 | 0.014515 |
| 6 | C | -1.468545 | 0.807711 | 0.024368 |
| 7 | N | 1.080350 | 1.157816 | 0.016989 |
| 6 | C | 2.046185 | 0.252334 | 0.003543 |
| 7 | N | 1.513205 | -1.023904 | -0.007128 |
| 7 | N | -1.851221 | 2.109529 | 0.039282 |
| 1 | H | -2.836826 | 2.332922 | 0.046444 |
| 1 | H | -1.159120 | 2.845250 | 0.044523 |
| 1 | H | -2.840201 | -2.180417 | -0.000426 |
| 1 | H | 3.119039 | 0.424615 | 0.000723 |
| 9 | F | 2.261545 | -2.177326 | -0.022663 |

N9-H-AD_9H $E_{tot}$ = -467.096786 a.u.
Gibbs free energy $E_G$= -467.019892 a.u.

| | | | | |
|---|---|---|---|---|
| 7 | N | 1.962029 | 0.527275 | 0.004592 |
| 6 | C | 1.304445 | 1.706449 | 0.002924 |
| 7 | N | -0.02351 | 1.934087 | -0.00124 |
| 6 | C | -0.71338 | 0.780032 | -0.00262 |
| 6 | C | -0.17923 | -0.52523 | -0.00271 |
| 6 | C | 1.234633 | -0.6137 | -0.00179 |
| 7 | N | -1.18892 | -1.48459 | 0.002006 |
| 6 | C | -2.30842 | -0.7791 | 0.002786 |
| 7 | N | -2.08635 | 0.591594 | -0.00028 |
| 7 | N | 1.889474 | -1.80705 | -0.03371 |
| 1 | H | 2.892382 | -1.80781 | 0.096014 |
| 1 | H | 1.367686 | -2.66248 | 0.09916 |
| 1 | H | 1.940976 | 2.597346 | 0.008033 |
| 1 | H | -3.31484 | -1.19342 | 0.006012 |
| 1 | H | -2.78351 | 1.326384 | -0.00041 |

N7-Cl-AD_7H $E_{tot}$ = -926.670997 a.u.
Gibbs free energy $E_G$= -926.60822 a.u.

| | | | | |
|---|---|---|---|---|
| 7 | N | -2.80994 | -0.06949 | 0.017768 |
| 6 | C | -2.47553 | -1.38183 | 0.008677 |
| 7 | N | -1.25707 | -1.93534 | -0.00542 |
| 6 | C | -0.26094 | -1.02558 | -0.00659 |
| 6 | C | -0.4934 | 0.373434 | 0.000519 |
| 6 | C | -1.82357 | 0.847701 | 0.007338 |
| 7 | N | 0.788468 | 0.920042 | -0.01854 |
| 6 | C | 1.701343 | -0.11971 | -0.01863 |
| 7 | N | 1.104268 | -1.29765 | -0.02061 |
| 7 | N | -2.17925 | 2.171512 | -0.04328 |
| 1 | H | -3.15529 | 2.369302 | 0.144027 |
| 1 | H | -1.50309 | 2.88129 | 0.204152 |
| 1 | H | -3.32272 | -2.07531 | 0.015881 |
| 1 | H | 2.772026 | 0.07174 | -0.0225 |
| 17 | Cl | 1.200857 | 2.610378 | 0.058586 |

N7-F-AD_7H $E_{tot}$ = -566.205716 a.u.
Gibbs free energy $E_G$= -566.141068 a.u

| | | | | |
|---|---|---|---|---|
| 7 | N | -2.41746 | -0.18549 | 0.027054 |
| 6 | C | -2.00367 | -1.47591 | 0.019985 |
| 7 | N | -0.75362 | -1.95657 | -0.00561 |
| 6 | C | 0.1879 | -0.99223 | -0.00537 |
| 6 | C | -0.13359 | 0.389182 | 0.001511 |
| 6 | C | -1.48697 | 0.786921 | 0.003553 |
| 7 | N | 1.107094 | 0.994942 | -0.10065 |
| 6 | C | 2.094015 | 0.033891 | -0.03796 |
| 7 | N | 1.569943 | -1.18118 | -0.04036 |
| 7 | N | -1.90212 | 2.091333 | -0.05353 |
| 1 | H | -2.88617 | 2.252387 | 0.126297 |
| 1 | H | -1.25617 | 2.831819 | 0.185109 |
| 1 | H | -2.80726 | -2.2191 | 0.035566 |
| 1 | H | 3.148841 | 0.297721 | -0.03675 |
| 9 | F | 1.333613 | 2.347926 | 0.200396 |

N7-H-AD_7H $E_{tot}$ = -467.085338 a.u.
Gibbs free energy $E_G$= -467.00833 a.u.

| | | | | |
|---|---|---|---|---|
| 7 | N | -1.96277 | -0.50017 | -0.01 |
| 6 | C | -1.32248 | -1.69578 | -0.00871 |
| 7 | N | -0.0069 | -1.94233 | 0.012901 |
| 6 | C | 0.742057 | -0.8186 | 0.01347 |
| 6 | C | 0.182197 | 0.48572 | -0.00383 |
| 6 | C | -1.2189 | 0.620205 | -0.00265 |
| 7 | N | 1.272567 | 1.348798 | -0.01317 |
| 6 | C | 2.402066 | 0.548237 | -0.00608 |
| 7 | N | 2.131882 | -0.74505 | 0.016318 |
| 7 | N | -1.87607 | 1.839342 | -0.05822 |
| 1 | H | -2.87193 | 1.76392 | 0.130168 |
| 1 | H | -1.4308 | 2.624304 | 0.404726 |
| 1 | H | -1.98003 | -2.57131 | -0.01873 |
| 1 | H | 3.400837 | 0.982174 | -0.01724 |
| 1 | H | 1.261259 | 2.358063 | -0.08695 |

N9-Me-AD_9H $E_{tot}$ = -506.385688 a.u.
Gibbs free energy $E_G$= -506.283306 a.u.

| | | | | |
|---|---|---|---|---|
| 7 | N | -2.450516 | -0.103569 | 0.009877 |
| 6 | C | -2.083262 | -1.403057 | 0.006481 |
| 7 | N | -0.843296 | -1.930292 | -0.000717 |
| 6 | C | 0.094587 | -0.965434 | -0.002740 |
| 6 | C | -0.124525 | 0.427846 | -0.001128 |
| 6 | C | -1.479411 | 0.839344 | 0.002305 |
| 7 | N | 1.080340 | 1.123197 | 0.002965 |
| 6 | C | 2.000718 | 0.169386 | 0.001831 |
| 7 | N | 1.472158 | -1.114897 | -0.002203 |
| 7 | N | -1.842904 | 2.152234 | -0.030960 |
| 1 | H | -2.816749 | 2.381943 | 0.116650 |
| 1 | H | -1.137460 | 2.861610 | 0.114693 |
| 1 | H | -2.908600 | -2.122618 | 0.011991 |
| 1 | H | 3.077584 | 0.332237 | 0.004023 |
| 6 | C | 2.191342 | -2.381488 | -0.003453 |
| 1 | H | 1.926846 | -2.966437 | 0.890166 |
| 1 | H | 1.927557 | -2.964163 | -0.898744 |
| 1 | H | 3.269596 | -2.173625 | -0.002827 |

N7-Me-AD_7H $E_{tot}$ = -506.37318104 a.u.
Gibbs free energy $E_G$= -506.270869 a.u.

| | | | | |
|---|---|---|---|---|
| 7 | N | 2.446743 | -0.14999 | 0.009402 |
| 6 | C | 2.057048 | -1.44874 | -0.00936 |
| 7 | N | 0.816832 | -1.94935 | -0.03205 |
| 6 | C | -0.13973 | -0.99552 | -0.01999 |
| 6 | C | 0.147375 | 0.397285 | 0.010573 |
| 6 | C | 1.49752 | 0.803244 | 0.018128 |
| 7 | N | -1.09358 | 1.031363 | 0.039096 |
| 6 | C | -2.03139 | 0.015335 | 0.017295 |
| 7 | N | -1.51288 | -1.20176 | -0.01895 |
| 7 | N | 1.922128 | 2.121822 | 0.1011 |
| 1 | H | 2.914047 | 2.228108 | -0.09156 |
| 1 | H | 1.339939 | 2.82766 | -0.33339 |
| 1 | H | 2.875441 | -2.17637 | -0.01296 |
| 1 | H | -3.0969 | 0.244056 | 0.033242 |
| 6 | C | -1.37121 | 2.459976 | 0.087823 |
| 1 | H | -0.84714 | 2.923233 | 0.937307 |
| 1 | H | -1.06715 | 2.953372 | -0.8506 |
| 1 | H | -2.45227 | 2.601812 | 0.219406 |

N9-OMe-AD_9H $E_{tot}$ = -581.508846 a.u.
Gibbs free energy $E_G$= -581.405627 a.u.

| | | | | |
|---|---|---|---|---|
| 7 | N | -2.510247 | 0.037766 | -0.391422 |
| 6 | C | -2.198395 | -1.274229 | -0.329004 |
| 7 | N | -1.020709 | -1.850729 | -0.017466 |
| 6 | C | -0.089209 | -0.921640 | 0.258895 |
| 6 | C | -0.248257 | 0.480013 | 0.231682 |
| 6 | C | -1.541527 | 0.942143 | -0.117268 |
| 7 | N | 0.928927 | 1.141277 | 0.572816 |
| 6 | C | 1.801912 | 0.169407 | 0.794762 |
| 7 | N | 1.242028 | -1.086467 | 0.615578 |
| 7 | N | -1.837083 | 2.267341 | -0.214445 |
| 1 | H | -2.804804 | 2.534803 | -0.336688 |
| 1 | H | -1.169525 | 2.948552 | 0.120960 |
| 1 | H | -3.017382 | -1.962058 | -0.563158 |
| 1 | H | 2.840565 | 0.281035 | 1.097021 |
| 8 | O | 1.890796 | -2.276989 | 0.870633 |
| 6 | C | 2.145052 | -2.986402 | -0.374840 |
| 1 | H | 2.678234 | -3.895158 | -0.065137 |
| 1 | H | 1.192975 | -3.243140 | -0.862516 |
| 1 | H | 2.777799 | -2.371971 | -1.036586 |

N7-OMe-AD_7H $E_{tot}$ = -581.501347 a.u.
Gibbs free energy $E_G$= -581.397678 a.u.

| | | | | |
|---|---|---|---|---|
| 7 | N | -2.53626 | -0.35058 | 0.323233 |
| 6 | C | -1.94163 | -1.50205 | 0.729734 |
| 7 | N | -0.64819 | -1.84254 | 0.680655 |
| 6 | C | 0.139075 | -0.87098 | 0.168613 |
| 6 | C | -0.3744 | 0.378346 | -0.26526 |
| 6 | C | -1.75965 | 0.617505 | -0.19282 |
| 7 | N | 0.74333 | 1.068171 | -0.71135 |
| 6 | C | 1.842231 | 0.253733 | -0.53159 |
| 7 | N | 1.517218 | -0.92331 | -0.01428 |
| 7 | N | -2.34603 | 1.81091 | -0.57093 |
| 1 | H | -3.35613 | 1.760662 | -0.66067 |
| 1 | H | -1.87391 | 2.337653 | -1.29776 |
| 1 | H | -2.62441 | -2.25098 | 1.144179 |
| 1 | H | 2.839577 | 0.584679 | -0.81426 |
| 8 | O | 0.763669 | 2.365429 | -1.20413 |
| 6 | C | 0.847447 | 3.314218 | -0.10088 |
| 1 | H | -0.03796 | 3.22091 | 0.547772 |
| 1 | H | 1.77189 | 3.144644 | 0.473627 |
| 1 | H | 0.869213 | 4.299656 | -0.58569 |

N9-OH-AD_9H  $E_{tot}$ = -542.229559 a.u.
Gibbs free energy  $E_G$= -542.152512 a.u.

| | | | | |
|---|---|---|---|---|
| 7 | N | -2.305009 | -0.204970 | -0.042086 |
| 6 | C | -1.853037 | -1.475369 | -0.093507 |
| 7 | N | -0.576833 | -1.912508 | -0.080414 |
| 6 | C | 0.273483 | -0.875755 | -0.005510 |
| 6 | C | -0.023402 | 0.499803 | 0.055895 |
| 6 | C | -1.407512 | 0.808426 | 0.034837 |
| 7 | N | 1.136007 | 1.262268 | 0.122557 |
| 6 | C | 2.127457 | 0.373339 | 0.103196 |
| 7 | N | 1.647577 | -0.918968 | 0.027065 |
| 7 | N | -1.870580 | 2.083049 | 0.099941 |
| 1 | H | -2.865064 | 2.248886 | 0.029327 |
| 1 | H | -1.222714 | 2.858162 | 0.102415 |
| 1 | H | -2.624935 | -2.248814 | -0.154308 |
| 1 | H | 3.192765 | 0.584911 | 0.140208 |
| 8 | O | 2.417125 | -2.073211 | -0.011583 |
| 1 | H | 1.740027 | -2.779454 | -0.064406 |

N7-OH-AD_7H  $E_{tot}$ = -542.219894 a.u.
Gibbs free energy  $E_G$= -542.14133 a.u.

| | | | | |
|---|---|---|---|---|
| 7 | N | -2.38376 | -0.12831 | 0.13653 |
| 6 | C | -2.00841 | -1.43297 | 0.114493 |
| 7 | N | -0.77869 | -1.95154 | 0.015696 |
| 6 | C | 0.190107 | -1.0126 | -0.0563 |
| 6 | C | -0.08837 | 0.377706 | -0.02098 |
| 6 | C | -1.42567 | 0.811945 | 0.066478 |
| 7 | N | 1.162893 | 0.972569 | -0.08982 |
| 6 | C | 2.105878 | -0.02905 | -0.19567 |
| 7 | N | 1.559197 | -1.23555 | -0.16685 |
| 7 | N | -1.7939 | 2.140213 | 0.134858 |
| 1 | H | -2.7849 | 2.303951 | -0.01177 |
| 1 | H | -1.17014 | 2.814024 | -0.29445 |
| 1 | H | -2.83295 | -2.15038 | 0.179653 |
| 1 | H | 3.164979 | 0.203483 | -0.28749 |
| 8 | O | 1.415166 | 2.342025 | -0.1898 |
| 1 | H | 1.535616 | 2.640703 | 0.730742 |

N9-$NH_2$-AD_9H  $E_{tot}$ = -522.395376 a.u.
Gibbs free energy  $E_G$= -522.297862 a.u.

| | | | | |
|---|---|---|---|---|
| 7 | N | 2.380696 | -0.164701 | 0.078897 |
| 6 | C | 1.979598 | -1.452799 | 0.093225 |
| 7 | N | 0.724962 | -1.946608 | 0.046701 |
| 6 | C | -0.178350 | -0.954815 | -0.023435 |
| 6 | C | 0.072554 | 0.430527 | -0.047080 |
| 6 | C | 1.437249 | 0.805318 | 0.011817 |
| 7 | N | -1.112897 | 1.158866 | -0.130738 |
| 6 | C | -2.062925 | 0.236648 | -0.155738 |
| 7 | N | -1.562133 | -1.056921 | -0.093003 |
| 7 | N | 1.836036 | 2.106226 | 0.027819 |
| 1 | H | 2.823544 | 2.309695 | -0.048243 |
| 1 | H | 1.158404 | 2.835760 | -0.145995 |
| 1 | H | 2.783363 | -2.194158 | 0.148709 |
| 1 | H | -3.134771 | 0.411760 | -0.217875 |
| 7 | N | -2.348879 | -2.220731 | -0.104496 |
| 1 | H | -2.029775 | -2.795552 | -0.890194 |
| 1 | H | -2.120496 | -2.750431 | 0.742201 |

N7-$NH_2$-AD_7H  $E_{tot}$ = -522.384077 a.u.
Gibbs free energy  $E_G$= -522.292721 a.u.

| | | | | |
|---|---|---|---|---|
| 7 | N | -2.12393 | 0.625906 | -0.07454 |
| 6 | C | -2.34242 | -0.71339 | -0.09109 |
| 7 | N | -1.45503 | -1.71394 | -0.02447 |
| 6 | C | -0.17887 | -1.27892 | 0.08035 |
| 6 | C | 0.166873 | 0.092241 | 0.110552 |
| 6 | C | -0.85333 | 1.061854 | 0.019326 |
| 7 | N | 1.552105 | 0.117297 | 0.226936 |
| 6 | C | 1.966397 | -1.2065 | 0.262553 |
| 7 | N | 0.966373 | -2.06777 | 0.176647 |
| 7 | N | -0.6077 | 2.409811 | -0.02213 |
| 1 | H | -1.40515 | 3.021511 | 0.100249 |
| 1 | H | 0.310055 | 2.73795 | 0.252611 |
| 1 | H | -3.39487 | -1.00595 | -0.17013 |
| 1 | H | 3.022307 | -1.46061 | 0.355977 |
| 7 | N | 2.320652 | 1.293652 | 0.335376 |
| 1 | H | 2.871907 | 1.245883 | 1.195308 |
| 1 | H | 2.96801 | 1.337992 | -0.45529 |

N3-NO$_2$-AD_3H   $E_{tot}$ = -671.477355 a.u.
Gibbs free energy   $E_G$= -671.403201 a.u.

| | | | | |
|---|---|---|---|---|
| 7 | N | -2.7773 | 0.027249 | 0.0004 |
| 6 | C | -2.52707 | -1.25953 | 7.52E-05 |
| 7 | N | -1.26534 | -1.81187 | -0.00025 |
| 6 | C | -0.15275 | -0.97858 | -0.0002 |
| 6 | C | -0.40536 | 0.435891 | 0.000162 |
| 6 | C | -1.72224 | 0.914953 | 0.000426 |
| 7 | N | 0.804574 | 1.085182 | 0.000267 |
| 6 | C | 1.681111 | 0.061891 | -8.4E-05 |
| 7 | N | 1.164392 | -1.22254 | -0.00032 |
| 7 | N | -2.03047 | 2.229246 | 0.000536 |
| 1 | H | -2.99894 | 2.51909 | 0.00163 |
| 1 | H | -1.28541 | 2.913659 | 0.001334 |
| 1 | H | -3.3461 | -1.97689 | 5.04E-05 |
| 1 | H | 2.7599 | 0.220202 | -9.3E-05 |
| 7 | N | -1.16273 | -3.33664 | -0.00068 |
| 8 | O | -0.03913 | -3.7683 | -0.0012 |
| 8 | O | -2.23121 | -3.9196 | -0.00024 |

N1-NO$_2$-AD_1H   $E_{tot}$ = -671.46525243 a.u.
Gibbs free energy   $E_G$= -671.392863a.u.

| | | | | |
|---|---|---|---|---|
| 7 | N | 2.656686 | 0.24338 | 0.011821 |
| 6 | C | 2.502994 | -1.17429 | 0.011646 |
| 7 | N | 1.360796 | -1.78633 | 0.012885 |
| 6 | C | 0.249064 | -0.97894 | 0.006173 |
| 6 | C | 0.327018 | 0.454863 | 0.017978 |
| 6 | C | 1.551423 | 1.118154 | 0.015819 |
| 7 | N | -0.9407 | 0.985578 | 0.009605 |
| 6 | C | -1.69917 | -0.13504 | -0.00432 |
| 7 | N | -1.04413 | -1.34975 | -0.00626 |
| 7 | N | 1.647155 | 2.462755 | -0.06229 |
| 1 | H | 2.507471 | 2.944408 | 0.155964 |
| 1 | H | 0.755688 | 2.946426 | -0.02149 |
| 1 | H | 3.447904 | -1.71175 | 0.024863 |
| 1 | H | -2.78896 | -0.08555 | -0.01111 |
| 7 | N | 4.040067 | 0.738297 | 0.035553 |
| 8 | O | 4.901758 | -0.07913 | -0.21965 |
| 8 | O | 4.198566 | 1.918163 | 0.323123 |

N3-CN-AD_3H   $E_{tot}$ =  -559.245777 a.u.
Gibbs free energy   $E_G$= -559.173896 a.u.

| | | | | |
|---|---|---|---|---|
| 7 | N | -2.77514 | 0.005039 | 0.000294 |
| 6 | C | -2.54144 | -1.27687 | 2.19E-05 |
| 7 | N | -1.26724 | -1.85239 | -0.00027 |
| 6 | C | -0.16932 | -0.98391 | -0.00021 |
| 6 | C | -0.40507 | 0.422687 | 9.14E-05 |
| 6 | C | -1.72256 | 0.901019 | 0.000283 |
| 7 | N | 0.819897 | 1.050807 | 0.00031 |
| 6 | C | 1.677521 | 0.009539 | -0.00026 |
| 7 | N | 1.131584 | -1.2668 | -0.0003 |
| 7 | N | -2.04057 | 2.212037 | 0.000129 |
| 1 | H | -3.01088 | 2.495988 | 0.002036 |
| 1 | H | -1.30106 | 2.902502 | 0.001626 |
| 1 | H | -3.365 | -1.99348 | 4E-05 |
| 1 | H | 2.758718 | 0.148425 | -0.00021 |
| 6 | C | -1.129 | -3.20313 | -0.00055 |
| 7 | N | -1.06102 | -4.37267 | -0.00078 |

N1-CN-AD_1H   $E_{tot}$ =  -559.234917 a.u.
Gibbs free energy   $E_G$= -559.165285 a.u.

| | | | | |
|---|---|---|---|---|
| 7 | N | 2.663727 | 0.240794 | 0.024688 |
| 6 | C | 2.51102 | -1.18871 | -0.0039 |
| 7 | N | 1.371089 | -1.79331 | -0.02279 |
| 6 | C | 0.257725 | -0.9846 | -0.01417 |
| 6 | C | 0.322554 | 0.457298 | 0.014199 |
| 6 | C | 1.54886 | 1.10367 | 0.034375 |
| 7 | N | -0.9501 | 0.973484 | 0.016141 |
| 6 | C | -1.6971 | -0.15437 | -0.01042 |
| 7 | N | -1.03171 | -1.36268 | -0.0296 |
| 7 | N | 1.743437 | 2.438327 | 0.061228 |
| 1 | H | 2.662832 | 2.860819 | 0.07593 |
| 1 | H | 0.917644 | 3.023942 | 0.067534 |
| 1 | H | 3.464585 | -1.71948 | -0.00812 |
| 1 | H | -2.78729 | -0.11543 | -0.0165 |
| 6 | C | 3.907769 | 0.766064 | 0.042725 |
| 7 | N | 4.977422 | 1.24908 | 0.058885 |

N3-CHO-AD_3H $E_{tot}$ = -580.360120 a.u.
Gibbs free energy $E_G$= -580.27727 a.u.

| | | | | |
|---|---|---|---|---|
| 7 | N | -2.81742 | 0.167658 | 0.000128 |
| 6 | C | -2.71025 | -1.13648 | -3.7E-05 |
| 7 | N | -1.51578 | -1.83841 | -9.6E-05 |
| 6 | C | -0.33895 | -1.09138 | -2.5E-05 |
| 6 | C | -0.41172 | 0.331053 | 0.00014 |
| 6 | C | -1.67226 | 0.944424 | 0.000204 |
| 7 | N | 0.874989 | 0.824711 | 0.00069 |
| 6 | C | 1.613536 | -0.30296 | -0.00068 |
| 7 | N | 0.931896 | -1.51144 | 0.000157 |
| 7 | N | -1.85054 | 2.281935 | 0.000175 |
| 1 | H | -2.78689 | 2.663566 | 0.001551 |
| 1 | H | -1.04344 | 2.891916 | 0.001408 |
| 1 | H | -3.604 | -1.76174 | -3.6E-05 |
| 1 | H | 2.703656 | -0.28212 | -0.00035 |
| 6 | C | -1.49166 | -3.27221 | -0.00018 |
| 8 | O | -2.49661 | -3.94893 | -0.00017 |
| 1 | H | -0.45082 | -3.64914 | -6.2E-05 |

N1-CHO-AD_1H $E_{tot}$ = -580.339335 a.u.
Gibbs free energy $E_G$= -580.256612 a.u.

| | | | | |
|---|---|---|---|---|
| 7 | N | 2.682397 | 0.133981 | 0.023441 |
| 6 | C | 2.507068 | -1.27607 | 0.067464 |
| 7 | N | 1.363567 | -1.89071 | 0.050699 |
| 6 | C | 0.2572 | -1.07735 | -0.01431 |
| 6 | C | 0.341987 | 0.365783 | -0.04657 |
| 6 | C | 1.571717 | 0.991625 | 0.007244 |
| 7 | N | -0.9278 | 0.899153 | -0.04941 |
| 6 | C | -1.68627 | -0.21665 | -0.03309 |
| 7 | N | -1.0352 | -1.43815 | -0.0003 |
| 7 | N | 1.738374 | 2.357876 | -0.00684 |
| 1 | H | 2.431454 | 2.763706 | 0.614171 |
| 1 | H | 0.839273 | 2.83367 | 0.040359 |
| 1 | H | 3.450507 | -1.82023 | 0.099519 |
| 1 | H | -2.77627 | -0.16678 | -0.03925 |
| 6 | C | 4.009798 | 0.637719 | -0.12443 |
| 8 | O | 5.00343 | -0.04652 | -0.01227 |
| 1 | H | 4.02893 | 1.713806 | -0.38612 |

N3-CHO-AD_3H $E_{tot}$ = -580.351426 a.u.
Gibbs free energy $E_G$= -580.269146 a.u.

| | | | | |
|---|---|---|---|---|
| 7 | N | -2.70587 | 0.178098 | 0.000313 |
| 6 | C | -2.55931 | -1.11987 | 0.000141 |
| 7 | N | -1.35554 | -1.81146 | -9.2E-05 |
| 6 | C | -0.18793 | -1.04419 | -8.7E-05 |
| 6 | C | -0.30803 | 0.382933 | 9.2E-05 |
| 6 | C | -1.57654 | 0.975815 | 0.000251 |
| 7 | N | 0.956436 | 0.921791 | 0.000143 |
| 6 | C | 1.733898 | -0.18154 | -7.7E-06 |
| 7 | N | 1.09886 | -1.41164 | -0.00014 |
| 7 | N | -1.77495 | 2.310673 | 9.2E-05 |
| 1 | H | -2.71574 | 2.680627 | 0.001536 |
| 1 | H | -0.9749 | 2.930064 | 0.001114 |
| 1 | H | -3.4516 | -1.75128 | 0.00019 |
| 1 | H | 2.822858 | -0.12243 | -1E-05 |
| 6 | C | -1.41108 | -3.24592 | -0.0003 |
| 8 | O | -0.45212 | -3.97389 | -0.00052 |
| 1 | H | -2.46932 | -3.59287 | -0.00019 |

N1-CHO-AD_1H $E_{tot}$ = -580.347637 a.u.
Gibbs free energy $E_G$= -580.264507 a.u.

| | | | | |
|---|---|---|---|---|
| 7 | N | 2.674345 | 0.288923 | 0.005122 |
| 6 | C | 2.517332 | -1.13291 | -0.01834 |
| 7 | N | 1.393466 | -1.77108 | -0.02806 |
| 6 | C | 0.267558 | -0.97659 | -0.01397 |
| 6 | C | 0.321466 | 0.461053 | 0.009918 |
| 6 | C | 1.540615 | 1.129849 | 0.02001 |
| 7 | N | -0.95657 | 0.966886 | 0.019343 |
| 6 | C | -1.69464 | -0.16599 | 0.001081 |
| 7 | N | -1.01813 | -1.36963 | -0.01951 |
| 7 | N | 1.673777 | 2.469452 | 0.042029 |
| 1 | H | 2.590322 | 2.902331 | 0.048634 |
| 1 | H | 0.815109 | 3.006575 | 0.052018 |
| 1 | H | 3.467892 | -1.67118 | -0.02798 |
| 6 | H | -2.7853 | -0.13707 | 0.002477 |
| 1 | C | 3.996225 | 0.776626 | 0.012002 |
| 1 | O | 4.316658 | 1.952708 | 0.031242 |
| 8 | H | 4.735718 | -0.04976 | -0.00243 |

N3-Cl-AD_3H $E_{tot}$ = -926.669178 a.u.
Gibbs free energy $E_G$= -926.605777 a.u.

| | | | | |
|---|---|---|---|---|
| 7 | N | -2.78318 | 0.029191 | 0.000415 |
| 6 | C | -2.54584 | -1.26662 | 0.000168 |
| 7 | N | -1.28957 | -1.80613 | -0.00035 |
| 6 | C | -0.18704 | -0.98552 | -0.00042 |
| 6 | C | -0.41517 | 0.430865 | -0.00014 |
| 6 | C | -1.73654 | 0.913298 | 0.000174 |
| 7 | N | 0.808648 | 1.051031 | -0.00057 |
| 6 | C | 1.665108 | 0.006326 | 0.000617 |
| 7 | N | 1.120357 | -1.26408 | -0.00078 |
| 7 | N | -2.03281 | 2.234643 | -0.00049 |
| 1 | H | -2.99709 | 2.535912 | 0.002599 |
| 1 | H | -1.2813 | 2.9109 | 0.001832 |
| 1 | H | -3.36908 | -1.98193 | 0.000289 |
| 1 | H | 2.746258 | 0.145058 | 0.000346 |
| 17 | Cl | -1.10206 | -3.55386 | -0.00074 |

N1-Cl-AD_1H $E_{tot}$ = -926.654125 a.u.
Gibbs free energy $E_G$= -926.590587 a.u.

| | | | | |
|---|---|---|---|---|
| 7 | N | 2.633253 | 0.220162 | 0.003839 |
| 6 | C | 2.507219 | -1.17674 | 0.004585 |
| 7 | N | 1.356754 | -1.78511 | -0.00784 |
| 6 | C | 0.244851 | -0.98511 | -0.01426 |
| 6 | C | 0.31428 | 0.453804 | -0.01017 |
| 6 | C | 1.557079 | 1.092119 | -0.00311 |
| 7 | N | -0.94717 | 0.980988 | -0.0032 |
| 6 | C | -1.70473 | -0.14925 | -0.00551 |
| 7 | N | -1.05305 | -1.357 | -0.01156 |
| 7 | N | 1.729864 | 2.438417 | -0.05745 |
| 1 | H | 2.601808 | 2.853112 | 0.246752 |
| 1 | H | 0.879231 | 2.974822 | 0.078561 |
| 1 | H | 3.456401 | -1.71383 | 0.01611 |
| 1 | H | -2.79488 | -0.09768 | -0.00126 |
| 17 | Cl | 4.258061 | 0.884796 | 0.039076 |

N3-F-AD_3H $E_{tot}$ = -566.205357 a.u.
Gibbs free energy $E_G$= -566.140402 a.u.

| | | | | |
|---|---|---|---|---|
| 7 | N | -2.79193 | 0.033398 | 0.000232 |
| 6 | C | -2.5517 | -1.26487 | 8.72E-05 |
| 7 | N | -1.29448 | -1.77683 | -0.00032 |
| 6 | C | -0.17656 | -0.98886 | -0.00039 |
| 6 | C | -0.41728 | 0.42757 | -0.00022 |
| 6 | C | -1.7406 | 0.911017 | 1.46E-05 |
| 7 | N | 0.804483 | 1.049975 | -0.00027 |
| 6 | C | 1.666947 | 0.009941 | 0.000238 |
| 7 | N | 1.131881 | -1.26556 | -0.00063 |
| 7 | N | -2.02974 | 2.234132 | -0.00057 |
| 1 | H | -2.99237 | 2.540495 | 0.002776 |
| 1 | H | -1.27483 | 2.906609 | 0.002011 |
| 1 | H | -3.36158 | -1.99544 | 0.000197 |
| 1 | H | 2.747514 | 0.154001 | 0.000279 |
| 9 | F | -1.16951 | -3.16962 | -0.00044 |

N1-F-AD_1H $E_{tot}$ = -566.192113 a.u.
Gibbs free energy $E_G$= -566.126786 a.u.

| | | | | |
|---|---|---|---|---|
| 7 | N | 2.586115 | 0.209885 | 0.000213 |
| 6 | C | 2.503965 | -1.17488 | 0.006816 |
| 7 | N | 1.352731 | -1.79269 | -0.00444 |
| 6 | C | 0.24316 | -0.9892 | -0.01311 |
| 6 | C | 0.298387 | 0.456345 | -0.01196 |
| 6 | C | 1.537952 | 1.100457 | -0.0037 |
| 7 | N | -0.96585 | 0.971803 | -0.00836 |
| 6 | C | -1.7141 | -0.16626 | -0.00882 |
| 7 | N | -1.05388 | -1.36682 | -0.01167 |
| 7 | N | 1.75796 | 2.439718 | -0.05575 |
| 1 | H | 2.652107 | 2.81653 | 0.230666 |
| 1 | H | 0.933034 | 3.00913 | 0.098098 |
| 1 | H | 3.465811 | -1.68906 | 0.020065 |
| 1 | H | -2.80445 | -0.12243 | -0.00582 |
| 9 | F | 3.868404 | 0.771293 | 0.04035 |

N3-H-AD_3H $E_{tot}$ = -467.085495 a.u.
Gibbs free energy $E_G$= -467.008726 a.u.

| | | | | |
|---|---|---|---|---|
| 7 | N | 1.956751 | 0.45435 | 0.000391 |
| 6 | C | 1.376085 | 1.637636 | 0.000118 |
| 7 | N | 0.027617 | 1.844188 | -0.00022 |
| 6 | C | -0.80757 | 0.749745 | 0.000092 |
| 6 | C | -0.23136 | -0.55468 | 0.000106 |
| 6 | C | 1.170301 | -0.6737 | -6.9E-05 |
| 7 | N | -1.256 | -1.47343 | 0.000079 |
| 6 | C | -2.3504 | -0.67884 | 0.000098 |
| 7 | N | -2.14758 | 0.689367 | -5.4E-05 |
| 7 | N | 1.805039 | -1.86988 | -0.00118 |
| 1 | H | 2.814875 | -1.90314 | 0.00295 |
| 1 | H | 1.259925 | -2.72134 | 0.002325 |
| 1 | H | 1.998167 | 2.535542 | 0.000062 |
| 1 | H | -3.3607 | -1.09023 | 0.000181 |
| 1 | H | -0.35538 | 2.786045 | -0.00071 |

N1-H-AD_1H $E_{tot}$ = -467.069792 a.u.
Gibbs free energy $E_G$= -466.99272 a.u.

| | | | | |
|---|---|---|---|---|
| 7 | N | 1.843838 | -0.48974 | -0.00037 |
| 6 | C | 1.238324 | -1.74401 | 0.005322 |
| 7 | N | -0.0518 | -1.94645 | 0.002588 |
| 6 | C | -0.8139 | -0.80699 | -0.00787 |
| 6 | C | -0.25316 | 0.529442 | -0.00976 |
| 6 | C | 1.1257 | 0.687191 | -0.00332 |
| 7 | N | -1.26468 | 1.454384 | 0.003038 |
| 6 | C | -2.35845 | 0.651982 | 0.008009 |
| 7 | N | -2.15946 | -0.7083 | 0.0051 |
| 7 | N | 1.789146 | 1.88985 | -0.07085 |
| 1 | H | 2.673125 | 1.975185 | 0.421389 |
| 1 | H | 1.168191 | 2.684323 | 0.060485 |
| 1 | H | 1.937806 | -2.58348 | 0.014141 |
| 1 | H | -3.36668 | 1.070055 | 0.017315 |
| 1 | H | 2.857129 | -0.45 | -0.04418 |

N3-Me-AD_3H $E_{tot}$ = -506.375091 a.u.
Gibbs free energy $E_G$= -506.274446 a.u.

| | | | | |
|---|---|---|---|---|
| 7 | N | 2.433879 | -0.03502 | -0.00038 |
| 6 | C | 2.132468 | -1.32064 | -0.00034 |
| 7 | N | 0.872358 | -1.84534 | -4.1E-05 |
| 6 | C | -0.17634 | -0.95029 | 0.000148 |
| 6 | C | 0.083123 | 0.451046 | 0.000169 |
| 6 | C | 1.419235 | 0.887757 | 4.86E-05 |
| 7 | N | -1.12589 | 1.109401 | -0.0001 |
| 6 | C | -2.00879 | 0.086039 | 0.00031 |
| 7 | N | -1.49708 | -1.19853 | -1.5E-05 |
| 7 | N | 1.766694 | 2.197921 | 0.001489 |
| 1 | H | 2.742485 | 2.459345 | -0.00503 |
| 1 | H | 1.042711 | 2.903242 | -0.00384 |
| 1 | H | 2.946441 | -2.05004 | -0.0007 |
| 1 | H | -3.08674 | 0.254249 | 0.000125 |
| 6 | C | 0.612774 | -3.28982 | 5.17E-05 |
| 1 | H | 0.029884 | -3.55391 | 0.89405 |
| 1 | H | 0.027508 | -3.55348 | -0.8925 |
| 1 | H | 1.572452 | -3.82277 | -0.00133 |

N3-OMe-AD_3H $E_{tot}$ = -581.500654 a.u.
Gibbs free energy $E_G$= -581.397613 a.u.

| | | | | |
|---|---|---|---|---|
| 7 | N | -2.67211 | -0.08031 | -0.10663 |
| 6 | C | -2.3482 | -1.35395 | 0.029047 |
| 7 | N | -1.07675 | -1.81068 | 0.198799 |
| 6 | C | -0.03107 | -0.91541 | 0.237496 |
| 6 | C | -0.33896 | 0.471363 | 0.079519 |
| 6 | C | -1.68055 | 0.864214 | -0.08684 |
| 7 | N | 0.840908 | 1.174803 | 0.14876 |
| 6 | C | 1.752992 | 0.195308 | 0.331347 |
| 7 | N | 1.288963 | -1.1062 | 0.393596 |
| 7 | N | -2.04721 | 2.160154 | -0.24557 |
| 1 | H | -3.02795 | 2.400177 | -0.28496 |
| 1 | H | -1.34528 | 2.883596 | -0.16623 |
| 1 | H | -3.11731 | -2.12793 | 0.024922 |
| 1 | H | 2.818209 | 0.408162 | 0.428407 |
| 8 | O | -0.90237 | -3.17445 | 0.43292 |
| 6 | C | -0.15726 | -3.78749 | -0.6587 |
| 1 | H | -0.70431 | -3.65551 | -1.60699 |
| 1 | H | 0.852475 | -3.35657 | -0.71303 |
| 1 | H | -0.11537 | -4.84953 | -0.38314 |

N1-Me-AD_1H $E_{tot}$ = -506.356916 a.u.
Gibbs free energy $E_G$= -506.254292 a.u.

| | | | | |
|---|---|---|---|---|
| 7 | N | 2.662128 | 0.234278 | 0.010102 |
| 6 | C | 2.494049 | -1.15377 | 0.033182 |
| 7 | N | 1.352484 | -1.78851 | 0.026004 |
| 6 | C | 0.244131 | -0.98728 | -0.00241 |
| 6 | C | 0.321675 | 0.454198 | -0.018 |
| 6 | C | 1.564767 | 1.074732 | -0.00841 |
| 7 | N | -0.94187 | 0.985295 | -0.02402 |
| 6 | C | -1.7024 | -0.1405 | -0.01487 |
| 7 | N | -1.05674 | -1.35255 | 0.000867 |
| 7 | N | 1.739022 | 2.437828 | -0.09845 |
| 1 | H | 2.513371 | 2.862052 | 0.400795 |
| 1 | H | 0.859505 | 2.937655 | 0.008365 |
| 1 | H | 3.432492 | -1.71321 | 0.059074 |
| 1 | H | -2.79254 | -0.08601 | -0.01769 |
| 6 | C | 4.014637 | 0.79914 | -0.00635 |
| 1 | H | 4.126665 | 1.480111 | -0.86342 |
| 1 | H | 4.224933 | 1.343454 | 0.929327 |
| 1 | H | 4.735788 | -0.02087 | -0.10197 |

N1-OMe-AD_1H $E_{tot}$ = -581.487770 a.u.
Gibbs free energy $E_G$= -581.383897 a.u.

| | | | | |
|---|---|---|---|---|
| 7 | N | 2.560577 | 0.518413 | -0.11374 |
| 6 | C | 2.560108 | -0.85331 | 0.117397 |
| 7 | N | 1.474944 | -1.56455 | 0.274565 |
| 6 | C | 0.301352 | -0.86291 | 0.190088 |
| 6 | C | 0.236649 | 0.559261 | -0.07123 |
| 6 | C | 1.416246 | 1.279827 | -0.23667 |
| 7 | N | -1.0708 | 0.960587 | -0.12982 |
| 6 | C | -1.71878 | -0.21225 | 0.100211 |
| 7 | N | -0.95917 | -1.3377 | 0.296345 |
| 7 | N | 1.521052 | 2.628317 | -0.43966 |
| 1 | H | 2.339315 | 2.957343 | -0.94083 |
| 1 | H | 0.638822 | 3.063554 | -0.69096 |
| 1 | H | 3.557973 | -1.29519 | 0.143636 |
| 1 | H | -2.80897 | -0.25864 | 0.12635 |
| 8 | O | 3.791816 | 1.156387 | -0.28211 |
| 6 | C | 4.291493 | 1.615728 | 1.008648 |
| 1 | H | 5.238727 | 2.117999 | 0.770762 |
| 1 | H | 3.579737 | 2.32123 | 1.464316 |
| 1 | H | 4.466672 | 0.75491 | 1.672768 |

N3-OH-AD_3H    $E_{tot}$ = -542.223954 a.u.
Gibbs free energy    $E_G$= -542.14811 a.u.

| | | | | |
|---|---|---|---|---|
| 7 | N | -2.78957 | 0.024269 | -0.00676 |
| 6 | C | -2.55563 | -1.28091 | -0.02666 |
| 7 | N | -1.30844 | -1.80994 | -0.03826 |
| 6 | C | -0.2217 | -0.98303 | -0.03004 |
| 6 | C | -0.41823 | 0.422508 | -0.00888 |
| 6 | C | -1.74437 | 0.906212 | 0.0025 |
| 7 | N | 0.827934 | 1.008972 | -0.00451 |
| 6 | C | 1.658971 | -0.05701 | -0.02236 |
| 7 | N | 1.077726 | -1.31427 | -0.03847 |
| 7 | N | -2.03989 | 2.229665 | 0.021921 |
| 1 | H | -3.0046 | 2.529321 | 0.030061 |
| 1 | H | -1.29045 | 2.907524 | 0.029646 |
| 1 | H | -3.37879 | -1.99658 | -0.03407 |
| 1 | H | 2.743342 | 0.054696 | -0.02401 |
| 8 | O | -1.13206 | -3.19383 | -0.05954 |
| 1 | H | -0.15251 | -3.27441 | -0.06358 |

N3-NH2-AD_3H    $E_{tot}$ = -522.386047 a.u.
Gibbs free energy    $E_G$= -522.295526 a.u.

| | | | | |
|---|---|---|---|---|
| 7 | N | -0.89433 | -1.49178 | 0.83506 |
| 6 | C | 0.42147 | -1.59342 | 0.843451 |
| 7 | N | 1.264938 | -0.86687 | 0.054485 |
| 6 | C | 0.712729 | 0.044616 | -0.8203 |
| 6 | C | -0.70256 | 0.194426 | -0.86983 |
| 6 | C | -1.4932 | -0.5988 | -0.01816 |
| 7 | N | -0.99301 | 1.155839 | -1.81292 |
| 6 | C | 0.234357 | 1.507922 | -2.25321 |
| 7 | N | 1.324093 | 0.866354 | -1.68758 |
| 7 | N | -2.84562 | -0.52798 | 0.005911 |
| 1 | H | -3.36827 | -1.11708 | 0.638874 |
| 1 | H | -3.32073 | 0.120818 | -0.60664 |
| 1 | H | 0.918109 | -2.29691 | 1.513911 |
| 1 | H | 0.376109 | 2.266587 | -3.02385 |
| 7 | N | 2.664732 | -1.07788 | 0.166769 |
| 1 | H | 3.074207 | -0.15884 | 0.366481 |
| 1 | H | 2.992493 | -1.30954 | -0.7772 |

N1-OH-AD_1H    $E_{tot}$ = -542.206189 a.u.
Gibbs free energy    $E_G$= -542.127644 a.u.

| | | | | |
|---|---|---|---|---|
| 7 | N | 2.612927 | 0.298102 | 0.013935 |
| 6 | C | 2.521286 | -1.091 | -0.03545 |
| 7 | N | 1.389184 | -1.74226 | -0.02538 |
| 6 | C | 0.264625 | -0.96106 | 0.020835 |
| 6 | C | 0.296903 | 0.485398 | 0.044968 |
| 6 | C | 1.522705 | 1.145406 | 0.03073 |
| 7 | N | -0.98006 | 0.977437 | 0.061838 |
| 6 | C | -1.70688 | -0.17146 | 0.051098 |
| 7 | N | -1.02516 | -1.36192 | 0.024963 |
| 7 | N | 1.715699 | 2.496032 | 0.107975 |
| 1 | H | 2.559693 | 2.872702 | -0.30974 |
| 1 | H | 0.865603 | 3.031146 | -0.04153 |
| 1 | H | 3.48861 | -1.59406 | -0.08922 |
| 1 | H | -2.79801 | -0.14879 | 0.061289 |
| 8 | O | 3.887732 | 0.875445 | -0.07129 |
| 1 | H | 4.168377 | 1.001706 | 0.854884 |

N1-NH2-AD_1H    $E_{tot}$ = -522.369277 a.u.
Gibbs free energy    $E_G$= -522.278009 a.u.

| | | | | |
|---|---|---|---|---|
| 7 | N | 2.64099 | 0.32375 | -0.01331 |
| 6 | C | 2.52917 | -1.07 | -0.11784 |
| 7 | N | 1.407725 | -1.73309 | -0.15395 |
| 6 | C | 0.275914 | -0.96077 | -0.08247 |
| 6 | C | 0.302855 | 0.476917 | 0.020337 |
| 6 | C | 1.526447 | 1.145218 | 0.052974 |
| 7 | N | -0.97754 | 0.960641 | 0.077905 |
| 6 | C | -1.69995 | -0.18856 | 0.007666 |
| 7 | N | -1.0119 | -1.37165 | -0.09045 |
| 7 | N | 1.686737 | 2.489665 | 0.10423 |
| 1 | H | 2.603647 | 2.86803 | 0.304212 |
| 1 | H | 0.854602 | 3.033472 | 0.295713 |
| 1 | H | 3.494024 | -1.58337 | -0.16497 |
| 1 | H | -2.79108 | -0.17257 | 0.029008 |
| 7 | N | 3.90802 | 0.953791 | 0.049412 |
| 1 | H | 4.442643 | 0.527946 | 0.809108 |
| 1 | H | 4.403617 | 0.778963 | -0.82769 |

**Table S15.** Values of total energies and Gibbs free energies, cartesian coordinates of equilibrium geometries of C8-X and C2-X substituted purine (PU) 9H, 7H, 3H and 1H tautomers.

C8-$NO_2$-PU_9H $E_{tot}$ = -616.168587 a.u.
Gibbs free energy $E_G$=-616.108778 a.u.

| | | | | |
|---|---|---|---|---|
| 7 | N | -3.21234 | 0.665164 | 0.000032 |
| 6 | C | -3.09987 | -0.68752 | 0.000431 |
| 7 | N | -1.97707 | -1.43298 | 0.000239 |
| 6 | C | -0.87638 | -0.6769 | 0.000192 |
| 6 | C | -0.83899 | 0.751088 | 0.000089 |
| 6 | C | -2.0872 | 1.396861 | -8.9E-05 |
| 7 | N | 0.472261 | 1.210068 | 0.00031 |
| 6 | C | 1.180865 | 0.102093 | -0.00005 |
| 7 | N | 0.447624 | -1.06877 | 0.000045 |
| 1 | H | -4.04243 | -1.24295 | -0.00019 |
| 1 | H | 0.836168 | -2.00608 | 0.000203 |
| 1 | H | -2.18138 | 2.487997 | -9.3E-05 |
| 7 | N | 2.64252 | 0.027359 | -0.00025 |
| 8 | O | 3.11465 | -1.12157 | -0.00031 |
| 8 | O | 3.273602 | 1.07674 | -0.00044 |

C2-$NO_2$-PU_9H $E_{tot}$ = -616.169698 a.u.
Gibbs free energy $E_G$=-616.108863 a.u

| | | | | |
|---|---|---|---|---|
| 7 | N | -0.77871 | 1.400018 | -0.00011 |
| 6 | C | -1.05727 | 0.095056 | -9.3E-05 |
| 7 | N | -0.2431 | -0.96291 | -8E-06 |
| 6 | C | 1.035035 | -0.58335 | 0.000125 |
| 6 | C | 1.511644 | 0.762079 | 0.000166 |
| 6 | C | 0.520509 | 1.754883 | -6E-06 |
| 7 | N | 2.901634 | 0.785829 | 0.000547 |
| 6 | C | 3.251993 | -0.48944 | 0.000097 |
| 7 | N | 2.173086 | -1.36453 | 0.00023 |
| 1 | H | 2.200888 | -2.37792 | 0.000512 |
| 1 | H | 0.754512 | 2.824021 | 0.000135 |
| 1 | H | 4.27696 | -0.85636 | 0.00012 |
| 7 | N | -2.54562 | -0.2517 | -0.00031 |
| 8 | O | -2.83395 | -1.44614 | 0.000405 |
| 8 | O | -3.33541 | 0.687129 | -0.00103 |

C8-CN-PU_9H $E_{tot}$ = -503.927175 a.u.
Gibbs free energy $E_G$= -503.86959 a.u.

| | | | | |
|---|---|---|---|---|
| 7 | N | -2.84737 | 0.654904 | -1E-06 |
| 6 | C | -2.73288 | -0.69601 | 0.000342 |
| 7 | N | -1.60678 | -1.43792 | 0.000208 |
| 6 | C | -0.50826 | -0.67817 | 0.000136 |
| 6 | C | -0.47233 | 0.74656 | 0.000039 |
| 6 | C | -1.72215 | 1.388475 | -0.00011 |
| 7 | N | 0.835442 | 1.208886 | 0.000313 |
| 6 | C | 1.572081 | 0.101683 | -0.00029 |
| 7 | N | 0.813518 | -1.07079 | 0.000063 |
| 1 | H | -3.6734 | -1.25492 | -0.00013 |
| 1 | H | 1.165045 | -2.02216 | 0.000484 |
| 1 | H | -1.81927 | 2.479371 | -0.0001 |
| 6 | C | 2.996748 | 0.054507 | -0.0004 |
| 7 | N | 4.16638 | -0.02716 | -0.00038 |

C2-CN-PU_9H $E_{tot}$ = -503.928272 a.u.
Gibbs free energy $E_G$= -503.870719 a.u.

| | | | | |
|---|---|---|---|---|
| 7 | N | -1.15942 | 1.351228 | -0.00021 |
| 6 | C | -1.44397 | 0.025322 | -0.00045 |
| 7 | N | -0.58349 | -1.01995 | -0.00022 |
| 6 | C | 0.684989 | -0.6142 | -3E-06 |
| 6 | C | 1.138827 | 0.737907 | 0.000105 |
| 6 | C | 0.134012 | 1.716294 | 0.000025 |
| 7 | N | 2.528666 | 0.786696 | 0.000238 |
| 6 | C | 2.901728 | -0.48179 | 0.000431 |
| 7 | N | 1.83765 | -1.37532 | 0.000115 |
| 1 | H | 1.883573 | -2.38794 | 0.000307 |
| 1 | H | 0.35734 | 2.788048 | 0.000063 |
| 1 | H | 3.932967 | -0.83126 | 0.000586 |
| 6 | C | -2.85155 | -0.3164 | -0.00019 |
| 7 | N | -3.98885 | -0.59574 | 0.000008 |

C8-CHO-PU_9H $E_{tot}$ = -525.019300 a.u.
Gibbs free energy $E_G$= -524.951194 a.u.

| | | | | |
|---|---|---|---|---|
| 7 | N | -2.95537 | 0.495729 | 0.000129 |
| 6 | C | -2.72403 | -0.84238 | 0.000165 |
| 7 | N | -1.54149 | -1.48716 | 0.000132 |
| 6 | C | -0.50713 | -0.63933 | 0.000047 |
| 6 | C | -0.59515 | 0.78785 | -5E-06 |
| 6 | C | -1.89633 | 1.320591 | 0.000029 |
| 7 | N | 0.667682 | 1.353881 | -8.9E-05 |
| 6 | C | 1.498639 | 0.310168 | -0.00012 |
| 7 | N | 0.839811 | -0.9138 | 0.000004 |
| 1 | H | -3.6149 | -1.47778 | 0.000271 |
| 1 | H | 1.292682 | -1.82196 | 0.000126 |
| 1 | H | -2.08569 | 2.399624 | -3E-06 |
| 6 | C | 2.966847 | 0.401155 | -0.00019 |
| 8 | O | 3.689421 | -0.58903 | -0.00011 |
| 1 | H | 3.361121 | 1.443433 | -0.0003 |

C2-CHO-PU_9H $E_{tot}$ = -525.012356 a.u.
Gibbs free energy $E_G$= -524.944891 a.u.

| | | | | |
|---|---|---|---|---|
| 7 | N | 0.996012 | 1.511681 | 0.000007 |
| 6 | C | 1.394739 | 0.212853 | 0.00008 |
| 7 | N | 0.611533 | -0.89063 | 0.000007 |
| 6 | C | -0.68106 | -0.58563 | 0.00001 |
| 6 | C | -1.24452 | 0.727092 | -1E-06 |
| 6 | C | -0.32019 | 1.781704 | -5.3E-05 |
| 7 | N | -2.63364 | 0.663992 | 0.000234 |
| 6 | C | -2.9028 | -0.63159 | -0.0003 |
| 7 | N | -1.77275 | -1.43639 | 0.000118 |
| 1 | H | -1.7371 | -2.44936 | -0.00001 |
| 1 | H | -0.62874 | 2.832534 | 0.000076 |
| 1 | H | -3.90352 | -1.06143 | -0.00037 |
| 6 | C | 2.887993 | 0.024921 | -0.00017 |
| 8 | O | 3.451561 | -1.0542 | 0.000094 |
| 1 | H | 3.443726 | 0.995177 | -0.00039 |

C8-CHO-PU_9H $E_{tot}$ = -525.009688 a.u.
Gibbs free energy $E_G$= -524.94224 a.u

| | | | | |
|---|---|---|---|---|
| 7 | N | 2.852408 | 0.823963 | -4.5E-05 |
| 6 | C | 2.858304 | -0.53441 | 0.000139 |
| 7 | N | 1.805447 | -1.37398 | 0.000027 |
| 6 | C | 0.640616 | -0.71582 | 0.000021 |
| 6 | C | 0.476799 | 0.702668 | -0.00002 |
| 6 | C | 1.667294 | 1.453256 | -0.00013 |
| 7 | N | -0.86196 | 1.044001 | 0.000164 |
| 6 | C | -1.50792 | -0.11952 | -0.00019 |
| 7 | N | -0.63815 | -1.21911 | 0.000095 |
| 7 | H | 3.846157 | -1.00501 | -0.00018 |
| 1 | H | -0.89217 | -2.20108 | -5.2E-05 |
| 1 | H | 1.666127 | 2.548675 | -4.4E-05 |
| 6 | C | -2.97199 | -0.33404 | -0.00024 |
| 8 | O | -3.8042 | 0.554751 | 0.00017 |
| 1 | H | -3.26945 | -1.41754 | -0.00026 |

C2-CHO-PU_9H $E_{tot}$ = -525.011257 a.u.
Gibbs free energy $E_G$= -524.94385 a.u

| | | | | |
|---|---|---|---|---|
| 7 | N | -1.17705 | 1.189913 | 0.000049 |
| 6 | C | -1.36426 | -0.15223 | 0.000121 |
| 7 | N | -0.41313 | -1.12061 | 0.000085 |
| 6 | C | 0.819633 | -0.61971 | 0.000033 |
| 6 | C | 1.163147 | 0.763449 | -6E-06 |
| 6 | C | 0.079273 | 1.657916 | 0.000012 |
| 7 | N | 2.543981 | 0.925254 | -0.00008 |
| 6 | C | 3.019611 | -0.30958 | 0.000051 |
| 7 | N | 2.032898 | -1.28556 | -0.00011 |
| 1 | H | 2.162148 | -2.29073 | 0.000023 |
| 1 | H | 0.219943 | 2.744411 | -5.4E-05 |
| 1 | H | 4.076177 | -0.57302 | 0.000031 |
| 6 | C | -2.77296 | -0.68318 | 0.000093 |
| 8 | O | -3.77783 | 0.002306 | -0.00013 |
| 1 | H | -2.80915 | -1.80211 | -0.00034 |

C8-Cl-PU_9H $E_{tot}$ = -871.372525 a.u.
Gibbs free energy $E_G$= -871.321934 a.u

| | | | | |
|---|---|---|---|---|
| 7 | N | -2.9564 | 0.662746 | 0.000026 |
| 6 | C | -2.85072 | -0.68453 | -0.00016 |
| 7 | N | -1.7248 | -1.43232 | -0.00003 |
| 6 | C | -0.62593 | -0.6783 | -4.3E-05 |
| 6 | C | -0.58024 | 0.744395 | -0.00004 |
| 6 | C | -1.82229 | 1.390895 | 0.000039 |
| 7 | N | 0.742719 | 1.197608 | -0.00026 |
| 6 | C | 1.450794 | 0.092886 | 0.000107 |
| 7 | N | 0.702847 | -1.07678 | -8.3E-05 |
| 1 | H | -3.79307 | -1.24052 | 0.000355 |
| 1 | H | 1.059307 | -2.02521 | 0.000484 |
| 1 | H | -1.91439 | 2.482211 | -5.9E-05 |
| 17 | Cl | 3.1687 | 0.0078 | 0.000131 |

C2-Cl-PU_9H $E_{tot}$ = -871.375960 a.u.
Gibbs free energy $E_G$= -871.325514 a.u

| | | | | |
|---|---|---|---|---|
| 7 | N | -1.06265 | 1.345126 | 0.000155 |
| 6 | C | -1.32641 | 0.0283 | 0.000745 |
| 7 | N | -0.4776 | -1.00924 | 0.00041 |
| 6 | C | 0.794294 | -0.59892 | 0.000268 |
| 6 | C | 1.242963 | 0.752134 | 0.000145 |
| 6 | C | 0.231568 | 1.720825 | -5.3E-05 |
| 7 | N | 2.636168 | 0.80477 | 0.000237 |
| 6 | C | 3.01219 | -0.46034 | 0.000057 |
| 7 | N | 1.946758 | -1.35772 | 0.000168 |
| 1 | H | 1.994174 | -2.36995 | 0.00043 |
| 1 | H | 0.441966 | 2.795162 | -0.00019 |
| 1 | H | 4.043406 | -0.80945 | -1.3E-05 |
| 17 | Cl | -3.02976 | -0.39696 | -0.00082 |

C8-F-PU_9H $E_{tot}$ = -510.943647 a.u.
Gibbs free energy $E_G$= -510.890557 a.u

| | | | | |
|---|---|---|---|---|
| 7 | N | -2.54161 | 0.661651 | 0.000001 |
| 6 | C | -2.43692 | -0.68408 | -0.00017 |
| 7 | N | -1.30939 | -1.43157 | -2.5E-05 |
| 6 | C | -0.21272 | -0.67821 | -2.2E-05 |
| 6 | C | -0.16538 | 0.744952 | -1.4E-05 |
| 6 | C | -1.40627 | 1.390228 | 0.000042 |
| 7 | N | 1.161569 | 1.198929 | -0.00019 |
| 6 | C | 1.850667 | 0.093303 | 0.000113 |
| 7 | N | 1.120378 | -1.07982 | -4.7E-05 |
| 1 | H | -3.37872 | -1.24089 | 0.000281 |
| 1 | H | 1.47934 | -2.0274 | 0.000564 |
| 1 | H | -1.49882 | 2.481461 | -4.9E-05 |
| 9 | F | 3.178369 | 0.016139 | 0.000142 |

C2-F-PU_9H $E_{tot}$ = -510.953511 a.u.
Gibbs free energy $E_G$= -510.900289 a.u

| | | | | |
|---|---|---|---|---|
| 7 | N | -1.57944 | 1.142159 | -0.00014 |
| 6 | C | -1.70193 | -0.18896 | -8.7E-05 |
| 7 | N | -0.77368 | -1.14668 | 0.000026 |
| 6 | C | 0.455979 | -0.61875 | 0.000054 |
| 6 | C | 0.772276 | 0.769349 | -1.2E-05 |
| 6 | C | -0.32563 | 1.638699 | -0.00012 |
| 7 | N | 2.155262 | 0.954766 | 0.000149 |
| 6 | C | 2.650683 | -0.2673 | -2.3E-05 |
| 7 | N | 1.67439 | -1.26324 | 0.000101 |
| 1 | H | 1.818377 | -2.26625 | 0.000405 |
| 1 | H | -0.21602 | 2.72778 | -0.00013 |
| 1 | H | 3.710467 | -0.51716 | -0.00004 |
| 9 | F | -2.97298 | -0.63907 | -7E-06 |

C8-H-PU_9H $E_{tot}$ = -411.741198 a.u.
Gibbs free energy $E_G$= -411.678769 a.u

| | | | | |
|---|---|---|---|---|
| 7 | N | 2.133176 | 0.656495 | 0.000257 |
| 6 | C | 2.020379 | -0.69113 | 0.000211 |
| 7 | N | 0.89314 | -1.43447 | 0.000044 |
| 6 | C | -0.20576 | -0.67547 | -4.3E-05 |
| 6 | C | -0.24493 | 0.747737 | 0.000004 |
| 6 | C | 1.001414 | 1.387556 | 0.000157 |
| 7 | N | -1.56412 | 1.203032 | -9.8E-05 |
| 6 | C | -2.29031 | 0.100127 | -0.0002 |
| 7 | N | -1.53258 | -1.06594 | -0.00028 |
| 1 | H | 1.099483 | 2.478907 | 0.000216 |
| 1 | H | -3.37889 | 0.065738 | -0.00032 |
| 1 | H | 2.960683 | -1.25118 | 0.000252 |
| 1 | H | -1.87332 | -2.02021 | -0.00041 |

C2-H-PU_9H $E_{tot}$ = -411.741198 a.u.
Gibbs free energy $E_G$= -411.678769 a.u

| | | | | |
|---|---|---|---|---|
| 7 | N | 2.133176 | 0.656495 | 0.000257 |
| 6 | C | 2.020379 | -0.69113 | 0.000211 |
| 7 | N | 0.89314 | -1.43447 | 0.000044 |
| 6 | C | -0.20576 | -0.67547 | -4.3E-05 |
| 6 | C | -0.24493 | 0.747737 | 0.000004 |
| 6 | C | 1.001414 | 1.387556 | 0.000157 |
| 7 | N | -1.56412 | 1.203032 | -9.8E-05 |
| 6 | C | -2.29031 | 0.100127 | -0.0002 |
| 7 | N | -1.53258 | -1.06594 | -0.00028 |
| 1 | H | 1.099483 | 2.478907 | 0.000216 |
| 1 | H | -3.37889 | 0.065738 | -0.00032 |
| 1 | H | 2.960683 | -1.25118 | 0.000252 |
| 1 | H | -1.87332 | -2.02021 | -0.00041 |

C8-Me-PU_9H $E_{tot}$ = -451.043994 a.u.
Gibbs free energy $E_G$= -450.957287 a.u

| | | | | |
|---|---|---|---|---|
| 7 | N | 2.586578 | 0.669494 | -0.00031 |
| 6 | C | 2.48465 | -0.67772 | 0.000005 |
| 7 | N | 1.360823 | -1.42888 | -5.6E-05 |
| 6 | C | 0.257082 | -0.67899 | 0.000052 |
| 6 | C | 0.206609 | 0.743328 | 0.000009 |
| 6 | C | 1.44716 | 1.391772 | -0.00024 |
| 7 | N | -1.11641 | 1.18448 | 0.000493 |
| 6 | C | -1.84841 | 0.079977 | -1.6E-05 |
| 7 | N | -1.06677 | -1.07812 | 0.00014 |
| 1 | H | 3.428843 | -1.23112 | -0.00077 |
| 1 | H | -1.3966 | -2.03604 | -0.00036 |
| 1 | H | 1.536806 | 2.483653 | -0.00014 |
| 6 | C | -3.33922 | 0.026244 | 0.000051 |
| 1 | H | -3.71821 | -0.50229 | -0.89159 |
| 1 | H | -3.7181 | -0.50265 | 0.891521 |
| 1 | H | -3.72954 | 1.051921 | 0.000275 |

C2-Me- PU _9H $E_{tot}$ = -451.041421 a.u.
Gibbs free energy $E_G$= -450.95599 a.u

| | | | | |
|---|---|---|---|---|
| 7 | N | 1.501101 | 1.193626 | 0.000014 |
| 6 | C | 1.709726 | -0.1518 | 0.000021 |
| 7 | N | 0.755725 | -1.11289 | 0.00002 |
| 6 | C | -0.4826 | -0.6139 | 0.000006 |
| 6 | C | -0.838 | 0.761681 | 0.000002 |
| 6 | C | 0.240404 | 1.656825 | 0.000009 |
| 7 | N | -2.2255 | 0.912782 | -1.6E-05 |
| 6 | C | -2.68894 | -0.32362 | -9E-06 |
| 7 | N | -1.68882 | -1.29108 | -1.1E-05 |
| 1 | H | -1.80631 | -2.29733 | 0.000084 |
| 1 | H | 0.097707 | 2.743341 | -7E-06 |
| 1 | H | -3.74231 | -0.59971 | -2.3E-05 |
| 6 | C | 3.145628 | -0.6045 | -2.3E-05 |
| 1 | H | 3.66708 | -0.20529 | 0.885218 |
| 1 | H | 3.202129 | -1.70046 | 0.000281 |
| 1 | H | 3.666852 | -0.20583 | -0.88564 |

C8-OMe- PU _9H $E_{tot}$ = -526.215871 a.u.
Gibbs free energy $E_G$= -526.126105 a.u

| | | | | |
|---|---|---|---|---|
| 7 | N | 3.041032 | 0.41492 | -0.00032 |
| 6 | C | 2.767354 | -0.90512 | -0.0003 |
| 7 | N | 1.551127 | -1.50177 | -0.00017 |
| 6 | C | 0.561001 | -0.61391 | -0.00006 |
| 6 | C | 0.690752 | 0.803172 | -9.3E-05 |
| 6 | C | 2.005484 | 1.282605 | -0.00023 |
| 7 | N | -0.56164 | 1.417619 | 0.000011 |
| 6 | C | -1.41414 | 0.41293 | 0.000099 |
| 7 | N | -0.81005 | -0.84747 | 0.000187 |
| 1 | H | 3.629598 | -1.57872 | -0.0004 |
| 1 | H | -1.24985 | -1.75859 | -0.00035 |
| 1 | H | 2.237825 | 2.352865 | -0.00024 |
| 8 | O | -2.74274 | 0.595497 | 0.000239 |
| 6 | C | -3.57752 | -0.57874 | 0.000491 |
| 1 | H | -4.60733 | -0.20347 | 0.000716 |
| 1 | H | -3.40481 | -1.18244 | -0.90731 |
| 1 | H | -3.40436 | -1.18237 | 0.908252 |

C2-OMe- PU _9H $E_{tot}$ = -526.226856 a.u.
Gibbs free energy $E_G$= -526.136473 a.u

| | | | | |
|---|---|---|---|---|
| 7 | N | 1.168668 | 1.068913 | -6.5E-05 |
| 6 | C | 1.275745 | -0.2798 | -0.00012 |
| 7 | N | 0.284448 | -1.19685 | -3.6E-05 |
| 6 | C | -0.91758 | -0.62342 | 0.000004 |
| 6 | C | -1.19279 | 0.774687 | -6E-06 |
| 6 | C | -0.06747 | 1.603708 | -2.4E-05 |
| 7 | N | -2.57101 | 1.00516 | 0.000061 |
| 6 | C | -3.10471 | -0.20005 | -2.1E-05 |
| 7 | N | -2.16023 | -1.22712 | 0.000087 |
| 1 | H | -2.33601 | -2.22472 | -0.00017 |
| 1 | H | -0.14269 | 2.696396 | 0.000012 |
| 1 | H | -4.17195 | -0.4161 | 0 |
| 8 | O | 2.511448 | -0.82765 | -0.00001 |
| 6 | C | 3.632839 | 0.082009 | 0.000094 |
| 1 | H | 4.517229 | -0.56786 | 0.000195 |
| 1 | H | 3.616295 | 0.719944 | -0.89652 |
| 1 | H | 3.616113 | 0.719975 | 0.896681 |

C8-OMe- PU _9H $E_{tot}$ = -526.224915 a.u.
Gibbs free energy $E_G$= -526.134812 a.u

| | | | | |
|---|---|---|---|---|
| 7 | N | -2.86968 | 0.912592 | 0.000064 |
| 6 | C | -2.94804 | -0.4322 | 0.000287 |
| 7 | N | -1.92971 | -1.32642 | 0.000154 |
| 6 | C | -0.74071 | -0.73074 | 0.000052 |
| 6 | C | -0.50013 | 0.67421 | -2.9E-05 |
| 6 | C | -1.6418 | 1.479295 | -6.9E-05 |
| 7 | N | 0.873437 | 0.940904 | -4E-06 |
| 6 | C | 1.427273 | -0.25722 | -0.00012 |
| 7 | N | 0.521473 | -1.31044 | -0.00013 |
| 1 | H | -3.95592 | -0.85846 | 0.000077 |
| 1 | H | 0.74228 | -2.29874 | 0.000327 |
| 1 | H | -1.58693 | 2.57328 | -0.00012 |
| 8 | O | 2.728439 | -0.55702 | -0.00014 |
| 6 | C | 3.618428 | 0.59254 | -6.2E-05 |
| 1 | H | 4.627738 | 0.165268 | -5.8E-05 |
| 6 | H | 3.44324 | 1.201568 | -0.89887 |
| 1 | H | 3.443196 | 1.201483 | 0.898792 |

C2-OMe- PU _9H $E_{tot}$ = -526.226415 a.u.
Gibbs free energy $E_G$= -526.135919 a.u

| | | | | |
|---|---|---|---|---|
| 7 | N | 0.872466 | 1.628833 | 0.000001 |
| 6 | C | 1.333519 | 0.353883 | 0.000077 |
| 7 | N | 0.623972 | -0.79271 | 0.000094 |
| 6 | C | -0.69405 | -0.56333 | -8.3E-05 |
| 6 | C | -1.32554 | 0.707604 | -0.00024 |
| 6 | C | -0.45574 | 1.809613 | -0.00017 |
| 7 | N | -2.71492 | 0.567124 | -0.00038 |
| 6 | C | -2.91401 | -0.7363 | -0.00043 |
| 7 | N | -1.73013 | -1.47584 | -0.00012 |
| 1 | H | -1.63686 | -2.48443 | -0.00029 |
| 1 | H | -0.82233 | 2.842041 | -0.00024 |
| 1 | H | -3.88586 | -1.22703 | -0.00053 |
| 8 | O | 2.683112 | 0.280653 | 0.000385 |
| 6 | C | 3.264953 | -1.03855 | 0.000634 |
| 1 | H | 4.34868 | -0.86755 | 0.000862 |
| 1 | H | 2.958295 | -1.59875 | 0.897695 |
| 1 | H | 2.958697 | -1.5989 | -0.89647 |

C8-OH- PU _9H  $E_{tot}$ = -486.938320 a.u.
Gibbs free energy  $E_G$= -486.874055 a.u

| | | | | |
|---|---|---|---|---|
| 7 | N | 2.556834 | 0.652548 | 0.000001 |
| 6 | C | 2.448037 | -0.69047 | -0.00034 |
| 7 | N | 1.314215 | -1.43286 | -0.00013 |
| 6 | C | 0.222506 | -0.67438 | -4.2E-05 |
| 6 | C | 0.177412 | 0.747923 | 0.00003 |
| 6 | C | 1.421781 | 1.385941 | 0.000135 |
| 7 | N | -1.14321 | 1.204335 | -0.00012 |
| 6 | C | -1.86154 | 0.105471 | 0.000092 |
| 7 | N | -1.1112 | -1.07242 | 0.000188 |
| 1 | H | 3.386456 | -1.25309 | 0.000137 |
| 1 | H | -1.43955 | -2.03111 | -0.00044 |
| 1 | H | 1.520011 | 2.476661 | 0.000157 |
| 8 | O | -3.20981 | 0.1117 | 0.00009 |
| 1 | H | -3.55413 | -0.79418 | 0.000582 |

C8-OH- PU _9H  $E_{tot}$ = -486.948145 a.u.
Gibbs free energy  $E_G$= -486.88269 a.u

| | | | | |
|---|---|---|---|---|
| 7 | N | -2.54592 | 0.681927 | 0.000005 |
| 6 | C | -2.4566 | -0.66242 | 0.000187 |
| 7 | N | -1.33427 | -1.42171 | 0.000108 |
| 6 | C | -0.22992 | -0.68212 | 0.000033 |
| 6 | C | -0.16568 | 0.741396 | -4.5E-05 |
| 6 | C | -1.39951 | 1.397867 | -0.0001 |
| 7 | N | 1.165611 | 1.17337 | -3.8E-05 |
| 6 | C | 1.858696 | 0.053357 | -6.1E-05 |
| 7 | N | 1.097047 | -1.10153 | -7.2E-05 |
| 1 | H | -3.40334 | -1.21098 | 0.000062 |
| 1 | H | 1.438809 | -2.05497 | 0.000415 |
| 1 | H | -1.482 | 2.489994 | -0.00016 |
| 8 | O | 3.197102 | -0.06042 | -5.2E-05 |
| 1 | H | 3.550537 | 0.846452 | -1.3E-05 |

C8-$NH_2$- PU _9H  $E_{tot}$ = -467.086682 a.u.
Gibbs free energy  $E_G$= -467.009632 a.u

| | | | | |
|---|---|---|---|---|
| 7 | N | -2.5647 | 0.671329 | 0.003387 |
| 6 | C | -2.4689 | -0.67129 | 0.000119 |
| 7 | N | -1.34113 | -1.42528 | 0.000245 |
| 6 | C | -0.24124 | -0.67898 | -0.00105 |
| 6 | C | -0.18249 | 0.744548 | -0.00091 |
| 6 | C | -1.42076 | 1.393514 | 0.003496 |
| 7 | N | 1.141784 | 1.183367 | 0.007028 |
| 6 | C | 1.858892 | 0.069586 | 0.000442 |
| 7 | N | 1.08603 | -1.09103 | 0.0028 |
| 1 | H | -3.41215 | -1.22594 | -0.00337 |
| 1 | H | 1.409753 | -2.04766 | -0.07306 |
| 1 | H | -1.50978 | 2.485132 | 0.00697 |
| 7 | N | 3.233981 | 0.013406 | -0.07985 |
| 1 | H | 3.664291 | 0.91868 | 0.085432 |
| 1 | H | 3.683126 | -0.73705 | 0.436153 |

C2-OH- PU _9H  $E_{tot}$ = -486.953186 a.u.
Gibbs free energy  $E_G$= -486.887437 a.u

| | | | | |
|---|---|---|---|---|
| 7 | N | 1.5719 | 1.124812 | 0.000317 |
| 6 | C | 1.708047 | -0.2241 | 0.000646 |
| 7 | N | 0.749558 | -1.1652 | 0.000287 |
| 6 | C | -0.4697 | -0.62033 | 0.000015 |
| 6 | C | -0.77877 | 0.769144 | -5.5E-05 |
| 6 | C | 0.325084 | 1.629853 | 0.000072 |
| 7 | N | -2.16144 | 0.965362 | -0.00026 |
| 6 | C | -2.66581 | -0.25257 | -0.00044 |
| 7 | N | -1.69488 | -1.25505 | -0.00024 |
| 1 | H | -1.8448 | -2.25699 | -0.00053 |
| 1 | H | 0.222217 | 2.719926 | 0 |
| 1 | H | -3.72708 | -0.49541 | -0.00063 |
| 8 | O | 2.978844 | -0.69523 | -8.6E-05 |
| 1 | H | 3.549791 | 0.092881 | -0.00039 |

C2-OH- PU _9H  $E_{tot}$ = -486.951744 a.u.
Gibbs free energy  $E_G$= -486.88609 a.u

| | | | | |
|---|---|---|---|---|
| 7 | N | 1.557025 | 1.185213 | 0.000152 |
| 6 | C | 1.723573 | -0.15349 | 0.000351 |
| 7 | N | 0.783402 | -1.12223 | 0.000195 |
| 6 | C | -0.45352 | -0.61545 | 0.000019 |
| 6 | C | -0.79016 | 0.765769 | -0.00008 |
| 6 | C | 0.296236 | 1.650934 | -1.7E-05 |
| 7 | N | -2.17706 | 0.93219 | -0.00024 |
| 6 | C | -2.65639 | -0.29529 | -0.00025 |
| 7 | N | -1.66361 | -1.27771 | -8.4E-05 |
| 1 | H | -1.79427 | -2.28217 | -0.00036 |
| 1 | H | 0.163626 | 2.738145 | -0.0001 |
| 1 | H | -3.71213 | -0.56114 | -0.00033 |
| 8 | O | 3.01653 | -0.56217 | 0.000062 |
| 1 | H | 2.993732 | -1.53456 | -3.7E-05 |

C2-$NH_2$- PU _9H  $E_{tot}$ = -467.093074 a.u.
Gibbs free energy  $E_G$= -467.016016 a.u

| | | | | |
|---|---|---|---|---|
| 7 | N | 1.54184 | 1.176136 | 0.00488 |
| 6 | C | 1.717681 | -0.17729 | -0.00287 |
| 7 | N | 0.759091 | -1.13752 | 0.004223 |
| 6 | C | -0.4694 | -0.6174 | -0.00073 |
| 6 | C | -0.80438 | 0.764518 | -0.00411 |
| 6 | C | 0.285626 | 1.645634 | 0.003742 |
| 7 | N | -2.19101 | 0.936357 | -0.0026 |
| 6 | C | -2.67479 | -0.28956 | 0.000545 |
| 7 | N | -1.68436 | -1.27473 | 0.003782 |
| 1 | H | -1.81706 | -2.27877 | 0.002638 |
| 1 | H | 0.155556 | 2.733523 | 0.013831 |
| 1 | H | -3.73146 | -0.55191 | 0.001158 |
| 7 | N | 3.022212 | -0.60817 | -0.0601 |
| 1 | H | 3.732152 | 0.07314 | 0.174947 |
| 1 | H | 3.198 | -1.5759 | 0.176695 |

C8-NO$_2$-PU_7H E$_{tot}$ = -616.162730 a.u.
Gibbs free energy E$_G$= -616.103174 a.u.

| | | | | |
|---|---|---|---|---|
| 7 | N | 3.216658 | 0.647833 | 0.000053 |
| 6 | C | 3.097687 | -0.70874 | -0.00017 |
| 7 | N | 1.979247 | -1.44724 | 0.000043 |
| 6 | C | 0.85242 | -0.71574 | 0.000089 |
| 6 | C | 0.861159 | 0.715935 | 0.00009 |
| 6 | C | 2.094873 | 1.379461 | 0.000087 |
| 7 | N | -0.472 | 1.082893 | 0.000098 |
| 6 | C | -1.17701 | -0.09881 | -8E-06 |
| 7 | N | -0.45102 | -1.19536 | 0.000195 |
| 1 | H | 4.042441 | -1.26069 | 0.000378 |
| 1 | H | -0.89882 | 2.003292 | 0.000202 |
| 1 | H | 2.193231 | 2.470913 | 0.000108 |
| 7 | N | -2.64341 | -0.0421 | -0.00013 |
| 8 | O | -3.12865 | 1.101536 | -0.00022 |
| 8 | O | -3.2611 | -1.09759 | -0.00016 |

C8-CN-PU_7H E$_{tot}$ = -503.921405 a.u.
Gibbs free energy E$_G$= -503.864055 a.u.

| | | | | |
|---|---|---|---|---|
| 7 | N | 2.848838 | 0.655869 | 0.000043 |
| 6 | C | 2.733274 | -0.69931 | -0.00021 |
| 7 | N | 1.615239 | -1.4401 | 0.000021 |
| 6 | C | 0.4859 | -0.71206 | 0.00006 |
| 6 | C | 0.491673 | 0.716993 | 0.000069 |
| 6 | C | 1.723033 | 1.383299 | 0.00008 |
| 7 | N | -0.83964 | 1.083723 | 0.000116 |
| 6 | C | -1.56935 | -0.10173 | -0.00022 |
| 7 | N | -0.81321 | -1.19538 | 0.000269 |
| 1 | H | 3.678579 | -1.25044 | 0.0004 |
| 1 | H | -1.23008 | 2.01924 | 0.000329 |
| 1 | H | 1.818583 | 2.475154 | 0.000137 |
| 6 | C | -2.99562 | -0.07756 | -0.00024 |
| 7 | N | -4.16559 | -0.00436 | -0.00018 |

C8-CHO-PU_7H E$_{tot}$ = -525.013652 a.u.
Gibbs free energy E$_G$= -524.945777 a.u.

| | | | | |
|---|---|---|---|---|
| 7 | N | 2.844098 | 0.815538 | -0.00002 |
| 6 | C | 2.845361 | -0.54703 | 0.000019 |
| 7 | N | 1.797586 | -1.38134 | 0.000093 |
| 6 | C | 0.606663 | -0.75388 | 0.000023 |
| 6 | C | 0.488809 | 0.674017 | -7.3E-05 |
| 6 | C | 1.660753 | 1.442802 | -8.4E-05 |
| 7 | N | -0.86599 | 0.922584 | -0.00014 |
| 6 | C | -1.49474 | -0.31131 | -0.00014 |
| 7 | N | -0.64561 | -1.34004 | 0.000095 |
| 1 | H | 3.835943 | -1.01228 | 0.000217 |
| 1 | H | -1.35651 | 1.810795 | -0.00021 |
| 1 | H | 1.661576 | 2.539006 | -0.00016 |
| 6 | C | -2.96458 | -0.42086 | -0.00023 |
| 8 | O | -3.69438 | 0.563568 | 0.00049 |
| 1 | H | -3.35028 | -1.46565 | -0.00102 |

C2-NO$_2$-AD_7H E$_{tot}$ = -616.164390 a.u.
Gibbs free energy E$_G$= -616.103789 a.u

| | | | | |
|---|---|---|---|---|
| 7 | N | -0.778286 | 1.394437 | 0.00003 |
| 6 | C | -1.05578 | 0.085755 | -0.000039 |
| 7 | N | -0.253008 | -0.97154 | -0.00004 |
| 6 | C | 1.046685 | -0.636799 | 0.000055 |
| 6 | C | 1.483745 | 0.727106 | 0.000138 |
| 6 | C | 0.520629 | 1.739294 | 0.000137 |
| 7 | N | 2.863665 | 0.651672 | 0.000196 |
| 6 | C | 3.185962 | -0.697903 | 0.000395 |
| 7 | N | 2.134251 | -1.497187 | -0.000083 |
| 1 | H | 3.519211 | 1.42408 | 0.000131 |
| 1 | H | 0.758995 | 2.808366 | 0.000226 |
| 1 | H | 4.223454 | -1.028278 | 0.000485 |
| 7 | N | -2.55264 | -0.245193 | -0.00024 |
| 8 | O | -2.855151 | -1.434027 | 0.000455 |
| 8 | O | -3.330722 | 0.70475 | -0.000955 |

C2-CN-PU_7H E$_{tot}$ = -503.922890 a.u.
Gibbs free energy E$_G$= -503.865575 a.u.

| | | | | |
|---|---|---|---|---|
| 7 | N | -1.160143 | 1.346391 | -0.000178 |
| 6 | C | -1.444089 | 0.016587 | -0.000381 |
| 7 | N | -0.594212 | -1.028503 | -0.000187 |
| 6 | C | 0.696014 | -0.666001 | 0.000006 |
| 6 | C | 1.109813 | 0.703521 | 0.000097 |
| 6 | C | 0.132959 | 1.702128 | 0.000025 |
| 7 | N | 2.491962 | 0.65321 | 0.000302 |
| 6 | C | 2.837914 | -0.689868 | 0.000239 |
| 7 | N | 1.800191 | -1.50749 | 0.000192 |
| 1 | H | 3.133293 | 1.437443 | 0.000332 |
| 1 | H | 0.360448 | 2.773896 | 0.00011 |
| 1 | H | 3.881128 | -1.002523 | 0.00037 |
| 6 | C | -2.857071 | -0.310695 | -0.000199 |
| 7 | N | -3.998955 | -0.569728 | -0.000062 |

C2-CHO-PU_7H E$_{tot}$ = -525.005733 a.u.
Gibbs free energy E$_G$= -524.938678 a.u.

| | | | | |
|---|---|---|---|---|
| 7 | N | 0.995362 | 1.507603 | -0.000148 |
| 6 | C | 1.394479 | 0.205626 | -0.000486 |
| 7 | N | 0.621999 | -0.897894 | -0.000252 |
| 6 | C | -0.689202 | -0.639235 | -0.000099 |
| 6 | C | -1.214014 | 0.694329 | 0.000061 |
| 6 | C | -0.320136 | 1.767499 | 0.000069 |
| 7 | N | -2.586981 | 0.534154 | 0.000245 |
| 6 | C | -2.824898 | -0.834041 | -0.000229 |
| 7 | N | -1.727345 | -1.566231 | 0.000215 |
| 1 | H | -3.288825 | 1.264642 | 0.000585 |
| 1 | H | -0.633932 | 2.817808 | 0.000191 |
| 1 | H | -3.840713 | -1.227231 | -0.000195 |
| 6 | C | 2.892859 | 0.034436 | -0.000026 |
| 8 | O | 3.471382 | -1.035354 | 0.000324 |
| 1 | H | 3.436639 | 1.0125 | 0.000662 |

C8-CHO-PU_7H $E_{tot}$ = -525.003418 a.u.

Gibbs free energy $E_G$= -524.936268 a.u.

| | | | | |
|---|---|---|---|---|
| 7 | N | 2.970464 | 0.487118 | -3.9E-05 |
| 6 | C | 2.733412 | -0.85547 | -7.9E-05 |
| 7 | N | 1.557347 | -1.49514 | 0.000024 |
| 6 | C | 0.49196 | -0.67113 | 0.000035 |
| 6 | C | 0.62689 | 0.754518 | 0.000004 |
| 6 | C | 1.913817 | 1.309395 | -1.5E-05 |
| 7 | N | -0.66428 | 1.233197 | -5.6E-05 |
| 6 | C | -1.50578 | 0.118192 | 0.000083 |
| 7 | N | -0.83993 | -1.03338 | 0.000126 |
| 1 | H | 3.628102 | -1.48576 | 0.000108 |
| 1 | H | -0.95993 | 2.203261 | -7.7E-05 |
| 1 | H | 2.105104 | 2.389038 | -6.9E-05 |
| 6 | C | -2.97445 | 0.313981 | 0.000114 |
| 8 | O | -3.79627 | -0.58263 | -0.00022 |
| 1 | H | -3.2834 | 1.395086 | 0.000574 |

C2-CHO-PU_7H $E_{tot}$ = -525.006594 a.u.

Gibbs free energy $E_G$= -524.939398 a.u.

| | | | | |
|---|---|---|---|---|
| 7 | N | -1.17644 | 1.188475 | 0.000053 |
| 6 | C | -1.364897 | -0.157068 | 0.00009 |
| 7 | N | -0.426089 | -1.127526 | 0.000068 |
| 6 | C | 0.832802 | -0.671024 | 0.000015 |
| 6 | C | 1.137149 | 0.725565 | -0.000004 |
| 6 | C | 0.081318 | 1.644597 | 0.000023 |
| 7 | N | 2.518348 | 0.785624 | 0.000006 |
| 6 | C | 2.971463 | -0.526164 | -0.000146 |
| 7 | N | 2.002426 | -1.423244 | 0.000059 |
| 1 | H | 3.094647 | 1.618805 | -0.000051 |
| 1 | H | 0.228816 | 2.731216 | 0.000015 |
| 1 | H | 4.036571 | -0.753492 | -0.00019 |
| 6 | C | -2.780711 | -0.674207 | 0.000101 |
| 8 | O | -3.778009 | 0.022746 | -0.000162 |
| 1 | H | -2.826422 | -1.791997 | -0.000252 |

C8-Cl-PU_7H $E_{tot}$ = -871.367421 a.u.

Gibbs free energy $E_G$= -871.31708 a.u.

| | | | | |
|---|---|---|---|---|
| 7 | N | 2.968099 | 0.639871 | 0.000137 |
| 6 | C | 2.839325 | -0.70945 | 0.000338 |
| 7 | N | 1.71181 | -1.44255 | 0.000045 |
| 6 | C | 0.591451 | -0.70601 | -1.1E-05 |
| 6 | C | 0.611177 | 0.721789 | 0.000021 |
| 6 | C | 1.842561 | 1.376751 | 0.000053 |
| 7 | N | -0.72887 | 1.094856 | -9.4E-05 |
| 6 | C | -1.44846 | -0.08588 | 0.000033 |
| 7 | N | -0.71949 | -1.17912 | -0.00023 |
| 1 | H | 3.778351 | -1.2718 | -0.00018 |
| 1 | H | -1.12337 | 2.027582 | 0.000034 |
| 1 | H | 1.946895 | 2.467893 | 0.000031 |
| 17 | Cl | -3.16701 | -0.03519 | -8.6E-05 |

C2-Cl-PU_7H $E_{tot}$ = -871.370729 a.u.

Gibbs free energy $E_G$= -871.320551 a.u.

| | | | | |
|---|---|---|---|---|
| 7 | N | -1.061812 | 1.342753 | -0.00007 |
| 6 | C | -1.326318 | 0.021505 | -0.00012 |
| 7 | N | -0.488964 | -1.015364 | -0.00004 |
| 6 | C | 0.805032 | -0.650891 | 0.00003 |
| 6 | C | 1.215678 | 0.717073 | 0.000034 |
| 6 | C | 0.232771 | 1.706831 | 0.000009 |
| 7 | N | 2.601478 | 0.668536 | 0.000088 |
| 6 | C | 2.947112 | -0.671574 | 0.000181 |
| 7 | N | 1.908524 | -1.49136 | 0.000054 |
| 1 | H | 3.242133 | 1.452793 | -0.00000 |
| 1 | H | 0.448982 | 2.780886 | 0.000011 |
| 1 | H | 3.990266 | -0.984774 | 0.000248 |
| 17 | Cl | -3.037742 | -0.383443 | -0.00008 |

C8-F-PU_7H $E_{tot}$ = -510.938689 a.u.

Gibbs free energy $E_G$= -510.885856 a.u.

| | | | | |
|---|---|---|---|---|
| 7 | N | 2.550567 | 0.650149 | 0.000117 |
| 6 | C | 2.428655 | -0.69817 | 0.000303 |
| 7 | N | 1.30401 | -1.43792 | 0.000031 |
| 6 | C | 0.180803 | -0.70903 | -0.00002 |
| 6 | C | 0.193978 | 0.719616 | 0.000009 |
| 6 | C | 1.41938 | 1.380996 | 0.000027 |
| 7 | N | -1.15332 | 1.086933 | -8.5E-05 |
| 6 | C | -1.84741 | -0.10128 | 0.000005 |
| 7 | N | -1.1299 | -1.19143 | -0.00021 |
| 1 | H | 3.3701 | -1.25653 | -0.00017 |
| 1 | H | -1.5566 | 2.015961 | 0.00007 |
| 1 | H | 1.517268 | 2.472754 | 0.00001 |
| 9 | F | -3.17586 | -0.0599 | -9.4E-05 |

C2-F-PU_7H $E_{tot}$ = -510.948485 a.u.

Gibbs free energy $E_G$= -510.895513 a.u.

| | | | | |
|---|---|---|---|---|
| 7 | N | -1.574291 | 1.148209 | -0.000048 |
| 6 | C | -1.703512 | -0.186637 | -0.000063 |
| 7 | N | -0.790435 | -1.149075 | -0.000019 |
| 6 | C | 0.466944 | -0.669475 | 0.000018 |
| 6 | C | 0.750479 | 0.731072 | 0.000006 |
| 6 | C | -0.317041 | 1.628546 | -0.000006 |
| 7 | N | 2.136255 | 0.80843 | 0.000022 |
| 6 | C | 2.601586 | -0.49328 | 0.000164 |
| 7 | N | 1.64132 | -1.404863 | 0.000005 |
| 1 | H | 2.703188 | 1.647398 | -0.000111 |
| 1 | H | -0.197769 | 2.717459 | -0.000019 |
| 1 | H | 3.668793 | -0.710914 | 0.000218 |
| 9 | F | -2.983876 | -0.614911 | -0.000057 |

C8-H-AD_7H $E_{tot}$ = -411.735954 a.u.
Gibbs free energy $E_G$= -411.673762 a.u.

| | | | | |
|---|---|---|---|---|
| 7 | N | -2.12563 | 0.684039 | -0.00026 |
| 6 | C | -2.0303 | -0.66959 | -9.7E-05 |
| 7 | N | -0.92259 | -1.42993 | 0.000027 |
| 6 | C | 0.219001 | -0.72192 | 0.000092 |
| 6 | C | 0.234645 | 0.706284 | -5.1E-05 |
| 6 | C | -0.98209 | 1.390693 | -0.00022 |
| 7 | N | 1.579429 | 1.045547 | 0.000083 |
| 6 | C | 2.285114 | -0.14627 | 0.000431 |
| 7 | N | 1.516735 | -1.22169 | 0.000003 |
| 1 | H | -1.0606 | 2.484487 | -0.0003 |
| 1 | H | 3.374333 | -0.15507 | 0.000616 |
| 1 | H | -2.98376 | -1.20761 | -0.00027 |
| 1 | H | 1.97617 | 1.977259 | 0.000072 |

C2-H-AD_7H $E_{tot}$ = -411.735954 a.u.
Gibbs free energy $E_G$= -411.673762 a.u.

| | | | | |
|---|---|---|---|---|
| 7 | N | -2.12563 | 0.684039 | -0.00026 |
| 6 | C | -2.0303 | -0.66959 | -9.7E-05 |
| 7 | N | -0.92259 | -1.42993 | 0.000027 |
| 6 | C | 0.219001 | -0.72192 | 0.000092 |
| 6 | C | 0.234645 | 0.706284 | -5.1E-05 |
| 6 | C | -0.98209 | 1.390693 | -0.00022 |
| 7 | N | 1.579429 | 1.045547 | 0.000083 |
| 6 | C | 2.285114 | -0.14627 | 0.000431 |
| 7 | N | 1.516735 | -1.22169 | 0.000003 |
| 1 | N | -1.0606 | 2.484487 | -0.0003 |
| 1 | H | 3.374333 | -0.15507 | 0.000616 |
| 1 | H | -2.98376 | -1.20761 | -0.00027 |
| 1 | H | 1.97617 | 1.977259 | 0.000072 |

C8-Me-PU_7H $E_{tot}$ = -451.038815 a.u.
Gibbs free energy $E_G$= -450.952769 a.u.

| | | | | |
|---|---|---|---|---|
| 7 | N | -2.60006 | 0.641637 | -0.00019 |
| 6 | C | -2.47222 | -0.70748 | -0.00048 |
| 7 | N | -1.3452 | -1.44164 | -9.1E-05 |
| 6 | C | -0.22117 | -0.70756 | 0.000014 |
| 6 | C | -0.23995 | 0.720219 | 0.000001 |
| 6 | C | -1.4709 | 1.375028 | -6.3E-05 |
| 7 | N | 1.097406 | 1.088611 | 0.000175 |
| 6 | C | 1.845648 | -0.08439 | -1.3E-05 |
| 7 | N | 1.087752 | -1.17354 | 0.000384 |
| 1 | H | -3.41207 | -1.269 | 0.000219 |
| 1 | H | 1.470007 | 2.030214 | 0.000098 |
| 1 | H | -1.57472 | 2.466668 | -6E-06 |
| 6 | C | 3.337952 | -0.06806 | 0.000099 |
| 1 | H | 3.729059 | 0.451038 | -0.89175 |
| 1 | H | 3.728928 | 0.451445 | 0.891764 |
| 1 | H | 3.703342 | -1.10245 | 0.000358 |

C2-Me-PU_7H $E_{tot}$ = -451.035984 a.u.
Gibbs free energy $E_G$= -450.95092 a.u.

| | | | | |
|---|---|---|---|---|
| 7 | N | 1.496747 | 1.199555 | -0.000042 |
| 6 | C | 1.712007 | -0.148928 | -0.000046 |
| 7 | N | 0.772433 | -1.115108 | -0.000032 |
| 6 | C | -0.492567 | -0.665247 | 0.000012 |
| 6 | C | -0.814002 | 0.723426 | 0.000034 |
| 6 | C | 0.233029 | 1.646748 | -0.00002 |
| 7 | N | -2.201237 | 0.768519 | -0.000057 |
| 6 | C | -2.635626 | -0.545832 | 0.000002 |
| 7 | N | -1.653773 | -1.431727 | -0.000004 |
| 1 | H | -2.787769 | 1.593873 | 0.000521 |
| 1 | H | 0.079495 | 2.732811 | -0.00002 |
| 1 | H | -3.697526 | -0.788367 | -0.000019 |
| 6 | C | 3.154414 | -0.584359 | 0.000041 |
| 1 | H | 3.671682 | -0.181647 | 0.886251 |
| 1 | H | 3.219264 | -1.679614 | -0.000518 |
| 1 | H | 3.672135 | -0.180589 | -0.885407 |

C8-OMe- PU _7H $E_{tot}$ = -526.220894 a.u.
Gibbs free energy $E_G$= -526.131069 a.u.

| | | | | |
|---|---|---|---|---|
| 7 | N | -3.05633 | 0.368489 | -0.00012 |
| 6 | C | -2.74174 | -0.94521 | 0.000067 |
| 7 | N | -1.52014 | -1.51663 | -0.00001 |
| 6 | C | -0.5119 | -0.63535 | -5E-06 |
| 6 | C | -0.73081 | 0.778008 | -4.6E-05 |
| 6 | C | -2.03673 | 1.25438 | -0.00012 |
| 7 | N | 0.546612 | 1.33624 | 0.000037 |
| 6 | C | 1.427135 | 0.268626 | 0.000099 |
| 7 | N | 0.84993 | -0.92142 | -6E-06 |
| 1 | H | -3.59202 | -1.63497 | -0.00031 |
| 1 | H | 0.80651 | 2.314456 | -0.00015 |
| 1 | H | -2.29063 | 2.320772 | -0.00017 |
| 8 | O | 2.733313 | 0.537797 | 0.000105 |
| 6 | C | 3.599356 | -0.63222 | 0.000065 |
| 1 | H | 4.617222 | -0.22576 | 0.000075 |
| 1 | H | 3.410018 | -1.23656 | 0.898774 |
| 1 | H | 3.410015 | -1.23651 | -0.89868 |

C2-OMe- PU _7H $E_{tot}$ = -526.222037 a.u.
Gibbs free energy $E_G$= -526.131784 a.u.

| | | | | |
|---|---|---|---|---|
| 7 | N | -0.87114 | 1.628906 | -0.000144 |
| 6 | C | -1.33506 | 0.350464 | 0.000054 |
| 7 | N | -0.636292 | -0.794465 | 0.000141 |
| 6 | C | 0.696294 | -0.614405 | 0.00011 |
| 6 | C | 1.293736 | 0.678106 | -0.000038 |
| 6 | C | 0.456547 | 1.797655 | -0.000174 |
| 7 | N | 2.663003 | 0.436389 | 0.00004 |
| 6 | C | 2.817884 | -0.935884 | 0.000154 |
| 7 | N | 1.671908 | -1.601882 | 0.000246 |
| 1 | H | 3.406517 | 1.123348 | -0.00012 |
| 1 | H | 0.829316 | 2.828901 | -0.000284 |
| 1 | H | 3.80622 | -1.393335 | 0.000213 |
| 8 | O | -2.686472 | 0.289639 | -0.000136 |
| 6 | C | -3.272229 | -1.029651 | -0.000148 |
| 1 | H | -2.965251 | -1.590829 | 0.895763 |
| 1 | H | -4.355436 | -0.854595 | -0.000326 |
| 1 | H | -2.964977 | -1.590945 | -0.895892 |

C8-OMe- PU _7H  $E_{tot}$ = -526.211236 a.u.
Gibbs free energy  $E_G$= -526.121766 a.u.

| | | | | |
|---|---|---|---|---|
| 7 | N | -2.89475 | 0.872809 | -0.00019 |
| 6 | C | -2.9321 | -0.47915 | -0.00019 |
| 7 | N | -1.904 | -1.3496 | -0.00011 |
| 6 | C | -0.69806 | -0.76271 | -3.9E-05 |
| 6 | C | -0.54512 | 0.65798 | -3.8E-05 |
| 6 | C | -1.67955 | 1.460684 | -9.7E-05 |
| 7 | N | 0.83491 | 0.86638 | 0.000074 |
| 6 | C | 1.410196 | -0.40146 | 0.000069 |
| 7 | N | 0.538137 | -1.39254 | 0.000032 |
| 1 | H | -3.9334 | -0.92228 | -0.00026 |
| 1 | H | 1.311685 | 1.758058 | -0.00011 |
| 1 | H | -1.64534 | 2.556757 | -9.9E-05 |
| 8 | O | 2.732727 | -0.61341 | 0.000146 |
| 6 | C | 3.594418 | 0.540888 | 0.00026 |
| 1 | H | 4.615129 | 0.141887 | 0.000367 |
| 1 | H | 3.43577 | 1.148069 | -0.90791 |
| 1 | H | 3.435561 | 1.148045 | 0.90841 |

C2-OMe- PU _7H  $E_{tot}$ = -526.220388 a.u.
Gibbs free energy  $E_G$= -526.130338 a.u.

| | | | | |
|---|---|---|---|---|
| 7 | N | 1.167766 | 1.068588 | 0.00002 |
| 6 | C | 1.277406 | -0.283115 | -0.000011 |
| 7 | N | 0.297709 | -1.203454 | -0.00002 |
| 6 | C | -0.931554 | -0.674188 | -0.000037 |
| 6 | C | -1.169425 | 0.736103 | -0.000042 |
| 6 | C | -0.071579 | 1.591281 | -0.000008 |
| 7 | N | -2.554096 | 0.863382 | -0.00013 |
| 6 | C | -3.063692 | -0.421545 | -0.000075 |
| 7 | N | -2.137747 | -1.36676 | -0.000118 |
| 1 | H | -3.091364 | 1.72123 | -0.000037 |
| 1 | H | -0.15354 | 2.684388 | -0.00002 |
| 1 | H | -4.138155 | -0.600946 | -0.000105 |
| 8 | O | 2.520977 | -0.818682 | 0.000121 |
| 6 | C | 3.633499 | 0.098462 | 0.000212 |
| 1 | H | 3.613997 | 0.736985 | 0.89693 |
| 1 | H | 4.52378 | -0.543523 | 0.000252 |
| 1 | H | 3.614106 | 0.737031 | -0.896473 |

C8-OH- PU _7H  $E_{tot}$ = -486.944078 a.u.
Gibbs free energy  $E_G$= -486.878838 a.u.

| | | | | |
|---|---|---|---|---|
| 7 | N | 2.570194 | 0.625693 | 0.000088 |
| 6 | C | 2.431312 | -0.71819 | -6.8E-05 |
| 7 | N | 1.295864 | -1.44548 | 0 |
| 6 | C | 0.18069 | -0.70452 | 0.000005 |
| 6 | C | 0.212035 | 0.725244 | 0.000039 |
| 6 | C | 1.443486 | 1.369789 | 0.000104 |
| 7 | N | -1.12971 | 1.111524 | -3.8E-05 |
| 6 | C | -1.85621 | -0.05941 | -8.1E-05 |
| 7 | N | -1.13378 | -1.16397 | -1.1E-05 |
| 1 | H | 3.365036 | -1.28985 | 0.000226 |
| 1 | H | -1.51562 | 2.047378 | 0.000135 |
| 1 | H | 1.554787 | 2.460305 | 0.000134 |
| 8 | O | -3.19603 | 0.014181 | -8.7E-05 |
| 1 | H | -3.52175 | -0.90311 | -0.00006 |

C2-OH- PU _7H  $E_{tot}$ = -486.947466 a.u.
Gibbs free energy  $E_G$= -486.882007 a.u.

| | | | | |
|---|---|---|---|---|
| 7 | N | 1.551645 | 1.192762 | 0.00015 |
| 6 | C | 1.72563 | -0.149076 | 0.000406 |
| 7 | N | 0.800436 | -1.122605 | 0.000187 |
| 6 | C | -0.462768 | -0.665405 | 0.000026 |
| 6 | C | -0.767733 | 0.728254 | -0.000038 |
| 6 | C | 0.287061 | 1.641855 | -0.000012 |
| 7 | N | -2.157194 | 0.785092 | -0.000183 |
| 6 | C | -2.602445 | -0.521756 | -0.000157 |
| 7 | N | -1.627323 | -1.419278 | -0.000114 |
| 1 | H | -2.736305 | 1.615409 | -0.000154 |
| 1 | H | 0.144246 | 2.728696 | -0.000106 |
| 1 | H | -3.665982 | -0.756241 | -0.000253 |
| 8 | O | 3.024932 | -0.541097 | -0.000106 |
| 1 | H | 3.00717 | -1.51412 | -0.000276 |

C8-OH- PU _7H  $E_{tot}$ = -486.933447 a.u.
Gibbs free energy  $E_G$= -486.869533 a.u.

| | | | | |
|---|---|---|---|---|
| 7 | N | 2.562673 | 0.655807 | 0.00007 |
| 6 | C | 2.44341 | -0.69066 | -0.00027 |
| 7 | N | 1.319565 | -1.43452 | -1.1E-05 |
| 6 | C | 0.191991 | -0.7114 | 0.000017 |
| 6 | C | 0.204105 | 0.71731 | 0.000036 |
| 6 | C | 1.424821 | 1.381994 | 0.00011 |
| 7 | N | -1.14377 | 1.081613 | -2.6E-05 |
| 6 | C | -1.85849 | -0.11182 | -0.0001 |
| 7 | N | -1.11173 | -1.19469 | 0.000109 |
| 1 | H | 3.385766 | -1.24799 | 0.000478 |
| 1 | H | -1.5175 | 2.022975 | 0.000235 |
| 1 | H | 1.5195 | 2.474305 | 0.000177 |
| 8 | O | -3.20418 | -0.15716 | -0.00006 |
| 1 | H | -3.57656 | 0.737926 | -0.00016 |

C2-OH- PU _7H  $E_{tot}$ = -486.946823 a.u.
Gibbs free energy  $E_G$= -486.881414 a.u.

| | | | | |
|---|---|---|---|---|
| 7 | N | 1.56693 | 1.131727 | 0.000219 |
| 6 | C | 1.710908 | -0.21991 | 0.000453 |
| 7 | N | 0.767112 | -1.167274 | 0.000181 |
| 6 | C | -0.480168 | -0.671373 | -0.000002 |
| 6 | C | -0.75696 | 0.730464 | -0.000022 |
| 6 | C | 0.315504 | 1.620254 | 0.000057 |
| 7 | N | -2.144126 | 0.818747 | -0.000198 |
| 6 | C | -2.617661 | -0.479074 | -0.000225 |
| 7 | N | -1.663705 | -1.397412 | -0.000184 |
| 1 | H | -2.705071 | 1.661452 | -0.000153 |
| 1 | H | 0.202387 | 2.710188 | -0.000009 |
| 1 | H | -3.686341 | -0.689296 | -0.000351 |
| 8 | O | 2.990436 | -0.67323 | -0.000106 |
| 1 | H | 3.552325 | 0.120813 | -0.000326 |

C8-$NH_2$- PU _7H  $E_{tot}$ = -467.082512 a.u.
Gibbs free energy  $E_G$= -467.00578 a.u.

| | | | | |
|---|---|---|---|---|
| 7 | N | 2.583791 | 0.634514 | 0.000082 |
| 6 | C | 2.449926 | -0.7089 | 0.002612 |
| 7 | N | 1.317595 | -1.44232 | 0.002619 |
| 6 | C | 0.196764 | -0.70749 | -0.00184 |
| 6 | C | 0.223813 | 0.722397 | -0.00168 |
| 6 | C | 1.450884 | 1.372637 | 0.001094 |
| 7 | N | -1.11831 | 1.103081 | 0.009631 |
| 6 | C | -1.85648 | -0.07435 | 0.000707 |
| 7 | N | -1.11077 | -1.1724 | 0.006696 |
| 1 | H | 3.385954 | -1.27717 | 0.004728 |
| 1 | H | -1.48478 | 2.039684 | -0.1053 |
| 1 | H | 1.557448 | 2.463839 | 0.000997 |
| 7 | N | -3.22982 | -0.06102 | -0.0774 |
| 1 | H | -3.63843 | -0.97517 | 0.091965 |
| 1 | H | -3.707 | 0.690016 | 0.410844 |

C2-$NH_2$- PU _7H  $E_{tot}$ = -467.087348 a.u.
Gibbs free energy  $E_G$= -467.010526 a.u.

| | | | | |
|---|---|---|---|---|
| 7 | N | -1.536799 | 1.183797 | 0.007392 |
| 6 | C | -1.720715 | -0.171669 | -0.00314 |
| 7 | N | -0.776714 | -1.137809 | 0.005359 |
| 6 | C | 0.478449 | -0.667724 | -0.000251 |
| 6 | C | 0.781574 | 0.72661 | -0.006434 |
| 6 | C | -0.276049 | 1.636627 | 0.005818 |
| 7 | N | 2.172069 | 0.789526 | -0.0053 |
| 6 | C | 2.621476 | -0.515675 | 0.001604 |
| 7 | N | 1.650057 | -1.416743 | 0.006692 |
| 1 | H | 2.748209 | 1.621665 | -0.011058 |
| 1 | H | -0.13514 | 2.724111 | 0.018397 |
| 1 | H | 3.686113 | -0.745971 | 0.002199 |
| 7 | N | -3.032326 | -0.588226 | -0.065868 |
| 1 | H | -3.207453 | -1.555795 | 0.174187 |
| 1 | H | -3.734149 | 0.093154 | 0.192774 |

C8-$NO_2$-PU_3H  $E_{tot}$ = -616.154310 a.u.
Gibbs free energy  $E_G$= -616.093342 a.u.

| | | | | |
|---|---|---|---|---|
| 7 | N | 0.726731 | 1.436678 | 0.000046 |
| 6 | C | 1.062845 | 0.173891 | 0.000039 |
| 7 | N | 0.214242 | -0.892265 | 0.00002 |
| 6 | C | -1.121382 | -0.640365 | 0.000036 |
| 6 | C | -1.565853 | 0.754405 | 0.000053 |
| 6 | C | -0.59918 | 1.75636 | 0.000041 |
| 7 | N | -2.93381 | 0.741005 | 0.000143 |
| 6 | C | -3.215506 | -0.581887 | 0.000137 |
| 7 | N | -2.156081 | -1.478015 | -0.000061 |
| 1 | H | 0.618149 | -1.832519 | -0.000032 |
| 1 | H | -0.846591 | 2.821367 | 0.000111 |
| 1 | H | -4.243444 | -0.947949 | 0.000092 |
| 7 | N | 2.507275 | -0.210606 | -0.000011 |
| 8 | O | 2.736153 | -1.431982 | -0.000208 |
| 8 | O | 3.338578 | 0.682869 | -0.000161 |

C2-$NO_2$-PU_3H  $E_{tot}$ = -616.156681 a.u.
Gibbs free energy  $E_G$= -616.097011 a.u.

| | | | | |
|---|---|---|---|---|
| 7 | N | 0.726731 | 1.436678 | 0.000046 |
| 6 | C | 1.062845 | 0.173891 | 0.000039 |
| 7 | N | 0.214242 | -0.892265 | 0.00002 |
| 6 | C | -1.121382 | -0.640365 | 0.000036 |
| 6 | C | -1.565853 | 0.754405 | 0.000053 |
| 6 | C | -0.59918 | 1.75636 | 0.000041 |
| 7 | N | -2.93381 | 0.741005 | 0.000143 |
| 6 | C | -3.215506 | -0.581887 | 0.000137 |
| 7 | N | -2.156081 | -1.478015 | -0.000061 |
| 1 | H | 0.618149 | -1.832519 | -0.000032 |
| 1 | H | -0.846591 | 2.821367 | 0.000111 |
| 1 | H | -4.243444 | -0.947949 | 0.000092 |
| 7 | N | 2.507275 | -0.210606 | -0.000011 |
| 8 | O | 2.736153 | -1.431982 | -0.000208 |
| 8 | O | 3.338578 | 0.682869 | -0.000161 |

C8-CN- PU _3H  $E_{tot}$ = -503.916727 a.u.
Gibbs free energy  $E_G$= -503.859019 a.u.

| | | | | |
|---|---|---|---|---|
| 7 | N | -2.804898 | 0.707231 | 0.000209 |
| 6 | C | -2.75132 | -0.612758 | 0.000277 |
| 7 | N | -1.590048 | -1.337483 | 0.000183 |
| 6 | C | -0.394882 | -0.677618 | 0.000077 |
| 6 | C | -0.409123 | 0.772658 | 0.000008 |
| 6 | C | -1.641325 | 1.420477 | 0.000054 |
| 7 | N | 0.896551 | 1.197873 | -0.000144 |
| 6 | C | 1.576328 | 0.028116 | 0.000266 |
| 7 | N | 0.845061 | -1.16029 | -0.000134 |
| 1 | H | -3.676213 | -1.193519 | 0.000341 |
| 1 | H | -1.611493 | -2.356472 | 0.000179 |
| 1 | H | -1.732907 | 2.510755 | 0.000026 |
| 6 | C | 3.009032 | -0.010064 | -0.000205 |
| 7 | N | 4.180242 | -0.048135 | -0.000601 |

C2-CN- PU _3H  $E_{tot}$ = -503.910832 a.u.
Gibbs free energy  $E_G$= -503.853456 a.u.

| | | | | |
|---|---|---|---|---|
| 7 | N | 1.091952 | 1.391241 | -0.000006 |
| 6 | C | 1.444886 | 0.112897 | -0.000129 |
| 7 | N | 0.550448 | -0.938958 | -0.000083 |
| 6 | C | -0.781173 | -0.667978 | 0.000007 |
| 6 | C | -1.208977 | 0.727107 | 0.000121 |
| 6 | C | -0.230529 | 1.715746 | 0.00015 |
| 7 | N | -2.579522 | 0.729353 | 0.000051 |
| 6 | C | -2.875693 | -0.588009 | -0.00018 |
| 7 | N | -1.824078 | -1.496002 | 0.000302 |
| 1 | H | 0.885397 | -1.902649 | -0.000066 |
| 1 | H | -0.469156 | 2.783189 | 0.000081 |
| 1 | H | -3.906866 | -0.944474 | 0.000106 |
| 6 | C | 2.835666 | -0.24546 | -0.000127 |
| 7 | N | 3.959136 | -0.580189 | -0.000146 |

C8-CHO- PU _3H    $E_{tot}$ = -524.999636 a.u.
Gibbs free energy    $E_G$= -524.931972 a.u.

| | | | | |
|---|---|---|---|---|
| 7 | N | -2.92171 | 0.565815 | -0.000067 |
| 6 | C | -2.766844 | -0.747008 | 0.000077 |
| 7 | N | -1.556038 | -1.382807 | 0.0001 |
| 6 | C | -0.409931 | -0.636037 | 0.000046 |
| 6 | C | -0.536583 | 0.811698 | -0.000077 |
| 6 | C | -1.814064 | 1.363939 | -0.000176 |
| 7 | N | 0.734403 | 1.326915 | 0.00011 |
| 6 | C | 1.507642 | 0.211836 | 0.000209 |
| 7 | N | 0.859286 | -1.022901 | -0.000175 |
| 1 | H | -3.646091 | -1.395066 | 0.000046 |
| 1 | H | -1.501361 | -2.40062 | 0.0001 |
| 1 | H | -1.988597 | 2.444301 | -0.000092 |
| 6 | C | 2.987071 | 0.353462 | 0.000253 |
| 8 | O | 3.775093 | -0.578255 | -0.000226 |
| 1 | H | 3.319957 | 1.420939 | -0.000013 |

C2-CHO- PU _3H    $E_{tot}$ = -525.006500 a.u.
Gibbs free energy    $E_G$= -524.938374 a.u.

| | | | | |
|---|---|---|---|---|
| 7 | N | 0.932823 | 1.567859 | -0.000141 |
| 6 | C | 1.390479 | 0.318788 | 0.000021 |
| 7 | N | 0.59096 | -0.795487 | 0.00008 |
| 6 | C | -0.754252 | -0.647247 | 0.000046 |
| 6 | C | -1.301552 | 0.710099 | -0.000116 |
| 6 | C | -0.409589 | 1.781838 | -0.000255 |
| 7 | N | -2.664416 | 0.597899 | 0.000249 |
| 6 | C | -2.848111 | -0.74426 | 0.000145 |
| 7 | N | -1.72886 | -1.559211 | -0.000227 |
| 1 | H | 1.050694 | -1.710337 | 0.000072 |
| 1 | H | -0.744038 | 2.823608 | -0.000087 |
| 1 | H | -3.847392 | -1.182923 | 0.000097 |
| 6 | C | 2.852291 | 0.067271 | -0.000001 |
| 8 | O | 3.32206 | -1.064563 | 0.000183 |
| 1 | H | 3.475107 | 0.989812 | -0.000313 |

C8-CHO- PU _3H    $E_{tot}$ = -524.998002 a.u.
Gibbs free energy    $E_G$= -524.930509 a.u.

| | | | | |
|---|---|---|---|---|
| 7 | N | 2.813552 | 0.85776 | -0.000017 |
| 6 | C | 2.867003 | -0.463683 | -0.000008 |
| 7 | N | 1.769576 | -1.279969 | 0.00005 |
| 6 | C | 0.522381 | -0.720493 | 0.000035 |
| 6 | C | 0.418375 | 0.728845 | -0.000006 |
| 6 | C | 1.596642 | 1.473429 | -0.000011 |
| 7 | N | -0.913541 | 1.047149 | 0.000018 |
| 6 | C | -1.507494 | -0.168125 | -0.000452 |
| 7 | N | -0.675509 | -1.294427 | 0.000261 |
| 1 | H | 3.836598 | -0.966505 | 0.000075 |
| 1 | H | 1.875841 | -2.293644 | 0.000148 |
| 1 | H | 1.599396 | 2.567744 | -0.000068 |
| 6 | C | -2.980195 | -0.384058 | -0.000366 |
| 8 | O | -3.813393 | 0.506215 | 0.000381 |
| 1 | H | -3.263502 | -1.466399 | -0.000535 |

C2-CHO- PU _3H    $E_{tot}$ = -524.995120 a.u.
Gibbs free energy    $E_G$= -524.927741 a.u.

| | | | | |
|---|---|---|---|---|
| 7 | N | -1.124141 | 1.212194 | 0.000152 |
| 6 | C | -1.367109 | -0.092932 | 0.000176 |
| 7 | N | -0.370475 | -1.048664 | 0.000002 |
| 6 | C | 0.928662 | -0.664147 | -0.000056 |
| 6 | C | 1.228767 | 0.762538 | -0.000065 |
| 6 | C | 0.157659 | 1.656701 | -0.000033 |
| 7 | N | 2.590729 | 0.890569 | 0.000153 |
| 6 | C | 3.007086 | -0.398416 | 0.00025 |
| 7 | N | 2.046375 | -1.395414 | -0.0006 |
| 1 | H | -0.610754 | -2.041488 | -0.000153 |
| 1 | H | 0.300977 | 2.741761 | 0.00025 |
| 1 | H | 4.06731 | -0.656208 | -0.000215 |
| 6 | C | -2.757556 | -0.634418 | 0.000147 |
| 8 | O | -3.767024 | 0.041047 | -0.000113 |
| 1 | H | -2.803808 | -1.759194 | 0.000558 |

C8-Cl- PU _3H    $E_{tot}$ = -871.362744 a.u.
Gibbs free energy    $E_G$= -871.31204 a.u.

| | | | | |
|---|---|---|---|---|
| 7 | N | -2.915236 | 0.70673 | 0.000108 |
| 6 | C | -2.863898 | -0.60994 | 0.000224 |
| 7 | N | -1.698123 | -1.335566 | 0.000192 |
| 6 | C | -0.505379 | -0.675812 | 0.000093 |
| 6 | C | -0.518404 | 0.775801 | -0.00002 |
| 6 | C | -1.748147 | 1.421744 | -0.00006 |
| 7 | N | 0.794006 | 1.196575 | 0.000104 |
| 6 | C | 1.454764 | 0.029645 | 0.000052 |
| 7 | N | 0.737642 | -1.158016 | -0.00011 |
| 1 | H | -3.787265 | -1.193159 | 0.000269 |
| 1 | H | -1.718424 | -2.354106 | 0.000212 |
| 1 | H | -1.840943 | 2.511697 | 0.000019 |
| 17 | Cl | 3.176764 | -0.028301 | -0.00025 |

C2-Cl- PU _3H    $E_{tot}$ = -871.358895 a.u.
Gibbs free energy    $E_G$= -871.308459 a.u.

| | | | | |
|---|---|---|---|---|
| 7 | N | 1.002408 | 1.373047 | 0.000046 |
| 6 | C | 1.329778 | 0.109361 | 0.000051 |
| 7 | N | 0.445577 | -0.9361 | -0.00004 |
| 6 | C | -0.894277 | -0.652501 | -0.00003 |
| 6 | C | -1.308786 | 0.740672 | -0.00000 |
| 6 | C | -0.324809 | 1.716556 | -0.00001 |
| 7 | N | -2.68764 | 0.751995 | -0.00004 |
| 6 | C | -2.990031 | -0.556666 | 0.000553 |
| 7 | N | -1.937577 | -1.474538 | -0.00029 |
| 1 | H | 0.779104 | -1.898543 | -0.00015 |
| 1 | H | -0.546329 | 2.787428 | 0.000044 |
| 1 | H | -4.022173 | -0.910962 | 0.000181 |
| 17 | Cl | 3.009339 | -0.360194 | -0.00007 |

C8-F- PU_3H $E_{tot}$ = -510.937515 a.u.
Gibbs free energy $E_G$= -510.884072 a.u.

| | | | | |
|---|---|---|---|---|
| 7 | N | -2.493728 | 0.717531 | 0.000044 |
| 6 | C | -2.451702 | -0.598259 | 0.00016 |
| 7 | N | -1.289058 | -1.331503 | 0.000151 |
| 6 | C | -0.094022 | -0.679703 | 0.000072 |
| 6 | C | -0.096707 | 0.774028 | -0.000048 |
| 6 | C | -1.321632 | 1.426146 | -0.000104 |
| 7 | N | 1.22044 | 1.184906 | 0.000107 |
| 6 | C | 1.855805 | 0.012509 | 0.000046 |
| 7 | N | 1.147853 | -1.169438 | -0.000106 |
| 1 | H | -3.378184 | -1.176495 | 0.000194 |
| 1 | H | -1.31561 | -2.34987 | 0.000173 |
| 1 | H | -1.408584 | 2.516525 | -0.000032 |
| 9 | F | 3.183709 | -0.04544 | -0.000274 |

C2-F- PU_3H $E_{tot}$ = -510.932605 a.u.
Gibbs free energy $E_G$= -510.879613 a.u.

| | | | | |
|---|---|---|---|---|
| 7 | N | 1.513638 | 1.192017 | -0.000008 |
| 6 | C | 1.708033 | -0.091007 | -0.000295 |
| 7 | N | 0.754806 | -1.062751 | -0.000195 |
| 6 | C | -0.559902 | -0.662358 | -0.000012 |
| 6 | C | -0.846009 | 0.762427 | 0.000189 |
| 6 | C | 0.217555 | 1.649344 | 0.000322 |
| 7 | N | -2.221346 | 0.896034 | -0.000227 |
| 6 | C | -2.638611 | -0.37783 | -0.000156 |
| 7 | N | -1.669377 | -1.387572 | 0.000544 |
| 1 | H | 1.013546 | -2.048277 | -0.000159 |
| 1 | H | 0.088874 | 2.73506 | 0.000102 |
| 1 | H | -3.697652 | -0.640365 | 0.000142 |
| 9 | F | 2.962754 | -0.57711 | -0.00013 |

C8-H- PU_3H $E_{tot}$ = -411.727345 a.u.
Gibbs free energy $E_G$= -411.664858 a.u.

| | | | | |
|---|---|---|---|---|
| 7 | N | 2.072937 | 0.745955 | 0.000539 |
| 6 | C | 2.048809 | -0.57306 | 0.000429 |
| 7 | N | 0.901102 | -1.3226 | -9E-06 |
| 6 | C | -0.30896 | -0.69164 | -0.00031 |
| 6 | C | -0.327 | 0.761381 | -0.00014 |
| 6 | C | 0.888871 | 1.432181 | 0.000271 |
| 7 | N | -1.6492 | 1.149621 | -0.00059 |
| 6 | C | -2.29914 | -0.0285 | 0.000644 |
| 7 | N | -1.54133 | -1.19673 | -0.00084 |
| 1 | H | 0.958232 | 2.524362 | 0.000366 |
| 1 | H | -3.38904 | -0.08448 | 0.000104 |
| 1 | H | 2.985325 | -1.13528 | 0.000592 |
| 1 | H | 0.945502 | -2.34051 | -0.00015 |

C2-H- PU_3H $E_{tot}$ = -411.727345 a.u.
Gibbs free energy $E_G$= -411.664858 a.u.

| | | | | |
|---|---|---|---|---|
| 7 | N | 2.072937 | 0.745955 | 0.000539 |
| 6 | C | 2.048809 | -0.57306 | 0.000429 |
| 7 | N | 0.901102 | -1.3226 | -9E-06 |
| 6 | C | -0.30896 | -0.69164 | -0.00031 |
| 6 | C | -0.327 | 0.761381 | -0.00014 |
| 6 | C | 0.888871 | 1.432181 | 0.000271 |
| 7 | N | -1.6492 | 1.149621 | -0.00059 |
| 6 | C | -2.29914 | -0.0285 | 0.000644 |
| 7 | N | -1.54133 | -1.19673 | -0.00084 |
| 1 | H | 0.958232 | 2.524362 | 0.000366 |
| 1 | H | -3.38904 | -0.08448 | 0.000104 |
| 1 | H | 2.985325 | -1.13528 | 0.000592 |
| 1 | H | 0.945502 | -2.34051 | -0.00015 |

C8-Me- PU_3H $E_{tot}$ = -451.030158 a.u.
Gibbs free energy $E_G$= -450.943926 a.u.

| | | | | |
|---|---|---|---|---|
| 7 | N | 2.544541 | 0.715094 | -0.000173 |
| 6 | C | 2.498045 | -0.601375 | -0.000283 |
| 7 | N | 1.335088 | -1.331912 | -0.000198 |
| 6 | C | 0.136032 | -0.680142 | -0.000061 |
| 6 | C | 0.144076 | 0.773607 | 0.000057 |
| 6 | C | 1.370568 | 1.422782 | 0.000042 |
| 7 | N | -1.16971 | 1.183228 | -0.000011 |
| 6 | C | -1.856055 | 0.020096 | -0.000037 |
| 7 | N | -1.102111 | -1.161698 | 0.000227 |
| 1 | H | 3.423758 | -1.181168 | -0.000356 |
| 1 | H | 1.361675 | -2.350128 | -0.000205 |
| 1 | H | 1.459328 | 2.513366 | -0.000044 |
| 6 | C | -3.34656 | -0.037763 | 0.000352 |
| 1 | H | -3.70622 | -0.587411 | -0.88567 |
| 1 | H | -3.705807 | -0.588034 | 0.886152 |
| 1 | H | -3.764033 | 0.977166 | 0.00078 |

C2-Me- PU_3H $E_{tot}$ = -451.028624 a.u.
Gibbs free energy $E_G$= -450.9412 a.u.

| | | | | |
|---|---|---|---|---|
| 7 | N | -1.440553 | 1.236491 | 0.000004 |
| 6 | C | -1.713073 | -0.060779 | 0.000129 |
| 7 | N | -0.728303 | -1.02184 | 0.000104 |
| 6 | C | 0.587614 | -0.657864 | 0.000011 |
| 6 | C | 0.911099 | 0.755026 | -0.000103 |
| 6 | C | -0.139829 | 1.659643 | -0.000141 |
| 7 | N | 2.287952 | 0.857846 | 0.000006 |
| 6 | C | 2.67409 | -0.429581 | 0.000033 |
| 7 | N | 1.685086 | -1.412484 | -0.00019 |
| 1 | H | -0.974528 | -2.010433 | 0.000117 |
| 1 | H | 0.01686 | 2.742897 | -0.000081 |
| 1 | H | 3.727425 | -0.71521 | -0.000086 |
| 6 | C | -3.13648 | -0.541864 | 0.000116 |
| 1 | H | -3.348309 | -1.156344 | -0.891645 |
| 1 | H | -3.348239 | -1.156601 | 0.891712 |
| 1 | H | -3.802995 | 0.32812 | 0.000258 |

C8-OMe- PU_3H $E_{tot}$ = -526.215092 a.u.
Gibbs free energy $E_G$= -526.124594 a.u.

| | | | | |
|---|---|---|---|---|
| 7 | N | -2.821288 | 0.951938 | 0.000203 |
| 6 | C | -2.949884 | -0.355737 | 0.000228 |
| 7 | N | -1.888695 | -1.234815 | 0.000153 |
| 6 | C | -0.617191 | -0.745403 | 0.000017 |
| 6 | C | -0.43304 | 0.698701 | -0.000017 |
| 6 | C | -1.563314 | 1.502571 | 0.000086 |
| 7 | N | 0.922491 | 0.933876 | -0.00005 |
| 6 | C | 1.425797 | -0.318308 | -0.000343 |
| 7 | N | 0.543017 | -1.393967 | -0.000122 |
| 1 | H | -3.942504 | -0.811383 | 0.000314 |
| 1 | H | -2.046443 | -2.240681 | 0.000174 |
| 1 | H | -1.509232 | 2.595047 | 0.000129 |
| 8 | O | 2.729463 | -0.597194 | -0.000217 |
| 6 | C | 3.614824 | 0.549188 | -0.000015 |
| 1 | H | 4.626593 | 0.125817 | 0.000063 |
| 1 | H | 3.442125 | 1.161803 | -0.897479 |
| 1 | H | 3.441928 | 1.161655 | 0.897514 |

C2-OMe- PU_3H $E_{tot}$ = -526.212989 a.u.
Gibbs free energy $E_G$= -526.122762 a.u.

| | | | | |
|---|---|---|---|---|
| 7 | N | 1.118481 | 1.102136 | -0.00004 |
| 6 | C | 1.281589 | -0.204989 | -0.000049 |
| 7 | N | 0.260839 | -1.118824 | 0.000039 |
| 6 | C | -1.032124 | -0.661023 | 0.00004 |
| 6 | C | -1.258648 | 0.769998 | 0.000049 |
| 6 | C | -0.155669 | 1.606773 | 0.000002 |
| 7 | N | -2.630141 | 0.964523 | 0.000552 |
| 6 | C | -3.100176 | -0.289906 | -0.000798 |
| 7 | N | -2.173874 | -1.339366 | 0.000159 |
| 1 | H | 0.470527 | -2.1152 | 0.00009 |
| 1 | H | -0.241765 | 2.697486 | 0.000204 |
| 1 | H | -4.169404 | -0.507903 | -0.000367 |
| 8 | O | 2.495857 | -0.786784 | -0.00002 |
| 6 | C | 3.633431 | 0.114394 | -0.000023 |
| 1 | H | 3.614178 | 0.747891 | -0.898216 |
| 1 | H | 3.61414 | 0.747947 | 0.898131 |
| 1 | H | 4.507906 | -0.546705 | 0.000016 |

C8-OMe- PU_3H $E_{tot}$ = -526.212890 a.u.
Gibbs free energy $E_G$= -526.122596 a.u.

| | | | | |
|---|---|---|---|---|
| 7 | N | 3.005447 | 0.443715 | -0.00002 |
| 6 | C | 2.778203 | -0.851569 | 0.000002 |
| 7 | N | 1.517731 | -1.409748 | 0.000008 |
| 6 | C | 0.428395 | -0.594716 | 0.000014 |
| 6 | C | 0.636425 | 0.84537 | -0.000007 |
| 6 | C | 1.945878 | 1.312893 | -0.000035 |
| 7 | N | -0.599462 | 1.440543 | 0.000018 |
| 6 | C | -1.425721 | 0.375481 | 0.000082 |
| 7 | N | -0.867677 | -0.902776 | 0.000012 |
| 1 | H | 3.610464 | -1.55847 | 0 |
| 1 | H | 1.398591 | -2.421013 | 0.000012 |
| 1 | H | 2.188315 | 2.37937 | -0.000037 |
| 8 | O | -2.746998 | 0.564693 | 0.000007 |
| 6 | C | -3.572786 | -0.623291 | -0.00005 |
| 1 | H | -4.604345 | -0.250163 | -0.000091 |
| 1 | H | -3.3758 | -1.227197 | -0.898632 |
| 1 | H | -3.375878 | -1.227223 | 0.898532 |

C2-OMe- PU_3H $E_{tot}$ = -526.203064 a.u.
Gibbs free energy $E_G$= -526.113033 a.u.

| | | | | |
|---|---|---|---|---|
| 7 | N | -0.814486 | 1.625303 | -0.000015 |
| 6 | C | -1.320984 | 0.407918 | -0.000067 |
| 7 | N | -0.567049 | -0.739351 | -0.000016 |
| 6 | C | 0.804403 | -0.627295 | -0.000034 |
| 6 | C | 1.394806 | 0.688695 | -0.000039 |
| 6 | C | 0.54009 | 1.782637 | 0.000042 |
| 7 | N | 2.768578 | 0.51836 | -0.000299 |
| 6 | C | 2.89515 | -0.816599 | -0.00031 |
| 7 | N | 1.726256 | -1.584818 | 0.000237 |
| 1 | H | -0.984218 | -1.666606 | 0.000084 |
| 1 | H | 0.904251 | 2.814709 | -0.000133 |
| 1 | H | 3.870027 | -1.306773 | -0.000198 |
| 8 | O | -2.669586 | 0.320424 | 0.000083 |
| 6 | C | -3.285208 | -0.97912 | 0.000281 |
| 1 | H | -3.016096 | -1.54641 | -0.908374 |
| 1 | H | -3.015849 | -1.546262 | 0.908955 |
| 1 | H | -4.364063 | -0.785932 | 0.000412 |

C8-OH- PU_3H $E_{tot}$ = -486.939490 a.u.
Gibbs free energy $E_G$= -486.873581 a.u.

| | | | | |
|---|---|---|---|---|
| 7 | N | -2.49731 | 0.737968 | 0.000016 |
| 6 | C | -2.471129 | -0.576255 | 0.000013 |
| 7 | N | -1.31293 | -1.322676 | -0.00002 |
| 6 | C | -0.110056 | -0.685983 | 0.00003 |
| 6 | C | -0.096398 | 0.769508 | 0.000032 |
| 6 | C | -1.315031 | 1.433885 | 0.000004 |
| 7 | N | 1.223518 | 1.15778 | -0.000162 |
| 6 | C | 1.865763 | -0.027816 | 0.000517 |
| 7 | N | 1.121323 | -1.194955 | 0.000069 |
| 1 | H | -3.402719 | -1.146111 | 0.000016 |
| 1 | H | -1.349511 | -2.340341 | -0.000033 |
| 1 | H | -1.391206 | 2.524994 | -0.000106 |
| 8 | O | 3.203109 | -0.119946 | -0.00026 |
| 1 | H | 3.537458 | 0.79416 | -0.000691 |

C8-OH- PU_3H $E_{tot}$ = -486.938355 a.u.
Gibbs free energy $E_G$= -486.872502 a.u.

| | | | | |
|---|---|---|---|---|
| 7 | N | 2.513791 | 0.692686 | -0.000238 |
| 6 | C | 2.456961 | -0.620366 | -0.00027 |
| 7 | N | 1.280703 | -1.33917 | -0.000176 |
| 6 | C | 0.094165 | -0.673264 | -0.000019 |
| 6 | C | 0.113729 | 0.78236 | 0.000021 |
| 6 | C | 1.348941 | 1.416709 | -0.000091 |
| 7 | N | -1.192808 | 1.213473 | 0.000004 |
| 6 | C | -1.867619 | 0.054506 | 0.000398 |
| 7 | N | -1.152923 | -1.142482 | 0.000168 |
| 1 | H | 3.374563 | -1.212456 | -0.000364 |
| 1 | H | 1.295319 | -2.357277 | -0.000194 |
| 1 | H | 1.449816 | 2.505708 | -0.000178 |
| 8 | O | -3.208656 | 0.029981 | 0.000241 |
| 1 | H | -3.468851 | -0.907035 | 0.000271 |

C8-$NH_2$- PU_3H $E_{tot}$ = -467.081976 a.u.
Gibbs free energy $E_G$= -467.0048 a.u.

| | | | | |
|---|---|---|---|---|
| 7 | N | -2.522604 | 0.712205 | 0.003991 |
| 6 | C | -2.480656 | -0.59974 | 0.003232 |
| 7 | N | -1.308658 | -1.331697 | 0.000187 |
| 6 | C | -0.114222 | -0.679297 | -0.002707 |
| 6 | C | -0.118907 | 0.777832 | -0.002659 |
| 6 | C | -1.34854 | 1.424788 | 0.001171 |
| 7 | N | 1.190386 | 1.189357 | -0.001494 |
| 6 | C | 1.86654 | 0.015951 | -0.003468 |
| 7 | N | 1.122831 | -1.168451 | -0.00252 |
| 1 | H | -3.403274 | -1.183931 | 0.004794 |
| 1 | H | -1.332856 | -2.349237 | -0.000948 |
| 1 | H | -1.439289 | 2.514629 | 0.002132 |
| 7 | N | 3.225037 | -0.043906 | -0.04324 |
| 1 | H | 3.736081 | 0.807076 | 0.150315 |
| 1 | H | 3.665107 | -0.9283 | 0.171835 |

C2-OH- PU_3H $E_{tot}$ = -486.937002 a.u.
Gibbs free energy $E_G$= -486.871473 a.u.

| | | | | |
|---|---|---|---|---|
| 7 | N | 1.510664 | 1.174249 | -0.00005 |
| 6 | C | 1.712858 | -0.127247 | -0.000105 |
| 7 | N | 0.732913 | -1.075942 | -0.000045 |
| 6 | C | -0.576998 | -0.660725 | 0.000022 |
| 6 | C | -0.851454 | 0.761647 | 0.000065 |
| 6 | C | 0.221313 | 1.637891 | 0.000039 |
| 7 | N | -2.22819 | 0.908762 | 0.000233 |
| 6 | C | -2.655746 | -0.360825 | -0.000162 |
| 7 | N | -1.693552 | -1.377796 | 0.000127 |
| 1 | H | 0.975337 | -2.065019 | -0.000048 |
| 1 | H | 0.099181 | 2.724844 | 0.000097 |
| 1 | H | -3.716712 | -0.615233 | 0.000004 |
| 8 | O | 2.965555 | -0.635651 | -0.000119 |
| 1 | H | 3.565076 | 0.131258 | -0.000107 |

C2-OH- PU_3H $E_{tot}$ = -486.924866 a.u.
Gibbs free energy $E_G$= -486.860497 a.u.

| | | | | |
|---|---|---|---|---|
| 7 | N | 1.491343 | 1.210464 | -0.000038 |
| 6 | C | 1.727887 | -0.081328 | -0.000216 |
| 7 | N | 0.751592 | -1.048302 | -0.000122 |
| 6 | C | -0.566696 | -0.654299 | 0.000014 |
| 6 | C | -0.866235 | 0.759516 | 0.000147 |
| 6 | C | 0.196548 | 1.649262 | 0.000197 |
| 7 | N | -2.245461 | 0.881019 | 0.000036 |
| 6 | C | -2.649684 | -0.396359 | -0.000159 |
| 7 | N | -1.668162 | -1.394947 | 0.000376 |
| 1 | H | 0.967922 | -2.044062 | -0.000099 |
| 1 | H | 0.058004 | 2.734591 | 0.000109 |
| 1 | H | -3.705609 | -0.67077 | 0.000098 |
| 8 | O | 3.027393 | -0.472388 | -0.000196 |
| 1 | H | 3.10444 | -1.439052 | -0.000198 |

C2-$NH_2$- PU_3H $E_{tot}$ = -467.073558 a.u.
Gibbs free energy $E_G$= -466.996686 a.u.

| | | | | |
|---|---|---|---|---|
| 7 | N | 1.481805 | 1.203572 | 0.005288 |
| 6 | C | 1.720895 | -0.102657 | -0.001532 |
| 7 | N | 0.731229 | -1.055968 | -0.015315 |
| 6 | C | -0.58305 | -0.65583 | 0.000584 |
| 6 | C | -0.876934 | 0.757634 | 0.006785 |
| 6 | C | 0.18999 | 1.643677 | -0.005605 |
| 7 | N | -2.256546 | 0.887137 | 0.010205 |
| 6 | C | -2.666898 | -0.388395 | 0.005034 |
| 7 | N | -1.690227 | -1.390995 | -0.002568 |
| 1 | H | 0.952521 | -2.047507 | -0.07979 |
| 1 | H | 0.051944 | 2.729408 | -0.016503 |
| 1 | H | -3.724373 | -0.65769 | 0.005794 |
| 7 | N | 3.029011 | -0.548417 | -0.064036 |
| 1 | H | 3.69756 | 0.195973 | 0.106743 |
| 1 | H | 3.251424 | -1.414061 | 0.417151 |

C8-$NO_2$-PU_1H $E_{tot}$ = -616.150185 a.u.
Gibbs free energy $E_G$= -616.089244 a.u.

| | | | | |
|---|---|---|---|---|
| 7 | N | 3.092628 | 0.602164 | 0.000434 |
| 6 | C | 3.03158 | -0.778356 | 0.000301 |
| 7 | N | 1.91666 | -1.471762 | 0.000091 |
| 6 | C | 0.776908 | -0.728514 | 0.000023 |
| 6 | C | 0.770026 | 0.742416 | 0.00014 |
| 6 | C | 1.980075 | 1.403689 | 0.00036 |
| 7 | N | -0.525469 | 1.182826 | 0 |
| 6 | C | -1.180454 | 0.010505 | -0.0001 |
| 7 | N | -0.492306 | -1.170186 | -0.000275 |
| 1 | H | 3.998514 | -1.286864 | 0.000368 |
| 1 | H | 4.010101 | 1.037249 | 0.000589 |
| 1 | H | 2.127003 | 2.484645 | 0.000476 |
| 7 | N | -2.672119 | -0.002616 | -0.000319 |
| 8 | O | -3.217633 | -1.105046 | -0.000147 |
| 8 | O | -3.237392 | 1.09049 | -0.000514 |

C2-$NO_2$-PU_1H $E_{tot}$ = -616.154259 a.u.
Gibbs free energy $E_G$= -616.094487 a.u.

| | | | | |
|---|---|---|---|---|
| 7 | N | 0.698681 | 1.293746 | 0.000192 |
| 6 | C | 1.021605 | -0.035864 | 0.000056 |
| 7 | N | 0.188416 | -1.041301 | -0.000133 |
| 6 | C | -1.117484 | -0.678739 | -0.000174 |
| 6 | C | -1.564016 | 0.729424 | -0.000056 |
| 6 | C | -0.601642 | 1.720796 | 0.000142 |
| 7 | N | -2.92584 | 0.756571 | -0.000264 |
| 6 | C | -3.230954 | -0.569236 | -0.000056 |
| 7 | N | -2.199678 | -1.476696 | -0.00052 |
| 1 | H | 1.492281 | 1.936195 | 0.000307 |
| 1 | H | -0.778341 | 2.796835 | 0.000227 |
| 1 | H | -4.269206 | -0.906068 | -0.000463 |
| 7 | N | 2.49265 | -0.281374 | 0.000123 |
| 8 | O | 2.883438 | -1.436684 | 0.000125 |
| 8 | O | 3.207888 | 0.73895 | 0.000458 |

C8-CN-PU_1H $E_{tot}$ = -503.912548 a.u.
Gibbs free energy $E_G$= -503.85486 a.u.

| | | | | |
|---|---|---|---|---|
| 7 | N | 2.720685 | 0.598393 | 0.000405 |
| 6 | C | 2.657642 | -0.780934 | 0.000284 |
| 7 | N | 1.54095 | -1.472108 | 0.000077 |
| 6 | C | 0.401006 | -0.727777 | -0.000093 |
| 6 | C | 0.395393 | 0.742703 | 0.000046 |
| 6 | C | 1.607463 | 1.400961 | 0.000307 |
| 7 | N | -0.89645 | 1.187806 | 0.000031 |
| 6 | C | -1.580145 | 0.013376 | -0.000865 |
| 7 | N | -0.865572 | -1.170904 | -0.000091 |
| 1 | H | 3.623351 | -1.291813 | 0.000439 |
| 1 | H | 3.638247 | 1.032891 | 0.000616 |
| 1 | H | 1.756556 | 2.481582 | 0.00047 |
| 6 | C | -3.015617 | -0.002694 | -0.000401 |
| 7 | N | -4.187129 | -0.014112 | -0.000021 |

C2-CN-PU_1H $E_{tot}$ = -503.907115 a.u.
Gibbs free energy $E_G$= -503.849669 a.u.

| | | | | |
|---|---|---|---|---|
| 7 | N | 1.066651 | 1.245509 | 0.000184 |
| 6 | C | 1.400964 | -0.102479 | 0.000063 |
| 7 | N | 0.519849 | -1.088984 | -0.000085 |
| 6 | C | -0.776893 | -0.700136 | -0.000169 |
| 6 | C | -1.20392 | 0.712239 | -0.000015 |
| 6 | C | -0.229541 | 1.688131 | 0.000147 |
| 7 | N | -2.567012 | 0.760913 | 0.000086 |
| 6 | C | -2.890492 | -0.558017 | -0.000806 |
| 7 | N | -1.871159 | -1.481636 | -0.000019 |
| 1 | H | 1.825658 | 1.920939 | 0.000342 |
| 1 | H | -0.393229 | 2.766627 | 0.000301 |
| 1 | H | -3.93305 | -0.881117 | -0.000393 |
| 6 | C | 2.810598 | -0.379269 | 0.000194 |
| 7 | N | 3.971147 | -0.545698 | 0.000301 |

C8-CHO- PU_1H $E_{tot}$ = -524.993922 a.u.
Gibbs free energy $E_G$= -524.926397 a.u.

| | | | | |
|---|---|---|---|---|
| 7 | N | 2.83078 | 0.466544 | 0.000403 |
| 6 | C | 2.663968 | -0.906207 | 0.000155 |
| 7 | N | 1.501332 | -1.513442 | -0.000072 |
| 6 | C | 0.415131 | -0.687357 | -0.000068 |
| 6 | C | 0.522387 | 0.782468 | 0.000202 |
| 6 | C | 1.779541 | 1.348799 | 0.000427 |
| 7 | N | -0.734564 | 1.315339 | 0.000264 |
| 6 | C | -1.511436 | 0.196026 | -0.000327 |
| 7 | N | -0.877477 | -1.03285 | -0.000182 |
| 1 | H | 3.59052 | -1.485591 | 0.000185 |
| 1 | H | 3.778249 | 0.830775 | 0.000591 |
| 1 | H | 2.009247 | 2.415508 | 0.000659 |
| 6 | C | -2.991893 | 0.362401 | -0.00036 |
| 8 | O | -3.797378 | -0.552876 | -0.000463 |
| 1 | H | -3.305672 | 1.436396 | -0.000801 |

C2-CHO- PU_1H $E_{tot}$ = -524.990732 a.u.
Gibbs free energy $E_G$= -524.923335 a.u.

| | | | | |
|---|---|---|---|---|
| 7 | N | 0.889993 | 1.424246 | 0.000217 |
| 6 | C | 1.360015 | 0.117938 | 0.000136 |
| 7 | N | 0.565688 | -0.942494 | -0.000024 |
| 6 | C | -0.75611 | -0.677019 | -0.000108 |
| 6 | C | -1.318523 | 0.689635 | -0.000011 |
| 6 | C | -0.435763 | 1.752987 | 0.000157 |
| 7 | N | -2.676624 | 0.61061 | -0.000141 |
| 6 | C | -2.874634 | -0.738591 | -0.00027 |
| 7 | N | -1.778184 | -1.558926 | -0.000235 |
| 1 | H | 1.582062 | 2.16995 | 0.000333 |
| 1 | H | -0.699973 | 2.811649 | 0.000232 |
| 1 | H | -3.883926 | -1.154786 | -0.000372 |
| 6 | C | 2.845661 | -0.003263 | 0.000243 |
| 8 | O | 3.461428 | -1.050041 | -0.000047 |
| 1 | H | 3.38043 | 0.989347 | 0.000572 |

C8-CHO- PU_1H  $E_{tot}$ = -524.993886 a.u.
Gibbs free energy  $E_G$= -524.926391 a.u.

| | | | | |
|---|---|---|---|---|
| 7 | N | 2.739122 | 0.735461 | 0.000036 |
| 6 | C | 2.784877 | -0.64599 | 0.00007 |
| 7 | N | 1.726112 | -1.422501 | 0.000048 |
| 6 | C | 0.530095 | -0.769813 | -0.000021 |
| 6 | C | 0.408484 | 0.698364 | -0.000053 |
| 6 | C | 1.567715 | 1.448565 | -0.00003 |
| 7 | N | -0.911075 | 1.041066 | -0.000076 |
| 6 | C | -1.511524 | -0.177301 | -0.000236 |
| 7 | N | -0.699452 | -1.302012 | 0.000057 |
| 1 | H | 3.788437 | -1.077954 | 0.000135 |
| 1 | H | 3.620144 | 1.23994 | 0.000067 |
| 1 | H | 1.632362 | 2.537708 | -0.000047 |
| 6 | C | -2.990489 | -0.369754 | -0.000213 |
| 8 | O | -3.808507 | 0.53483 | 0.000302 |
| 1 | H | -3.290781 | -1.446852 | -0.000125 |

C2-CHO- PU_1H  $E_{tot}$ = -525.004460 a.u.
Gibbs free energy  $E_G$= -524.936198 a.u.

| | | | | |
|---|---|---|---|---|
| 7 | N | 1.102854 | 1.055043 | 0.000251 |
| 6 | C | 1.308136 | -0.308051 | 0.000114 |
| 7 | N | 0.334962 | -1.211442 | -0.000095 |
| 6 | C | -0.915487 | -0.701357 | -0.000163 |
| 6 | C | -1.203278 | 0.745818 | -0.000029 |
| 6 | C | -0.134363 | 1.625336 | 0.000187 |
| 7 | N | -2.552453 | 0.92774 | -0.000186 |
| 6 | C | -3.006776 | -0.356981 | -0.000269 |
| 7 | N | -2.086908 | -1.372331 | -0.00038 |
| 1 | H | 1.955298 | 1.617671 | 0.000405 |
| 1 | H | -0.197114 | 2.714664 | 0.000294 |
| 1 | H | -4.077014 | -0.57215 | -0.00047 |
| 6 | C | 2.726908 | -0.730285 | 0.000212 |
| 8 | O | 3.649965 | 0.079043 | 0.000254 |
| 1 | H | 2.879084 | -1.83248 | 0.000296 |

C8-Cl- PU_1H  $E_{tot}$ = -871.359156 a.u.
Gibbs free energy  $E_G$= -871.308481 a.u.

| | | | | |
|---|---|---|---|---|
| 7 | N | 2.832796 | 0.593994 | 0.000373 |
| 6 | C | 2.767002 | -0.78089 | 0.000256 |
| 7 | N | 1.647131 | -1.471771 | 0.000073 |
| 6 | C | 0.51038 | -0.725927 | -0.00000 |
| 6 | C | 0.505878 | 0.746548 | 0.000113 |
| 6 | C | 1.717171 | 1.401544 | 0.000308 |
| 7 | N | -0.791165 | 1.188252 | 0.000005 |
| 6 | C | -1.458137 | 0.017383 | -0.00019 |
| 7 | N | -0.759301 | -1.166705 | -0.00019 |
| 1 | H | 3.73087 | -1.295471 | 0.000328 |
| 1 | H | 3.750546 | 1.026949 | 0.000516 |
| 1 | H | 1.870345 | 2.481235 | 0.000414 |
| 17 | Cl | -3.183044 | -0.010062 | -0.00035 |

C2-Cl- PU_1H  $E_{tot}$ = -871.354833 a.u.
Gibbs free energy  $E_G$= -871.304418 a.u.

| | | | | |
|---|---|---|---|---|
| 7 | N | -0.965273 | 1.256414 | -0.00021 |
| 6 | C | -1.284298 | -0.082421 | -0.00009 |
| 7 | N | -0.429934 | -1.061912 | 0.000072 |
| 6 | C | 0.882333 | -0.686582 | 0.000139 |
| 6 | C | 1.31235 | 0.722882 | 0.000031 |
| 6 | C | 0.343523 | 1.695915 | -0.00015 |
| 7 | N | 2.684446 | 0.762615 | 0.000108 |
| 6 | C | 2.995573 | -0.55026 | 0.000359 |
| 7 | N | 1.963467 | -1.473783 | 0.000318 |
| 1 | H | -1.725571 | 1.928136 | -0.00035 |
| 1 | H | 0.502879 | 2.774841 | -0.00026 |
| 1 | H | 4.034638 | -0.885408 | 0.000448 |
| 17 | Cl | -3.004575 | -0.399889 | -0.00021 |

C8-F- PU_1H  $E_{tot}$ = -510.934568 a.u.
Gibbs free energy  $E_G$= -510.881123 a.u.

| | | | | |
|---|---|---|---|---|
| 7 | N | 2.413951 | 0.596893 | 0.000321 |
| 6 | C | 2.351342 | -0.776312 | 0.000205 |
| 7 | N | 1.231985 | -1.470084 | 0.000025 |
| 6 | C | 0.095163 | -0.72815 | -0.000047 |
| 6 | C | 0.086609 | 0.747072 | 0.000065 |
| 6 | C | 1.296019 | 1.403254 | 0.000258 |
| 7 | N | -1.213289 | 1.18379 | -0.000041 |
| 6 | C | -1.860307 | 0.010439 | -0.000235 |
| 7 | N | -1.174973 | -1.171309 | -0.000239 |
| 1 | H | 3.315795 | -1.289753 | 0.000275 |
| 1 | H | 3.330643 | 1.031951 | 0.000462 |
| 1 | H | 1.448059 | 2.482993 | 0.000364 |
| 9 | F | -3.19013 | -0.015337 | -0.000338 |

C2-F- PU_1H  $E_{tot}$ = -510.928373 a.u.
Gibbs free energy  $E_G$= -510.875401 a.u.

| | | | | |
|---|---|---|---|---|
| 7 | N | 1.469456 | 1.068095 | 0.000236 |
| 6 | C | 1.644023 | -0.28909 | 0.000117 |
| 7 | N | 0.722793 | -1.195048 | -0.000048 |
| 6 | C | -0.552911 | -0.698638 | -0.000112 |
| 6 | C | -0.848478 | 0.747548 | -0.000005 |
| 6 | C | 0.202292 | 1.628832 | 0.000176 |
| 7 | N | -2.213861 | 0.913196 | -0.000078 |
| 6 | C | -2.644048 | -0.362346 | -0.000332 |
| 7 | N | -1.699803 | -1.379937 | -0.000289 |
| 1 | H | 2.294665 | 1.658616 | 0.000374 |
| 1 | H | 0.143167 | 2.717421 | 0.000288 |
| 1 | H | -3.709324 | -0.60139 | -0.000417 |
| 9 | F | 2.946237 | -0.641847 | 0.000216 |

C8-H- PU_1H $E_{tot}$ = -411.722874 a.u.
Gibbs free energy $E_G$= -411.660423 a.u.

| | | | | |
|---|---|---|---|---|
| 7 | N | 1.998293 | 0.60907 | 0.000281 |
| 6 | C | 1.944534 | -0.76881 | 0.000166 |
| 7 | N | 0.831723 | -1.46826 | -2.5E-05 |
| 6 | C | -0.31401 | -0.73292 | -9.3E-05 |
| 6 | C | -0.33035 | 0.740831 | 0.00001 |
| 6 | C | 0.875838 | 1.404443 | 0.000212 |
| 7 | N | -1.63187 | 1.170039 | -0.00012 |
| 6 | C | -2.30446 | -0.00402 | -0.00017 |
| 7 | N | -1.58092 | -1.1785 | -0.00034 |
| 1 | H | 1.020069 | 2.486083 | 0.000314 |
| 1 | H | -3.39633 | -0.02647 | -0.00036 |
| 1 | H | 2.913982 | -1.27315 | 0.000243 |
| 1 | H | 2.912345 | 1.049889 | 0.000424 |

C2-H- PU_1H $E_{tot}$ = -411.722874 a.u.
Gibbs free energy $E_G$= -411.660423 a.u.

| | | | | |
|---|---|---|---|---|
| 7 | N | 1.998293 | 0.60907 | 0.000281 |
| 6 | C | 1.944534 | -0.76881 | 0.000166 |
| 7 | N | 0.831723 | -1.46826 | -2.5E-05 |
| 6 | C | -0.31401 | -0.73292 | -9.3E-05 |
| 6 | C | -0.33035 | 0.740831 | 0.00001 |
| 6 | C | 0.875838 | 1.404443 | 0.000212 |
| 7 | N | -1.63187 | 1.170039 | -0.00012 |
| 6 | C | -2.30446 | -0.00402 | -0.00017 |
| 7 | N | -1.58092 | -1.1785 | -0.00034 |
| 1 | H | 1.020069 | 2.486083 | 0.000314 |
| 1 | H | -3.39633 | -0.02647 | -0.00036 |
| 1 | H | 2.913982 | -1.27315 | 0.000243 |
| 1 | H | 2.912345 | 1.049889 | 0.000424 |

C8-Me- PU_1H $E_{tot}$ = -451.025819 a.u.
Gibbs free energy $E_G$= -450.939693 a.u.

| | | | | |
|---|---|---|---|---|
| 7 | N | -2.463188 | 0.595764 | 0.000055 |
| 6 | C | -2.397897 | -0.779803 | 0.000061 |
| 7 | N | -1.279177 | -1.471421 | 0 |
| 6 | C | -0.137902 | -0.727484 | 0.000003 |
| 6 | C | -0.133516 | 0.747381 | -0.000043 |
| 6 | C | -1.343978 | 1.401181 | -0.000013 |
| 7 | N | 1.163733 | 1.182722 | -0.000196 |
| 6 | C | 1.860814 | 0.017928 | 0.000342 |
| 7 | N | 1.128063 | -1.162006 | -0.000251 |
| 1 | H | -3.362826 | -1.292929 | 0.000072 |
| 1 | H | -3.380023 | 1.029767 | 0.000093 |
| 1 | H | -1.498113 | 2.481111 | -0.000028 |
| 6 | C | 3.352727 | -0.013089 | 0.00001 |
| 1 | H | 3.719219 | -0.555915 | 0.887249 |
| 1 | H | 3.718936 | -0.562167 | -0.883422 |
| 1 | H | 3.755306 | 1.00804 | -0.003381 |

C2-Me- PU_1H $E_{tot}$ = -451.023980 a.u.
Gibbs free energy $E_G$= -450.93801 a.u.

| | | | | |
|---|---|---|---|---|
| 7 | N | -1.382972 | 1.124998 | -0.000232 |
| 6 | C | -1.659978 | -0.231989 | -0.000302 |
| 7 | N | -0.713267 | -1.151004 | -0.000166 |
| 6 | C | 0.567893 | -0.692025 | -0.000004 |
| 6 | C | 0.918058 | 0.737319 | 0.000138 |
| 6 | C | -0.109275 | 1.649002 | -0.000012 |
| 7 | N | 2.28575 | 0.859988 | 0.000553 |
| 6 | C | 2.673493 | -0.433919 | -0.000269 |
| 7 | N | 1.699733 | -1.414138 | 0.000484 |
| 1 | H | -2.168557 | 1.766887 | -0.000302 |
| 1 | H | -0.012475 | 2.735827 | 0.000055 |
| 1 | H | 3.731228 | -0.705791 | 0.000427 |
| 6 | C | -3.112288 | -0.6212 | -0.000233 |
| 1 | H | -3.631451 | -0.232771 | 0.89304 |
| 1 | H | -3.63214 | -0.230849 | -0.892258 |
| 1 | H | -3.178726 | -1.715341 | -0.001339 |

C8-OMe- PU_1H $E_{tot}$ = -526.211017 a.u.
Gibbs free energy $E_G$= -526.120493 a.u.

| | | | | |
|---|---|---|---|---|
| 7 | N | -2.755709 | 0.822855 | -0.000366 |
| 6 | C | -2.871348 | -0.54508 | -0.000267 |
| 7 | N | -1.853396 | -1.380571 | -0.000094 |
| 6 | C | -0.624842 | -0.794983 | -0.000037 |
| 6 | C | -0.424089 | 0.668337 | -0.000136 |
| 6 | C | -1.536935 | 1.476835 | -0.000298 |
| 7 | N | 0.91863 | 0.928563 | -0.000019 |
| 6 | C | 1.43126 | -0.326608 | 0.000018 |
| 7 | N | 0.567358 | -1.403165 | 0.000159 |
| 1 | H | -3.894916 | -0.928298 | -0.000326 |
| 1 | H | -3.607028 | 1.373901 | -0.000488 |
| 1 | H | -1.548161 | 2.567056 | -0.00038 |
| 8 | O | 2.74064 | -0.581533 | 0.000374 |
| 6 | C | 3.609291 | 0.575768 | 0.000517 |
| 1 | H | 3.429621 | 1.186872 | 0.897913 |
| 1 | H | 4.627067 | 0.166623 | 0.00082 |
| 1 | H | 3.430093 | 1.186723 | -0.897075 |

C2-OMe- PU_1H $E_{tot}$ = -526.196289 a.u.
Gibbs free energy $E_G$= -526.10652 a.u.

| | | | | |
|---|---|---|---|---|
| 7 | N | 1.0487 | 1.02521 | 0.000126 |
| 6 | C | 1.228286 | -0.343589 | -0.000003 |
| 7 | N | 0.251568 | -1.216914 | -0.000103 |
| 6 | C | -1.003828 | -0.695667 | -0.000205 |
| 6 | C | -1.279598 | 0.75405 | -0.000034 |
| 6 | C | -0.210942 | 1.60862 | 0.000124 |
| 7 | N | -2.642966 | 0.945017 | 0.000152 |
| 6 | C | -3.092593 | -0.325282 | -0.001059 |
| 7 | N | -2.168774 | -1.357371 | 0.000101 |
| 1 | H | 1.855722 | 1.636426 | 0.000283 |
| 1 | H | -0.246226 | 2.698548 | 0.000303 |
| 1 | H | -4.162514 | -0.545146 | -0.000418 |
| 8 | O | 2.490855 | -0.840866 | 0.000157 |
| 6 | C | 3.605849 | 0.062269 | 0.000392 |
| 1 | H | 3.613579 | 0.69036 | 0.910052 |
| 1 | H | 4.496014 | -0.577749 | 0.000476 |
| 1 | H | 3.613846 | 0.690495 | -0.909172 |

C8-OMe- PU_1H  $E_{tot}$ = -526.210636 a.u.

Gibbs free energy  $E_G$= -526.120166 a.u.

| | | | | |
|---|---|---|---|---|
| 7 | N | -2.909529 | 0.344349 | -0.000431 |
| 6 | C | -2.657801 | -1.004138 | -0.000288 |
| 7 | N | -1.450268 | -1.535705 | -0.000087 |
| 6 | C | -0.428174 | -0.642347 | -0.000043 |
| 6 | C | -0.620633 | 0.821759 | -0.000184 |
| 6 | C | -1.913403 | 1.301119 | -0.000378 |
| 7 | N | 0.597942 | 1.433791 | -0.00008 |
| 6 | C | 1.430532 | 0.364859 | 0.000032 |
| 7 | N | 0.887246 | -0.90597 | 0.000182 |
| 1 | H | -3.540922 | -1.647374 | -0.000341 |
| 1 | H | -3.87748 | 0.647643 | -0.000576 |
| 1 | H | -2.216805 | 2.348161 | -0.000499 |
| 8 | O | 2.748906 | 0.572044 | 0.000358 |
| 6 | C | 3.584864 | -0.610915 | 0.000709 |
| 1 | H | 3.390528 | -1.216698 | -0.896773 |
| 1 | H | 4.613329 | -0.228845 | 0.000938 |
| 1 | H | 3.39006 | -1.216509 | 0.898217 |

C8-OH- PU_1H  $E_{tot}$ = -486.935811 a.u.

Gibbs free energy  $E_G$= -486.869894a.u.

| | | | | |
|---|---|---|---|---|
| 7 | N | 2.419491 | 0.615631 | 0.000308 |
| 6 | C | 2.370953 | -0.75581 | 0.000187 |
| 7 | N | 1.258402 | -1.462186 | -0.000001 |
| 6 | C | 0.111256 | -0.734372 | -0.000021 |
| 6 | C | 0.086337 | 0.742835 | 0.000097 |
| 6 | C | 1.289567 | 1.411362 | 0.000251 |
| 7 | N | -1.216784 | 1.156925 | -0.000013 |
| 6 | C | -1.871617 | -0.029512 | 0.000075 |
| 7 | N | -1.148458 | -1.1971 | -0.000266 |
| 1 | H | 3.340593 | -1.259774 | 0.000225 |
| 1 | H | 3.330978 | 1.060566 | 0.000432 |
| 1 | H | 1.432218 | 2.492189 | 0.000343 |
| 8 | O | -3.210916 | -0.090512 | -0.000515 |
| 1 | H | -3.523998 | 0.831198 | -0.000602 |

C8-OH- PU_1H  $E_{tot}$ = -486.936245 a.u.

Gibbs free energy  $E_G$= -486.870299 a.u.

| | | | | |
|---|---|---|---|---|
| 7 | N | 2.433062 | 0.571228 | 0.000292 |
| 6 | C | 2.35179 | -0.79824 | 0.000195 |
| 7 | N | 1.221384 | -1.477302 | 0.000026 |
| 6 | C | 0.094466 | -0.719562 | 0.000003 |
| 6 | C | 0.103221 | 0.757321 | 0.000086 |
| 6 | C | 1.32388 | 1.394963 | 0.000223 |
| 7 | N | -1.185832 | 1.214283 | -0.000002 |
| 6 | C | -1.872151 | 0.054445 | 0.000089 |
| 7 | N | -1.179306 | -1.141177 | -0.000261 |
| 1 | H | 3.308545 | -1.325991 | 0.000236 |
| 1 | H | 3.355284 | 0.993577 | 0.000407 |
| 1 | H | 1.492209 | 2.472 | 0.000301 |
| 8 | O | -3.213202 | 0.054743 | -0.000512 |
| 1 | H | -3.482805 | -0.880324 | -0.000809 |

C2-OMe- PU_1H  $E_{tot}$ = -526.208965 a.u.

Gibbs free energy  $E_G$= -526.118637 a.u.

| | | | | |
|---|---|---|---|---|
| 7 | N | 0.775122 | 1.532804 | 0.000254 |
| 6 | C | 1.297382 | 0.257452 | 0.000142 |
| 7 | N | 0.587924 | -0.843633 | -0.00006 |
| 6 | C | -0.765409 | -0.662273 | -0.000128 |
| 6 | C | -1.398884 | 0.667188 | -0.000022 |
| 6 | C | -0.586596 | 1.771239 | 0.000174 |
| 7 | N | -2.767575 | 0.502528 | -0.000212 |
| 6 | C | -2.879369 | -0.838106 | -0.000129 |
| 7 | N | -1.719192 | -1.599945 | -0.000405 |
| 1 | H | 1.431391 | 2.306268 | 0.000389 |
| 1 | H | -0.906501 | 2.813796 | 0.000258 |
| 1 | H | -3.856893 | -1.325021 | -0.000415 |
| 8 | O | 2.646844 | 0.267645 | 0.000219 |
| 6 | C | 3.281461 | -1.037825 | 0.000093 |
| 1 | H | 2.987773 | -1.601084 | -0.897446 |
| 1 | H | 4.356339 | -0.822125 | 0.000183 |
| 1 | H | 2.987668 | -1.601308 | 0.897457 |

C2-OH- PU_1H  $E_{tot}$ = -486.918429 a.u.

Gibbs free energy  $E_G$= -486.854697 a.u.

| | | | | |
|---|---|---|---|---|
| 7 | N | 1.45011 | 1.080308 | 0.000235 |
| 6 | C | 1.66376 | -0.285815 | 0.000118 |
| 7 | N | 0.715805 | -1.18359 | -0.000046 |
| 6 | C | -0.555457 | -0.694228 | -0.000117 |
| 6 | C | -0.868456 | 0.747481 | -0.000007 |
| 6 | C | 0.175291 | 1.63176 | 0.000174 |
| 7 | N | -2.237186 | 0.902129 | -0.000074 |
| 6 | C | -2.653076 | -0.378159 | -0.000377 |
| 7 | N | -1.70004 | -1.386114 | -0.000262 |
| 1 | H | 2.241095 | 1.714915 | 0.000377 |
| 1 | H | 0.110854 | 2.720361 | 0.000289 |
| 1 | H | -3.716263 | -0.627414 | -0.000417 |
| 8 | O | 2.951163 | -0.728762 | 0.000211 |
| 1 | H | 3.581813 | 0.006875 | 0.000343 |

C2-OH- PU_1H  $E_{tot}$ = -486.932603 a.u.

Gibbs free energy  $E_G$= -486.867106 a.u.

| | | | | |
|---|---|---|---|---|
| 7 | N | 1.445227 | 1.107741 | 0.000181 |
| 6 | C | 1.665817 | -0.246305 | 0.000055 |
| 7 | N | 0.735701 | -1.167545 | -0.000047 |
| 6 | C | -0.545258 | -0.695288 | -0.000129 |
| 6 | C | -0.867215 | 0.744072 | 0.000017 |
| 6 | C | 0.167411 | 1.641913 | 0.000172 |
| 7 | N | -2.238354 | 0.885681 | 0.000182 |
| 6 | C | -2.644424 | -0.396369 | -0.000874 |
| 7 | N | -1.681975 | -1.397515 | 0.000039 |
| 1 | H | 2.255104 | 1.718536 | 0.000329 |
| 1 | H | 0.086747 | 2.72923 | 0.000342 |
| 1 | H | -3.705263 | -0.65503 | -0.000406 |
| 8 | O | 2.982771 | -0.567393 | 0.000206 |
| 1 | H | 3.019074 | -1.540268 | 0.000161 |

C8-NH2- PU_1H  $E_{tot}$ = -467.078817 a.u.
Gibbs free energy  $E_G$= -467.001666 a.u.

| | | | | |
|---|---|---|---|---|
| 7 | N | -2.44375 | 0.589642 | 0.00269 |
| 6 | C | -2.376093 | -0.778511 | 0.003045 |
| 7 | N | -1.25264 | -1.47066 | 0.000977 |
| 6 | C | -0.115094 | -0.725487 | -0.002372 |
| 6 | C | -0.108136 | 0.753059 | -0.002081 |
| 6 | C | -1.323059 | 1.403812 | 0.000475 |
| 7 | N | 1.184199 | 1.189835 | -0.000814 |
| 6 | C | 1.87207 | 0.015061 | -0.003665 |
| 7 | N | 1.148643 | -1.168223 | -0.003138 |
| 1 | H | -3.33813 | -1.297005 | 0.005342 |
| 1 | H | -3.361071 | 1.021617 | 0.004038 |
| 1 | H | -1.483021 | 2.48196 | 0.00082 |
| 7 | N | 3.230244 | -0.019373 | -0.039668 |
| 1 | H | 3.731798 | 0.838697 | 0.145314 |
| 1 | H | 3.685413 | -0.901424 | 0.151735 |

C2-NH2- PU_1H  $E_{tot}$ = -467.067709 a.u.
Gibbs free energy  $E_G$= -466.990713 a.u.

| | | | | |
|---|---|---|---|---|
| 7 | N | -1.420004 | 1.118265 | -0.016349 |
| 6 | C | -1.670248 | -0.241475 | -0.000483 |
| 7 | N | -0.725396 | -1.160614 | 0.007226 |
| 6 | C | 0.552851 | -0.692938 | -0.006119 |
| 6 | C | 0.889767 | 0.74198 | 0.008474 |
| 6 | C | -0.139592 | 1.644722 | -0.002758 |
| 7 | N | 2.261762 | 0.874018 | 0.017874 |
| 6 | C | 2.656802 | -0.412734 | 0.004088 |
| 7 | N | 1.687803 | -1.404535 | -0.009559 |
| 1 | H | -2.206264 | 1.751352 | -0.110466 |
| 1 | H | -0.051636 | 2.731866 | -0.007669 |
| 1 | H | 3.716008 | -0.679296 | 0.00392 |
| 7 | N | -3.010703 | -0.614643 | -0.069347 |
| 1 | H | -3.1069 | -1.621003 | 0.040545 |
| 1 | H | -3.642923 | -0.107697 | 0.54553 |

**Table S16.** Values of total energies and Gibbs free energies, cartesian coordinates of equilibrium geometries of NX substituted purine (PU) 9H, 7H, 3H and 1H tautomers.

N9-NO2- PU_9H  $E_{tot}$ = -616.134907 a.u
Gibbs free energy  $E_G$= -616.076861 a.u.

| | | | | |
|---|---|---|---|---|
| 7 | N | -3.03509 | -0.44948 | -0.0002 |
| 6 | C | -2.17337 | -1.48916 | -0.00037 |
| 7 | N | -0.82136 | -1.4556 | -0.00024 |
| 6 | C | -0.3507 | -0.21251 | 0.000043 |
| 6 | C | -1.13088 | 0.974354 | 0.000235 |
| 6 | C | -2.51907 | 0.794221 | 0.000208 |
| 7 | N | -0.32518 | 2.121217 | 0.000251 |
| 6 | C | 0.906596 | 1.675697 | -8E-06 |
| 7 | N | 0.971735 | 0.269562 | 0.000088 |
| 1 | H | -2.62143 | -2.48709 | -0.00075 |
| 1 | H | 1.816901 | 2.268613 | -7.8E-05 |
| 1 | H | -3.2146 | 1.64029 | 0.00022 |
| 7 | N | 2.21308 | -0.48359 | 0.000015 |
| 8 | O | 2.102086 | -1.69077 | 0.000965 |
| 8 | O | 3.223075 | 0.204243 | -0.0009 |

N7-NO2- PU_7H  $E_{tot}$ = -616.137609 a.u.
Gibbs free energy  $E_G$= -616.078701 a.u.

| | | | | |
|---|---|---|---|---|
| 7 | N | -2.28333 | -1.52189 | -0.00007 |
| 6 | C | -2.96115 | -0.35174 | -0.0002 |
| 7 | N | -2.46228 | 0.89968 | -0.00027 |
| 6 | C | -1.12684 | 0.945111 | 0.000016 |
| 6 | C | -0.31652 | -0.22572 | 0.000422 |
| 6 | C | -0.93815 | -1.4765 | 0.00033 |
| 7 | N | 0.987502 | 0.280011 | 0.000696 |
| 6 | C | 0.900854 | 1.68316 | 0.000156 |
| 7 | N | -0.33804 | 2.1064 | -0.0002 |
| 1 | H | -4.05232 | -0.43439 | -0.00044 |
| 1 | H | 1.801955 | 2.290814 | 0.000187 |
| 1 | H | -0.38821 | -2.41952 | 0.000324 |
| 7 | N | 2.209212 | -0.47021 | -5.4E-05 |
| 8 | O | 2.076692 | -1.68407 | -0.00032 |
| 8 | O | 3.235541 | 0.191485 | -0.00032 |

N9-CN- PU_9H $E_{tot}$ = -503.905930 a.u.
Gibbs free energy $E_G$= -503.848773 a.u.

| | | | | |
|---|---|---|---|---|
| 7 | N | 2.711185 | -0.49264 | -0.0002 |
| 6 | C | 1.854541 | -1.53745 | 0.000417 |
| 7 | N | 0.502193 | -1.50214 | 0.00008 |
| 6 | C | 0.04102 | -0.25871 | 0.000068 |
| 6 | C | 0.807555 | 0.931992 | -2.7E-05 |
| 6 | C | 2.19741 | 0.752092 | -0.00033 |
| 7 | N | -0.01706 | 2.062649 | 0.000579 |
| 6 | C | -1.23928 | 1.600189 | -0.00044 |
| 7 | N | -1.29892 | 0.179944 | 0.000133 |
| 1 | H | 2.306094 | -2.53354 | -0.00079 |
| 1 | H | -2.15992 | 2.180734 | -0.00057 |
| 1 | H | 2.895665 | 1.595636 | -0.00022 |
| 6 | C | -2.41303 | -0.57653 | 0.00004 |
| 7 | N | -3.40185 | -1.20673 | -0.00013 |

N7-CN- PU_7H $E_{tot}$ = -503.902987 a.u.
Gibbs free energy $E_G$= -503.845936 a.u.

| | | | | |
|---|---|---|---|---|
| 7 | N | -1.97322 | -1.57119 | 0.000239 |
| 6 | C | -2.64684 | -0.39544 | 0.000678 |
| 7 | N | -2.14725 | 0.854702 | -0.00012 |
| 6 | C | -0.80921 | 0.897519 | -0.00014 |
| 6 | C | -0.01222 | -0.27661 | 0.000049 |
| 6 | C | -0.62875 | -1.52577 | 0.000182 |
| 7 | N | 1.31614 | 0.189068 | -0.00018 |
| 6 | C | 1.226464 | 1.604986 | 0.000286 |
| 7 | N | -0.00426 | 2.042777 | -0.00078 |
| 1 | H | -3.73802 | -0.47686 | -0.00092 |
| 1 | H | 2.135154 | 2.204474 | 0.000302 |
| 1 | H | -0.07737 | -2.47197 | 0.000191 |
| 6 | C | 2.438779 | -0.55155 | -0.00019 |
| 7 | N | 3.418715 | -1.19742 | 0.000151 |

N9-CHO- PU_9H $E_{tot}$ = -525.017987 a.u.
Gibbs free energy $E_G$= -524.949910 a.u.

| | | | | |
|---|---|---|---|---|
| 7 | N | -2.84998 | -0.27953 | 0.00008 |
| 6 | C | -2.09967 | -1.40133 | -0.00025 |
| 7 | N | -0.74964 | -1.49937 | -5.6E-05 |
| 6 | C | -0.16303 | -0.30511 | -2E-06 |
| 6 | C | -0.81661 | 0.951705 | -1.4E-05 |
| 6 | C | -2.21566 | 0.910699 | 0.000034 |
| 7 | N | 0.120609 | 1.995267 | 0.000416 |
| 6 | C | 1.290848 | 1.402654 | -0.00056 |
| 7 | N | 1.20841 | -0.00571 | -8.2E-05 |
| 1 | H | -2.64675 | -2.34858 | 0.000292 |
| 1 | H | 2.262306 | 1.891491 | -0.00076 |
| 1 | H | -2.82981 | 1.817351 | 0.000354 |
| 6 | C | 2.27873 | -0.92579 | 0.000101 |
| 8 | O | 3.442969 | -0.58227 | 0.00016 |
| 1 | H | 1.917104 | -1.97375 | 0.000477 |

N7-CHO- PU_7H $E_{tot}$ = -525.012837 a.u.
Gibbs free energy $E_G$= -524.945000 a.u.

| | | | | |
|---|---|---|---|---|
| 7 | N | -2.2512 | -1.40624 | -0.00012 |
| 6 | C | -2.78737 | -0.1637 | 0.00008 |
| 7 | N | -2.1474 | 1.021497 | 0.000005 |
| 6 | C | -0.81407 | 0.909777 | -0.00013 |
| 6 | C | -0.14466 | -0.34442 | -0.00001 |
| 6 | C | -0.90865 | -1.51048 | -0.00013 |
| 7 | N | 1.224542 | -0.02706 | 0.0001 |
| 6 | C | 1.287745 | 1.380942 | -0.00047 |
| 7 | N | 0.11308 | 1.961633 | 0.000434 |
| 1 | H | -3.88091 | -0.12029 | -6.4E-05 |
| 1 | H | 2.254284 | 1.879809 | -0.00051 |
| 1 | H | -0.48213 | -2.51954 | -0.00016 |
| 6 | C | 2.320547 | -0.90851 | 0.000509 |
| 8 | O | 3.476236 | -0.53713 | -0.00026 |
| 1 | H | 2.004444 | -1.97347 | 0.000747 |

N9-CHO- PU_9H $E_{tot}$ = -525.011084 a.u.
Gibbs free energy $E_G$= -524.943514 a.u.

| | | | | |
|---|---|---|---|---|
| 7 | N | 2.700881 | -0.5777 | -0.00015 |
| 6 | C | 1.783474 | -1.57017 | 0.000297 |
| 7 | N | 0.43605 | -1.46335 | 0.000196 |
| 6 | C | 0.031895 | -0.19575 | 0.000109 |
| 6 | C | 0.87661 | 0.944755 | -6.7E-05 |
| 6 | C | 2.253697 | 0.692441 | -0.00027 |
| 7 | N | 0.126027 | 2.129069 | 0.000128 |
| 6 | C | -1.1207 | 1.729613 | 0.000014 |
| 7 | N | -1.27719 | 0.326655 | 0.000171 |
| 1 | H | 2.179069 | -2.59018 | -0.00027 |
| 1 | H | -1.99378 | 2.381346 | 0.000046 |
| 1 | H | 2.995233 | 1.498522 | -0.00034 |
| 6 | C | -2.53034 | -0.33035 | -4.6E-05 |
| 8 | O | -2.68583 | -1.5284 | -0.00025 |
| 1 | H | -3.36208 | 0.411496 | -6.6E-05 |

N7-CHO- PU_7H $E_{tot}$ = -525.014519 a.u.
Gibbs free energy $E_G$= -524.946347 a.u.

| | | | | |
|---|---|---|---|---|
| 7 | N | -1.88575 | -1.6093 | -3.6E-05 |
| 6 | C | -2.62641 | -0.47678 | 0.000327 |
| 7 | N | -2.20093 | 0.801851 | -0.00001 |
| 6 | C | -0.86826 | 0.918443 | -6E-06 |
| 6 | C | 0.003922 | -0.20306 | -1.5E-05 |
| 6 | C | -0.54437 | -1.4866 | -0.00009 |
| 7 | N | 1.297238 | 0.342124 | -2.4E-05 |
| 6 | C | 1.117992 | 1.740918 | 0.000238 |
| 7 | N | -0.13539 | 2.116945 | -0.00025 |
| 1 | H | -3.71146 | -0.62086 | -0.00058 |
| 1 | H | 1.979837 | 2.407647 | 0.000273 |
| 1 | H | 0.061619 | -2.39564 | -0.00014 |
| 6 | C | 2.528699 | -0.32948 | -1E-06 |
| 8 | O | 2.636016 | -1.53901 | 0.000015 |
| 1 | H | 3.386315 | 0.378942 | -0.00012 |

N9-Cl- PU_9H  $E_{tot}$ = -871.323437 a.u.

Gibbs free energy  $E_G$= -871.274114 a.u.

| | | | | |
|---|---|---|---|---|
| 7 | N | 2.809599 | -0.45155 | 0.000055 |
| 6 | C | 1.958755 | -1.50413 | -0.00022 |
| 7 | N | 0.609271 | -1.48213 | 0.000049 |
| 6 | C | 0.130818 | -0.2401 | 0.000031 |
| 6 | C | 0.892761 | 0.960346 | -1.1E-05 |
| 6 | C | 2.283875 | 0.787158 | 0.000142 |
| 7 | N | 0.063297 | 2.084755 | -0.00049 |
| 6 | C | -1.16247 | 1.600452 | 0.000399 |
| 7 | N | -1.18279 | 0.205676 | 0.000188 |
| 1 | H | 2.420456 | -2.49597 | 0.000452 |
| 1 | H | -2.08944 | 2.170092 | 0.000536 |
| 1 | H | 2.974554 | 1.637788 | -4.3E-05 |
| 17 | Cl | -2.58963 | -0.79009 | -9.3E-05 |

N7-Cl- PU_7H  $E_{tot}$ = -871.320300 a.u.

Gibbs free energy  $E_G$= -871.271340 a.u.

| | | | | |
|---|---|---|---|---|
| 7 | N | 2.088335 | -1.53647 | 0.000143 |
| 6 | C | 2.750518 | -0.35108 | 0.000638 |
| 7 | N | 2.237895 | 0.890598 | 0.000189 |
| 6 | C | 0.896957 | 0.927437 | 0.0001 |
| 6 | C | 0.108943 | -0.26158 | 0.000053 |
| 6 | C | 0.745511 | -1.50349 | 0.000014 |
| 7 | N | -1.19361 | 0.208775 | 0.000015 |
| 6 | C | -1.15119 | 1.595261 | 0.00041 |
| 7 | N | 0.086123 | 2.058283 | -0.00041 |
| 1 | H | 3.842578 | -0.42197 | -0.00046 |
| 1 | H | -2.06664 | 2.18334 | 0.000416 |
| 1 | H | 0.203538 | -2.45517 | -0.00007 |
| 17 | Cl | -2.62442 | -0.77022 | -0.0004 |

N9-F- PU_9H  $E_{tot}$ = -510.856023 a.u.

Gibbs free energy  $E_G$= -510.804949 a.u.

| | | | | |
|---|---|---|---|---|
| 7 | N | 2.537585 | 0.088479 | 0.000031 |
| 6 | C | 1.990566 | -1.1506 | 0.000089 |
| 7 | N | 0.683792 | -1.48618 | -8.8E-05 |
| 6 | C | -0.09942 | -0.40807 | -6.3E-05 |
| 6 | C | 0.318931 | 0.953534 | 0.000103 |
| 6 | C | 1.707849 | 1.147092 | 0.000131 |
| 7 | N | -0.76982 | 1.828008 | 0.000535 |
| 6 | C | -1.83541 | 1.047527 | -0.00048 |
| 7 | N | -1.47495 | -0.29019 | -0.0002 |
| 7 | H | 2.696313 | -1.98659 | -0.00044 |
| 1 | H | -2.87927 | 1.352446 | -0.00061 |
| 1 | H | 2.152681 | 2.14852 | 0.000315 |
| 9 | F | -2.36679 | -1.3369 | 0.000009 |

N7-F- PU_7H  $E_{tot}$ = -510.854103 a.u.

Gibbs free energy  $E_G$= -510.803556 a.u.

| | | | | |
|---|---|---|---|---|
| 7 | N | -2.10207 | -1.17526 | -6.8E-05 |
| 6 | C | -2.45093 | 0.137951 | -0.00032 |
| 7 | N | -1.64747 | 1.213053 | -5.9E-05 |
| 6 | C | -0.33826 | 0.921031 | -1.9E-05 |
| 6 | C | 0.129391 | -0.43109 | 0.000002 |
| 6 | C | -0.79554 | -1.47883 | 0.000059 |
| 7 | N | 1.494405 | -0.26652 | 0.000026 |
| 6 | C | 1.81355 | 1.072087 | -0.00032 |
| 7 | N | 0.720356 | 1.821715 | 0.000304 |
| 7 | H | -3.52654 | 0.338936 | 0.000201 |
| 1 | H | 2.84591 | 1.41391 | -0.00036 |
| 1 | H | -0.50644 | -2.5346 | 0.000079 |
| 9 | F | 2.420137 | -1.29956 | 0.000247 |

N9-H-PU_9H  $E_{tot}$ = -411.741198 a.u.

Gibbs free energy  $E_G$= -411.678769 a.u

| | | | | |
|---|---|---|---|---|
| 7 | N | 2.133176 | 0.656495 | 0.000257 |
| 6 | C | 2.020379 | -0.69113 | 0.000211 |
| 7 | N | 0.89314 | -1.43447 | 0.000044 |
| 6 | C | -0.20576 | -0.67547 | -4.3E-05 |
| 6 | C | -0.24493 | 0.747737 | 0.000004 |
| 6 | C | 1.001414 | 1.387556 | 0.000157 |
| 7 | N | -1.56412 | 1.203032 | -9.8E-05 |
| 6 | C | -2.29031 | 0.100127 | -0.0002 |
| 7 | N | -1.53258 | -1.06594 | -0.00028 |
| 1 | H | 1.099483 | 2.478907 | 0.000216 |
| 1 | H | -3.37889 | 0.065738 | -0.00032 |
| 1 | H | 2.960683 | -1.25118 | 0.000252 |
| 1 | H | -1.87332 | -2.02021 | -0.00041 |

N7-H-AD_7H  $E_{tot}$ = -411.735954 a.u.

Gibbs free energy  $E_G$= -411.673762 a.u.

| | | | | |
|---|---|---|---|---|
| 7 | N | -2.12563 | 0.684039 | -0.00026 |
| 6 | C | -2.0303 | -0.66959 | -9.7E-05 |
| 7 | N | -0.92259 | -1.42993 | 0.000027 |
| 6 | C | 0.219001 | -0.72192 | 0.000092 |
| 6 | C | 0.234645 | 0.706284 | -5.1E-05 |
| 6 | C | -0.98209 | 1.390693 | -0.00022 |
| 7 | N | 1.579429 | 1.045547 | 0.000083 |
| 6 | C | 2.285114 | -0.14627 | 0.000431 |
| 7 | N | 1.516735 | -1.22169 | 0.000003 |
| 1 | H | -1.0606 | 2.484487 | -0.0003 |
| 1 | H | 3.374333 | -0.15507 | 0.000616 |
| 1 | H | -2.98376 | -1.20761 | -0.00027 |
| 1 | H | 1.97617 | 1.977259 | 0.000072 |

N9-Me- PU_9H $E_{tot}$ = -451.030304 a.u.
Gibbs free energy $E_G$= -450.943859 a.u.

| | | | | |
|---|---|---|---|---|
| 7 | N | -2.55503 | -0.05808 | 0.000154 |
| 6 | C | -1.91621 | -1.25073 | -0.00017 |
| 7 | N | -0.58657 | -1.48419 | -0.00017 |
| 6 | C | 0.120613 | -0.34977 | -0.00015 |
| 6 | C | -0.40918 | 0.972117 | 0.000028 |
| 6 | C | -1.80717 | 1.063223 | 0.000231 |
| 7 | N | 0.623242 | 1.908045 | -0.00024 |
| 6 | C | 1.723629 | 1.173729 | 0.000124 |
| 7 | N | 1.494738 | -0.1977 | -0.00023 |
| 1 | H | -2.55774 | -2.13743 | 0.000242 |
| 1 | H | 2.739912 | 1.567163 | 0.000115 |
| 1 | H | -2.33134 | 2.025359 | 0.000236 |
| 6 | C | 2.477373 | -1.27458 | 0.00023 |
| 1 | H | 2.349174 | -1.90076 | 0.895872 |
| 1 | H | 2.348364 | -1.90221 | -0.89427 |
| 1 | H | 3.482629 | -0.83262 | -0.00059 |

N7-Me- PU_7H $E_{tot}$ = -451.024895 a.u.
Gibbs free energy $E_G$= -450.937923 a.u.

| | | | | |
|---|---|---|---|---|
| 7 | N | -2.04051 | -1.27589 | 0.000107 |
| 6 | C | -2.4762 | 0.00967 | 0.000192 |
| 7 | N | -1.75065 | 1.141135 | -0.00014 |
| 6 | C | -0.42377 | 0.93226 | -0.00011 |
| 6 | C | 0.143416 | -0.37949 | 0.000028 |
| 6 | C | -0.71133 | -1.48289 | 0.000108 |
| 7 | N | 1.516125 | -0.18712 | -2.7E-05 |
| 6 | C | 1.702906 | 1.185054 | 0.000078 |
| 7 | N | 0.581882 | 1.890138 | -0.00043 |
| 1 | H | -3.56371 | 0.135731 | -0.00039 |
| 1 | H | 2.70767 | 1.607782 | 0.000058 |
| 1 | H | -0.35798 | -2.52104 | 0.000187 |
| 6 | C | 2.53953 | -1.22273 | 0.000179 |
| 1 | H | 2.445574 | -1.85388 | 0.898459 |
| 1 | H | 2.445482 | -1.85436 | -0.89775 |
| 1 | H | 3.527763 | -0.74332 | -5E-06 |

N9-OMe-PU_9H $E_{tot}$ = -526.152929 a.u.
Gibbs free energy $E_G$= -526.06453 a.u.

| | | | | |
|---|---|---|---|---|
| 7 | N | 2.754488 | -0.58466 | 0.113884 |
| 6 | C | 1.851843 | -1.57062 | -0.09667 |
| 7 | N | 0.513534 | -1.46141 | -0.23577 |
| 6 | C | 0.107819 | -0.19193 | -0.14963 |
| 6 | C | 0.932269 | 0.947993 | 0.072354 |
| 6 | C | 2.301804 | 0.680981 | 0.201876 |
| 7 | N | 0.173181 | 2.118408 | 0.110824 |
| 6 | C | -1.0717 | 1.712044 | -0.07136 |
| 7 | N | -1.17018 | 0.335562 | -0.22691 |
| 7 | H | 2.256713 | -2.58526 | -0.16048 |
| 1 | H | -1.95964 | 2.33958 | -0.11584 |
| 1 | H | 3.03621 | 1.475431 | 0.375536 |
| 1 | O | -2.33329 | -0.33761 | -0.53752 |
| 8 | C | -2.76995 | -1.14119 | 0.596549 |
| 6 | H | -3.70707 | -1.60103 | 0.256049 |
| 1 | H | -2.01924 | -1.91299 | 0.821701 |
| 1 | H | -2.95027 | -0.4938 | 1.470306 |

N7-OMe- PU_7H $E_{tot}$ = -526.143533 a.u.
Gibbs free energy $E_G$= -526.053804 a.u.

| | | | | |
|---|---|---|---|---|
| 7 | N | 2.320222 | -1.41318 | 0.000512 |
| 6 | C | 2.861286 | -0.16541 | 0.001195 |
| 7 | N | 2.23157 | 1.01935 | 0.001021 |
| 6 | C | 0.890334 | 0.923883 | 0.000029 |
| 6 | C | 0.223638 | -0.34033 | -0.00073 |
| 6 | C | 0.981755 | -1.51435 | -0.0005 |
| 7 | N | -1.11538 | -0.01189 | -0.00151 |
| 6 | C | -1.21537 | 1.368164 | -0.00137 |
| 7 | N | -0.02471 | 1.9624 | -0.00034 |
| 1 | H | 3.955406 | -0.12994 | 0.001972 |
| 1 | H | -2.1708 | 1.884154 | -0.00191 |
| 1 | H | 0.537729 | -2.51504 | -0.00105 |
| 8 | O | -2.10065 | -1.00391 | -0.00353 |
| 6 | C | -3.42659 | -0.45084 | 0.00383 |
| 1 | H | -3.60894 | 0.15342 | -0.90182 |
| 1 | H | -4.07936 | -1.33249 | 0.003685 |
| 1 | H | -3.6011 | 0.147633 | 0.91485 |

N7-OMe- PU_7H $E_{tot}$ = -526.141230 a.u.
Gibbs free energy $E_G$= -526.051497 a.u.

| | | | | |
|---|---|---|---|---|
| 7 | N | 1.860871 | -1.67661 | 0.000442 |
| 6 | C | 2.660919 | -0.5801 | 0.000523 |
| 7 | N | 2.295189 | 0.708238 | 0.000191 |
| 6 | C | 0.967221 | 0.908559 | 0.000017 |
| 6 | C | 0.023807 | -0.18039 | 0.000033 |
| 6 | C | 0.533469 | -1.48837 | 0.000222 |
| 7 | N | -1.20341 | 0.473071 | 0.00006 |
| 6 | C | -0.96739 | 1.827938 | -0.00044 |
| 7 | N | 0.324313 | 2.131129 | 0.000117 |
| 1 | H | 3.736877 | -0.78066 | 0.000296 |
| 1 | H | -1.79688 | 2.531867 | -0.00055 |
| 1 | H | -0.08894 | -2.38659 | 0.000437 |
| 8 | O | -2.52773 | 0.011744 | 0.000376 |
| 6 | C | -2.63253 | -1.42164 | -0.0011 |
| 1 | H | -2.18269 | -1.85348 | 0.907794 |
| 1 | H | -3.71548 | -1.60033 | -0.00121 |
| 1 | H | -2.18281 | -1.85157 | -0.91095 |

N9-OH- PU_9H $E_{tot}$ = -486.872939 a.u.
Gibbs free energy $E_G$= -486.809613 a.u.

| | | | | |
|---|---|---|---|---|
| 7 | N | 2.523059 | 0.043752 | 0 |
| 6 | C | 1.945214 | -1.17992 | -8E-06 |
| 7 | N | 0.626196 | -1.47137 | -3.6E-05 |
| 6 | C | -0.11607 | -0.36252 | -5.8E-05 |
| 6 | C | 0.33466 | 0.986924 | 0.000007 |
| 6 | C | 1.72923 | 1.133963 | 0.000049 |
| 7 | N | -0.74643 | 1.863983 | 0.000003 |
| 6 | C | -1.8189 | 1.080363 | 0.000005 |
| 7 | N | -1.4847 | -0.26158 | -1.2E-05 |
| 1 | H | 2.626979 | -2.03537 | -4.6E-05 |
| 1 | H | -2.85748 | 1.40303 | 0.000004 |
| 1 | H | 2.214938 | 2.115979 | 0.000035 |
| 8 | O | -2.38248 | -1.3195 | -0.00007 |
| 1 | H | -1.79636 | -2.10405 | 0.000912 |

N7-OH- PU_7H $E_{tot}$ = -486.868409 a.u.
Gibbs free energy $E_G$= -486.805107 a.u.

| | | | | |
|---|---|---|---|---|
| 7 | N | 2.082536 | -1.20873 | -0.00765 |
| 6 | C | 2.459118 | 0.095706 | 0.003501 |
| 7 | N | 1.680988 | 1.190874 | 0.008534 |
| 6 | C | 0.36549 | 0.923323 | 0.004628 |
| 6 | C | -0.13507 | -0.41465 | 0.003122 |
| 6 | C | 0.766407 | -1.48023 | -0.0078 |
| 7 | N | -1.50874 | -0.25005 | 0.021732 |
| 6 | C | -1.77707 | 1.105881 | -0.0063 |
| 7 | N | -0.6777 | 1.843183 | -0.00247 |
| 1 | H | 3.539284 | 0.272475 | 0.006439 |
| 1 | H | -2.80238 | 1.471079 | -0.02563 |
| 1 | H | 0.455916 | -2.53049 | -0.02358 |
| 8 | O | -2.45825 | -1.25793 | -0.11019 |
| 1 | H | -2.63964 | -1.55678 | 0.800296 |

N7-OH- PU_7H $E_{tot}$ = -486.863033 a.u.
Gibbs free energy $E_G$= -486.800056 a.u.

| | | | | |
|---|---|---|---|---|
| 7 | N | -2.08042 | -1.20465 | -4.8E-05 |
| 6 | C | -2.45583 | 0.101332 | -0.00057 |
| 7 | N | -1.6771 | 1.194777 | -0.00023 |
| 6 | C | -0.36038 | 0.932037 | -9.8E-05 |
| 6 | C | 0.139726 | -0.41202 | 0.00003 |
| 6 | C | -0.76552 | -1.47607 | 0.000124 |
| 7 | N | 1.508596 | -0.2453 | 0.000047 |
| 6 | C | 1.781922 | 1.101793 | -0.00035 |
| 7 | N | 0.679622 | 1.845055 | 0.000409 |
| 1 | H | -3.5359 | 0.277748 | 0.000316 |
| 1 | H | 2.80745 | 1.464916 | -0.00033 |
| 1 | H | -0.46853 | -2.53252 | 0.000289 |
| 8 | O | 2.513027 | -1.21923 | 0.000494 |
| 1 | H | 2.038374 | -2.06798 | -0.00033 |

N9-$NH_2$- PU_9H  $E_{tot}$ = -467.033124 a.u.
Gibbs free energy  $E_G$= -466.956984 a.u.

| | | | | |
|---|---|---|---|---|
| 7 | N | -2.55977 | 0.000158 | -7.8E-05 |
| 6 | C | -1.95688 | -1.21238 | 0.000259 |
| 7 | N | -0.63701 | -1.49099 | 0.000017 |
| 6 | C | 0.108928 | -0.38424 | 0.000024 |
| 6 | C | -0.37945 | 0.95263 | -5E-06 |
| 6 | C | -1.77335 | 1.093033 | -0.00018 |
| 7 | N | 0.673936 | 1.86926 | 0.000386 |
| 6 | C | 1.758014 | 1.118234 | -0.0003 |
| 7 | N | 1.492011 | -0.25282 | -0.00014 |
| 1 | H | -2.62661 | -2.07814 | -0.00043 |
| 1 | H | 2.78583 | 1.481794 | -0.00041 |
| 1 | H | -2.26034 | 2.074629 | -7.6E-05 |
| 7 | N | 2.398944 | -1.32127 | 0.000175 |
| 1 | H | 2.989935 | -1.25682 | -0.83082 |
| 1 | H | 2.990838 | -1.25558 | 0.830421 |

N7-$NH_2$- PU_7H  $E_{tot}$ = -467.031592 a.u.
Gibbs free energy  $E_G$= -466.955304 a.u.

| | | | | |
|---|---|---|---|---|
| 7 | N | -2.06876 | -1.23992 | -4.4E-05 |
| 6 | C | -2.47113 | 0.05698 | -0.00047 |
| 7 | N | -1.71559 | 1.168069 | -0.00011 |
| 6 | C | -0.39498 | 0.921898 | -4.6E-05 |
| 6 | C | 0.13426 | -0.40174 | -2E-06 |
| 6 | C | -0.74659 | -1.48454 | 0.000049 |
| 7 | N | 1.510939 | -0.23413 | 0.000033 |
| 6 | C | 1.740464 | 1.136477 | -0.00033 |
| 7 | N | 0.634201 | 1.860052 | 0.000354 |
| 1 | H | -3.55485 | 0.211959 | 0.000457 |
| 1 | H | 2.757392 | 1.529885 | -0.00036 |
| 1 | H | -0.41048 | -2.52655 | 0.000131 |
| 7 | N | 2.43607 | -1.29153 | 0.000337 |
| 1 | H | 3.028833 | -1.21395 | -0.82897 |
| 1 | H | 3.028888 | -1.21352 | 0.829568 |

N3-$NO_2$- PU_3H  $E_{tot}$ = -616.117010 a.u.
Gibbs free energy  $E_G$= - 616.058258 a.u.

| | | | | |
|---|---|---|---|---|
| 7 | N | -0.34809 | 2.373466 | 0.000179 |
| 6 | C | 0.739387 | 1.629408 | 0.000201 |
| 7 | N | 0.726203 | 0.255127 | 0.000199 |
| 6 | C | -0.46055 | -0.43268 | 0.000151 |
| 6 | C | -1.68192 | 0.383958 | 0.000124 |
| 6 | C | -1.57247 | 1.762835 | 0.00013 |
| 7 | N | -2.74773 | -0.48447 | -9.1E-05 |
| 6 | C | -2.154 | -1.68542 | 0.000575 |
| 7 | N | -0.75972 | -1.72892 | 0.000119 |
| 1 | H | 1.726339 | 2.089856 | 0.000215 |
| 1 | H | -2.72429 | -2.61544 | 0.000339 |
| 1 | H | -2.4466 | 2.420171 | -1.4E-05 |
| 7 | N | 2.135671 | -0.4869 | 0.000067 |
| 8 | O | 2.06469 | -1.68256 | -0.00095 |
| 8 | O | 3.0825 | 0.264903 | -0.00042 |

N1-$NO_2$- PU_1H  $E_{tot}$ = -616.120089 a.u.
Gibbs free energy  $E_G$= -616.061928 a.u.

| | | | | |
|---|---|---|---|---|
| 7 | N | 1.04705 | 0.057802 | -0.00015 |
| 6 | C | 0.635688 | 1.388564 | -2.5E-05 |
| 7 | N | -0.62326 | 1.748897 | 0.000165 |
| 6 | C | -1.53656 | 0.733598 | 0.000236 |
| 6 | C | -1.15379 | -0.68824 | 0.000112 |
| 6 | C | 0.178547 | -1.01567 | -8.3E-05 |
| 7 | N | -2.29706 | -1.45316 | 0.000244 |
| 6 | C | -3.25569 | -0.50881 | 0.000375 |
| 7 | N | -2.86802 | 0.827176 | 0.000432 |
| 1 | H | 1.439147 | 2.122864 | -9.6E-05 |
| 1 | H | -4.31462 | -0.7742 | 0.000536 |
| 1 | H | 0.612911 | -2.01337 | -0.00019 |
| 7 | N | 2.528665 | -0.23668 | -0.00036 |
| 8 | O | 2.818419 | -1.41379 | -0.00041 |
| 8 | O | 3.249297 | 0.738768 | -0.00038 |

N3-CN- PU_3H  $E_{tot}$ = -503.884158 a.u.
Gibbs free energy  $E_G$= -503.827426 a.u.

| | | | | |
|---|---|---|---|---|
| 7 | N | 0.109033 | 2.292427 | -8.4E-05 |
| 6 | C | 1.149605 | 1.506368 | 0.000095 |
| 7 | N | 1.072742 | 0.102319 | 0.000106 |
| 6 | C | -0.18667 | -0.47977 | 0.000037 |
| 6 | C | -1.35057 | 0.394688 | -0.00013 |
| 6 | C | -1.16005 | 1.763501 | -0.00033 |
| 7 | N | -2.46128 | -0.42412 | 0.000774 |
| 6 | C | -1.92293 | -1.65128 | 0.000362 |
| 7 | N | -0.52557 | -1.75579 | -0.00062 |
| 1 | H | 2.166129 | 1.904287 | -2.4E-05 |
| 1 | H | -2.53485 | -2.55422 | 0.000153 |
| 1 | H | -1.98663 | 2.47918 | 0.000188 |
| 6 | C | 2.211316 | -0.64484 | -5.2E-05 |
| 7 | N | 3.220951 | -1.23788 | -0.00021 |

N1-CN- PU_1H  $E_{tot}$ = -503.882294 a.u.
Gibbs free energy  $E_G$= -503.825574 a.u.

| | | | | |
|---|---|---|---|---|
| 7 | N | 1.432611 | -0.0258 | -0.00019 |
| 6 | C | 1.021369 | 1.328048 | -6.3E-05 |
| 7 | N | -0.22205 | 1.711225 | 0.000128 |
| 6 | C | -1.15823 | 0.716843 | 0.000204 |
| 6 | C | -0.80873 | -0.71613 | 0.000064 |
| 6 | C | 0.50977 | -1.07972 | -0.00013 |
| 7 | N | -1.97133 | -1.45084 | 0.000223 |
| 6 | C | -2.90626 | -0.48327 | 0.000322 |
| 7 | N | -2.48572 | 0.841911 | 0.000352 |
| 1 | H | 1.846041 | 2.043521 | -0.00013 |
| 1 | H | -3.97127 | -0.72255 | 0.000468 |
| 1 | H | 0.918791 | -2.09005 | -0.00023 |
| 6 | C | 2.762795 | -0.29786 | -0.00038 |
| 7 | N | 3.915358 | -0.51056 | -0.00054 |

N3-CHO- PU_3H $E_{tot}$ = -524.989977 a.u.
Gibbs free energy $E_G$= -524.922834 a.u

| | | | | |
|---|---|---|---|---|
| 7 | N | -0.23901 | 2.364611 | -8.8E-05 |
| 6 | C | 0.895032 | 1.712687 | -0.00017 |
| 7 | N | 1.03607 | 0.326184 | -7.6E-05 |
| 6 | C | -0.12367 | -0.43283 | 0.00005 |
| 6 | C | -1.40403 | 0.272238 | 0.000122 |
| 6 | C | -1.41654 | 1.652586 | 0.000051 |
| 7 | N | -2.39775 | -0.68434 | 0.00058 |
| 6 | C | -1.69966 | -1.82759 | -0.00045 |
| 7 | N | -0.30297 | -1.74651 | 0.000246 |
| 1 | H | 1.83612 | 2.268766 | -0.00025 |
| 1 | H | -2.18564 | -2.80449 | 0.000085 |
| 1 | H | -2.3407 | 2.237334 | 0.000224 |
| 6 | C | 2.37697 | -0.2152 | -0.00011 |
| 8 | O | 2.640104 | -1.38752 | -0.00018 |
| 1 | H | 3.126358 | 0.60757 | -0.00025 |

N1-CHO- PU_1H $E_{tot}$ = -524.996050 a.u.
Gibbs free energy $E_G$= -524.928256 a.u

| | | | | |
|---|---|---|---|---|
| 7 | N | -1.38263 | 0.198685 | 0.000154 |
| 6 | C | -0.84681 | 1.492327 | 0.000044 |
| 7 | N | 0.426391 | 1.779464 | -0.00013 |
| 6 | C | 1.266774 | 0.700302 | -0.0002 |
| 6 | C | 0.781797 | -0.69349 | -8.8E-05 |
| 6 | C | -0.56664 | -0.92737 | 0.00009 |
| 7 | N | 1.872832 | -1.53447 | -0.00021 |
| 6 | C | 2.893448 | -0.66057 | -0.00037 |
| 7 | N | 2.599691 | 0.700903 | -0.00033 |
| 1 | H | -1.59114 | 2.293146 | 0.000114 |
| 1 | H | 3.931874 | -0.99772 | -0.00046 |
| 1 | H | -1.07531 | -1.89128 | 0.00018 |
| 6 | C | -2.80558 | 0.048058 | 0.000332 |
| 8 | O | -3.36167 | -1.02515 | 0.000557 |
| 1 | H | -3.32406 | 1.029396 | 0.00048 |

N3-CHO- PU_3H $E_{tot}$ = -524.998093 a.u.
Gibbs free energy $E_G$= -524.930545 a.u

| | | | | |
|---|---|---|---|---|
| 7 | N | 0.343274 | 2.208838 | 0.000014 |
| 6 | C | 1.255045 | 1.266158 | -0.00019 |
| 7 | N | 0.979738 | -0.09619 | -0.00014 |
| 6 | C | -0.34619 | -0.48588 | 0.000016 |
| 6 | C | -1.37791 | 0.538466 | 0.000233 |
| 6 | C | -0.98888 | 1.865134 | 0.000311 |
| 7 | N | -2.59599 | -0.11238 | 0.000065 |
| 6 | C | -2.23859 | -1.40317 | -0.00032 |
| 7 | N | -0.87171 | -1.70492 | 0.000464 |
| 1 | H | 2.317097 | 1.514099 | -0.00023 |
| 1 | H | -2.97363 | -2.20929 | 0.000098 |
| 1 | H | -1.70433 | 2.692572 | 0.000193 |
| 6 | C | 2.041186 | -1.078 | -0.00014 |
| 8 | O | 3.209387 | -0.77191 | -0.00029 |
| 1 | H | 1.630592 | -2.1058 | -3.6E-05 |

N1-CHO- PU_1H $E_{tot}$ = -524.995159 a.u.
Gibbs free energy $E_G$= -524.927547

| | | | | |
|---|---|---|---|---|
| 7 | N | -1.34593 | -0.21817 | 0.000198 |
| 6 | C | -1.05368 | 1.152846 | 0.000066 |
| 7 | N | 0.153484 | 1.653318 | -0.00012 |
| 6 | C | 1.173393 | 0.743744 | -0.0002 |
| 6 | C | 0.9497 | -0.71264 | -6.5E-05 |
| 6 | C | -0.33682 | -1.17572 | 0.000134 |
| 7 | N | 2.173361 | -1.34551 | -0.0002 |
| 6 | C | 3.020458 | -0.3014 | -0.00038 |
| 7 | N | 2.486198 | 0.983196 | -0.00035 |
| 1 | N | -1.93497 | 1.794801 | 0.000137 |
| 1 | H | 4.102495 | -0.44658 | -0.00049 |
| 1 | H | -0.64623 | -2.22319 | 0.000239 |
| 6 | C | -2.7113 | -0.65173 | 0.000403 |
| 8 | O | -3.65724 | 0.099456 | 0.000404 |
| 1 | H | -2.78362 | -1.76111 | 0.000417 |

N3-Cl- PU_3H $E_{tot}$ = -871.312447 a.u.
Gibbs free energy $E_G$= -871.263308 a.u.

| | | | | |
|---|---|---|---|---|
| 7 | N | 0.008437 | 2.317039 | -0.00015 |
| 6 | C | 1.059333 | 1.520998 | -0.00011 |
| 7 | N | 0.953657 | 0.151991 | -4E-06 |
| 6 | C | -0.26434 | -0.45374 | 0.000052 |
| 6 | C | -1.44244 | 0.416952 | 0.000022 |
| 6 | C | -1.2476 | 1.791919 | -0.0001 |
| 7 | N | -2.54202 | -0.4037 | 0.000567 |
| 6 | C | -1.99838 | -1.63403 | -0.00017 |
| 7 | N | -0.61273 | -1.73586 | -8E-06 |
| 7 | H | 2.074639 | 1.918997 | -0.00018 |
| 1 | H | -2.612 | -2.53606 | 0.000015 |
| 1 | H | -2.07918 | 2.502376 | 0.000143 |
| 17 | Cl | 2.430921 | -0.82613 | -5.8E-05 |

N1-Cl- PU_1H $E_{tot}$ = -871.305480 a.u.
Gibbs free energy $E_G$= -871.256569 a.u.

| | | | | |
|---|---|---|---|---|
| 7 | N | 1.292863 | 0.008534 | -0.00015 |
| 6 | C | 0.910976 | 1.33877 | -3.6E-05 |
| 7 | N | -0.34706 | 1.712556 | 0.000132 |
| 6 | C | -1.27926 | 0.723028 | 0.000203 |
| 6 | C | -0.92517 | -0.7034 | 0.000085 |
| 6 | C | 0.411337 | -1.04747 | -9.6E-05 |
| 7 | N | -2.07292 | -1.44736 | 0.000145 |
| 6 | C | -3.0212 | -0.47997 | 0.000462 |
| 7 | N | -2.61594 | 0.839141 | 0.000322 |
| 1 | H | 1.727823 | 2.061672 | -0.00011 |
| 1 | H | -4.08312 | -0.73372 | 0.000488 |
| 1 | H | 0.825882 | -2.05454 | -0.00021 |
| 17 | Cl | 3.008866 | -0.35584 | -0.00041 |

N3-F- PU_3H  $E_{tot}$ = -510.848903 a.u.
Gibbs free energy  $E_G$= -510.797916 a.u.

| | | | | |
|---|---|---|---|---|
| 7 | N | 1.455888 | 1.68292 | 0.00006 |
| 6 | C | 1.915763 | 0.443999 | -0.00039 |
| 7 | N | 1.079207 | -0.63247 | -0.00028 |
| 6 | C | -0.27044 | -0.52851 | -1E-06 |
| 6 | C | -0.78924 | 0.843868 | 0.000296 |
| 6 | C | 0.112002 | 1.902545 | 0.000496 |
| 7 | N | -2.1568 | 0.740379 | -0.00043 |
| 6 | C | -2.36235 | -0.58893 | -0.00025 |
| 7 | N | -1.25175 | -1.42518 | 0.000801 |
| 7 | H | 2.982679 | 0.217973 | -0.0004 |
| 1 | H | -3.36544 | -1.01777 | -8.4E-05 |
| 1 | H | -0.21407 | 2.94625 | 0.00001 |
| 9 | F | 1.675178 | -1.90486 | -0.00017 |

N1-F- PU_1H  $E_{tot}$ = -510.844843 a.u.
Gibbs free energy  $E_G$= -510.794099

| | | | | |
|---|---|---|---|---|
| 7 | N | 1.647114 | -0.16703 | -0.0002 |
| 6 | C | 1.406374 | 1.18318 | -0.00011 |
| 7 | N | 0.178172 | 1.658354 | 0.000048 |
| 6 | C | -0.82341 | 0.740237 | 0.000104 |
| 6 | C | -0.58881 | -0.71588 | 0.00003 |
| 6 | C | 0.718256 | -1.16671 | -0.00014 |
| 7 | N | -1.79094 | -1.36139 | 0.000056 |
| 6 | C | -2.65843 | -0.31749 | 0.000346 |
| 7 | N | -2.14825 | 0.960978 | 0.000388 |
| 7 | H | 2.2903 | 1.822088 | -0.00018 |
| 1 | H | -3.73723 | -0.48539 | 0.000447 |
| 1 | H | 1.081232 | -2.19406 | -0.00026 |
| 9 | F | 2.982129 | -0.56878 | -0.00039 |

N3-H- PU_3H  $E_{tot}$ = -411.727345 a.u.
Gibbs free energy  $E_G$= -411.664858 a.u.

| | | | | |
|---|---|---|---|---|
| 7 | N | 2.072937 | 0.745955 | 0.000539 |
| 6 | C | 2.048809 | -0.57306 | 0.000429 |
| 7 | N | 0.901102 | -1.3226 | -9E-06 |
| 6 | C | -0.30896 | -0.69164 | -0.00031 |
| 6 | C | -0.327 | 0.761381 | -0.00014 |
| 6 | C | 0.888871 | 1.432181 | 0.000271 |
| 7 | N | -1.6492 | 1.149621 | -0.00059 |
| 6 | C | -2.29914 | -0.0285 | 0.000644 |
| 7 | N | -1.54133 | -1.19673 | -0.00084 |
| 1 | H | 0.958232 | 2.524362 | 0.000366 |
| 1 | H | -3.38904 | -0.08448 | 0.000104 |
| 1 | H | 2.985325 | -1.13528 | 0.000592 |
| 1 | H | 0.945502 | -2.34051 | -0.00015 |

N1-H- PU_1H  $E_{tot}$ = -411.722874 a.u.
Gibbs free energy  $E_G$= -411.660423 a.u.

| | | | | |
|---|---|---|---|---|
| 7 | N | 1.998293 | 0.60907 | 0.000281 |
| 6 | C | 1.944534 | -0.76881 | 0.000166 |
| 7 | N | 0.831723 | -1.46826 | -2.5E-05 |
| 6 | C | -0.31401 | -0.73292 | -9.3E-05 |
| 6 | C | -0.33035 | 0.740831 | 0.00001 |
| 6 | C | 0.875838 | 1.404443 | 0.000212 |
| 7 | N | -1.63187 | 1.170039 | -0.00012 |
| 6 | C | -2.30446 | -0.00402 | -0.00017 |
| 7 | N | -1.58092 | -1.1785 | -0.00034 |
| 1 | H | 1.020069 | 2.486083 | 0.000314 |
| 1 | H | -3.39633 | -0.02647 | -0.00036 |
| 1 | H | 2.913982 | -1.27315 | 0.000243 |
| 1 | H | 2.912345 | 1.049889 | 0.000424 |

N3-Me- PU_3H  $E_{tot}$ = -451.017870 a.u.
Gibbs free energy  $E_G$= -450.930743 a.u.

| | | | | |
|---|---|---|---|---|
| 7 | N | -1.2689 | 1.849171 | -0.00003 |
| 6 | C | -1.83101 | 0.653586 | 0.000013 |
| 7 | N | -1.15325 | -0.53917 | 0.000074 |
| 6 | C | 0.212758 | -0.4884 | 0.000019 |
| 6 | C | 0.879626 | 0.79907 | -3.6E-05 |
| 6 | C | 0.093052 | 1.943477 | -3.2E-05 |
| 7 | N | 2.236326 | 0.555153 | -0.00005 |
| 6 | C | 2.291318 | -0.78928 | -0.00049 |
| 7 | N | 1.092142 | -1.49379 | 0.000229 |
| 1 | H | -2.92084 | 0.573639 | 0.000093 |
| 1 | H | 3.241552 | -1.32608 | -0.00023 |
| 1 | H | 0.515801 | 2.952788 | -0.00011 |
| 6 | C | -1.8413 | -1.83915 | 0.000196 |
| 1 | H | -1.54158 | -2.40459 | -0.89296 |
| 1 | H | -1.54118 | -2.40462 | 0.893191 |
| 1 | H | -2.92461 | -1.66651 | 0.000439 |

N1-Me- PU_1H  $E_{tot}$ = -451.009572 a.u.
Gibbs free energy  $E_G$= -450.921889 a.u.

| | | | | |
|---|---|---|---|---|
| 7 | N | -1.67365 | -0.15413 | 0.000273 |
| 6 | C | -1.33739 | 1.186998 | 0.000155 |
| 7 | N | -0.1123 | 1.666916 | -8.5E-05 |
| 6 | C | 0.877218 | 0.735878 | -0.00013 |
| 6 | C | 0.610062 | -0.70855 | -5.5E-05 |
| 6 | C | -0.70185 | -1.12835 | 0.000164 |
| 7 | N | 1.804247 | -1.38067 | -0.00045 |
| 6 | C | 2.691779 | -0.35745 | 0.000371 |
| 7 | N | 2.207619 | 0.932797 | -0.00078 |
| 1 | H | -2.18812 | 1.873412 | 0.000182 |
| 1 | H | 3.767459 | -0.54587 | -0.00043 |
| 1 | H | -1.03376 | -2.16796 | 0.000202 |
| 6 | C | -3.09976 | -0.52155 | 0.000454 |
| 1 | H | -3.59108 | -0.12065 | 0.899258 |
| 1 | H | -3.59131 | -0.12067 | -0.89823 |
| 1 | H | -3.18494 | -1.61449 | 0.000476 |

N3-OMe- PU_3H $E_{tot}$ = -526.143912 a.u.
Gibbs free energy $E_G$= -526.055290 a.u.

| | | | | |
|---|---|---|---|---|
| 7 | N | 0.28004 | 2.359251 | 0.082993 |
| 6 | C | -0.85311 | 1.704759 | -0.10474 |
| 7 | N | -0.93567 | 0.343441 | -0.21916 |
| 6 | C | 0.191026 | -0.42262 | -0.13789 |
| 6 | C | 1.451892 | 0.270605 | 0.082277 |
| 6 | C | 1.444349 | 1.657402 | 0.179287 |
| 7 | N | 2.434974 | -0.69237 | 0.128362 |
| 6 | C | 1.744287 | -1.83195 | -0.0514 |
| 7 | N | 0.366854 | -1.74311 | -0.22084 |
| 1 | H | -1.80574 | 2.229141 | -0.19344 |
| 1 | H | 2.234105 | -2.80681 | -0.06756 |
| 1 | H | 2.357609 | 2.239503 | 0.333299 |
| 8 | O | -2.18746 | -0.19998 | -0.51313 |
| 6 | C | -2.64337 | -1.06648 | 0.568536 |
| 1 | H | -2.71046 | -0.48986 | 1.505437 |
| 1 | H | -3.64102 | -1.38539 | 0.240188 |
| 1 | H | -1.96868 | -1.92756 | 0.671092 |

N1-OMe- PU_1H $E_{tot}$ = -526.138516 a.u.
Gibbs free energy $E_G$= -526.050245 a.u.

| | | | | |
|---|---|---|---|---|
| 7 | N | 1.271897 | 0.041568 | 0.309704 |
| 6 | C | 0.883339 | 1.354782 | 0.164942 |
| 7 | N | -0.36663 | 1.725412 | -0.01668 |
| 6 | C | -1.27826 | 0.717799 | -0.05699 |
| 6 | C | -0.92005 | -0.70249 | 0.101558 |
| 6 | C | 0.407924 | -1.02258 | 0.294505 |
| 7 | N | -2.05785 | -1.45871 | 0.026568 |
| 6 | C | -3.00377 | -0.50758 | -0.16736 |
| 7 | N | -2.60863 | 0.810498 | -0.22607 |
| 1 | H | 1.696905 | 2.079829 | 0.225093 |
| 1 | H | -4.05665 | -0.7765 | -0.27512 |
| 1 | H | 0.83924 | -2.01225 | 0.445815 |
| 8 | O | 2.626801 | -0.21363 | 0.53682 |
| 6 | C | 3.305796 | -0.47308 | -0.72212 |
| 1 | H | 3.234306 | 0.404596 | -1.38484 |
| 1 | H | 4.349719 | -0.65529 | -0.43493 |
| 1 | H | 2.880604 | -1.3639 | -1.21243 |

N3-OH- PU_3H $E_{tot}$ = -486.868000 a.u.
Gibbs free energy $E_G$= -486.803932 a.u.

| | | | | |
|---|---|---|---|---|
| 7 | N | -1.50236 | 1.658162 | 0.000008 |
| 6 | C | -1.93276 | 0.403854 | -0.00002 |
| 7 | N | -1.08333 | -0.66166 | -4.4E-05 |
| 6 | C | 0.256884 | -0.46648 | -2.4E-05 |
| 6 | C | 0.764006 | 0.884424 | -1.5E-05 |
| 6 | C | -0.16745 | 1.922564 | -1.6E-05 |
| 7 | N | 2.138815 | 0.782915 | 0.000103 |
| 6 | C | 2.346393 | -0.54751 | 0.000028 |
| 7 | N | 1.230386 | -1.37872 | -0.00015 |
| 1 | H | -2.99678 | 0.164056 | 0.000017 |
| 1 | H | 3.349741 | -0.97516 | 0.000044 |
| 1 | H | 0.122383 | 2.977179 | -4.5E-05 |
| 8 | O | -1.59751 | -1.95575 | 0.000071 |
| 1 | H | -0.78227 | -2.50603 | 0.000285 |

N1-OH- PU_1H $E_{tot}$ = -486.853390 a.u.
Gibbs free energy $E_G$= -486.790443 a.u.

| | | | | |
|---|---|---|---|---|
| 7 | N | -1.66575 | -0.16515 | -0.00524 |
| 6 | C | -1.38176 | 1.18371 | -0.01293 |
| 7 | N | -0.15675 | 1.663722 | -0.00367 |
| 6 | C | 0.839374 | 0.738844 | 0.005915 |
| 6 | C | 0.59257 | -0.71388 | -0.00402 |
| 6 | C | -0.71472 | -1.15384 | -0.01977 |
| 7 | N | 1.793645 | -1.3685 | -0.00262 |
| 6 | C | 2.667431 | -0.33258 | 0.009225 |
| 7 | N | 2.166081 | 0.950599 | 0.013905 |
| 1 | H | -2.25781 | 1.834384 | -0.03159 |
| 1 | H | 3.745328 | -0.50714 | 0.014326 |
| 1 | H | -1.0722 | -2.18329 | -0.04665 |
| 8 | O | -3.01194 | -0.54829 | -0.08329 |
| 1 | H | -3.29774 | -0.65588 | 0.843046 |

N3-OH- PU_3H $E_{tot}$ = -486.867999 a.u.
Gibbs free energy $E_G$= -486.803936 a.u.

| | | | | |
|---|---|---|---|---|
| 7 | N | 1.50276 | 1.65784 | -6.9E-05 |
| 6 | C | 1.933052 | 0.403605 | -0.00018 |
| 7 | N | 1.083279 | -0.66174 | -0.00011 |
| 6 | C | -0.25686 | -0.46632 | 0.000006 |
| 6 | C | -0.76378 | 0.884742 | 0.000122 |
| 6 | C | 0.16792 | 1.922632 | 0.000092 |
| 7 | N | -2.1387 | 0.783327 | 0.000362 |
| 6 | C | -2.34636 | -0.54703 | -0.00024 |
| 7 | N | -1.23048 | -1.37857 | 0.000179 |
| 1 | H | 2.997031 | 0.163654 | -0.00025 |
| 1 | H | -3.34977 | -0.97458 | 0.000018 |
| 1 | H | -0.12167 | 2.97736 | 0.000205 |
| 8 | O | 1.596477 | -1.95655 | -0.00015 |
| 1 | H | 0.780628 | -2.50587 | -5.7E-05 |

N1-OH- PU_1H $E_{tot}$ = -486.852982 a.u.
Gibbs free energy $E_G$= -486.790022 a.u

| | | | | |
|---|---|---|---|---|
| 7 | N | -1.66871 | -0.15297 | 0.000172 |
| 6 | C | -1.38033 | 1.190513 | 0.000058 |
| 7 | N | -0.14946 | 1.663061 | -7.1E-05 |
| 6 | C | 0.842382 | 0.739622 | -0.00016 |
| 6 | C | 0.590238 | -0.71132 | -1.5E-05 |
| 6 | C | -0.72234 | -1.14216 | 0.000148 |
| 7 | N | 1.783558 | -1.3732 | 0.000075 |
| 6 | C | 2.665641 | -0.33901 | -0.00079 |
| 7 | N | 2.173577 | 0.943419 | -3.5E-05 |
| 1 | H | -2.25189 | 1.846281 | 0.00015 |
| 1 | H | 3.742073 | -0.52207 | -0.00044 |
| 1 | H | -1.06387 | -2.17989 | 0.0003 |
| 8 | O | -3.03649 | -0.46222 | 0.000385 |
| 1 | H | -3.08068 | -1.4346 | 0.000459 |

N3-$NH_2$-PU_3H  $E_{tot}$ = -467.018518 a.u.
Gibbs free energy  $E_G$= -466.942304 a.u.

| | | | | |
|---|---|---|---|---|
| 7 | N | -1.29864 | 1.809859 | -2.1E-05 |
| 6 | C | -1.83895 | 0.608173 | 0.000186 |
| 7 | N | -1.1355 | -0.57734 | 0.000159 |
| 6 | C | 0.233649 | -0.52499 | 0.000019 |
| 6 | C | 0.866383 | 0.791167 | -0.00018 |
| 6 | C | 0.063545 | 1.921733 | -0.00027 |
| 7 | N | 2.227055 | 0.587595 | 0.000081 |
| 6 | C | 2.319641 | -0.75522 | 0.000088 |
| 7 | N | 1.144184 | -1.49685 | -0.00034 |
| 1 | H | -2.92658 | 0.494676 | 0.000232 |
| 1 | H | 3.285112 | -1.26445 | -0.00011 |
| 1 | H | 0.473514 | 2.935965 | -0.00014 |
| 7 | N | -1.78478 | -1.84242 | 0.000159 |
| 1 | H | -2.3817 | -1.88873 | 0.828918 |
| 1 | H | -2.3822 | -1.88847 | -0.82825 |

N1-$NH_2$-PU_1H  $E_{tot}$ = -467.018746 a.u.
Gibbs free energy  $E_G$= -466.942667 a.u.

| | | | | |
|---|---|---|---|---|
| 7 | N | -1.67314 | -0.18019 | 0.000204 |
| 6 | C | -1.36445 | 1.171215 | 0.000085 |
| 7 | N | -0.14541 | 1.660718 | -7.9E-05 |
| 6 | C | 0.853993 | 0.737588 | -0.00015 |
| 6 | C | 0.605099 | -0.71144 | -2.7E-05 |
| 6 | C | -0.70056 | -1.1523 | 0.000151 |
| 7 | N | 1.808761 | -1.36678 | -5.5E-05 |
| 6 | C | 2.682141 | -0.33274 | -0.00048 |
| 7 | N | 2.180139 | 0.951859 | -0.00024 |
| 1 | H | -2.23215 | 1.836769 | 0.000161 |
| 1 | H | 3.760255 | -0.50627 | -0.00045 |
| 1 | H | -1.05087 | -2.18415 | 0.000276 |
| 7 | N | -3.02248 | -0.62845 | 0.000408 |
| 1 | H | -3.48471 | -0.24015 | 0.826298 |
| 1 | H | -3.48492 | -0.24025 | -0.82541 |
